# SIRT Family: Biological Functions and Therapeutic Targets

**DOI:** 10.1002/mco2.70866

**Published:** 2026-07-22

**Authors:** Jia‐Yi Wang, Feng‐Li Jiang, Fang‐Yuan Zhang, Dong‐Hui Huang, Xiao‐Ying Li, Song Gao, Hua You, Qi‐Jun Wu, Huan‐Huan Chen, Ting‐Ting Gong

**Affiliations:** ^1^ Department of Obstetrics and Gynecology Liaoning Institute of Birth Health and Development Reproductive Hospital of China Medical University Liaoning China; ^2^ Department of Obstetrics and Gynecology Shengjing Hospital of China Medical University Shenyang China; ^3^ Department of General Surgery Shengjing Hospital of China Medical University Shenyang China; ^4^ Department of Clinical Epidemiology Shengjing Hospital of China Medical University Shenyang China; ^5^ Department of Pediatric Oncology Sichuan Clinical Research Center for Cancer Sichuan Cancer Hospital & Institute, Sichuan Cancer Center Affiliated Cancer Hospital of University of Electronic Science and Technology of China Chengdu China; ^6^ Department of Epidemiology School of Public Health China Medical University Shenyang China; ^7^ Key Laboratory of Environmental Stress and Chronic Disease Control & Prevention Ministry of Education China Medical University Shenyang China; ^8^ NHC Key Laboratory of Advanced Reproductive Medicine and Fertility (China Medical University) National Health Commission Shenyang China; ^9^ Department of Oncology Shengjing Hospital of China Medical University Shenyang China

**Keywords:** inflammation, metabolism, Sirtuins, transgenic mouse model, therapeutic targets

## Abstract

Sirtuins (SIRT1–SIRT7) are nicotinamide adenine dinucleotide (NAD^+^) dependent deacylases that serves as metabolic sensors, coupling cellular energy status to chromatin structure, mitochondrial function, and stress responses. Dysregulated SIRT activity has been extensively studied in aging, metabolic syndrome, cardiovascular disease, neurodegeneration, cancer, and immune disorders. However, robust human evidence and SIRT‐targeted therapies are lacking. Transgenic mouse models serve as key platforms to study gene function and guide therapeutic development. This review synthesizes evidence from *Sirt1–7* transgenic mouse models regarding the core cellular processes governed by SIRTs: metabolism, genome integrity, stress resistance, immunity, and autophagy, and illustrates their operation across different organ systems. By comparing global, tissue‐specific, and inducible knockout (KO) and overexpression (OE) models of cardiovascular, respiratory, digestive, nervous, endocrine, urogenital, musculoskeletal, malignant, and immune diseases, we identified central regulatory SIRTs (SIRT1, SIRT3, and SIRT6), context‐dependent modifiers (SIRT2, SIRT4, SIRT5, and SIRT7), and their organ‐ and cell type‐specific functions. We also summarize representative small‐molecule SIRT activators, inhibitors, and degraders, covering both clinical and preclinical studies, and highlight where contradictions and knowledge gaps remain. Together, these analyses help clarify which aspects of SIRT modulation are most promising and under what isoform, tissue, and disease contexts they should be pursued for the development of SIRT‑targeted therapies in human disease.

## Graphical abstract

SIRT1–SIRT7 networks from transgenic mice to human‑relevant therapeutic targets. SIRT1–SIRT7 form an isoform‑, organ‑, and disease‑specific regulatory network. Transgenic Sirt1–7 mouse models define central regulatory SIRTs (SIRT1, SIRT3, SIRT6), context‑dependent modifiers (SIRT2, SIRT4, SIRT5, SIRT7), and their key mechanisms and target organs. These findings clarify the functions of SIRTs and may be exploited for the treatment of human diseases.

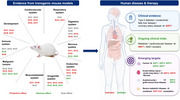

## 1 Introduction

SIRTs are a highly evolutionarily conserved protein family comprising NAD^+^‐dependent enzymes that catalyze protein lysine deacylation and mono‐adenosine diphosphate (ADP)‐ribosylation. They are present in organisms ranging from prokaryotes to eukaryotes [1]. They have attracted significant attention since the discovery that the yeast SIRT, silent information regulator 2, can extend lifespan [2]. Evolutionary evidence from zebrafish suggests that SIRTs arose during early vertebrate evolution [3]. In humans, the SIRT family consists of seven members, SIRT1–SIRT7 [1], and is characterized by a conserved catalytic core domain flanked by N‐ and/or C‐terminal sequences of variable length and sequence [4, 5]. The conserved catalytic core is formed by a Rossmann fold and a small Zn2+‐binding domain. They create a deep cleft for substrate and oxidized NAD^+^ binding. The distinct N‐ and C‐terminal structures dictate their subcellular localization, mediate protein‒protein interactions, and contribute to substrate selection [6]. Accordingly, SIRT1, SIRT6, and SIRT7 are predominantly nuclear, SIRT2 is mainly cytoplasmic, and SIRT3, SIRT4, and SIRT5 are largely mitochondrial; this distribution drives their distinct functional roles.

Phylogenetic analysis groups mammalian SIRTs into four classes [1], each of which exhibit distinct enzymatic activities [6]. Class I, which includes SIRT1, SIRT2, and SIRT3, exhibits strong deacetylase activity, although SIRT2 has also been shown to possess demyristoylase activity [7, 8]; Class II, represented by SIRT4, displays mono‐ADP‐ribosyltransferase, deacetylase, lipoamidase, and decarbamylase activities and additionally catalyzes the removal of 3‐hydroxy‐3‐methyl‐glutaryl moieties from lysine residues [9, 10]. Class III, represented by SIRT5, is characterized by strong deglutarylase, desuccinylase, and demalonylase activities but only weak deacetylase activity [11, 12]. Class IV comprises two members: SIRT6 and SIRT7. SIRT6 functions as a mono‐ADP‐ribosyltransferase and a long‐chain fatty acid deacylase, while its activity as a deacetylase is weak [13, 14]. SIRT7 functions primarily as a deacetylase and desuccinylase [15] and has been shown to possess auto‐ADP‐ribosylation activity [16, 17].

Cellular SIRTs detect changes in NAD^+^/reduced NAD (NADH) levels and translate energy status into adaptive responses, thereby maintaining cellular and organismal homeostasis. They achieve this goal by orchestrating a wide array of fundamental processes, including metabolism, aging, genomic stability, inflammation, oxidative stress, and autophagy [18]. They are involved in cardiovascular and respiratory disease, neurodegeneration, cancer, immune disorders, and acute and chronic organ injury. However, most data come from single pathways in single tissues, either in cells or in animals, and organ‐to‐organ communication is rarely considered. Large‐scale, long‐term human studies on metabolism and aging are difficult to conduct, and most studies are sparse, heterogeneous, and largely correlative. Clinical SIRT‐directed therapies are lacking for most indications. Therefore, an integrated in vivo framework covering the full spectrum of SIRT functions is urgently needed to guide human therapy.

Although our previous systematic review [18] examined SIRT functions from the cellular level to the clinical level, key questions remain unresolved, notably whether cellular effects occur at the organ or organism level. These uncertainties complicate drug development because target selection, dosing, and safety windows cannot be reliably inferred from in vitro data alone. They also hinder disease treatment, as it is unclear in which tissues, stages, and patient subsets SIRT‑directed therapies will be beneficial rather than harmful. Therefore, in vivo models, particularly transgenic mouse models, have become indispensable platforms for research on SIRTs. Since the first SIRT (SIRT1) KO mouse was developed in 2003, a wide range of transgenic mouse models, including conventional KO, OE, tissue‐specific deletion, and conditional or inducible systems, have been generated to investigate SIRT functions. They have revealed where specific SIRTs are essential for development, metabolism, stress resistance, and organ integrity. In this review, we therefore place *Sirt1–7* transgenic mouse models at the center of our analysis, comprehensively review their in vivo functions, and integrate human observational and interventional data to discuss unresolved questions, future therapeutic directions, and challenges.

In this review, we first define five core cellular processes controlled by SIRTs: metabolism, genome integrity, stress resistance, immunity, and autophagy. We summarize how individual SIRT isoforms engage these axes in vivo, primarily on the basis of transgenic mouse data. Second, we mapped these processes onto major organ systems by systematically comparing global, tissue‐specific, and inducible *Sirt1–Sirt7* KO and OE models in cardiovascular, respiratory, digestive, nervous, endocrine, urogenital, musculoskeletal, malignant, and immune diseases. We highlight the consistencies, contradictions, and unresolved gaps between mouse models and human therapy. Third, we review representative small‐molecule SIRT activators, inhibitors, and degraders. We cover both preclinical studies and ongoing early‐phase clinical trials that target SIRTs. We then linked their actions back to the pathways defined in transgenic mice. Finally, we discuss the opportunities and limitations of extrapolating from mice to humans and outline priorities for future translational and clinical research on SIRT modulation.

## 2 Core Mechanisms of SIRT Action in Pathophysiology

In vitro and in vivo studies have confirmed that SIRTs act as nodal integrators that control metabolism, genome integrity, stress responses, immunity, and autophagy. However, it remains unclear under which context each function is dominant and how it interacts. Findings across laboratories, cell types, animal models, and human studies are often diverse or contradictory, causing considerable confusion. In this section, we focus on evidence from transgenic mouse models to delineate core SIRT functions. Unlike many other approaches, transgenic models more directly establish causal relationships, providing solid and reliable evidence. Establishing direct causality in humans is difficult, and studying gene function in humans is costly and time consuming. Therefore, findings from transgenic mouse models provide crucial insights into human SIRT biology.

### 2.1 Metabolic Master Regulators: Glucose/Lipid Metabolism and Mitochondrial Function

Glucose and lipid catabolism converge in mitochondria to fuel adenosine triphosphate (ATP) production via the tricarboxylic acid cycle (TCA) and oxidative phosphorylation. Systemic homeostasis emerges from crosstalk across the liver, adipose tissue, skeletal muscle, and pancreatic islets. Mouse models indicate that individual SIRTs play distinct, nonredundant, and functionally unequal roles along this axis.

SIRT1 is the most broadly acting regulator of systemic metabolism. Global *Sirt1* OE improves glucose tolerance, enhances hepatic insulin sensitivity, reduces white adipose mass, and decreases oxygen consumption [19, 20], whereas global loss produces a paradoxical hypermetabolic yet glucose‐intolerant phenotype under stress conditions [21, 22, 23, 24]. Tissue‐specific models have shown that hepatic SIRT1 constrains steatosis, dyslipidemia, and insulin resistance via Akt–forkhead box O1 (FOXO1)‐dependent control of gluconeogenesis and adipose triacylglyceride lipase‐dependent lipophagy [25, 26]. Adipose SIRT1 limits chronic low‐grade inflammation and adipogenesis through peroxisome proliferator‐activated receptor (PPAR) gamma deacetylation [27]. β‐Cell SIRT1 supports mitochondrial function and postprandial insulin secretion without major changes in body weight or food intake [28]. In contrast, the manipulation of SIRT1 in skeletal muscle has little effect on basal whole‐body glucose tolerance [29]. Together, these findings identify SIRT1 as a central metabolic regulator that acts most prominently in the liver, adipose tissue, and β‐cells.

SIRT2 and SIRT3 act as stress‐dependent regulators, with SIRT3 primarily being mitochondrial and SIRT2 being cytosolic/nuclear. SIRT2 has modest effects at baseline but becomes relevant under high‐fat conditions. Global *Sirt2* KO disrupts mitochondrial protein acetylation in muscle, impairs insulin sensitivity, and promotes secondary hepatic insulin resistance; centrally, SIRT2 loss is associated with hyperphagia and increased adiposity [30]. SIRT3, a major mitochondrial deacetylase, exerts relatively mild effects under basal conditions but provides robust protection under stress. Global *Sirt3* KO mice often appear metabolically normal at baseline but develop insulin resistance [31, 32, 33], β‐cell dysfunction [25], and dyslipidemia [25, 34, 35] and exhibit accelerated age‐associated phenotypes under high‐fat diet (HFD) or cold exposure [36], whereas *Sirt3* OE preserves mitochondrial function and cardiovascular and sensory integrity [37, 38]. In diet‐induced obesity models, pancreas‐specific *Sirt3* KO impaired glucose tolerance and promoted hepatic steatosis [39], whereas liver‐ or muscle‐specific manipulations often failed to produce clear systemic changes [40, 41, 42]. This pattern indicates that SIRT3 functions primarily as a mitochondrial stress regulator, while pancreatic islets serve as key mediators of its systemic effects.

SIRT4 and SIRT5 act as more specialized metabolic regulators. SIRT4 regulates mitochondrial branched‐chain amino acid metabolism and thereby fine‐tunes insulin secretion and lipid handling. Global *Sirt4* deficiency alters leucine flux, increases amino acid‐stimulated β‐cell insulin release, and gradually leads to hyperinsulinemia and age‐related insulin resistance [10, 43]. However, β‐cell‐specific deletion does not recapitulate these phenotypes, indicating that whole‐body effects involve crosstalk among multiple tissues [44]. In fasted 129/Sv mice, *Sirt4* deficiency increases hepatic NAD^+^ levels, upregulates PPARα target genes and fatty acid oxidation (FAO), and activates AMP‑activated protein kinase (AMPK)–PPAR gamma coactivator‐1 alpha (PGC‐1α)‐dependent mitochondrial biogenesis [45, 46]. SIRT5, a desuccinylase/demalonylase, is largely dispensable for basal glucose and lipid homeostasis; global KO or OE has minimal effects under standard conditions [47, 48, 49, 50], but under metabolic stress, it becomes functionally important. In obesity, hepatic SIRT5 OE demalonylates and desuccinylates glycolytic and FAO enzymes to attenuate steatosis [51], and *the* loss of *Sirt5* impairs CPS1‐dependent ureagenesis and lysine/tryptophan oxidation, linking nitrogen disposal to systemic metabolic adaptation [50, 52, 53].

SIRT6 and SIRT7 influence systemic metabolism balance through nuclear regulatory mechanisms, with SIRT6 acting as a chromatin deacetylase and SIRT7 modulating nucleolar transcription. Global *Sirt6* KO causes severe hypoglycemia, loss of subcutaneous fat, and early death [54, 55], whereas OE extends the lifespan of males [56] and improves age‐related glucose tolerance [56, 57], likely by reducing insulin‐like growth factor 1 (IGF1) signaling [49]. At the tissue level, β‐cell‐specific *Sirt6* KO impairs glucose‐stimulated insulin secretion through the derepression of Txnip [58], and liver‐ or muscle‐specific deletions predispose cells to steatosis, insulin resistance, and reduced glucose uptake under metabolic stress [59, 60]. SIRT7 helps maintain adipose stores and limit hepatic lipid accumulation. Global and adipose‐specific *Sirt7* KO results in the development of lipodystrophy and hepatic steatosis with cardiac mitochondrial dysfunction [61, 62, 63], and liver‐specific *Sirt7* KO leads to fatty liver owing to a failure to suppress endoplasmic reticulum (ER)‐stress‐driven lipogenesis [64]. In adipocytes, SIRT7 can inhibit SIRT1 autodeacetylation, indicating the intrafamily balancing of lipolysis and lipogenesis.

Overall, SIRT1 has the broadest function in metabolic regulation. SIRT3, SIRT6, and SIRT7 exert strong but more organ‐focused effects, whereas SIRT2, SIRT4, and SIRT5 act predominantly as context‐dependent modulators. These observations provide a global framework for understanding the distinct severity and tissue specificity of SIRT functions, which we will further illustrate in the context of disease pathogenesis.

### 2.2 Guardians of Genome Integrity: DNA Damage Repair and Genomic Stability

Genome stability is essential for preventing cancer, premature aging, and degenerative disease. SIRT1–SIRT7 regulate genome stability through distinct mechanisms.

Global *Sirt1‐*null embryos are nonviable, largely because of severely impaired DNA damage responses and diminished repair capacity [65]. In *p53‐*deficient mice, *Sirt1* haploinsufficiency markedly increases spontaneous tumor formation across multiple tissues [65], indicating that even partial SIRT1 loss can cooperate with checkpoint defects. In adult vessels, SIRT1 deficiency in vascular smooth muscle cells (VSMCs) increases genomic instability and accelerates atherosclerosis [66]. In addition to affecting canonical repair pathways, SIRT1 also modulates tumorigenesis through effects on autophagy and androgen signaling in the prostate [67], highlighting the crosstalk between genome surveillance and metabolic/hormonal axes.

SIRT2 constrains chromosomal instability by supporting proper mitosis. Two independent global *Sirt2* KO strains develop normally but have high incidences of spontaneous tumors, including liver and breast cancer (BC), at advanced ages [68, 69, 70, 71], accompanied by chromosomal aneuploidy and centrosome amplification. Mechanistically, SIRT2 deacetylates cell‐division cycle 20 and cadherin 1 to maintain anaphase‐promoting complex/cyclosome function, thereby preserving mitotic fidelity [70, 71].

SIRT3–SIRT5 contributes to genome stability more indirectly through mitochondrial and metabolic control. Global *Sirt3* KO does not consistently provoke overt nuclear genomic instability or a strong cancer predisposition, but under oxidative stress, these mice accumulate substantial mitochondrial DNA (mtDNA) damage and exhibit aggravated tissue injury in cardiomyopathy and lung fibrosis models [72, 73, 74]. SIRT4 and SIRT5 are more often linked to metabolic tumor suppression: SIRT4 inhibits mitochondrial glutamine metabolism and modulates oncogenic signaling [75, 76, 77], whereas *Sirt5* KO suggests a role in limiting oxidative DNA damage and hepatocarcinogenesis [78]. In both cases, it remains unclear whether their effects are driven by shifts in redox balance, changes in metabolic flux, or direct control of DNA repair.

SIRT6 stands out as a key genome stability factor with particularly strong in vivo support. Global *Sirt6* KO mice develop chromosomal instability and severe progeroid syndrome and die at approximately 4 weeks of age [55]. Mechanistically, SIRT6 coordinates DNA damage responses via epigenetic control of chromatin and posttranslational regulation of multiple repair factors [54, 55]. Its loss accelerates tumor initiation and metastasis in the liver, pancreas, and other tissues [79, 80], and SIRT6 can reprogram tumor metabolism with either suppressive effects [81] or, in selected contexts, promoting effects [82], emphasizing its context‐dependent role in cancer biology.

*Sirt7* KO mice display partial embryonic lethality, a progeroid‐like phenotype, and a shortened lifespan [62, 83], together with increased genomic instability and defective DNA repair [78], although the direct contribution of SIRT7‐dependent genome maintenance to specific tumor entities remains to be clarified.

Taken together, these findings suggest that SIRT1, SIRT6, and SIRT7 are primary nuclear genome guardians; SIRT2 preserves the accuracy of mitosis, and SIRT3–SIRT5 indirectly support genome stability through controlling mtDNA integrity and oxidative stress. These functions collectively contribute to their roles in cancer, aging, and degenerative disease.

### 2.3 Orchestrators of Cellular Stress Resilience: Oxidative Stress, ER Stress, Hypoxia, and Ferroptosis

Cellular fate under stress is determined by the integrated response to multiple, interconnected insults. Oxidative stress, ER stress, hypoxia, and ferroptosis are tightly interconnected and together determine cell fate under injury and disease. Oxidative stress reflects an imbalance between reactive oxygen species (ROS) production and antioxidant defenses; ER stress triggers the unfolded protein response and, if unresolved, cell death; hypoxia stabilizes hypoxia‑inducible factors (HIFs) and rewires transcription; and ferroptosis is an iron‐dependent, lipid‐peroxidation‐driven form of regulated cell death. Different SIRTs intersect these pathways at different levels. Thus, their specific functions help determine which types of stress most strongly influence cell survival or death.

In multiple tissue‐specific mouse models, SIRT1 has emerged as a key coordinator of cellular stress responses. In the heart, it attenuates ROS and ER stress, limiting dysfunction in several injury settings [84, 85, 86], and promotes developmental adaptation to hypoxia, reducing congenital heart defects [87]. In lung and testicular injury models, SIRT1 restrains ferroptosis and oxidative damage via the nuclear factor erythroid 2‐related factor 2 (NRF2)/heme oxygenase‐1 (HO‐1) axis [88, 89]. In the kidney, it reduces oxidative stress by deacetylating nuclear factor kappa‐B (NF‐κB) and signal transducer and activator of transcription 3 [90, 91] and counters hypoxia‐driven fibrosis by suppressing HIF‐2α [92]. In oocytes, SIRT1 mitigates oxidative injury and promotes female fertility [93]. Together, these findings reveal SIRT1 as a central node in stress‐response pathways that support tissue resilience.

SIRT3 contributes strongly but importantly to stress resistance as a mitochondrial stress defender. In the cardiovascular system, it counteracts arterial thrombosis, doxorubicin (DOX) cardiomyopathy, atherosclerosis, and hypertension via superoxide dismutase 2 (SOD2) deacetylation, mitochondrial ROS (mROS) reduction, and the maintenance of iron homeostasis [74, 94, 95, 96, 97, 98]. Beyond the heart, *Sirt3* protects macrophages during infection, attenuates radiation and kidney injury, preserves β‐cell survival, and supports cartilage homeostasis in osteoarthritis (OA), with many of these effects attributable to improved mitochondrial redox balance [99, 100, 101, 102, 103, 104, 105].

SIRT6 protects multiple organs from injury by modulating oxidative stress, ferroptosis, and related stress pathways. In the heart and vasculature, it limits NAD(P)H oxidase‐derived superoxide, supports antioxidant defenses, and reduces stress‐induced cell death, thereby preserving cardiac and endothelial function [106, 107]. In the liver, SIRT6 activation decreases oxidative and inflammatory burdens and improves outcomes in acute liver failure [108]. In the kidney, it represses BRCA1‐associated protein 1 to curb ferroptosis and attenuate cisplatin‐induced acute injury [109].

SIRT2, SIRT4, SIRT5, and SIRT7 modulate more specific stress contexts. SIRT2 protects against cold‐induced intestinal injury by preserving barrier integrity and modulating ER stress through FOXO1 acetylation [110]. SIRT4 alleviates severe acute pancreatitis (SAP) by regulating HIF‐1α/HO‐1‐mediated ferroptosis in *Sirt4* KO models [111]. SIRT5 suppresses liver cancer development by desuccinylating and inhibiting peroxisomal acyl‐CoA oxidase 1 (ACOX1), thereby limiting oxidative damage [78]. Studies in 4–6‐month‐old global *Sirt7* KO mice have indicated that SIRT7 alleviates ER stress and the proteotoxic load in the liver by deacetylating histone H3 lysine 18 (H3K18) and repressing ribosomal protein genes, thus protecting against fatty liver [62].

In summary, SIRT1 is a central coordinator of oxidative, ER, and hypoxic stress; SIRT3 is a key mitochondrial defender against oxidative stress and ferroptosis; and SIRT6 is an epigenetic integrator of multiple stress pathways. SIRT2, SIRT4, SIRT5, and SIRT7 act as context‐specific modulators in epithelial cells and metabolically active organs. These stress‐related roles intersect with those of the other four functional axes to mediate physiological outcomes.

### 2.4 Key Modulators of Inflammation and Immunity

SIRT couples cellular metabolism to innate and adaptive immunity and thereby shapes both acute and chronic inflammation. Mouse models reveal a layered organization. Nuclear SIRTs (mainly SIRT1, SIRT6, and SIRT7) act at the epigenetic/transcriptional level; mitochondrial SIRTs (SIRT3, SIRT5, and partly SIRT4) control immunometabolism and redox; and cytosolic SIRT2 modulates inflammatory signaling and cell behavior.

SIRT1 fine‐tunes immune homeostasis across organs, with its pro‐ or anti‐inflammatory effects shaped by cell type and disease context. In vascular, renal and hepatic tissues, it generally suppresses the activity of NF‐κB, signal transducer and activator of transcription 3, and NOD‑like receptor family pyrin domain‑containing 3 (NLRP3), thereby limiting injury‐induced inflammation, fibrosis, arterial stiffness, and aneurysm formation [90, 91, 112, 113, 114, 115, 116]. In the gut and skin, it helps maintain barrier integrity and reduces age‐related or allergen‐driven inflammation [117, 118], and it tends to restrain neuroinflammation after central nervous system injury [119]. In the respiratory tract, SIRT1 promotes T helper type 2 (Th2) responses in dendritic cells (DCs) during allergic asthma [120], but in macrophages, it suppresses allergic airway inflammation [121]. During viral infection, SIRT1 in DCs supports mitochondrial metabolism and autophagy to sustain Type I interferon production and antiviral defense [122, 123].

SIRT2 has a mixed, context‐dependent effect on inflammation and immunity. It promotes pathology in allergic asthma and colitis by driving macrophage activation and NF‐κB signaling [124, 125] and similarly exacerbates renal injury via mitogen‐activated protein kinase (MAPK) phosphatase‐1/MAPK and activator protein‐1 (AP‐1)‐mediated inflammation [126, 127]. In contrast, SIRT2 protects against obesity‐induced insulin resistance by modulating inflammatory pathways [30]. Furthermore, in macrophages, SIRT2 cooperates with SIRT3 to regulate immunometabolism, and their combined deficiency enhances anti‐inflammatory responses [69].

SIRT3 protects many organs mainly by maintaining mitochondrial homeostasis and limiting ROS‐driven inflammation. When *Sirt3* is deleted, chronic cardiovascular inflammation increases, leading to cardiac fibrosis, endothelial dysfunction, hypertension, atherosclerosis, and vascular aging [37, 95, 128]. In acute conditions, such as lung and liver injury, *Sirt3* deficiency also exacerbates damage by increasing mROS levels, increasing inflammasome activation, and promoting fibrosis and tissue injury [101, 129, 130, 131].

SIRT5 plays context‐dependent roles in innate immunity through distinct mechanisms. During sepsis and inflammatory diseases, it increases antibacterial defenses by promoting NF‐κB activation by increasing p65 acetylation, which antagonizes SIRT2‐mediated deacetylation [132]. Moreover, SIRT3 and SIRT5 redundantly restrain inflammatory cytokine release and bactericidal activity; their combined loss enhances these functions and increases resistance to Listeria infection [133].

SIRT6 is a broad regulator of immune homeostasis and inflammation. Global *Sirt6* KO causes lymphoid atrophy and lymphopenia [55]. In innate immunity, SIRT6 restrains macrophage‐driven inflammation in arthritis via FOXO1 deacetylation [134] and limits mast cell degranulation and anaphylaxis by dampening FcepsilonRI signaling [135]. It also inhibits vascular inflammation and protects against hepatic steatosis and acute liver failure through NRF2‐dependent antioxidant pathways [108, 136]. In the kidney, SIRT6 reduces dysfunction, inflammation, and apoptosis, and proximal‐tubule‐specific deletion worsens fibrosis [137, 138]. SIRT7 plays context‐dependent, sometimes opposing roles: its deficiency protects against cisplatin‐induced acute kidney injury (AKI) by inhibiting NF‐κB/p65‐driven renal inflammation [139, 140]; however, in cancer, its loss enhances antitumor immunity and suppresses tumor growth [141].

Overall, SIRT1, SIRT3, and SIRT6 function as central integrators of innate immunity and chronic inflammation, whereas SIRT2, SIRT5, and SIRT7 act as context‐sensitive switches that can either amplify or limit inflammatory responses. SIRT4 currently contributes mainly to specialized conditions such as the tumor microenvironment. This offers a useful framework for determining the functions of SIRT in infection, autoimmunity, allergy, and cardiometabolic inflammatory disease.

### 2.5 Promotion of Autophagy and Protein Homeostasis

Autophagy and proteostasis are interdependent cellular quality control systems that are essential for maintaining intracellular homeostasis. Autophagy clears damaged components via lysosomal degradation, while proteostasis regulates protein synthesis, folding, and turnover. Together, they enable adaptation to metabolic and oxidative stress. Their dysfunction is a hallmark of aging and contributes to neurodegenerative diseases, metabolic disorders, and cancer.

SIRT1 protects against autophagy across tissues. Its loss impairs stress‐induced autophagy, causing organ‐specific dysfunction, such as defective lipophagy and steatosis in the liver [142]; worsened cardiac aging [143], immune dysregulation [123], and infertility [23]; and reduced tumor suppression in the prostate [67]. Global KO leads to perinatal lethality with the accumulation of damaged organelles [144]. Mechanistically, SIRT1 maintains autophagic flux by directly deacetylating autophagy‐related protein 5/autophagy‐related protein 7/microtubule‐associated protein light chain 3 (LC3) [144], transcriptionally activating autophagy‒lysosome genes via FOXO1/transcription factor EB (TFEB) [145], and enabling tissue‐specific adaptation, such as perilipin‐1 deacetylation, for hepatic lipophagy [142]. These actions mechanistically link the metabolic, stress, and immune effects of SIRT1 described above to a common autophagy–proteostasis axis.

SIRT3 is a key mitochondrial deacetylase that protects against organ injury by promoting mitophagy. In various organ injury models, such as renal ischemia–reperfusion (I/R) [146], drug‐induced cardiomyopathy [147, 148, 149], septic cardiomyopathy [149], and age‐related cardiac dysfunction [150], the loss of SIRT3 impairs mitophagy, exacerbating cell death and organ damage. SIRT3 activates mitophagy directly by deacetylating FOXO3a to upregulate BCL2/adenovirus E1B 19 kDa interacting protein 3/NIX and by facilitating PTEN‐induced kinase 1 (PINK1)/Parkin pathway activation [149, 150]. It also fine‐tunes autophagic activity metabolically, for instance, by sustaining mitochondrial glycolysis to reduce cellular dependence on autophagy [151, 152] and by context‐dependently modulating the AMPK/mammalian target of rapamycin (mTOR) axis to prevent excessive autophagic activation and maintain metabolic homeostasis [153].

Other SIRTs act as context‐dependent modifiers of autophagy. SIRT2 generally restrains protective autophagy in chronic cold‐induced intestinal injury and in Alzheimer's disease (AD)‐like models by modulating FOXO1 and α‐tubulin acetylation and disrupting autophagic trafficking [110, 154] but can directly promote mitophagy via deacetylation of mitochondrial autophagy‐related protein 5 [155]. SIRT6 protects against age‐related jawbone loss by sustaining autophagic activity in bone marrow stromal cells [156]. SIRT7 has bidirectional, stress‐dependent effects. It promotes autophagic induction via transcriptional reprogramming under nutrient stress to support survival [16], while it inhibits excessive autophagy during acute tissue injury to facilitate repair [157].

Taken together, these findings suggest that autophagy provides a unifying mechanism through which SIRTs integrate their roles in metabolism, genome stability, stress resistance, and immune regulation. SIRT1 and SIRT3 occupy central positions on this axis, whereas SIRT2, SIRT5, SIRT6, and SIRT7 fine‐tune autophagic responses according to cellular context. SIRT4 modulates cellular metabolism and may thereby affect autophagy, but its direct regulatory role remains unclear.

In transgenic mouse models, SIRT1, SIRT3, and SIRT6 consistently function as central SIRTs, each engaging all five axes and producing strong, often pleiotropic, phenotypes when altered. SIRT7 increases tissuerestricted control over lipid metabolism, ER/proteotoxic stress, and the inflammatory tone. In contrast, SIRT2, SIRT4, and SIRT5 function mainly as context‐dependent modifiers, whose effects become most apparent under metabolic, oxidative, or inflammatory stress in specific cell types. Taken together, these findings suggest that the physiological and pathological actions of SIRT1–7 in transgenic mouse models reflect coordinated control of these five tightly interconnected processes rather than the influence of any single, isolated pathway.

## 3 SIRTs in Disease: Insights From Transgenic Mouse Models

Directly manipulating genes to study their function in the human body is not ethically or technically feasible. Studies in humans are largely correlative and cannot establish causal in vivo functions of the SIRT family under controlled conditions. Mice allow us to systematically delete or activate each *Sirt* gene from development to adulthood, defining which family members are essential for survival, which are dispensable at baseline but critical under stress, and what dosage of increased activity is tolerated. These whole‐body models provide the phenotypic hierarchy, background dependence, and aging‐related baseline changes that are impossible to obtain from human subjects. Only after this foundational knowledge is gained can we move to tissue‐specific and inducible models and eventually translate candidate findings to human cohorts.

In global *Sirt1–7* KO mice, in which each SIRT protein is individually deleted organism‑wide, a clear and stable phenotypic hierarchy is revealed. The key characteristics of these lines—including targeting strategy, strain background, survival, development, and multisystem organ function—are summarized in Table S1. *Sirt1*, *Sirt6*, and *Sirt*7 full‑deletion mice exhibit embryonic or perinatal lethality and widespread developmental defects [19, 21, 22, 55, 62, 83, 158, 159, 160]. These features shorten lifespan, prevent adult and late‐onset aging studies, and greatly limit their value for modeling chronic degenerative diseases. In addition, developmental compensation in global KOs can obscure the true contribution of each gene. In contrast, *Sirt2*, *Sirt3*, *Sirt4*, and *Sirt5* nulls are viable and fertile with minimal baseline phenotypes, and their survival into old age makes them well suited for mechanistic studies across development, aging, and diverse disease models [10, 43, 50, 68, 70, 71, 76, 77, 161, 162, 163]. To overcome the constraints of early lethality and developmental adaptation, tamoxifen‑inducible and tissue‑specific conditional systems are being increasingly used, as they allow temporal control (including late‑life induction) and reduce non‑cell‑autonomous confounds. Heterozygotes provide useful partial loss models when full deletion is impractical, and they can better mimic the gradual functional decline observed in aging.

Allele design critically shapes phenotypes. Classical gene‑targeting strategies involve the deletion of one or more exons to abolish protein expression, whereas knock‑in point mutations can selectively disrupt catalytic or interaction domains while preserving protein structure. SIRT1 illustrates these principles particularly well. Exons 4–6 encode a critical part of the catalytic domain and are common targets for inactivation. Three widely used *Sirt1* KO models include the following: McBurney's *Sirt1*‐null mice [21], in which exons 5–6 are deleted, resulting in complete loss of protein expression (null mutation); Cheng's *Sirt1* ∆E4 mice [22], in which exon 4 deletion generates a truncated, catalytically inactive protein; and McBurney's *Sirt1^Y/Y^* mice [23], in which an exon 5 point mutation is carried, resulting in the production of a full‐length but enzymatically inactive SIRT1 protein. *Sirt1‐null* and *Sirt1ΔE4* mice exhibit almost complete postnatal or perinatal lethality with widespread developmental, cardiac, and retinal defects [21, 22]. In contrast, *Sirt1^Y/Y^* mice survive into adulthood with milder craniofacial and adipose abnormalities and partial preservation of caloric restriction responses [23]. Together, these models highlight allele‑specific effects, noncatalytic functions of SIRT1 in vivo, and the strong impact of genetic design on phenotype. With respect to other SIRTs (e.g., SIRT2, SIRT3, and SIRT7), multiple KO lines targeting different exons generally result in complete loss of protein and broadly similar phenotypes, and true catalytic point mutants have been less explored. In principle, shorter deletions should perturb the genome and local regulatory architecture less than large or full‑gene KO does, whereas full‑gene deletions can also alter gene structure and neighboring elements in ways unrelated to protein function. Thus, minimal exon deletions and well‑designed point mutants represent valuable next‑generation tools for dissecting SIRT biology.

The background of a mouse strain is another major modification of its phenotype. Most *Sirt* KO lines have been generated on, or backcrossed to, 129/Sv or C57BL/6 backgrounds, with 129‑based strains more often used for developmental and aging studies and C57BL/6‑based strains for cardiometabolic, inflammatory, and cancer models. SIRT1 again provides a clear example: *Sirt1*‐null mice on an inbred 129/SvJ background show near‑complete postnatal lethality with severe growth retardation and multiorgan developmental defects [21], whereas outcrossing the same allele onto a 129/Sv‐CD1 mixed background rescues many homozygotes to adulthood but increases phenotypic variability [164, 165, 166, 167]. With respect to other SIRTs, strain‑dependent phenotypic differences have not been systematically compared, although *Sirt2* and *Sirt3* lines are typically maintained on C57BL/6 or C57BL/6J, *Sirt4*, *Sirt5*, and *Sirt6* on 129/Sv, and *Sirt7* nulls on mixed C57BL/6×129 backgrounds. Taken together, these observations underscore the need to consider strain effects carefully and to remain aware that gene functions in mice may not fully mirror those in humans.

Global OE strains provide a complementary view of SIRT function in vivo. Rather than uniformly extending lifespan or preventing disease, these models reveal dose‐dependent, tissue‐dependent, and context‐dependent effects of SIRT OE. The phenotypes of these different whole‑body OE lines are summarized in Table S2. As illustrated here, SirBACO (*Sirt1* Bacterial Artificial Chromosome Overexpressor) mice show a two‐ to threefold increase in *Sirt1* expression in most tissues (approximately sevenfold in the spleen) and are born at Mendelian ratios without overt developmental defects [19]. They display improved metabolic efficiency, with enhanced glucose tolerance, reduced hepatic glucose production and increased adiponectin, which is consistent with a lower diabetes risk. In a second *Sirt1* OE model, *Sirt1* cDNA is knocked into the β‐actin locus [*Sirt1*‐knock in (KI)], which has a contrasting profile: heterozygotes share improved glucose tolerance but are infertile, and homozygotes are nonviable [20]. In *Sirt1*‐KI mice, SIRT1 is overexpressed in white and brown adipose tissue, the brain and fibroblasts but not in the liver or muscle, and this pattern is associated with impaired adipocyte differentiation and marked insulin sensitivity. Because these changes are tightly linked to reduced adiposity, this model may mask direct effects of SIRT1 with secondary consequences of fat loss. Together, these systems illustrate that the outcomes of SIRT1 OE depend on expression level and tissue distribution: moderate and tissue‐balanced activation confers metabolic benefits, whereas excessive or mislocalized OE of SIRT1 impairs development and adipose tissue function.

OE models support a protective role of the SIRT family, particularly in aging and metabolic regulation. *Sirt2* OE extends lifespan in BubR1‐deficient backgrounds [168, 169]. With respect to SIRT3, hemizygous and homozygous OE lines on a C57BL/6J background show organ‑specific, age‑dependent benefits, including improved age‑related hearing and preserved vascular function with attenuated vascular aging [37, 38]. Global *Sirt5* OE is well tolerated, with normal growth and fertility but enhanced ammonia detoxification and widespread protein hypoacylation, which is consistent with a buffering role in nitrogen metabolism that may become especially important under metabolic or aging‑associated stress [47, 49]. *Sirt6* OE extends the lifespan of males and protects against HFD‐induced obesity and dyslipidemia, improving glucose tolerance and lipid handling without compromising survival, whereas these effects are weaker in females, indicating sex‐specific modulation of aging trajectories [56, 57]. *Sirt7* KO models display features of premature aging [62], but whether global *Sirt7* OE exerts bona fide antiaging effects remains to be determined.

**FIGURE 1:**
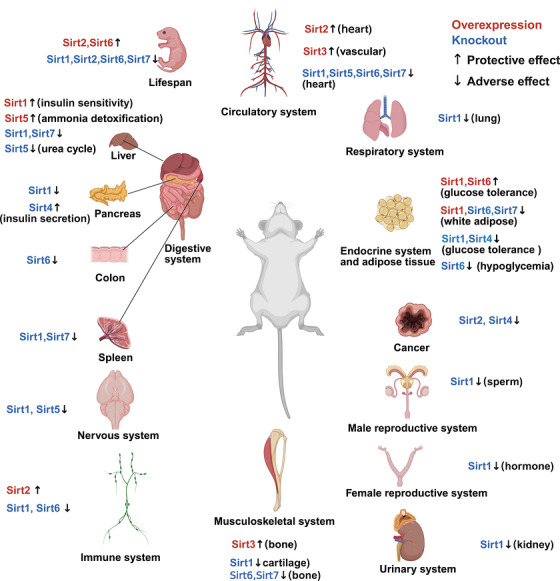
Schematic representation of the *Sirt* gene OE (red) and KO (blue) in mice. A comprehensive overview of how *Sirt* genes influence different aspects of mouse physiology and pathology is provided. Upward‐pointing arrows indicate protective effects; downward‐pointing arrows indicate adverse effects.

The major phenotypic outcomes of the whole‐body *Sirt1–7* KO and OE models are presented in Figure 1, which highlights that the organ systems are primarily affected by each manipulation. In these models, SIRT1, SIRT6, and, to a lesser extent, SIRT7 have emerged as critical regulators: their deletion causes embryonic or perinatal lethality with widespread developmental defects, whereas their OE confers context‐dependent metabolic or survival benefits. SIRT3 also plays important roles, particularly in stress resistance and age‑related functional preservation, although its loss is compatible with normal development. In contrast, SIRT2, SIRT4, and SIRT5 act primarily as stress‑dependent modulators. In the following organ‐ and disease‐focused sections, this framework is used to interpret how specific *Sirt1–7* manipulations translate into specific cardiovascular, respiratory, digestive, nervous, and other disease phenotypes along the five mechanistic axes. For a concise and comprehensive overview, Table 1 summarizes how each gene acts in specific diseases, particularly in tissue‐specific systems.

**TABLE 1:** Summary of *Sirt* genes’ role in diseases: evidence from transgenic mouse models.

	*Sirt1*	*Sirt2*	*Sirt3*	*Sirt4*	*Sirt5*	*Sirt6*	*Sirt7*
Cardiovascular system diseases							
Arrhythmia	↑S [85, 170]; ↑S+D [171]						
Myocardial metabolism and contractile function	↑S+D [86, 172, 173, 174]		↑G [175]; ↑G+D [176]		↑G+D [177]		
Cardiac fibrosis			↑S+D [94]↑;G+D [128];↑G [178]				
Cardiomyopathy	↑G [179]	↑G+D [180]	↑S+D [181]			↑S+D [182]	↑S+D [63, 183]
Myocardial infarction, ischemia‒reperfusion injury, and thrombosis	↑S+D [113, 184]; ↑S [185]		↑G+D [97, 186, 187]		↑G+D [188]		↑G+D [157]
Drug‐induced cardiac damage	↑S+D [84]		↑G+D [74]; ↓G+D [96, 148]			↑G+D [107]	
Other heart diseases	↑S+D [189]; ↓S+D [87]		↑S+D [190]; ↑G+D [191, 192]				
Arterial stiffness and atherosclerosis	↑G [193]; ↑G+D [116, 194]; ↑S+D [114, 195, 196, 197]	↑G+D [198]	↑S+D [95]			↑S+D [199, 200, 201, 202]	
Hypertension and an aortic aneurysm	↑S+D [203, 204]		↑G+D [37, 98, 205, 206]			↑G+D [207, 208]; ↑S+D [209, 210, 211]	
Metabolic and ischemic vascular diseases	↑S+D [212, 213]						
Respiratory system diseases							
Pulmonary fibrosis	↓G+D [214]		↑G+D [72, 73]				
COPD/emphysema	↑G+D [215]; ↑S+D [216]						
Asthma	↓S+D [120]; ↑S+D [121]	↑G+D [124]					
Pulmonary infection	↑S+D [122, 123, 217]		↑G+D [100, 101]				
Acute lung injury	↑G+D [88]		↑G+D [131, 218, 219]				
Digestive system diseases							
Nonalcoholic fatty liver disease	↑S+D [220]	↓G+D [221]	↓G [153]	↑G+D [10, 45, 46, 222, 223]	↑G+D [49, 52, 224, 225]; ↑S+D [51]	↑S+D [60, 226]	↑G+D [61]; ↓S+D [63]
Liver injury	↑S+D [112, 227]; ↓S+D [228]	↓G+D [229]	↑G+D [105, 129, 130]			↑S+D [108, 230]	↑S+D [231]
Inflammatory bowel disease	↑S+D [117, 232]; ↓S+D [233]	↑G+D [125]; ↓G+D [110, 234]				↑S+D [235]; ↓S+D [236]	
Severe acute pancreatitis				↑G+D [111]			
Nervous system diseases							
Parkinson's disease	↑G+D [237]	↓G+D [238, 239, 240]	↑G+D [241] [242]		↑G+D [243]		
Alzheimer's disease	↑G+D [244]	↓G+D [154, 245]	↑G+D [246]				
Huntington's disease	↑G+D [247]	‐G+D [248]					
Depression	↑S [249]	↓G+D [250]				↓S+D [251]	
Anxiety	↑S+D [252, 253]					↓S+D [251]	
Injury	↑S+D [119, 254]		↑S+D [255]				
Endocrine system diseases							
Diabetes mellitus	↑S+D [26, 91]	↑G+D [30, 256]	↑G+D [99, 103]	↑G+D [10, 43, 223]; ↑S+D [44, 257]		↑S+D [258, 259]	↓S+D [260, 261]
Hyperlipidemia	↑S+D [262]; ↑G+D		↑G+D [263]	↑G+D [45, 46]		↑G+D [57]	↓S+D [63]
Urogenital system diseases							
Acute kidney injury	↑G+D [145, 264]	↓G+D [127, 265]	↑G+D [102, 266, 267, 268, 269]		↓G+D [270]	↑G+D [109, 138]	↓G+D [139, 140]
Chronic kidney diseases	↑S+D [90, 92, 271, 272, 273]	↓G+D [126]				↑S+D [137, 274, 275]	
Female infertility	↑S [93, 276] [277]; ↑G [144, 278, 279]		↑G [280]				
Male infertility	↑S [23]						
Musculoskeletal system diseases							
Osteoporosis	↑S+D [281, 282, 283]; ↑G+D [284];		↓G+D [285, 286]			↑S [287]; ↑S+D [288]	
Osteoarthritis	↑S+D [286, 289]; ↑G [290]; ↑G+D		↑G [104]				
Age‐related sarcopenia pathogenesis	↑S [291]; ↑S+D [292]					↑G [293]	
Malignant tumors							
Breast cancer		↑G [294]		↑G+D [75]	↓G+D [295]	↓G+D [82]	
Non‐small cell lung cancer	↓G+D [296]						
Hepatocellular carcinoma and	↓S+D [297]	↑G [71]		↑G+D [76]	↑G [78]	↑G [80]	↓G+D [141]
Pancreatic ductal adenocarcinoma	↓S+D [298]					↑S+D [79]	
Other solid malignancies	↓G [67]				↓G+D [299]	↑S+D [81]	
Hematologic malignancies	↓S+D [300, 301]		↓G+D [302]	↑G+D [77]			
Immune system diseases							
Autoimmune diseases	↓S+D [303, 304, 305]	↓G+D [306]				↑G+D [134]	
Graft versus host disease	↓S+D [307]		↓G+D [308]				
Allergic and inflammatory diseases	↑S+D [118]					↑S+D [135]	
Infection and sepsis		↓G+D [69, 309, 310]	↑G+D [69, 133, 311, 312] ↓G+D [132, 133]		↑G+D [132, 133]		

Abbreviations: ↓: adverse effect; ↑: protective effect; –, no effect; G: global OE or KO model; S: tissue‐specific OE or KO model; D: disease‐based model.

### 3.1 SIRT Family Transgenic Mouse Models of Cardiovascular Disease

**FIGURE 2:**
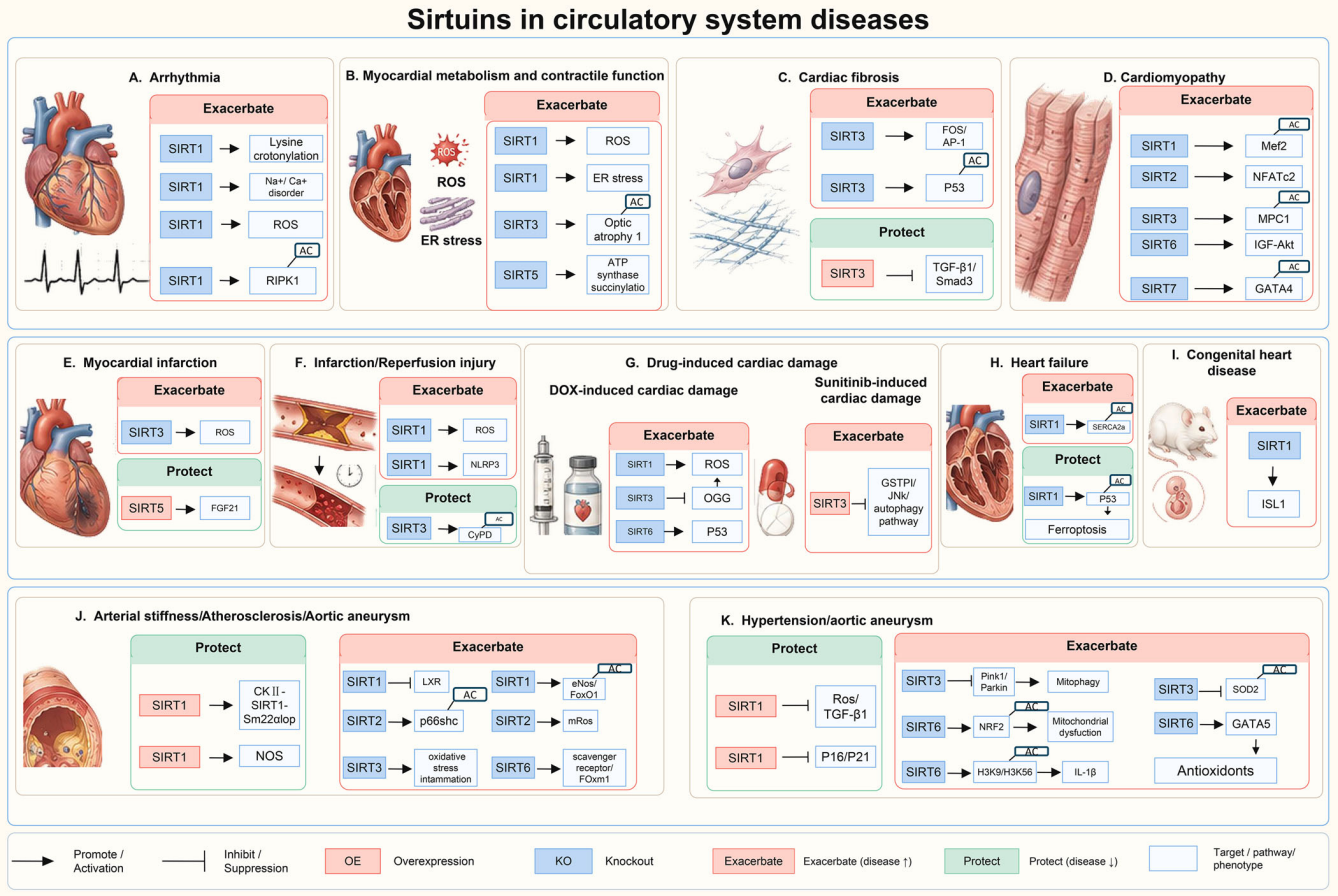
Sirtuins in circulatory system diseases. This schematic summarizes the protective (green) and detrimental (red) roles of sirtuins in cardiovascular and vascular pathologies on the basis of OE (pink) and KO (blue) mouse models. The arrows indicate activation/promotion, the blunt‐ended lines indicate inhibition/suppression, and “AC” denotes acetylation‐related regulation. (A and B) Arrhythmia and cardiac dysfunction and remodeling of SIRT1, SIRT3, and SIRT5 regulate arrhythmia and cardiac dysfunction via lysine crotonylation, Na^+^/Ca^2^^+^ imbalance, ROS generation, RIPK1, ER stress, OPA1, and ATP synthase succinylation. (C–G) Cardiac remodeling: IRT1, SIRT3, SIRT5, SIRT6, and SIRT7 control fibrosis and cardiomyopathy through FOS/AP‐1, p53, TGF‐β1/Smad3, Mef2, MPC1, IGF–Akt, GATA4, and NFATc2. They also modulate myocardial infarction, ischemia/reperfusion injury, drug‐induced damage, heart failure, and congenital defects via the ROS, NLRP3, CyPD, p53‐dependent ferroptosis, ISL1, OGG1, and GSTP1/JNK/autophagy pathways. (J and K) Vascular homeostasis and disease: sirtuin‐dependent regulation involves eNOS/NOS, oxidative stress, inflammation, mitophagy, antioxidant responses, and key mediators, including FOXM1, NRF2, SOD2, and GATA5. AC, acetylation; AP‐1, activator protein‐1; CyPD, cyclophilin D; ER stress, endoplasmic reticulum stress; FOS, proto‐oncogene FOS; FGF21, fibroblast growth factor 21; FoxO, forkhead box protein O; GSTP, glutathione S‐transferase P1; GATA4, GATA binding protein 4; IGF–Akt, insulin‐like growth factor–Akt; IL‐1β, interleukin‐1 beta; JNK, c‐Jun N‐terminal kinase; KO, knockout; LXR, liver X receptor; Mef2, myocyte enhancer factor 2; MCP‐1, monocyte chemoattractant protein 1; NFATc2, nuclear factor of activated T cells, cytoplasmic 2; NLRP3, nucleotide‐binding oligomerization domain‐like receptor with a pyrin domain 3; NOS, nitric oxide synthase; NRF2, nuclear factor erythroid 2‐related factor 2; OE, overexpression; OGG1, 8‐oxoguanine DNA glycosylase 1; Parkin, parkin RBR E3 ubiquitin protein ligase; Pink, PTEN‐induced kinase; ROS, reactive oxygen species; RIPK1, receptor‐interacting protein kinase 1; Smad, mothers against decapentaplegic homolog 3; SIRT, sirtuin; SERCA2a, sarco‐endoplasmic reticulum Ca2+‐ATPase; SOD, superoxide dismutase; TGF‐β, transforming growth factor‐β.

The cardiovascular system is uniquely constrained by continuous mechanical work, the tight coupling of ATP production to contractile demand, and lifelong endothelial exposure to stress. These features make the heart and vasculature particularly sensitive to changes in NAD^+^ availability. In this context, *Sirt1–7* mouse models allow us to dissect how individual SIRTs integrate metabolic and stress signals into specific phenotypes. We summarize the key phenotypes and mechanisms in Figure 2 and Table S3 and discuss them in detail below.

#### 3.1.1 Arrhythmia

SIRT1 is the best‐studied SIRT in cardiac rhythm control. Cardiomyocyte‐specific (α‐MHC–*Cre*)*Sirt1* KO in C57BL/6J mice is characterized by left ventricular dysfunction, frequent ventricular arrhythmias, disrupted Ca^2^^+^ and Na^+^ handling, and increased ROS levels [85]. Furthermore, loss of *Sirt1* in adult cardiomyocytes leads to hypertrophy, contractile dysfunction, and abnormal electrophysiology, which are associated with increased lysine crotonylation of sarcoplasmic/ER Ca^2^^+^‐ATPase 2a (SERCA2a) and altered expression of PPAR‐pathway proteins [170]. SIRT1 also plays a role in atrial electrophysiology. In C57BL/6 mice, myocyte enhancer factor 2c (Mef2c)–Cre‐driven *Sirt1* KO in the atria and right ventricle promotes atrial enlargement and increased susceptibility to age‐related atrial fibrillation via enhanced receptor‐interacting serine/threonine‐protein kinase 1 (RIPK1) acetylation and necroptosis [171]. Together, these models indicate that SIRT1 preserves the cardiac rhythm in a chamber‐specific manner by coordinating ion channel function, sarcoplasmic reticulum function, and necroptotic signaling.

#### 3.1.2 Myocardial Metabolism and Contractile Function

SIRT1, SIRT3, and SIRT5 protect myocardial metabolism and contractile function via distinct mechanisms. Cardiomyocyte‐specific *Sirt1* KO (C57BL/6J) impairs mitochondrial biogenesis, increases ROS levels, and leads to progressive systolic dysfunction, with greater sensitivity to transverse aortic constriction [172]. *Sirt1*‐deficient aging hearts exhibit severe contractile failure and apoptosis caused by oxidative and ER stress. Inducible global deletion of *Sirt1* further aggravates tunicamycin‐ and isoproterenol‐induced ER stress‐induced cardiomyopathy by impairing eEF2K/eEF2‐dependent autophagy [86, 174]. In FVB mice with cardiomyocyte‐specific *Sirt1* OE, a 2.5–7.5‐fold increase in SIRT1 delays age‐related hypertrophy, fibrosis, and pump failure and increases paraquat resistance through FOXO‐dependent induction of antioxidant enzymes [173]. In contrast, excessive *Sirt1* OE (∼12.5‐fold) causes cardiomyopathy with increased baseline apoptosis and oxidative stress [173]. Thus, modest SIRT1 activation supports mitochondrial function and stress resistance, whereas excessive activation is deleterious.

SIRT3 directly regulates mitochondrial proteins. Global *Sirt3* KO on a C57BL/6;129 background results in age‐dependent cardiac hypertrophy and fibrosis with impaired optic atrophy 1 deacetylation and cristae structure [175]. Under pressure overload, *Sirt3* deficiency (129/SvImJ) exacerbates contractile dysfunction and fibrosis through defective substrate oxidation and ATP depletion [176].

In C57BL/6J global *Sirt5* KO mice, fasting reduces cardiac ATP levels, increases the AMP/ATP ratio, and increases AMPK phosphorylation, accompanied by decreased ATP synthase activity and increased succinylation of its subunits [177]. Under transverse aortic constriction, this energetic reprogramming attenuates hypertrophy and contractile dysfunction [177], suggesting that SIRT‐dependent desuccinylation of ATP synthase tunes the myocardial ATP supply and AMPK activation during pressure overload.

Taken together, these findings suggest that cardiomyocyte SIRT1 plays protective roles by modulating ROS production, oxidative and ER stress, apoptosis, and autophagy, especially in aged mice, whereas excessive SIRT1 is detrimental. Consistent with these findings, studies in different mouse strains have demonstrated that SIRT3 protects against age‐related cardiac dysfunction by regulating mitochondrial proteins. In contrast, SIRT5 further fine‐tunes myocardial energetics: its loss increases ATP synthase succinylation, decreases ATP production, and activates AMPK, which can attenuate pressure overload‐induced hypertrophy and dysfunction.

#### 3.1.3 Cardiac Remodeling

Cardiac remodeling encompasses fibrosis, hypertrophy, and postischemic structural changes. Transgenic mouse models indicate that SIRT1, SIRT2, SIRT3, SIRT5, SIRT6, and SIRT7 act at different levels of these processes.

##### 3.1.3.1 Cardiac Fibrosis

The antifibrotic profile of SIRT3 is the clearest. Global *Sirt3* KO mice (B6;129S5) spontaneously develop cardiac fibrosis and inflammation during aging [128]. This phenotype stems from elevated FOS/AP‐1 transcriptional activity due to loss of SIRT3‐mediated H3 deacetylation at the FOS promoter [128]. In pressure‐overload models, global *Sirt3* deficiency aggravates hypertrophy and fibrosis after transverse aortic constriction, indicating that SIRT3 also limits fibrotic remodeling under conditions of hemodynamic stress, partially via inhibition of transforming growth factor β (TGF‐β)/Smad3 activity [178].

Cardiomyocyte‐specific *Sirt3* KO further revealed that SIRT3 regulates ferroptosis. Loss of *Sirt3* increases p53 acetylation, enhances ferroptosis, and promotes the release of damage‐associated molecular patterns, thereby amplifying fibrosis via paracrine mechanisms [94]. In these models, SIRT3 therefore restrains cardiac fibrosis through three mechanisms: by suppressing FOS/AP‐1‐driven transcription, by inhibiting TGF‐β/Smad3 signaling, and by limiting ferroptosis‐associated inflammation.

##### 3.1.3.2 Cardiomyopathy

Multiple SIRTs restrain cardiomyopathy. Global *Sirt1*ΔE4 mice (mixed backgrounds) develop dilated cardiomyopathy with ventricular dilation and smaller cardiomyocyte size and lack classic concentric hypertrophy or fibrosis. This phenotype stems from disrupted mitochondrial integrity and abnormal cardiac development induced by impaired MEF2 deacetylation [179].

Global *Sirt2* KO C57BL/6 mice exhibit spontaneous age‐dependent cardiac hypertrophy, fibrosis, and heart failure (HF) and display exaggerated responses to isoproterenol. Mechanistically, SIRT2 normally deacetylates and inhibits nuclear factor of activated T cells 2 (NFATc2); its absence stabilizes nuclear NFATc2 and promotes pathological remodeling [180].

Cardiomyocyte‐specific *Sirt3* KO exacerbates salt‐induced cardiac hypertrophy and dysfunction. In this model, SIRT3 functions by deacetylating mitochondrial pyruvate carrier 1, sustaining glucose oxidation and preventing maladaptive metabolic reprogramming [181].

SIRT6 and SIRT7 constrain hypertrophy through nuclear mechanisms. In mixed 129 sv/C57BL/6 mice, cardiomyocyte‐specific *Sirt6* KO mice develop cardiac hypertrophy and HF in response to transverse aortic constriction, isoproterenol, or Angiotensin II. Conversely, *Sirt6* OE prevents these phenotypes. SIRT6 suppresses IGF–Akt signaling by deacetylating H3K9 and suppressing c‐Jun, thereby preventing chronic pathological growth [182]. On a C57BL/6J background, cardiomyocyte‐specific *Sirt7* KO aggravates TAC‐ and Angiotensin II‐induced hypertrophy and fibrosis [183]. These mice exhibit increased heart weight and cardiomyocyte enlargement. Mechanistically, SIRT7 binds to GATA binding protein 4 and promotes its deacetylation, suppressing its prohypertrophic activity [183]. In mixed C57BL/6×129 mice, global *Sirt7* deficiency results in inflammatory cardiomyopathy with a shortened lifespan, progressive hypertrophy, fibrosis, and increased apoptosis [63]. Mechanistically, this reflects enhanced p53 acetylation/activation and overactive Akt–Ras–Raf–MEK–extracellular signal‐regulated kinase (ERK) signaling [63].

These data indicate that SIRT1, SIRT2, SIRT3, SIRT6, and SIRT7 collectively restrain pathological hypertrophy. They operate through different mechanisms that target the MEF2, NFATc2, IGF–Akt, and GATA binding protein 4/p53 pathways, ultimately preventing HF progression.

##### 3.1.3.3 Myocardial Infarction, I/R Injury, and Thrombosis

Acute myocardial infarction (AMI) and I/R injury (IRI) involve SIRT‐regulated axes of thrombosis, microvascular function, oxidative stress, and cell death.

Systemic SIRT3 limits arterial thrombosis. In C57BL/6J mice, global *Sirt3* KO accelerates carotid thrombus formation, increases neutrophil extracellular trap formation, and elevates plasma tissue factor activity. These effects suggest that SIRT3 limits thrombosis by attenuating oxidative stress and neutrophil extracellular trap‐driven coagulation [97].

Within the infarcted heart, SIRT3 also preserves coronary microvascular function. In 129S1/SvImJ mice subjected to left‐anterior‐descending ligation, global *Sirt3* deficiency impaired capillary density, coronary angiogenesis, and cardiac recovery. This defect is accompanied by reduced expression of angiogenic factors and altered endothelial metabolism [186].

SIRT5 exerts cardioprotective effects via liver‒heart cross talk. In C57BL/6J mice, albumin–Cre‐driven hepatocyte‐specific *Sirt5* OE reduces MI and fibrosis size while improving function after left coronary artery ligation. This protection coincides with elevated levels of circulating and myocardial fibroblast growth factor 21, indicating that hepatic SIRT5 increases fibroblast growth factor 21 secretion to support systemic metabolism in the injured heart [188].

In cardiomyocytes, SIRT1 mitigates IRI. Cardiomyocyte‐specific *Sirt1* KO in C57BL/6J mice worsens I/R outcomes, with larger infarcts, poorer functional recovery, increased ROS, and enhanced NLRP3 inflammasome activation. Mechanistic studies have shown that SIRT1 deacetylates liver kinase B1 to activate AMPK and alter metabolism during ischemia [184] and regulates pyruvate dehydrogenase to suppress NLRP3‐mediated pyroptosis [113]. SIRT1 is also required for the cardioprotective effect of caloric restriction, acting via the downregulation of complement C3 and the suppression of local complement activation [185].

In 129S6/SvEvTac mice, global *Sirt3* KO increases susceptibility to IRI, resulting in larger infarcts and loss of protection from ischemic postconditioning. SIRT3 deacetylates cyclophilin D, inhibiting the mitochondrial permeability transition pore and limiting cell death [187]. SIRT7 further affects structural outcomes after ischemia. In C57BL/6 mice, global *Sirt7* KO resulted in impaired fibrosis, delayed blood flow recovery, and an increased risk of cardiac rupture after MI and hind‐limb ischemia. SIRT7 stabilizes TβRI through autophagy‐dependent mechanisms and interactions with its binding partner PICK1, thereby sustaining the TGF‐β signaling required for tissue repair [157].

Collectively, during MI and IRI, SIRT1 and SIRT3 primarily safeguard cardiomyocyte survival and mitochondrial function, whereas SIRT7 modulates vascular and reparative remodeling, and hepatic SIRT5 contributes to these processes via endocrine support of the injured heart. Future work should define how these SIRT pathways interact across organs, cell types, and times after infarction and assess whether selective, context‐dependent SIRT modulation can be harnessed to improve clinical outcomes in AMI and reperfusion therapies.

##### 3.1.3.4 Drug‐Induced Cardiomyopathy

Drug‐induced cardiomyopathy, especially from anthracyclines and kinase inhibitors, is characterized by sharp increases in oxidative stress, mtDNA damage, and regulated cell death.

In a DOX model, cardiomyocyte‐specific *Sirt1* KO (C57BL/6J) exacerbated cardiac dysfunction, hypertrophy, and oxidative stress. Conversely, resveratrol or *Sirt1* OE upregulates Sestrin2 and mitigates injury, establishing a SIRT1–Sestrin2–AMPK axis that suppresses ROS and apoptosis [84].

Global *Sirt3* KO mice exhibit exacerbated DOX‐induced cardiomyopathy, resulting in severe hypertrophy and adverse remodeling. Mechanistically, SIRT3 preserves mtDNA integrity by maintaining 8‐oxoguanine DNA glycosylase 1 (OGG1) activity and reducing oxidative stress [74]. Conversely, *Sirt3* OE protects C57BL/6 hearts from DOX‐induced injury, which is correlated with reduced mitochondrial protein acetylation and decreased ROS levels [96]. In contrast, SIRT3 can be detrimental in specific pharmacologic contexts. In sunitinib‐induced cardiotoxicity, global *Sirt3* KO mice are paradoxically protected. SIRT3 binds and inhibits glutathione S‐transferase P1 in cardiac pericytes, disrupting the glutathione S‐transferase P1/c‐Jun N‐terminal kinase/autophagy protective axis. This sensitizes the heart to sunitinib, whereas SIRT3 deficiency preserves this pathway [148].

SIRT6 also contributes to DOX tolerance. Heterozygous *Sirt6* KO mice on a C57BL/6J background display worsened cardiac dysfunction, apoptosis, and oxidative stress. SIRT6 protects against this injury by suppressing p53/Fas‐dependent cell death and bolstering endogenous antioxidant defenses [107].

In drug‐induced cardiomyopathy models, SIRT1 and SIRT6 are largely protective, limiting oxidative stress and cell death, whereas SIRT3 can either preserve mitochondrial integrity (DOX) or aggravate injury (sunitinib) depending on the cell type and pathway involved. This divergence highlights that “SIRT activation” is not uniformly beneficial and that SIRT‐targeted therapies must account for the isoform, drug context, and cellular compartment to avoid unintended harm.

##### 3.1.3.5 Other Heart Diseases

In addition to modulating classical remodeling, SIRTs also modulate HF progression and congenital heart disease.

Cardiomyocyte‐specific *Sirt1* KO mice exhibit impaired Ca^2^^+^ handling and reduced contractility, leading to adverse left ventricular remodeling and a diminished ejection fraction. Mechanistically, SIRT1 reduces sarcoplasmic/ER SERCA2a acetylation to restore its activity in HFs, establishing SIRT1 as a regulator of Ca^2^^+^ ATPase during decompensation [189]. In congenital heart disease, SIRT1 functions at the progenitor level. In C57BL/6 N mice, deletion of *Sirt1* in Isl1^+^ cardiac progenitor cells (Elf5–Cre) prevents CoCl_2_‐induced hypoxic defects by maintaining Isl1 expression and progenitor proliferation. Under hypoxia, the HIF‐1α/HES1/SIRT1 complex represses Isl1; SIRT1 loss disrupts this complex, preserving progenitor pools and preventing structural defects [87].

Multiple *Sirt3* models support a central role in HF. Mice with global *Sirt3* deletion and mitochondrial lysine acetyltransferase MOF OE develop HF via disrupted energy metabolism. Mechanistically, this reflects hyperacetylation of ATP5B, a critical ATP synthase subunit, which is normally counteracts by SIRT3 [191]. Global and cardiomyocyte‐specific *Sirt3* KO models on a C57BL/6 background show severe cardiac dysfunction, hypertrophy, and HF following ISO or TAC. Notably, NAD phosphate (NADPH) treatment fails to rescue these phenotypes, demonstrating that SIRT3‐mediated deacetylation of mitochondrial targets is essential for preventing pathological hypertrophy and failure [192]. Mechanistic work has indicated that SIRT3 functions by regulating mitochondrial iron homeostasis and ferroptosis; its loss increases p53 acetylation and impairs iron‒sulfur cluster biogenesis, triggering mitochondrial ferroptosis and contractile decline [190].

In these models, SIRT1 promotes SERCA2a function in HF, but paradoxically, its loss can preserve cardiac progenitor pools under hypoxia. In contrast, SIRT3 consistently safeguards adult cardiac function by maintaining mitochondrial protein deacetylation, ATP synthase activity, and iron‒sulfur cluster biogenesis. Together, these data suggest that SIRT targeting in HF and congenital heart disease requires careful distinction between developmental and adult roles.

#### 3.1.4 Vascular Homeostasis and Disease

Vascular aging, stiffness, atherogenesis, hypertension, and aneurysms form a pathological continuum rooted in chronic inflammation, oxidative damage, and maladaptive remodeling. SIRT1, SIRT2, SIRT3, and SIRT6 operate at critical nodes within this spectrum across endothelial cells (ECs), VSMCs, and immune compartments, as shown by modified mouse models.

##### 3.1.4.1 Arterial Stiffness and Atherosclerosis

During age‐related arterial stiffening, SIRT1 functions primarily in VSMCs. Lifelong global *Sirt1* OE in C57BL/6 mice attenuates age‐dependent aortic stiffening and structural deterioration. This protection involves preserved elastin content and reduced collagen deposition, advanced glycation end product accumulation, and calcification [193]. Conversely, SM22α–Cre‐driven *Sirt1* KO in VSMCs fed high‐fat/high‐sucrose diets increased aortic stiffness and remodeling. Notably, VSMC‐specific *Sirt1* OE reduces stiffness and injury‐induced neointima through a CKII–SIRT1–SM22α loop that maintains the contractile phenotype and suppresses inflammation [114, 116].

In atherosclerosis, SIRT1 controls systemic lipid handling and local vascular responses. Global *Sirt1* deficiency (complete or haploinsufficient) in apoE‐null backgrounds (129/Sv and C57BL/6) reduces high‐density lipoprotein levels and impairs cholesterol efflux. These mice develop larger, more inflammatory plaques, reflecting defective SIRT1‐mediated liver X receptor activation in hepatocytes and macrophages [194, 208]. In the endothelium, *Sirt1* deletion (Tie2–Cre) also accelerates atherosclerosis and senescence [195]. Conversely, endothelial *Sirt1* OE (Tie2–Cre or VE–cadherin–Cre) limits lesion size and improves function [196, 197]. Mechanistically, SIRT1 promotes an anti‐inflammatory and antioxidative phenotype in ECs through the deacetylation of targets such as eNOS and FoxO1, thereby reducing atherosclerosis [195].

SIRT2 and SIRT3 contribute to vascular aging and atherogenesis via redox control. Global *Sirt2* KO in C57BL/6 mice exacerbates aggravated aging‐induced vascular remodeling, arterial stiffness, and vasomotor dysfunction. These defects stem from p66Shc activation and mROS overproduction, establishing SIRT2 as a cytoplasmic suppressor of mROS‐driven vascular aging [198]. Endothelial‐specific *Sirt3* KO (VE–cadherin–Cre) in an apoE‐deficient background increases vascular oxidative stress, inflammation, and endothelial dysfunction, exacerbating atherosclerosis; moreover, NAD^+^ supplementation partially reverses these defects in a SIRT3‐dependent manner [95].

SIRT6 as a protector of ECs. In brain microvascular ECs, SIRT6 deficiency increases the expression of scavenger receptor 1, exacerbating vascular inflammation and the plaque burden. This loss also drives EC growth arrest and premature senescence through forehead box M1 dysregulation [199, 200].

Across vascular beds, VSMC–SIRT1 preserves an elastic, contractile phenotype and limits age‐related stiffening and neointimal growth. Endothelial SIRT1 maintains endothelial nitric oxide synthase/FOXO1 signaling and restrains plaque formation. SIRT2 and SIRT3 act primarily through mitochondrial redox control to curb mROS‐driven vascular aging and atherogenesis. SIRT6 suppresses scavenger receptor expression, inflammation, and premature senescence.

##### 3.1.4.2 Hypertension and Aortic Aneurysm

In hypertension and aneurysm models, vascular SIRTs integrate redox control, matrix remodeling, and mitochondrial function.

VSMC‐specific *Sirt1* KO causes Ang II‐induced aortic dissection and rupture. Conversely, *Sirt1* OE in VSMCs decreases systolic blood pressure and attenuates Ang II‐induced remodeling [203, 204]. Mechanistically, SIRT1 suppresses oxidant‐induced matrix metalloproteinase (MMP) activity to preserve elastin integrity and suppress TGF‐β1 expression and downstream signaling.

Global *Sirt3* KO in C57BL/6J mice exacerbates hypertension, vascular inflammation, and premature vascular aging following Ang II or deoxycorticosterone acetate‐salt challenge. These defects include SOD2 hyperacetylation, mitochondrial superoxide accumulation, NO depletion, and telomerase downregulation. Notably, *Sirt3* OE reverses these pathological changes [37, 98]. Furthermore, SIRT3 promotes angiogenesis and lymphangiogenesis through Pink1/Parkin‐mediated mitophagy and VEGF‐C/VEGFR3–ERK signaling. This activity attenuates Ang II‐induced cardiac fibrosis and microvascular rarefaction [205, 206].

In Ang II models, global *Sirt6* OE suppresses abdominal aortic aneurysms, preserving elastic fibers and VSMC content and reducing p16/p21 levels [207, 210, 211]. VSMC‐specific *Sirt6* KO markedly accelerated thoracic aortic aneurysm and dissection and resulted in increased inflammation, senescence, mitochondrial dysfunction, and elastin rupture. Mechanistically, SIRT6 restricts thoracic aortic aneurysm by repressing interleukin (IL)‐1β transcription through H3K9/H3K56 deacetylation and preventing interferon regulatory factor 8 recruitment and by maintaining NRF2‐dependent mitochondrial biogenesis and oxidative phosphorylation in VSMCs [207, 210, 211]. Endothelial‐specific *Sirt6* KO (Tie2) further exacerbates deoxycorticosterone acetate‐salt/Ang II‐induced hypertension, endothelial dysfunction, and cardiorenal injury via disruption of Nkx3.2–GATA binding protein 5 (GATA5) signaling and antioxidant defenses [209].

These findings suggest that SIRT1 and SIRT3 are central regulators of hypertensive vascular remodeling and that SIRT6 is a critical epigenetic safeguard against aneurysm‐prone inflammation, senescence and mitochondrial failure. They also point to the need for vascular‐bed‐ and isoform‐specific SIRT targeting in hypertensive disease.

##### 3.1.4.3 Metabolic and Ischemic Vascular Diseases

Endothelial dysfunction is a hallmark of aging and metabolic vascular disease. SIRT1 plays a pivotal role in developmental and ischemia‐induced angiogenesis, as well as in hyperglycemia‐induced endothelial injury.

Tie2–Cre‐driven endothelial *Sirt1* deletion in 129/Sv and C57BL/6 mice impaired postnatal neovascularization and hindered ischemia‐induced angiogenesis in a hindlimb ischemia model. This is attributable to the loss of FOXO1 deacetylation, excessive FOXO1 activity, and reduced EC sprouting and branching [212]. In streptozotocin‐induced diabetes, endothelial *Sirt1* OE decreases p66Shc expression, improves endothelial function, and attenuates oxidative stress. Conversely, endogenous SIRT1 protects against hyperglycemia‐induced dysfunction by epigenetically silencing p66Shc through chromatin modification [213].

Collectively, the results of cardiovascular *Sirt* models reveal that SIRT1, SIRT2, SIRT3, and SIRT6 constitute a regulatory network within the vessel wall. These enzymes integrate metabolic, redox, and epigenetic signals to sustain arterial compliance, suppress plaque formation, restrain hypertension and aneurysm, and preserve angiogenic capacity.

#### 3.1.5 Research Limitations and Future Perspectives

Taken together, these findings reveal a multidimensional regulatory network in cardiovascular Sirt1–7 transgenic mice. SIRT1, SIRT3, and SIRT6 function as central regulators. SIRT1 and SIRT3 safeguard cardiomyocyte metabolism, redox balance, and survival. SIRT6 affects pathological hypertrophy, vascular inflammation, and aneurysm formation. SIRT2, SIRT5, and SIRT7 provide context‑dependent modulation of remodeling, thrombosis, and inflammatory injury. These findings are consistent across many studies, but the experimental landscape is unbalanced. Most work relies on global KO lines or a single type of cardiomyocyte‑ or endothelial‑specific deletion. There are relatively few OE lines. Catalytic–dead and point–mutant alleles are virtually absent. Inducible systems are rarely used to separate development from adult roles. Sex, age, and strain effects have also been incompletely explored. SIRT4 and SIRT5 remain underrepresented in cardiovascular models despite their clear roles in mitochondrial metabolism. Future studies will need multiallelic designs, including those involving KOs, OEs, and point mutants. They should broaden cell‑type coverage to fibroblasts, immune cells, and conduction‑system cells. Integration with single‑cell and spatial omics will be essential to define when and where individual SIRTs are truly rate‑limiting in human‑relevant cardiac and vascular disease.

### 3.2 SIRT family Transgenic Mouse Models of Respiratory Diseases

Unlike other organ systems, the respiratory tract is uniquely exposed to inhaled toxins, pathogens, and mechanical stretch and is organized around a delicate air‒blood barrier where epithelial, endothelial, and immune cells continuously interact. As outlined in Section 2, SIRTs regulate mitochondrial function, redox balance, DNA repair, inflammation, and autophagy. In the lung, dysregulation of these pathways manifests as chronic fibrosis, obstructive airway disease, asthma, susceptibility to infection, and acute lung injury (ALI). Transgenic mouse models have been particularly useful for determining how SIRTs operate at three key levels: the alveolar epithelium and fibroblasts, airway epithelium and immune cells, and the alveolar‒capillary barrier under acute stress. Table S4 provides an overview of these phenotypes and mechanisms, which are discussed in detail below.

#### 3.2.1 Pulmonary Fibrosis

Pulmonary fibrosis (PF) is a chronic progressive interstitial lung disease in which oxidative stress, mitochondrial dysfunction, and impaired cell death control converge to destroy alveolar architecture. *Sirt1* and *Sirt3* transgenic mouse models indicate that these two SIRTs act at distinct, compartment‐specific nodes in this process.

At the fibroblast level, global *Sirt1^Y/Y^* mice on the C57BL/6 background exhibit attenuated bleomycin‐induced PF. *Sirt1*‐deficient mice exhibit reduced fas‐associated death‐domain‐like IL‐1beta‐converting enzyme‐like inhibitory protein levels, increased Ku70 acetylation, and enhanced fibroblast apoptosis. These changes collectively restrict fibrosis accumulation [214]. These data support a model in which SIRT1 promotes fibrosis by deacetylating Ku70, stabilizing fas‐associated death‐domain‐like IL‐1beta‐converting enzyme‐like inhibitory protein, and conferring apoptosis resistance to lung fibroblasts.

*Sirt3* genetic models highlight mtDNA integrity as a key upstream determinant at the alveolar epithelial level. In 129/SJ mice, global *Sirt3* KO exacerbates asbestos‐ and bleomycin‐induced PF, with increased interstitial collagen deposition, mtDNA damage, and apoptosis in alveolar epithelial cells [73]. Mechanistically, SIRT3 deacetylates mitochondrial targets such as manganese superoxide dismutase and OGG1, thereby limiting oxidative mtDNA damage and epithelial cell loss. Gain‐of‐function studies in C57BL/6J mice with ∼4‐fold global *Sirt3* OE support these findings. Following asbestos exposure, these animals exhibit attenuated fibrosis, preserved lung mtDNA integrity, and decreased recruitment of profibrotic monocyte‐derived alveolar macrophages [72]. Together, these models indicate that SIRT3 protects against PF by preserving mitochondrial genome integrity in the alveolar epithelium and preventing the secondary influx of fibrogenic macrophages.

Overall, the data from PF models demonstrate a profibrotic role for SIRT1 in lung fibroblasts and an antifibrotic role for SIRT3 in alveolar epithelial cells, highlighting the cell type‐ and pathway‐specific nature of SIRT function within the injured lung. However, these conclusions are currently based on global models. They therefore cannot fully distinguish cell‐autonomous effects from systemic effects and require confirmation in cell‐specific and inducible systems.

#### 3.2.2 Chronic Obstructive Pulmonary Disease

Cigarette smoke (CS) drives chronic obstructive pulmonary disease (COPD) through converging pathways of oxidative stress, autophagic dysregulation, and cellular senescence that destroy airspace integrity. Studies in *Sirt1‐genetic* mouse models, spanning pure 129/SvJ and mixed C57BL/6J backgrounds, revealed that SIRT1 confers broad protection against CS‐induced injury, although the magnitude of this effect varies with both expression level and cellular context.

In 129/SvJ global *Sirt1*‐null heterozygous mice, CS exposure markedly increased autophagy in the lung. Pharmacological inhibition of poly(ADP‐ribose)‐polymerase‐1 (PARP‐1) activity attenuates CS‐induced autophagy by increasing SIRT1 activity, indicating that the PARP‐1–SIRT1 axis is involved in controlling autophagic responses to smoke injury [215]. In a complementary model using airway epithelium‐ and myeloid cell‐specific *Sirt1* deletions in C57BL/6J;129/SvJ backgrounds, *Sirt1* loss in the airway epithelium, but not in myeloid cells, led to spontaneous airspace enlargement and heightened susceptibility to emphysema after CS exposure [216]. Mechanistically, epithelial SIRT1 limits emphysema by activating FOXO3 and reducing cellular senescence, largely independent of inflammatory changes. Collectively, these findings position SIRT1 as a guardian against COPD‐like injury: global insufficiency unleashes PARP‐1‐driven autophagy, epithelial deletion accelerates FOXO3‐mediated senescence, and myeloid deficiency remains phenotypically silent.

#### 3.2.3 Asthma

Asthma is characterized by reversible airflow limitation and Th2‐biased immune responses [313]. *Sirt1* and *Sirt2* transgenetic mouse models indicate that these deacetylases shape asthma pathogenesis through distinct immune compartments.

Integrin alpha X (CD11c)–Cre‐mediated *Sirt1* deletion in DCs attenuated ovalbumin‐induced airway inflammation and Th2 responses. In these DCs, PPAR‐γ activity increases, whereas Th2 priming and Type‐2 cytokine output decrease. These findings imply that SIRT1 ordinarily drives allergic sensitization by restraining the ability of PPAR‐γ to regulate Th2 immunity [120].

LysM–Cre‐mediated *Sirt1* deletion in myeloid cells exacerbates house dust mite‐induced airway inflammation and cytokine elevation, inversely mirroring the DC phenotype. This finding reflects unchecked ERK/p38 MAPK signaling in macrophages, which is normally suppressed by SIRT1 to restrain allergic responses. Consequently, SIRT1 functions as a proallergic factor in DCs but as a suppressor in macrophages, with the prevailing disease phenotype dictated by which compartment is predominant [121].

Studies in global *Sirt2* KO C57BL/6 mice exposed to dust mites, ragweed or Aspergillus fumigatus have demonstrated that SIRT2 exacerbates allergic pathology. Consistent with these findings, *Sirt2*‐deficient animals exhibit attenuated airway inflammation and goblet‐cell hyperplasia, phenotypes that correlate with altered macrophage activation and decreased C‐C motif chemokine ligand 17 production [124].

Collectively, these findings establish SIRT1 as a context‐dependent immune regulator and SIRT2 as a general promoter of Type 2 inflammation. While SIRT1 licenses Th2 priming in DCs, it simultaneously suppresses MAPK signaling in macrophages; in contrast, SIRT2 uniformly exacerbates allergic responses across compartments.

#### 3.2.4 Pulmonary Infection

Respiratory syncytial virus (RSV) and mycobacterial infections reveal context‐specific roles for SIRT1 and SIRT3 in pulmonary innate immunity, with effects on mitochondrial bioenergetics, autophagic flux, and inflammatory signal regulation.

During viral infection, CD11c–Cre‐driven *Sirt1* deletion exacerbates RSV‐induced lung pathology in C57BL/6J mice. The loss of *Sirt1* in DCs compromises mitochondrial fitness: the membrane potential decreases, oxygen consumption decreases, and ROS accumulate. Concurrently, these cells shift toward fatty acid synthesis and exhibit dysregulated cytokine profiles [122]. A complementary study using the same DC‐specific KO model reported increased viral load, increased Th2 cytokines, and impaired autophagosome formation and DC activation during RSV infection [123]. Together, these data indicate that SIRT1 in DCs supports effective antiviral immunity by coordinating mitochondrial function and autophagy, ultimately limiting the RSV burden and lung damage.

In mycobacterial infection, myeloid cell‐specific *Sirt1*KO mice on a C57BL/6 background show increased susceptibility to acute and chronic *M. tuberculosis* infection, with increased bacterial loads and exaggerated inflammatory responses [217]. Biochemically, SIRT1 promotes autophagy and phagosome‒lysosome fusion in macrophages and deacetylates p65 to normalize *M. tuberculosis*‐induced inflammation, thereby restricting bacterial growth.

SIRT3 functions as a complementary mitochondrial checkpoint in myeloid cells. With respect to *Mycobacterium tuberculosis* infection, global *Sirt3* KO mice exhibit increased mortality and pulmonary bacterial burden, which are rooted in mitochondrial dysfunction, oxidative overload, and macrophage death [100]. In parallel, *Sirt3* deficiency during *M. abscessus* challenge drives elevated bacterial loads and exaggerated lung inflammation, reflecting an inability to preserve mitochondrial homeostasis and redox balance [101]. Collectively, these models identify SIRT1 and SIRT3 as key host‐directed regulators of antiviral and antibacterial defense in the lung, acting primarily in DCs and macrophages to integrate mitochondrial function, autophagy, and inflammatory signaling.

#### 3.2.5 Acute Lung Injury

ALI is a high‐mortality inflammatory syndrome whose severity increases markedly with age. In this context, SIRT1 and SIRT3 occupy distinct cellular niches: while SIRT1 operates primarily in myeloid cells, SIRT3 safeguards mitochondrial function within the alveolar epithelium [314].

In sepsis‐induced ALI, compared with wild‐type (WT) controls, global *Sirt1* KO C57BL/6 mice subjected to cecal ligation and puncture develop more severe lung tissue damage, oxidative stress, and ferroptosis [88]. At the molecular level, SIRT1 in macrophages affects the NRF2/HO‐1 axis to inhibit ROS accumulation and ferroptosis; loss of this signaling exacerbates sepsis‐associated ALI. In toxic models such as those involving paraquat poisoning, pharmacologic SIRT1 activation also reduces lung injury through the activity of NRF2‐dependent antioxidant pathways [315, 316].

SIRT3 has been investigated in several ALI settings. In 129S1/SvImJ mice, global *Sirt3* KO aggravates endotoxin‐induced ALI, with increased inflammation and mitochondrial dysfunction. Conversely, intact SIRT3 signaling protects against lipopolysaccharide (LPS) challenge by attenuating mROS and restraining inflammasome activation [131]. In transgenic mouse models, *Sirt3* deficiency similarly exacerbated sepsis‐induced ALI and lung‐cell injury, and SIRT3 was shown to exert its protective effect largely through inhibition of the NLRP3 inflammasome pathway [218]. In C57BL/6 mice exposed to CdCl_2_, *Sirt3* KO further promoted alveolar epithelial senescence, COPD‐like structural damage, and decreased lung function. In this context, SIRT3 maintains mitochondrial function and restrains epithelial aging by deacetylating targets such as SOD2 and isocitrate dehydrogenase 2 [219].

Together, these models identify SIRT1 as a regulator of macrophage‐mediated oxidative stress and ferroptosis in sepsis‐associated ALI and SIRT3 as a key mitochondrial defender that limits ROS, inflammasome activation, and epithelial senescence across diverse ALI stimuli.

#### 3.2.6 Research Limitations and Future Perspectives

In the context of respiratory diseases, SIRT1 and SIRT3 serve as protective factors in most respiratory diseases, although SIRT1 can have profibrotic effects on pulmonary fibroblasts. SIRT2 instead acts as a context‑dependent amplifier of allergic inflammation. SIRT1 regulates fibroblast survival in fibrosis, epithelial senescence in COPD, and myeloid responses in asthma, infection, and ALI. SIRT3 safeguards mtDNA and limits oxidative damage to the alveolar epithelium. Notably, the same SIRT can be pro‑ or anti‑inflammatory depending on the immune cell type. Together with the promotion of Type‑2 inflammation by SIRT2, these findings suggest that cell‑type‑specific rather than global SIRT modulation occurs in PF, COPD, asthma, and pulmonary infection. Current evidence is still limited. Most data come from acute injury models in a few mouse strains. SIRT4–7 is rarely studied in the lung, and mechanical factors such as ventilation‑induced stretching are largely ignored. Future work should combine cell‑type‑specific *Sirt1–7* manipulations with chronic exposure models to define SIRT targeting in pulmonary disease.

### 3.3 SIRT Family Transgenic Mouse Models of Digestive System Diseases

**FIGURE 3:**
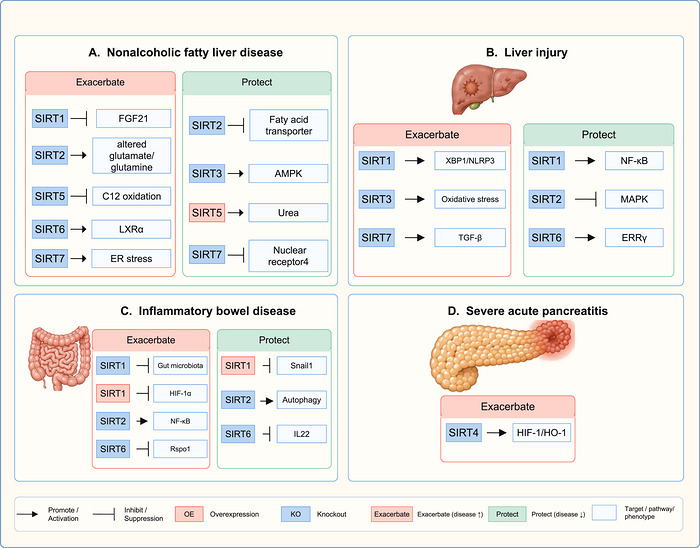
Sirtuins in digestive system diseases. This schematic summarizes the protective (green) and detrimental (red) roles of sirtuins in digestive system diseases on the basis of OE (pink) and KO (blue) mouse models. Arrows indicate activation/promotion; blunt‐ended lines indicate inhibition/suppression. (A) Nonalcoholic fatty liver disease: SIRT1/2/3/5/6/7 regulate hepatic lipid and amino acid metabolism via the FGF21, AMPK, ER stress, and nuclear receptor pathways. (B) Hepatic injury: Sirtuins modulate inflammation and stress responses, including those involving NLRP3, oxidative stress, TGF‐β, NF‐κB, and MAPK signaling. (C) Inflammatory bowel disease: SIRT1/2/6 affects the gut microbiota, HIF‐1α, NF‐κB, autophagy, and IL‐22‐mediated immunity. (D) Severe acute pancreatitis: SIRT4 is linked to HIF‐1/HO‐1‐mediated protection against pancreatic injury. AMPK, AMP‐activated protein kinase; ERRγ, estrogen‐related receptor gamma; HIF‐1α, hypoxia‐inducible factor‐1 alpha; HO‐1, heme oxygenase‐1; MAPK, mitogen‐activated protein kinase; NF‐κB, nuclear factor‐kappa B; XBP1,X‐box‐binding protein 1.

The digestive system is highly metabolic and is continuously exposed to dietary, microbial, and xenobiotic stress. As outlined in Section 2, SIRTs regulate hepatic and intestinal homeostasis through coordinated control of lipid and glucose metabolism, genome integrity, oxidative and ER stress, immunity, and autophagy. Transgenic mouse models have now begun to link these mechanistic axes to specific digestive diseases, particularly nonalcoholic fatty liver disease (NAFLD), liver injury, inflammatory bowel disease (IBD), and SAP. The main phenotypes and pathways are summarized in detail below in Figure 3 and Table S5.

#### 3.3.1 Nonalcoholic Fatty Liver Disease

NAFLD is characterized by hepatocellular triglyceride accumulation, lipotoxicity, oxidative and ER stress, mitochondrial dysfunction, and low‐grade inflammation [317]. In this context, the SIRT family members act at partly distinct, and sometimes opposing, nodes of hepatic lipid and stress regulation, as detailed below.

Consistent with its central metabolic role, hepatic SIRT1 promotes FAO and limits steatosis. In liver‐specific *Sirt1* KO mice (albumin–Cre), β‑oxidation programs are not induced in response to nutritional cues, and these mice display exacerbated steatosis and NAFLD progression, which is in line with the ability of this gene to deacetylate PGC‑1α and regulate fibroblast growth factor signaling and β‑oxidation genes [220].

In contrast, SIRT2 plays complex roles in hepatic lipid metabolism, senescence, and fibrosis. Under a normal diet, *Sirt2* KO mice have greater glycogen storage in the liver. However, compared with their WT littermates, HFD‐fed global *Sirt2* KO mice have less hepatic fat accumulation, along with attenuated weight gain and reduced visceral adipose tissue  [221]. Mechanistically, SIRT2 deficiency downregulates the expression of glucose and fatty acid transporters and aquaporin‐9 in the liver, thereby limiting substrate uptake for lipid synthesis. On a HFD, *Sirt2* KO livers also display hepatocellular senescence and a fibrotic phenotype independent of fat accumulation. Thus, although SIRT2 deficiency effectively alleviates hepatic steatosis, it directly promotes hepatocyte senescence and liver fibrosis—effects that are detrimental. Therefore, targeting SIRT2 for NAFLD therapy requires comprehensive consideration.

SIRT3 fine‑tunes the balance between autophagy‐dependent lipid handling and lipotoxic apoptosis in hepatocytes [318]. *Sirt3* KO enhances AMPK activation and autophagic flux, protecting cells against saturated fatty acid toxicity [153]. In contrast, liver‐specific *Sirt3* OE suppresses AMPK signaling and reduces autophagic flux, similarly rendering cells more vulnerable to lipid stress [153]. These opposite phenotypes indicate that aberrant SIRT3 overactivation under disease conditions is the underlying problem. Therefore, therapeutic strategies should aim to curb excessive SIRT3 activity rather than simply inhibiting or activating it.

Although less extensively studied in NAFLD, SIRT4 also influences hepatic lipid metabolism indirectly through amino acid metabolism and energy sensing. *Sirt4*‑deficient mice exhibit altered glutamate/glutamine and BCAA flux, increased amino‑acid‐stimulated insulin secretion, and activation of PPARα‑ and AMPK–PGC‑1α‑dependent fatty‑acid oxidation and mitochondrial biogenesis in the liver, particularly during fasting [10, 43, 45, 46, 223]. These changes may serve as short‐term compensatory responses to promote lipid combustion, but their persistence ultimately accelerates the progression toward insulin resistance and NAFLD [10, 43, 223].

SIRT5 links amino acid and nitrogen handling to NAFLD progression. In Sirt5 KO mice, impaired CPS1 activity causes hyperammonemia and defective adaptation to dietary nitrogen stress [53], whereas increased inactivation of GCDH glutarylation disrupts mitochondrial amino acid oxidation [52]. Conversely, hepatic Sirt5 OE enhances urea production [49] and, in obese ob/ob mice, improves glycolysis and FAO while reducing hepatic triglyceride levels without altering body weight [50]. Recent work in C57BL/6NJ *Sirt5* KO mice confirms that SIRT5 maintains mitochondrial amino acid oxidation by deglutarylating GCDH, as loss of SIRT5 increases GCDH glutarylation, reduces tetramer stability, impairs glutaryl‐CoA oxidation, and causes the accumulation of lysine/tryptophan‐derived amino acids. Together with its deacetylation of pyruvate dehydrogenase and succinate dehydrogenase, which tunes TCA cycle entry and respiration [224], these data indicate that SIRT5 shapes the balance between carbon and nitrogen disposal in the fatty liver. In a coconut‑oil model on a mixed C57BL/6J×129 background, global *Sirt5* deficiency caused periportal macrovesicular steatosis and increased hepatic triglycerides because of impaired mitochondrial activation and oxidation of C12 fatty acids despite compensatory peroxisomal and ω‑oxidation [225]. Conversely, hepatocyte‑specific *Sirt5* OE in obese ob/ob mice demalonylates and desuccinylates glycolytic, gluconeogenic, and oxidative‑phosphorylation enzymes; enhances glycolysis and fatty‑acid oxidation; suppresses gluconeogenesis; and reduces hepatic triglyceride content without altering body weight or glucose tolerance [51]. Notably, global *Sirt5* OE on a low‐fat diet causes widespread hypomethylation and hyposuccinylation in brown fat, liver, muscle, and heart but does not affect body weight, energy expenditure, or lipid indices [47]. These findings suggest that SIRT5 is functionally important in NAFLD under lipid‐ and nitrogen‐rich stress conditions but not under basal conditions.

SIRT6 acts as a chromatin‐based brake on lipogenesis while facilitating fatty acid disposal and appears to be protective in NAFLD models. Liver‐specific *Sirt6* KO mice are more susceptible to Western diet‐induced fatty liver disease, with elevated hepatic and serum diacylglycerol and triglyceride levels [60]. Mechanistically, SIRT6 suppresses hepatic lipogenesis by directly deacetylating and suppressing the transcriptional activity of liver X receptor α, sterol regulatory element‐binding protein 1, and carbohydrate response element‐binding protein [60]. Furthermore, SIRT6 directly binds to saturated fatty acids such as palmitate in the cytoplasm, inducing its translocation from the nucleus. In this cytoplasmic compartment, SIRT6 deacetylates acyl‐CoA synthetase long chain family member 5 to increase FAO [226]. Overall, hepatic SIRT6 appears to be protective in NAFLD models, and its loss promotes steatosis.

The role of SIRT7 in hepatic lipid accumulation remains incompletely understood. Under normal chow conditions, global *Sirt7* KO mice spontaneously develop hepatic steatosis, which is attributed to unrestrained ER stress‐driven lipogenesis; liver‐specific Sirt7 KO similarly causes fatty liver [61]. In striking contrast, Sirt7 KO mice fed a HFD are paradoxically protected against steatosis and obesity. Mechanistically, SIRT7 stabilizes the nuclear receptor TR4 by inhibiting its ubiquitination, thereby promoting fatty acid uptake and triglyceride synthesis [63]. Thus, SIRT7 may prevent steatosis under normal conditions but promote lipid accumulation under HFD conditions [63]. Alternatively, these opposing phenotypes may arise from differences in gene‐targeting strategies (e.g., the presence or absence of a LacZ fusion) and genetic backgrounds (C57BL/6 vs. 129 Sv). Collectively, these findings suggest that specific SIRT7 inhibitors could be beneficial for treating hepatic steatosis; however, caution is warranted given the context‐dependent nature of SIRT7 function.

In summary, SIRT1 and SIRT6 protect against steatosis. SIRT2 reduces fat but drives senescence and fibrosis. SIRT3 overactivation worsens lipotoxicity. SIRT4 and SIRT5 mediate stress‐dependent responses. SIRT7 exerts context‐dependent effects on hepatic lipid accumulation, which vary with diet and genetic background. Together, these findings reveal a complex SIRT network in NAFLD. Isoform‐specific and context‐dependent modulation, not global activation or inhibition, holds therapeutic promise.

#### 3.3.2 Liver Injury

Liver repair and regeneration are crucial physiological responses to hepatic injury [319]. Liver regeneration after acute injury relies on coordinated mitochondrial quality control, redox homeostasis, and inflammation resolution. In I/R, endotoxin, and toxic injury models, SIRTs have largely protective effects, with notable context‑dependent exceptions [112, 320, 321].

In hepatic IRI, liver‐specific *Sirt1* KO mice exhibit aggravated injury. Mechanistically, SIRT1 deacetylates X‐box binding protein 1 to activate miR‑182, which in turn dampens NLRP3 inflammasome signaling and reduces ER stress‐driven ROS [112, 227]. However, in galactosamine/LPS‑induced liver injury, *Sirt1 KO* mice are paradoxically protected. In this context, SIRT1‐mediated deacetylation of NF‐κB p65 blunts NF‐κB activation and hyperacetylated p65 [228]. Thus, SIRT1 can either attenuate or facilitate liver injury depending on the dominant stress pathway.

In the Sirt2 KO mice, I/R triggered less liver damage. Mechanistically, SIRT2‐dependent deacetylation and inhibition of MAPK phosphatase‑1 lead to sustained MAPK activation and heightened proinflammatory cytokine output [229]. In contrast, SIRT3 acts as a stress buffer. *Sirt3* deficiency increases superoxide‐mediated liver damage and oxidative stress after irradiation and exacerbates liver fibrosis and inflammation in a CCl_4_‐induced fibrosis model [105, 129, 130].

SIRT6 protects against cholestatic injury by deacetylating and destabilizing estrogen‐related receptor gamma, which suppresses cholesterol 7‐alpha hydroxylase‐mediated bile acid synthesis and prevents lipotoxic mitochondrial dysfunction [108, 230]. *Sirt7* KO mice exhibit exacerbated liver fibrosis and increased stellate cell activation after CCl_4_ treatment. SIRT7 protects against liver fibrosis by suppressing stellate cell activation via deacetylation and inhibition of the TGF‐β/SMAD2/3 signaling pathway [231].

Taken together, these findings suggest that hepatic SIRT1, SIRT3, SIRT6, and SIRT7 protect against I/R, radiation, and cholestatic injury through mitochondrial and transcriptional control, whereas SIRT2 can amplify inflammatory signaling in I/R and that SIRT1 can be detrimental under specific endotoxin conditions. Dissecting these context‑dependent roles will be important for translating SIRT‑targeted interventions to acute and chronic liver injury.

#### 3.3.3 Inflammatory Bowel Disease

IBD, including Crohn's disease and ulcerative colitis, reflects a breakdown of intestinal barrier integrity, microbiota‒host interactions and immune regulation [117, 322, 323, 324]. Intestine‐specific SIRT1, SIRT2, and SIRT6 models indicate that SIRTs act at the intersection of epithelial metabolism, barrier maintenance, and mucosal immunity [117, 125, 234, 235, 322].

Studies in intestinal‐specific KO (villin–Cre) mice revealed that *Sirt1* deficiency alters the fecal microbiota composition by 4–6 months of age, partly through the dysregulation of bile acid metabolism. This phenotype is abolished by antibiotics, indicating that SIRT1 mitigates intestinal inflammation by maintaining a balanced gut microbiota [117]. Moreover, epithelial *Sirt1* OE stabilizes β‐transducin repeat‐containing protein 1 and downregulates Snail1, preserving tight and adherent junctions and ameliorating dextran sulfate sodium (DSS) colitis [233], whereas smooth muscle‐specific *Sirt1* OE impairs mucosal repair via inhibition of HIF‑1α nuclear translocation [232]. Thus, SIRT1 exerts compartment‑specific effects within the gut wall.

*Sirt2* deficiency does not induce spontaneous intestinal inflammation but exacerbates DSS‐induced colitis by enhancing NF‐κB acetylation and impairing the M2‐associated anti‐inflammatory pathway [125, 234]. In a cold‑stress model, chronic cold exposure damages the ileal epithelial barrier and increases intestinal permeability, which can be alleviated by *Sirt2* KO through enhanced autophagy [110]. These findings suggest that SIRT2 can both promote and restrain inflammation depending on the dominant stressor and cell type [125].

SIRT6 also plays dual roles. The intestinal‐specific knockout of *Sirt6* in mice results in increased susceptibility to DSS‐induced colitis, partly because LPS suppressed the expression of R‐spondin‐1, a crucial intestinal epithelial growth factor, specifically in the epithelium of Sirt6 KO mice but not in that of WT controls [235]. In contrast, *Sirt6* deficiency in Group 3 innate lymphoid cells increases IL‐22 production and protects against Citrobacter rodentium infection and DSS‐induced colitis, indicating that SIRT6 restrains Group 3 innate lymphoid cell‑driven mucosal immunity in a cell‐intrinsic manner [112].

#### 3.3.4 Severe Acute Pancreatitis

Acute pancreatitis is a form of pancreatic autodigestion caused by enzymatic activation [325]. Approximately 20% of acute pancreatitis cases progress to SAP, a life‐threatening condition characterized by enzyme‑mediated autodigestion, multiorgan failure, and high mortality [326]. Recent work has begun to implicate SIRT4 in SAP pathogenesis using l‐arginine‐induced SAP in *Sirt4* KO and adeno‐associated virus (AAV)‐mediated *Sirt4* OE mice [111]. *Sirt4*‐deficient mice develop more severe pancreatic necrosis and remote lung and kidney injury, accompanied by reduced levels of antioxidant (glutathione and superoxide dismutase) defenses and increased levels of oxidative and lipid peroxidation markers. Mechanistically, SIRT4 modulates HIF‐1α/HO‐1 signaling to influence ferroptosis: *Sirt4* loss upregulates HIF‐1α and ferroptosis‐related proteins, whereas *Sirt4* OE suppresses this axis and attenuates tissue damage [111].

These findings highlight SIRT4 as a critical regulator of oxidative damage and ferroptosis in SAP. Targeting SIRT4‐mediated HIF‐1α/HO‐1 signaling may offer a novel strategy for mitigating SAP progression. Given the emerging role of SIRT4 in SAP pathogenesis, further studies are warranted to explore its therapeutic potential.

#### 3.3.5 Research Limitations and Future Perspectives

*Sirt1–7* consistently places SIRT1, SIRT3, and SIRT6 at the center of hepatic and intestinal homeostasis across digestive diseases. SIRT1 coordinates β‐oxidation, gluconeogenesis, and bile acid/microbiota signaling. SIRT3 preserves mitochondrial integrity and redox balance. SIRT6 restrains lipogenesis, fibrosis, and barrier failure. SIRT2, SIRT4, SIRT5, and SIRT7 exert context‐dependent effects on glycolysis, amino acid handling, ER stress, and stellate cell activation.

Moreover, the experimental landscape is incomplete. Most studies rely on global or single‑tissue knockouts, with few OE, catalytic‑dead, or point‑mutant alleles and limited use of inducible systems to separate developmental from adult roles. SIRT4, SIRT5, and SIRT7 remain largely unexplored. Moreover, chronic, microbiota‐friendly models are rarely used. Future work will require multiallelic designs, systematic age‑ and sex‑stratification, and integration with human‑relevant liver and intestinal organoids to define when and how individual SIRTs or specific SIRT axes can be safely targeted in NAFLD, liver injury, IBD, and SAP.

### 3.4 Transgenic SIRT Family Mouse Models of Nervous System Diseases

**FIGURE 4:**
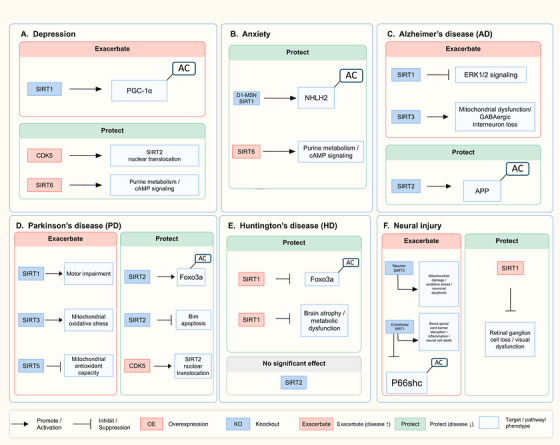
Sirtuins in nervous system diseases. This schematic summarizes the protective (green) and detrimental (red) roles of sirtuins in nervous system diseases on the basis of OE (pink) and KO (blue) mouse models. The arrows indicate activation/promotion, the blunt‐ended lines indicate inhibition/suppression, and “AC” denotes acetylation‐related regulation. (A) The suppression of SIRT1 and SIRT6 expression regulates PGC‐1α activity, purine metabolism, and cAMP signaling; SIRT2 nuclear translocation is also involved. (B) Anxiety SIRT1 (in D1 receptor‐expressing neurons) and SIRT6 modulate NHLH2, purine metabolism, and cAMP signaling. (C) AD SIRT1, SIRT2, and SIRT3 affect ERK1/2 signaling, APP acetylation, mitochondrial dysfunction, and GABAergic neuron loss. (D) Parkinson's disease SIRT1, SIRT2, SIRT3, and SIRT5 regulate motor impairment, mitochondrial oxidative stress, antioxidant capacity, Foxo3a, Bim‐mediated apoptosis, and CDK5‐dependent SIRT2 nuclear translocation. (E) HD SIRT1 modulates Foxo3a expression, brain atrophy, and metabolic dysfunction; SIRT2 expression is not significantly altered (gray). (F) Neural injury‐related endothelial SIRT1 and neuronal SIRT3 are involved in mitochondrial damage, oxidative stress, apoptosis, blood–spinal cord barrier disruption, inflammation, p66Shc acetylation, retinal ganglion cell loss, and visual dysfunction. APP, amyloid precursor protein; Bim, Bcl‐2 interacting mediator of cell death; CDK, cyclin‐dependent kinase; ERK, extracellular signal‐regulated kinase; NHLH2, nescient helix‐loop‐helix 2; PGC‐1α, peroxisome proliferator‐activated receptor gamma coactivator 1‐alpha.

In the nervous system, SIRTs converge on a well‐established set of axes—mitochondrial quality control, genome integrity, oxidative and inflammatory stress, and autophagy—but their effects manifest as neurodegeneration, psychiatric traits, and responses to injury. Transgenic mouse models have begun to map how *Sirt1–7* occupy distinct positions in this network across Parkinson's disease (PD), AD, Huntington's disease (HD), mood and anxiety disorders, and traumatic injury. An overview of these phenotypes and pathways is provided in Figure 4 and Table S6; they serve as a guide for the detailed discussion that follows.

#### 3.4.1 Degenerative Diseases

Neurodegenerative diseases, particularly PD, AD, and HD, are characterized by the progressive degeneration and functional impairment of select neuronal populations [327]. Their pathophysiologic features include protein aggregation, neuroinflammation, oxidative stress, and mitochondrial dysfunction [328, 329, 330]. In this context, SIRT1, SIRT3, and SIRT5 generally act as neuroprotective factors, SIRT2 often has opposing or context‐dependent effects, and evidence for SIRT4, SIRT6, and SIRT7 remains incomplete.

##### 3.4.1.1 Parkinson's Disease

Accumulating evidence suggests that metabolic reprogramming of substantia nigra dopaminergic neurons, oxidative stress, and neuroinflammation serve as key drivers of neuronal damage and neurodegeneration in PD [331, 332, 333, 334]. In toxin‐based PD models, SIRT1 and SIRT3 limit dopaminergic neurodegeneration. Compared with WT control mice, *Sirt1* KO mice exposed to 1‑methyl‑4‑phenyl‑1,2,3,6‑tetrahydropyridine (MPTP) show more severe motor impairments [237], which is consistent with the protective role of SIRT1 in substantia nigra dopaminergic neurons. Similarly, *Sirt3* KO mice generated by CRISPR–Cas9 exhibit heightened susceptibility to MPTP‐induced neuronal loss and more pronounced mitochondrial oxidative stress [241], whereas preserved SIRT3 function mitigates degeneration of substantia nigra pars compacta neurons [242, 335, 336]. Additionally, *Sirt5* deficiency aggravates MPTP‐induced nigrostriatal damage, which is consistent with the protective role of SIRT5 against dopaminergic neuron loss in PD through the preservation of mitochondrial antioxidant capacity [243].

In contrast, MPTP‐induced nigrostriatal damage is attenuated in *Sirt2* KO mice [238]. Mechanistically, SIRT2 promotes neurodegeneration by deacetylating Foxo3a and activating Bim to induce apoptosis. However, developmental studies report that *Sirt2* KO mice have fewer dopaminergic neurons in the substantia nigra under basal conditions [239], underscoring the tension between developmental requirements and adult neurotoxicity. Another PD model confirmed that Cdk5‐mediated phosphorylation of SIRT2 induces its nuclear translocation and promotes neuronal death [240].

Despite these significant findings, several critical questions remain unresolved. The precise physiological and pathological functions of SIRT4, SIRT6, and SIRT7 in transgenic mouse models of PD have yet to be fully elucidated. Moreover, whether SIRT proteins can be developed as therapeutic targets for PD remains to be investigated.

##### 3.4.1.2 Alzheimer's Disease

The pathology of AD is characterized by the deposition of amyloid‐β (Aβ) in the brain and the tangling of hyperphosphorylated tau [337]. The amyloidogenic processing pathway involves sequential cleavage of amyloid precursor protein (APP) by β‐secretase and γ‐secretase complex, generating neurotoxic Aβ peptides [338]. Previous studies have shown that SIRT proteins primarily restore protein microenvironment homeostasis by clearing Aβ or hyperphosphorylated tau protein aggregates, consequently alleviating oxidative stress and increasing mitochondrial function [154, 246, 339, 340, 341]. Transgenic mouse models suggest that SIRT1 and SIRT3 support synaptic and neuronal integrity, whereas SIRT2 facilitates amyloidogenic processing.

Specifically, brain‐specific *Sirt1* KO mice exhibit significant cognitive deficits and impaired synaptic plasticity [244]. SIRT1 regulates chromatin remodeling, gene expression, and signaling pathways, such as ERK1/2, and promotes neuronal dendritic complexity. *Sirt2* deficiency in APP/PS1 models has been reported to improve cognitive deficits and reduce Aβ pathology. SIRT2 deacetylates the conserved N‐terminus of APP, and its loss promotes nonamyloidogenic APP processing, increasing the level of soluble APP‐α and reducing the Aβ burden [154, 245]. *Sirt3* haploinsufficiency in App/Ps1 mice (*Sirt3* ^+/−^AppPs1) exacerbated GABAergic neuron loss and neuronal hyperexcitability, phenotypes that were reversible upon *Sirt3* OE [246]. To date, no studies have reported the roles of SIRT4, SIRT5, SIRT6, and SIRT7 in AD using transgenic mice, which represents a clear gap in the field.

##### 3.4.1.3 Huntington's Disease

HD is a rare autosomal dominant neurodegenerative disease characterized by involuntary dance movements, cognitive impairment, and psychosis [342]. Its neuropathological hallmark involves selective degeneration of striatal medium spiny neurons within the basal ganglia circuitry [343]. Emerging evidence from transgenic mouse models implicates SIRT family members in HD pathogenesis. Notably, *Sirt1* OE has neuroprotective effects in HD models, ameliorating brain atrophy, improving motor performance, and modulating FOXO3a expression levels [247]. In contrast, *Sirt2* KO failed to modify disease progression in HD transgenic mice [248]. Current data therefore support SIRT1 as a disease modifier in HD, but the contribution of other SIRTs remains only partially understood.

Overall, neurodegeneration models point to SIRT1, SIRT3, and SIRT5 as principal protectors and to SIRT2 as a potentially tractable prodegenerative factor in specific contexts (PD and AD). However, strain, sex, and age are often incompletely understood, and the role of SIRT4/6/7 in PD/AD/HD at the genetic‐model level remains largely unexplored.

#### 3.4.2 Neuropsychiatric Disorders

Neuropsychiatric disorders, including depression, anxiety, bipolar disorder, and schizophrenia [344], affect a substantial global population [345]. By 2019, more than 100,000 individuals had been screened for anxiety and depression [346]. In mice, SIRT1, SIRT2, and SIRT6 have been linked to mood‐ and anxiety‐related behaviors through cell type‐specific actions in cortical, striatal, and glial networks [249, 250, 251, 252, 347, 348, 349].

##### 3.4.2.1 Depression

Recent studies have demonstrated that SIRT1 plays a critical role in regulating depression‐like behaviors in a brain region‐ and sex‐dependent manner. In Emx1–ires–Cre mice, the endogenous Emx1 locus drives Cre recombinase expression in the vast majority of glutamatergic neurons within the neocortex and hippocampus [249] *Sirt1* Emx1KO mice lack SIRT1 activity in forebrain glutamatergic neurons; these mice exhibit depression‐like phenotypes in males but not females [249]. This study revealed that *Sirt1 *deficiency impaired neuronal excitability and synaptic transmission in medial prefrontal cortex excitatory neurons, suggesting a sex‐specific regulatory role in depression‐related behaviors [249]. SIRT1 enhances neuronal excitability and synaptic transmission by deacetylating substrates such as PGC‐1α, thereby promoting mitochondrial biogenesis and alleviating depressive behaviors. In contrast, *Sirt2* KO mice are resistant to social defeat stress‐induced depressive‐like behaviors [250]. Astrocyte‐specific *Sirt6* KO mice do not spontaneously develop depression‐like behaviors, but reexpression of SIRT6 in astrocytes reverses the antidepressant‐like effects observed upon global or regional reduction in SIRT6 expression [251]. In vitro data indicate that astrocytic SIRT6 influences purine metabolism in the medial prefrontal cortex [251].

##### 3.4.2.2 Anxiety

Anxiety disorders represent the most prevalent class of psychiatric diseases, yet their underlying mechanisms and optimal treatments remain incompletely understood [350, 351].

Emerging evidence suggests that SIRT proteins are involved in the regulation of anxiety through the maintenance of protein homeostasis and metabolism and that *Sirt1* KO in neurons reduces anxiety and social defeat stress susceptibility [252, 352]. Brain‐specific (nestin–Cre) *Sirt1* KO mice exhibit decreased MAO‐A activity, which is mediated through a mechanism in which SIRT1 deacetylates the brain‐specific helix‐loop‐helix transcription factor NHLH2 at lysine 49, thereby enhancing its activation of the MAO‐A promoter [252]. SIRT1 in D1‐expressing medium spiny neurons of the nucleus accumbens coordinates the transcriptional regulation of neural activity by modulating the expression of glutamatergic and GABAergic receptor subunits, which in turn influences electrophysiological, morphological, and behavioral endpoints related to depression and anxiety [253]. Furthermore, SIRT6 has been identified as another important antianxiety regulator in a re‐expression model [251].

Collectively, these findings suggest that SIRT1 and SIRT2 influence neuropsychiatric phenotypes via synaptic, metabolic, and transcriptional mechanisms in defined neuronal populations, whereas SIRT6 acts in astrocytes to shape neuromodulatory tone. However, current models capture only selected symptom dimensions, and translation to human affective disorders diagnosed by heterogeneous symptom clusters remains challenging.

#### 3.4.3 Injury

In acute injury models, SIRT1 and SIRT3 predominantly mitigate secondary damage by regulating oxidative stress, mitochondrial quality, and barrier integrity. In a model of traumatic optic neuropathy induced by blunt head impact, pharmacological activation of SIRT1 with resveratrol or genetic OE of SIRT1 significantly attenuated retinal ganglion cell loss and preserved visual function [254]. This neuroprotective effect was mediated through the deacetylation of key regulatory proteins. In a traumatic brain injury model, *Sirt3 KO* mice (with a fibronectin Type III domain‐containing *KO*‐mediated background) exhibited exacerbated mitochondrial damage, oxidative stress, and neuronal apoptosis [255]. SIRT3‐dependent mitochondrial quality control plays a protective role [255]. Furthermore, in a spinal cord injury model, endothelial‐specific S*irt1* KO exacerbated blood‒spinal cord barrier disruption, leading to increased inflammation and neural cell death, whereas activation of SIRT1 with the agonist SRT1720 ameliorated these effects [119]. SIRT1 was shown to protect the blood–spinal cord barrier by deacetylating p66Shc, thereby reducing oxidative stress and preventing barrier disruption [119].

#### 3.4.4 Research Limitations and Future Perspectives

Taken together, the results of nervous system models reinforce SIRT1 and SIRT3 as central protectors against degeneration and injury, with SIRT2 and SIRT6 exerting more nuanced, often cell type specific, influences on PD, AD, and mood/anxiety behaviors. Moreover, the roles of SIRT4, SIRT6, and SIRT7 in central nervous system disease remain largely unexplored at the genetic level, whereas the role of SIRT5 has been studied in PD but not in AD/HD. Most current models are limited to single‐gene manipulations in a narrow set of strains and ages. More translationally oriented models integrating multiple SIRTs, sex and age differences, and human‐relevant behavioral end points will be needed to clarify when and how targeting the SIRT network can meaningfully modify the course of neurodegenerative and neuropsychiatric disease.

### 3.5 Transgenic SIRT family Mouse Models of the Endocrine System

Endocrine organs sit at the convergence of the five mechanistic axes outlined in Section 2: nutrient sensing and metabolism, genome integrity, cellular stress resilience, immunity, and autophagy [353, 354, 355, 356, 357]. Transgenic mouse models therefore provide a particularly informative view of how these axes are integrated at the organismal level to generate endocrine phenotypes such as diabetes mellitus (DM) and dyslipidemia [358] (Table S7). Broadly, SIRT1, SIRT3, and SIRT6 act as endocrine integrators coordinating liver, pancreatic islets, adipose tissue, and myeloid cells, whereas SIRT2, SIRT4, SIRT5, and SIRT7 serve as context‐dependent modulators that regulate glycolytic, amino acid, and thermogenic programs.

#### 3.5.1 Diabetes Mellitus

DM is fundamentally characterized by hyperglycemia resulting from either absolute or relative insulin deficiency [359, 360, 361]. Building on their roles in metabolism and stress regulation (Sections 2.1 and 2.3), SIRT1, SIRT3, SIRT4, and SIRT6 have emerged as central regulators of glucose homeostasis in vivo [18, 362]. In liver‐specific *Sirt1* KO mice, impaired gluconeogenesis and increased ROS levels lead to persistent hyperglycemia and systemic insulin resistance [26], whereas podocyte‐specific *Sirt1* deletion exacerbates diabetic nephropathy (DN) via increased acetylation and activation of NF‑κB p65 and signal transducer and activator of transcription 3 [91], highlighting the dual role of SIRT1 in hepatic and renal protection in DM models. Under HFD conditions, *Sirt2* KO mice exhibit impaired muscle insulin sensitivity, enhanced hepatic insulin resistance, and excessive weight gain [30], in part through reduced deacetylation of pyruvate kinase M2 and altered glycolytic flux, indicating a protective role for SIRT2 against overnutrition‐induced metabolic dysfunction [256].

*Sirt3* contributes primarily through β‑cell and brown adipose tissue mitochondria [103]. Islets from *Sirt3* KO mice displayed elevated SOD2 acetylation, which compromised both glucose‐stimulated insulin secretion and ATP production [103]. *Sirt3* deficiency in aging mice led to abnormal acylcarnitine metabolism and impaired thermogenesis in brown adipose tissue (BAT), further linking SIRT3‐dependent mitochondrial quality control to systemic glucose tolerance and energy expenditure [99].

SIRT4 adds an amino acid‐centered layer of control to glucose homeostasis. In β‐cells, SIRT4 suppresses amino acid‐stimulated insulin secretion through the ADP‐ribosylation‐mediated inhibition of glutamate dehydrogenase. Global *Sirt4 KO* mice therefore show increased insulin release, which may be beneficial acutely but can be predisposed to hyperinsulinemia and insulin resistance under conditions of chronic nutrient excess [43]. Consistently, C57BL/6NJ global *Sirt4* KO mice exhibit the methylcrotonyl‐CoA carboxylase complex hyperacylation, reduced leucine/BCAA flux, increased glutamate dehydrogenase activity, and increased glucose‐ and leucine‐stimulated insulin secretion, and they develop age‐dependent hyperinsulinemia, glucose intolerance, insulin resistance, and hyperglycemia [10]. A mixed 129Sv×C57BL/6J *Sirt4* KO line shows a similar age‐related shift toward hyperglycemia, hyperinsulinemia, and insulin resistance, with only modest changes in body weight and plasma lipids [223]. In contrast, β‐cell‐specific *Sirt4* deletion (MIP–CreERT1; C57BL/6J) leaves body weight, glucose tolerance, and glucose‐ or leucine‐stimulated insulin secretion unchanged even in aged mice, indicating that whole‐body phenotypes cannot be explained by a dominant intrinsic role in β‐cells [44]. Adipose‐specific *Sirt4* KO (Adipoq–Cre; C57BL/6) increases BCAA levels in gonadal white adipose tissue and the plasma BCAA/ketoacid ratio without affecting body weight, food intake or adipocyte morphology, which is consistent with impaired adipose BCAA catabolism [257]. Taken together, these findings suggest that SIRT4 restrains BCAA‐driven insulin secretion and delays age‐related insulin resistance mainly through coordinated actions in β‐cells and liver and adipose tissue.

SIRT6 connects the chromatin‐level control of inflammatory and metabolic genes to endocrine outputs. Myeloid *Sirt6* deficiency in mice leads to obesity and severe insulin resistance, characterized by reduced norepinephrine concentrations in BAT and impaired thermogenic function [258]. In contrast, knocking out muscle‐specific *Sirt6* expression decreases the expression of key metabolic genes involved in glucose/lipid uptake and FAO through attenuated AMPK phosphorylation [259]. These models collectively highlight how SIRT6 coordinates myeloid inflammation, BAT function, and muscle substrates to shape insulin sensitivity.

In BAT, *Sirt7* deficiency enhances thermogenesis and energy expenditure by deacetylating IGF2 mRNA‐binding protein 2 and relieving the translational repression of UCP1, increasing UCP1 and fibroblast growth factor 21 levels, and improving systemic metabolism and lifespan in some settings [260, 261].

Collectively, the results of the DM models support a layered endocrine network in which SIRT1, SIRT3, SIRT4, and SIRT6 preserve insulin sensitivity and secretory capacity across liver–islet–BAT–myeloid axes, whereas SIRT2, SIRT4, and SIRT7 alter glycolytic, amino acid‐driven insulin secretion, and thermogenic responses in a diet‐, age‐, and tissue‐dependent manner.

#### 3.5.2 Hyperlipidemia

Hyperlipidemia, a prevalent metabolic disorder in aging populations, is clinically defined by elevated low‐density lipoprotein (LDL) cholesterol levels [363, 364]. Hyperlipidemia emerges in Sirt‐modified mice as a systems‐level consequence of altered hepatic lipid handling, lipoprotein metabolism, and thermogenesis.

Global *Sirt1* deletion reduces body weight but causes severe developmental abnormalities rather than metabolic health [21, 22]. In contrast, *Sirt1*‑KI mice with moderate OE in adipose tissue and the brain have lower body weight and fat mass, improved cholesterol profiles, reduced adipokines and insulin, and lower fasting glucose levels, indicating that carefully titrated SIRT1 activation can beneficially remodel lipid and glucose metabolism [365, 366, 367]. Hepatocyte‑specific *Sirt1* KO mice, however, developed pronounced hepatic steatosis and hypercholesterolemia when challenged with a HFD [262]. These findings confirm that SIRT1 is a regulator of FAO and cholesterol homeostasis in the liver.

SIRT2 also contributes to lipid homeostasis. Global *Sirt2* KO mice fed a HFD exhibit dyslipidemia characterized by elevated triglyceride levels and free fatty acid levels, partly through derepression of SREBP‐1c‐dependent lipogenesis [30].

SIRT3 and SIRT6 influence lipid profiles through mitochondrial FAO and chromatin‐mediated control of lipogenesis. *Sirt3*‐haploinsufficient mice accumulate FAO intermediates and triglycerides during fasting, which is consistent with impaired hepatic FAO [263]. Conversely, *Sirt6* OE mice on a C57BL/6 background maintain lower LDL levels and are protected from diet‐induced obesity and dyslipidemia, in accordance with SIRT6‐dependent repression of lipogenic genes and promotion of fatty acid disposal [57].

SIRT4 also modulates lipid handling under fasting conditions. In fasted 129/Sv global *Sirt4* KO mice, increased expression of PPARα target genes, increased hepatic FAO, increased NAD^+^ levels, and elongated mitochondria are observed [46]. Mechanistically, SIRT4 normally represses PPARα activity by limiting NAD^+^ and SIRT1; its loss activates SIRT1, which binds to PPARα and promotes lipid catabolism [46]. Another study revealed that *Sirt4 KO* mice have reduced ATP levels in the liver and muscle, increased AMPK and acetyl‐CoA‐carboxylase phosphorylation, and elevated PGC‐1α expression, suggesting that SIRT4 acts as a brake on fasting‐induced FAO and mitochondrial remodeling [45].

SIRT5 modulates amino acid and nitrogen disposal, which may indirectly affect lipid metabolism under stress [46, 51, 52]. *Sirt7* KO mice fed a HFD exhibit lower plasma cholesterol and triglyceride levels, which is consistent with reduced lipogenesis [63].

Overall, SIRT1, SIRT3, and SIRT6 protect against dyslipidemia, whereas SIRT4 acts as a brake on fasting‐induced lipid combustion. SIRT2 promotes lipid accumulation under a HFD, and SIRT7 deficiency decreases plasma lipid levels. The roles of SIRT5 remain incompletely defined and warrant further investigation.

#### 3.5.3 Research Limitations and Future Perspectives

In summary, the endocrine phenotypes of *Sirt1–7* mice reveal that SIRTs organize glucose and lipid control in a hierarchical way. SIRT1, SIRT3, and SIRT6 act as core regulators in the liver, pancreatic islets, adipose tissue, and brown fat, influencing insulin secretion, insulin sensitivity, and systemic lipid handling. SIRT2, SIRT4, SIRT5, and SIRT7 fine‑tune more specific processes, including glycolytic flux, amino‑acid‐stimulated insulin release, nitrogen disposal, and thermogenic capacity. Endocrine SIRT research remains incomplete. Most data come from global or single‑tissue KOs, with few OE, catalytic‑dead or point‑mutant models and little temporal control. SIRT2, SIRT5, and SIRT7 are still poorly characterized in vivo, and interactions between metabolic organs and the central nervous system or gut are underexplored.

Because glucose and lipid homeostasis emerge from coordinated crosstalk among multiple organs and cell types, single *Sirt* genes can exert distinct, sometimes opposing, effects in different tissues, making their net impact on whole‐body metabolism inherently complex. Transgenic mouse models are therefore indispensable for pinpointing the key organs in which individual SIRTs are rate‐limiting and for dissecting organ‐specific and cooperative actions that cannot be resolved in human studies. Mechanistic evidence derived from these models is critical for identifying druggable nodes, prioritizing target tissues and clarifying the logical basis for SIRT‐directed therapy in diabetes and dyslipidemia. Future work should use multiallelic, age‐, and sex‐stratified models and combined manipulations to define where and when individual SIRTs can be targeted safely in endocrine disease.

### 3.6 SIRT Family Transgenic Mouse Models of Urogenital System Diseases

In the urogenital system, SIRTs modulate mitochondrial function, redox homeostasis, inflammation, autophagy, and chromatin regulation; however, their disruption manifests as acute and chronic kidney injury and impaired fertility. Global and tissue‐specific *Sirt1–7* mouse models have been particularly informative for mapping how these pathways operate in the tubular epithelium, glomerular cells, renal vasculature, and reproductive axis, including in oocytes, trophoblasts, germ cells, and Leydig cells. These phenotypes and pathways are summarized in Table S8, and a framework for detailed discussion is provided below.

#### 3.6.1 Acute Kidney Diseases

##### 3.6.1.1 I/R‐Induced AKI

In global *Sirt3*‐deficient mice, mitochondrial dynamics are disturbed, resulting in excessive fission and impaired fusion; this imbalance precipitates early fibrosis after IRI through altered mitofusin‐2 ubiquitination and degradation [266]. These effects are sexually dimorphic, stemming from differential hormonal regulation: estrogen upregulates mitochondrial SIRT3, whereas testosterone suppresses it. Consequently, compared with females, C57BL/6J mice with *Sirt3* OE exhibit more robust protection [266].

In contrast, SIRT7 influences the inflammatory phase. Global *Sirt7* KO mice exhibit reduced NF‐κB activation and are partially protected from IRI‐induced AKI, with decreased inflammatory cell infiltration and tubular damage [139]. Together, these models place mitochondrial SIRT3 upstream in limiting ROS‐driven injury and nuclear SIRT7 downstream in shaping the inflammatory response.

##### 3.6.1.2 Infection‑Induced AKI

In infection‐related AKI, SIRT2 and SIRT3 modulate distinct nodes of innate immunity. In LPS‐induced gram‐negative sepsis, global *Sirt2* KO C57BL/6J mice displayed decreased renal C‐X‐C motif chemokine ligand 2 and C‐C motif ligand 2 expression, reduced neutrophil and macrophage infiltration, and attenuated acute tubular injury and dysfunction. At the molecular level, SIRT2 promotes renal inflammatory injury by regulating C‐X‐C motif chemokine ligand 2/C‐C motif ligand 2 transcription through MAPK phosphatase‐1/MAPK signaling and p65 binding at its promoter [265].

As a mitochondrial deacetylase, SIRT3 links metabolism to inflammasome activation in sepsis‐associated AKI. In cecal ligation and puncture models on a C57BL/6 background, global *Sirt3* KO exacerbates renal dysfunction, characterized by mitochondrial alterations and ROS production. Mechanistically, this reflects heightened NLRP3 inflammasome activation, oxidative stress, and tubular apoptosis [267]. In LPS‐induced AKI, SIRT3 protects renal tubular cells through YME1‐like 1 adenosine triphosphatase deacetylation and subsequent optic atrophy 1‐mediated mitochondrial fusion. This preservation of mitochondrial integrity curtails apoptosis and attenuates pathological injury [267, 268].

##### 3.6.1.3 Drug‑ and Contrast‑Induced AKI

Cisplatin and contrast agents are major causes of hospital‐acquired AKI, in which oxidative stress and inflammation reinforce each other [102, 368, 369]. Individual SIRTs target specific steps in this pathogenic cascade. Global *Sirt1*ΔE4 heterozygous C57BL/6 mice exhibit heightened vulnerability to cisplatin‐induced AKI, manifested as exacerbated renal damage and ROS accumulation. Molecular characterization revealed that SIRT1 protects cells via dual specificity phosphatase 16 and FOXO3 deacetylation, thereby modulating c‐Jun N‐terminal kinase signaling, enhancing autophagy, and limiting apoptosis under cisplatin or hypoxic stress [145, 264].

SIRT3 plays a parallel mitochondrial role in contrast nephropathy. In 129S1/SvImJ mice, global *Sirt3* KO aggravates contrast‐induced AKI, which is characterized by renal dysfunction, histological injury, oxidative stress, and apoptosis. This phenotype reflects the loss of SIRT3‐mediated protection against ROS accumulation and apoptotic signaling [102].

SIRT5 and SIRT6 provide additional nuclear–mitochondrial control. In B6;129 *Sirt5*‐null mice, proximal‐tubular cells exhibit dysregulated mitochondrial and peroxisomal FAO, yet these animals exhibit improved survival following IRI or cisplatin challenge [270]. This paradox suggests that SIRT5 normally constrains FAO and ROS production in a context‐specific manner. In 129 Sv/C57BL/6 mice, global *Sirt6* OE (∼4‐fold) attenuated cisplatin‐induced renal dysfunction, inflammation, and apoptosis through the suppression of ERK1/2 signaling [138]. Conversely, global *Sirt6* KO in C57BL/6 mice exacerbates cisplatin injury and ferroptosis, reflecting BRCA1‐associated protein 1 derepression and disrupted System Xc^−^/glutathione peroxidase 4 activity [109].

In contrast, SIRT2 and SIRT7 tend to facilitate cisplatin injury. Global *Sirt2* KO in C57BL/6J mice confers renal protection against cisplatin, as evidenced by improved kidney function, diminished tubular damage, and enhanced survival. Mechanistically, this reflects preserved MAPK phosphatase‐1 expression, which dampens MAPK/NF‐κB activation and subsequently decreases tumor necrosis factor alpha (TNF‐α)/IL‐6 release [127]. Global *Sirt7* KO in C57BL/6J mice also confers protection against cisplatin‐induced AKI, characterized by attenuated proinflammatory cytokines and diminished renal damage. This phenotype mirrors the normal role of SIRT7 in driving NF‐κB p65 nuclear translocation and TNF‐α expression; its absence thus alleviates inflammatory injury [140].

Overall, these models suggest that SIRT1, SIRT3, SIRT5, and SIRT6 broadly counteract toxic AKI through redox, FAO, and ferroptosis control, whereas SIRT2 and SIRT7 often amplify inflammatory signaling, and their loss can be renoprotective.

##### 3.6.1.4 Obstruction‑Induced AKI

In unilateral ureteral obstruction (UUO), SIRT1 and SIRT6 limit fibrosis progression via distinct transcriptional pathways. Proximal‐tubule *Sirt1* deletion (Ggt1–Cre; C57BL/6J) stabilizes HIF‐2α after UUO, driving persistent expression of profibrotic genes and extracellular‐matrix accumulation [92]. Endothelial *Sirt1* KO (Tie2–Cre; B6;129) reduces MMP‐14 activity, impairs microvascular remodeling, and exacerbates tubulointerstitial fibrosis after folic acid nephrotoxicity [273]. Proximal‐tubule *Sirt6* KO similarly worsened UUO‐induced inflammation and fibrosis via increased β‐catenin acetylation and activation of downstream extracellular matrix targets [137]. These findings indicate that SIRT1 maintains microvascular and tubular homeostasis, whereas SIRT6 inhibits β‐catenin‐dependent fibrotic transcription, jointly slowing the transition from obstructive AKI to chronic kidney disease (CKD).

#### 3.6.2 Chronic Kidney Diseases

Epidemiological studies have shown that AKI survivors are at high risk of CKD, which is projected to become the fifth leading cause of years of life lost worldwide by 2040 [370, 371]. *Sirt*‐modified mice are particularly informative for diabetic and hypertensive nephropathy and CKD‐associated vascular calcification.

##### 3.6.2.1 Diabetic Nephropathy

In DN, disordered metabolism, oxidative stress, and inflammation reinforce one another. SIRT1 and SIRT2 exert opposing effects within this network.

SIRT1 preserves renal homeostasis through metabolic, antioxidant, and anti‑inflammatory mechanisms. In offspring exposed to a maternal HFD, global *Sirt1* OE normalizes renal triglyceride content and hypertrophy and reduces oxidative markers, inflammation, and albuminuria [90]. Proximal‑tubule‑specific *Sirt1* KO (γGT–Cre; C57BL/6J) aggravates glomerular lesions and proteinuria in streptozotocin‑induced and obese‑type diabetic mice by disturbing the nicotinamide mononucleotide‐NAD^+^ balance around glomeruli and derepressing claudin‑1 in podocytes [271]. Podocyte‑specific *Sirt1* KO (NPHS2–rtTA; Dox‑inducible) accelerates aging‐induced glomerulosclerosis, albuminuria, and oxidative stress, which are associated with impaired PGC‑1α and FOXO3/FOXO4 expression and enhanced NF‑κB signaling [272].

In contrast, SIRT2 amplifies tubular inflammation. Global *Sirt2* KO attenuates renal injury and inflammation in HFD/streptozotocin‐induced DN. This protection stems from suppressed tubular AP‐1 activity, which reflects decreased c‐Jun/c‐Fos deacetylation and the consequent downregulation of downstream inflammatory genes [126]. Thus, SIRT1 acts as a protective barrier against metabolic and inflammatory injury, whereas SIRT2 acts as a proinflammatory amplifier in DN.

##### 3.6.2.2 Hypertension‑Induced Nephropathy and CKD‑Associated Vascular Calcification

In hypertensive and CKD contexts, mROS and dysregulated lipid and mineral metabolism shape both parenchymal and vascular damage.

SIRT6 protects podocytes and the vasculature. Podocyte‑specific *Sirt6* KO (NPHS2–Cre; C57BL/6J) impairs ATP‐binding cassette subfamily G member 1‐mediated cholesterol efflux under Angiotensin II, leading to lipid accumulation and podocyte injury [274]. In CKD‐associated vascular calcification, VSMC‐specific *Sirt6* knockdown (AAV2–Cre; C57BL/6J) accelerates medial calcification by derepressing Runx2, promoting osteogenic transdifferentiation of VSMCs [275]. Together, these models indicate that SIRT6 guards against podocyte lip toxicity and vascular calcification.

#### 3.6.3 Infertility

##### 3.6.3.1 Female Infertility

Female fertility depends on oocyte quality, placental development, and mammary gland function. In these processes, SIRT1 plays a dominant role, with SIRT3 providing partial mitochondrial support.

Oocyte‑specific *Sirt1* KO (Zp3–Cre) leads to ROS accumulation, chromosomal instability, and an age‑dependent decline in fertility, with ∼50% of females becoming sterile between 9 and 11 months of age because of compromised oocyte quality [93]. Global *Sirt1* deficiency also impairs autophagic flux by reducing the deacetylation of core autophagy proteins (e.g., LC3 and autophagy‐related protein 7), causing the accumulation of damaged organelles and perinatal lethality [144], which is consistent with its fundamental role in cellular nutrient adaptation. In contrast, global *Sirt3* KO (129S1/SvImJ) increases oocyte ROS but maintains ATP levels and female fertility under both standard and HFDs, likely reflecting compensation by increased mitochondrial mass and SIRT1‐PGC‑1α‑driven biogenesis [280].

SIRT1 is further required for placental and mammary function. In *Sirt1*‐null 129/Sv mice, the placenta is small, with morphological defects and blunted trophoblast differentiation, indicating that it plays an essential role in placental formation [278]. In mixed 129SvJ/C57BL6 mice, global *Sirt1*ΔE4 KO compromises mammary development, resulting in defective ductal morphogenesis and lactation failure. Mechanistically, this reflects aberrant estrogen–IGF‐1 signaling [279]. Trophoblast‐specific *Sirt1* deletion (Elf5–Cre; C57BL/6 N) compromises placental development, inducing senescence and fetal growth restriction. Mechanistically, SIRT1 normally drives trophoblast epithelial‐mesenchymal transition through vimentin deacetylation; its loss disrupts this transition [276]. Together, these models establish SIRT1 as a key safeguard of female reproduction from oocyte maturation through placentation to postnatal nutrition.

##### 3.6.3.2 Male Infertility

In males, SIRT1 likewise governs spermatogenesis and endocrine function. Germ cell‐specific deletion (Tnap–Cre or SF1/Tnap combinations) results in severe infertility, accompanied by reduced testis size and profound sperm defects—specifically, malformed heads and defective acrosomes [23, 277]. At the molecular level, SIRT1 deacetylates LC3 and autophagy‐related protein 7 in spermatids to preserve autophagic flux and organelle turnover. Consequently, *the* loss of *Sirt1* causes residual body retention, epididymal round cell degeneration, and impaired motility and fertilization failure [23, 277]. Global *Sirt1* deficiency further increases germ‐apoptosis in a p53‐dependent manner, depleting the seminiferous epithelium [21].

Environmental insults converge on this regulatory axis. In a PM_2_._5_ exposure model, SF1–Cre‐mediated *Sirt1* deletion in Leydig cells exacerbated testicular injury, manifested as ferroptosis, cellular vacuolation, and subsequent testosterone decline and deterioration of sperm quality. Mechanistically, SIRT1 normally constrains ferroptosis and ROS‐driven damage in interstitial cells through HIF‐1α‐dependent signaling [89].

#### 3.6.4 Research Limitations and Future Perspectives

Across urogenital models, distinct SIRTs govern renal and reproductive integrity through divergent strategies. SIRT1, SIRT3, and SIRT6 function as conserved guardians, leveraging NAD^+^‐dependent mechanisms—mitochondrial quality control, autophagy, redox homeostasis, and transcriptional regulation—across tubular, glomerular, vascular, and germline compartments. Conversely, SIRT2, SIRT5, and SIRT7 modulate inflammatory and metabolic pathways in a context‐specific manner; their activity can paradoxically worsen AKI or cisplatin nephrotoxicity, whereas their removal confers protection. Notably, SIRT4 remains largely uncharacterized in these tissues.

Current evidence, however, suffers from constraints: reliance on single‐gene, germline manipulations within limited strain backgrounds and a pronounced male bias. Functional redundancies, such as SIRT1–PGC‐1α compensation for SIRT3 deficiency and incomplete substrate maps for SIRT4, SIRT5, and SIRT7, further complicate therapeutic targeting. Closing these gaps demands temporally and spatially precise models, sex‐ and age‐stratified analyses, and human‐relevant systems, including kidney and gonadal organoids coupled with single‐cell multiomics. Only through such integrated approaches can we delineate when and how SIRT network modulation might be harnessed to prevent or treat AKI, CKD, and infertility.

### 3.7 Transgenic SIRT Family Mouse Models of the Musculoskeletal System

Musculoskeletal aging is characterized by progressive bone loss, cartilage degeneration, and a decrease in muscle mass and strength, leading to osteoporosis, OA, and sarcopenia. In this context, *Sirt1–7* transgenic mouse models highlight how SIRTs shape bone remodeling (osteoblasts, osteoclasts, and stromal cells), cartilage homeostasis (chondrocytes), and muscle regeneration (satellite cells and myofibers). Global and tissue‑specific *Sirt1–7* KO and OE lines exhibit strong cell type and context dependence: the same isoform can be protective in one compartment and detrimental in another. These musculoskeletal phenotypes are summarized in Table S9, and the stages for the detailed analysis are presented below.

#### 3.7.1 Osteoporosis

Osteoporosis, a metabolic bone disease characterized by reduced bone mass and increased fracture risk, is highly prevalent among postmenopausal women and older adults [282, 372, 373].

C57BL/6J mice subjected to chronic energy deprivation (separation‐based anorexia) exhibit bone loss and marrow fat accumulation upon global *Sirt1* deletion. SIRT1 normally maintains bone mass by deacetylating Runx2 and FOXO1. This epigenetic modification promotes the differentiation of bone marrow stromal cells toward osteoblasts rather than adipocytes [284]. Consistent with the results of the KO experiments, mesenchymal‐specific *Sirt1* OE (Prx1–Cre) increased alveolar bone mass. SIRT1 promotes osteoblastic differentiation through Bmi1 deacetylation. This epigenetic modification induces nuclear translocation of Bmi1 to support bone formation [283].

SIRT6 restrains osteoclast‐mediated bone resorption via a two‐cell mechanism. Osteocalcin–Cre‐driven *Sirt6* KO in osteoblasts/osteocytes increases the expression of the receptor activator of NF‐κB ligand and decreases osteoprotegerin expression, thereby promoting osteoclastogenesis and resulting in reduced bone mass [287]. Myeloid‐specific *Sirt6* deletion (LysM–Cre) accelerates cancellous bone loss in sham and ovariectomized B6/129 mice. These animals exhibit reduced estrogen receptor alpha protein expression and excessive osteoclast formation. SIRT6 normally preserves bone by deacetylating estrogen receptor alpha. This stabilizes the receptor and suppresses osteoclast‐mediated resorption [288]. These data support a “two‐cell” model in which SIRT6 limits osteoclast formation from the osteoblast/osteocyte side and promotes osteoclast apoptosis within the myeloid compartment.

In contrast, SIRT3 facilitates bone resorption in aging and estrogen‐deficient conditions. B6;129 global *Sirt3*KO mice exhibit increased bone mass at baseline. SIRT3 drives osteoclastogenesis by increasing mitochondrial function and mitophagy in osteoclasts. This mechanism underlies age‐ and hormone deficiency‐induced skeletal loss [285]. Osteoclast‐specific *Sirt3* KO (LysM–Cre; C57BL/6) attenuates aging‐associated bone loss in female mice. Mutant osteoclasts exhibit reduced function and mitochondrial dysfunction. Consistent with these genetic findings, the *Sirt3* inhibitor LC‐0296 selectively decreases mitochondrial respiration in osteoclasts derived from aged mice [285, 286]. These findings suggest that SIRT3 maintains osteoclast bioenergetics through oxidative phosphorylation and ATP synthesis. Genetic ablation of *Sirt3* preferentially protected against age‐related bone loss, whereas ovariectomy‐induced bone loss remained unaffected.

Studies on bone biology have demonstrated that SIRT1 promotes osteogenic function and osteogenic differentiation. SIRT6 limits osteoclast formation and viability. In contrast, SIRT3 exacerbates osteoclast‐driven bone resorption in the context of hormone deficiency and aging. Given these antagonistic functions, therapeutic strategies should target specific cell types rather than systemically modulating SIRT activity.

#### 3.7.2 Osteoarthritis

OA is a degenerative joint disease characterized by cartilage loss, osteophyte formation, and chronic inflammation, especially in weight‐bearing joints [374, 375]. *Sirt1* and *Sirt3* models indicate that both SIRTs support cartilage homeostasis via protein quality control and antioxidant defense.

C57BL/6 mice with posttraumatic or age‐dependent OA exhibit elevated serum N‐terminal/C‐terminal SIRT1 fragment ratios. Cartilage‐specific *Sirt1* deletion (α‐tubulin–Cre) accelerates cartilage degradation and worsens OA severity. These mice also have reduced N‐terminal/C‐terminal SIRT1 ratios. Full‐length SIRT1 therefore maintains cartilage integrity [376]. Global *Sirt1^Y/Y^* mice express catalytically inactive SIRT1. These animals develop cartilage defects in the skull, spine, ribs, and joints. Their chondrocytes exhibit impaired FOXO‐dependent autophagy. The clearance of damaged proteins is reduced. Type II collagen and proteoglycan synthesis decreases. MMPs increase. SIRT1 therefore preserves cartilage by maintaining autophagy‐linked protein quality control and anabolic gene expression [290]. Cartilage‐specific *Sirt1* KO (Aggrecan‐enhancer; C57BL/6) exacerbates posttraumatic OA. Mutant mice display marked cartilage mineralization and structural deterioration, predominantly affecting the lateral compartment. SIRT1 restrains the expression of lymphoid enhancer binding factor 1 to inhibit Wnt signaling and inflammation. Loss of this regulation accelerates disease progression [289].

SIRT3 preserves cartilage through antioxidant defense. In OA with HFD‐induced obesity, global *Sirt3* KO C57BL/6J mice develop early‐stage disease. These animals exhibit elevated chondrocyte ROS levels and decreased SOD2 activity. Cartilage destruction and synovitis are also more severe. SIRT3 normally deacetylates and activates SOD2. This enzymatic modification sustains the antioxidant capacity of chondrocytes. In the absence of SIRT3, ROS accumulate and degrade the matrix [104].

These findings implicate SIRT1 in chondrocyte autophagy and Wnt/lymphoid enhancer binding factor 1 signaling. They also establish SIRT3 as a mitochondrial antioxidant in cartilage. Most studies, however, rely on germline or lifelong tissue‐specific mutations. Whether these interventions work safely in adult‐onset, load‐bearing or metabolic challenge models that are closer to human OA requires further validation.

#### 3.7.3 Age‐Related Sarcopenia Pathogenesis

Neuromuscular junctions and skeletal muscle deteriorate with advancing age, leading to sarcopenia and frailty. Transgenic mouse models have demonstrated that SIRT1 and SIRT6 protect neuromuscular integrity through distinct mechanisms. SIRT1 acts centrally and at the neuromuscular synapse. SIRT6 supports satellite‐cell‐mediated repair and myofiber homeostasis.

Prion–Cre‐mediated *Sirt1* OE in C57BL/6J mice maintains young neuromuscular junction morphology. Mutants display more terminal Schwann cells and greater innervation density. In contrast, restricting *Sirt1* deletion to the medial dorsal hypothalamus hastens neuromuscular junction deterioration. These findings indicate that hypothalamic SIRT1 safeguards peripheral junctions via autonomic output and central neural control [291]. Muscle‐creatine kinase–Cre‐driven *Sirt1* deletion leaves baseline muscle performance and regeneration intact. However, satellite‐cell‐specific KO (Pax7–Cre) in aged mice diminishes maximal force and impairs postinjury repair. SIRT1 normally activates satellite cells and enhances mitochondrial function. These changes enhance muscle regeneration and contractile performance. This process involves p53 and metabolic regulators [292].

Global *Sirt6* KO 129SvJ mice develop muscle wasting. These animals show reduced fiber size and increased fibrosis. Myostatin expression also increased. SIRT6 normally suppresses NF‐κB at the myostatin promoter. Without this enzyme, NF‐κB signaling becomes unchecked. This drives myostatin‐mediated skeletal muscle atrophy [293].

#### 3.7.4 Research Limitations and Future Perspectives

SIRT1 and SIRT6 protect musculoskeletal tissues. SIRT1 promotes osteogenic function and osteogenic differentiation. It also maintains the cartilage matrix and supports satellite‐cell‐mediated muscle repair. In contrast, SIRT6 suppresses osteoclastogenesis and induces osteoclast apoptosis. In muscle, it limits NF‐κB‐myostatin‐driven atrophy.

SIRT3 supports mitochondrial function in most tissues. However, in bone, it enhances osteoclast resorption and accelerates skeletal aging under hormone‐ and age‐related stress. Evidence for other SIRTs in bone, cartilage, and muscle remains sparse.

Translation faces several hurdles. Most studies rely on germline or constitutive tissue‐specific mutations. These approaches may introduce developmental artifacts. They also fail to capture the timing and biomechanics of adult‐onset disease. SIRTs exhibit opposing cell type‐specific actions. For example, SIRT3 protects the heart but promotes resorption in osteoclasts. Systemic modulation therefore risks on‐target, off‐tissue toxicity. Moreover, the degree of cross‐talk between nuclear and mitochondrial SIRTs in the musculoskeletal niche remains poorly understood. Future studies require temporally controlled, niche‐specific mouse models. Human‐relevant systems, such as bone and cartilage organoids and engineered muscle, are also essential. These tools define how and when specific SIRTs, or SIRT axes, can be targeted to safely treat osteoporosis, OA, and sarcopenia.

### 3.8 SIRT Family Transgenic Mouse Models of Malignant Tumors

**FIGURE 5:**
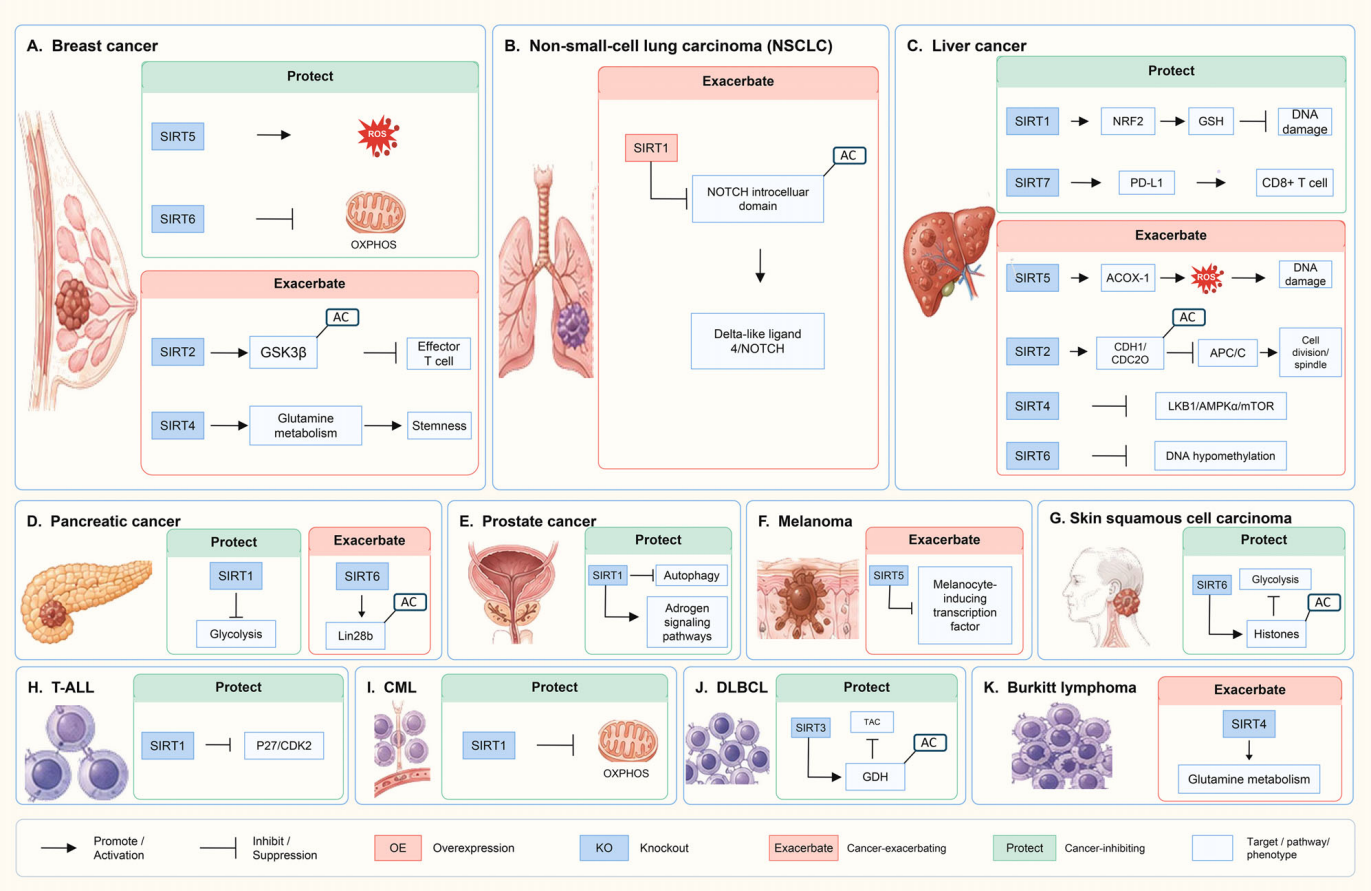
Sirtuins in tumors. This schematic summarizes the protective (green) and detrimental (red) roles of sirtuins across cancers on the basis of OE (pink) and KO (blue) mouse models. The arrows indicate activation/promotion, the blunt‐ended lines indicate inhibition/suppression, and “AC” denotes acetylation‐related regulation. (A) Sirtuins regulate ROS, oxidative phosphorylation, GSK3β acetylation, T‐cell function, glutamine metabolism, and stemness in breast cancer. (B) Non‐small‐ cell lung carcinoma: SIRT1 is linked to NOTCH signaling via NICD acetylation and DLL4. (C) Liver cancer: Sirtuins control NRF2/GSH‐mediated DNA repair, PD‐L1/CD8^+^ T‐cell crosstalk, ACOX1‐dependent ROS, APC/C‐mediated mitosis, LKB1/AMPK/mTOR signaling, and DNA hypomethylation. (D) Pancreatic cancer: SIRT1 and SIRT6 regulate glycolysis and Lin28b. (E) Prostate cancer: SIRT1 affects autophagy and androgen signaling. (F) Melanoma: SIRT5 is linked to transcription factor regulation. (G) Skin squamous cell carcinoma: SIRT6 regulates glycolysis and histone acetylation. (H–K) Hematologic malignancies: Sirtuins control p27/CDK2, oxidative phosphorylation GDH/TCA cycle, and glutamine metabolism. *Abbreviations*. ACOX1, acyl‐CoA oxidase 1; CDH1, epithelial cadherin; CDC20, cell division cycle protein 20; APC/C, anaphase‐promoting complex/cyclosome; GSK3β, glycogen synthase kinase 3β; mTOR, mammalian target of rapamycin; PD‐L1, programmed cell death 1 ligand 1.

Transgenic mouse models are essential for linking candidate cancer genes to tumor initiation, progression, and therapeutic response in vivo [377]. Across malignancies, SIRTs again act along shared axes—metabolism, redox balance, genome stability, autophagy, and immunity—but their net effect is highly context dependent. *Sirt1–7* models show that the same SIRT can either restrain or support tumor growth depending on tissue type, oncogenic drivers, and treatment conditions. An overview of these cancer phenotypes and pathways is provided in Figure 5 and Table S10, which serve as a guide for the detailed analysis.

#### 3.8.1 Breast Cancer

BC remains a leading cause of cancer‐related death in women [378]. In BC models, SIRT2, SIRT4, SIRT5, and SIRT6 regulate distinct metabolic and immune checkpoints with different effects on tumor progression.

SIRT2 sustains antitumor CD8^+^ T‐cell immunity. In C57BL/6 N mice, global *Sirt2* KO disrupts T‐cell differentiation. These mice accumulate naïve T cells but lose effector memory populations. This enzyme normally deacetylates glycogen synthase kinase‐3 beta to increase aerobic oxidation. This metabolic shift drives CD8^+^ T cells into effector‐memory subsets. Consequently, SIRT2 deficiency impairs antitumor immunity [294].

SIRT4 acts as a tumor suppressor in a luminal‐type BC model. In murine mammary tumor virus (MMTV)‐Neu mice, germline *Sirt4* loss increases mammary duct side‐branching and expands stem cell pools. These alterations promote tumorigenesis and metastasis. At the molecular level, SIRT4 suppresses BC stemness through a metabolic‒epigenetic cascade: it inhibits glutamine metabolism, downregulates SIRT1, and consequently reduces H4K16ac and BC susceptibility gene 1 expression [75].

SIRT5 supports tumor survival by facilitating ROS handling. In MMTV mammary tumor models, *Sirt5* KO reduces tumor volume and weight while increasing oxidative stress. This enzyme normally maintains mitochondrial redox homeostasis by facilitating the handling of ROS. This function is essential for BC growth [295].

SIRT6 plays a tumor‐promoting role in PyMT‐driven BC. In 129SvJ mice carrying the MMTV–PyMT transgene, global *Sirt6* deletion delays tumor onset and extends survival. SIRT6 normally promotes BC progression by increasing oxidative phosphorylation and cellular energetics. Its loss triggers metabolic stress that restricts tumor growth [82].

SIRT family members operate distinctly in BC. While SIRT2 empowers T cells against tumors, SIRT4 restricts cancer stemness. Moreover, SIRT5 and SIRT6 provide metabolic support to malignant cells. These contrasting functions underscore context‐dependent roles across SIRT isoforms.

#### 3.8.2 Non‐Small Cell Lung Cancer

Non‐small cell lung cancer accounts for ∼85% of lung cancer cases and most lung cancer‐related deaths [379]. The available Sirt models mainly involve SIRT1 in tumor angiogenesis.

In a Lewis lung carcinoma xenograft model, compared with WT control mice, Sirt1‐KI mice developed tumors approximately 15% larger. This accelerated growth originates in the endothelium. SIRT1 deacetylates the Notch1 intracellular domain (N1IC), which suppresses delta‐like ligand 4/Notch signaling. Consequently, ECs proliferate excessively and form dense vessel branches. The resulting hypervascularity improves tumor perfusion [296]. These data indicate that SIRT1 can support lung tumor growth by driving pathological angiogenesis.

#### 3.8.3 Hepatocellular Carcinoma and Pancreatic Ductal Adenocarcinoma

In hepatocellular carcinoma (HCC) and pancreatic ductal adenocarcinoma (PDAC), SIRT proteins govern glutamine metabolism, redox balance, and epigenetic programs.

Albumin–Cre‐mediated *Sirt1* deletion in the liver protects against dimethylnitrosamine‐induced HCC. Mutant animals exhibit a lower tumor burden and elevated glutathione metabolism. At the molecular level, SIRT1 loss activates NRF2 signaling. The resulting glutathione surge establishes a reductive milieu. This environment shields DNA from damage and restrains hepatocyte proliferation [297]. Together, these changes prevent HCC initiation.

In HCC, SIRT4 functions as a metabolic checkpoint. Germline *Sirt4* deletion in dimethylnitrosamine‐exposed mice accelerates tumorigenesis, and tumors grow larger and more numerous, with frequent lung metastases and poor survival. SIRT4 normally restrains mitochondrial glutamine metabolism. This suppression maintains high ADP/AMP ratios. Energy depletion activates AMPKα through liver kinase B1. This kinase cascade ultimately suppresses mTOR activity and halts tumor expansion [76].

SIRT7 enables immune evasion in HCC. In C57BL/6 mice with liver‐specific *Sirt7* deletion (albumin–Cre), orthotopic tumors grow more slowly. However, these tumors paradoxically display elevated programmed death‐ligand 1 expression and dense T‐cell infiltration. SIRT7 normally represses programmed death‐ligand 1 through MEF2D deacetylation. When SIRT7 is absent, programmed death‐ligand 1 expression increases. This attracts T cells and restricts tumor expansion [141].

Genomic instability links SIRT2 loss to sex‐specific tumorigenesis. Male *Sirt2*‐deficient mice (NIH Black Swiss or C57BL/6) develop HCC; females develop mammary tumors. The underlying defect involves anaphase‑promoting complex/cyclosome coactivators. SIRT2 deacetylates cadherin 1 and cell division cycle 20 to maintain mitotic fidelity. Loss of this regulation triggers centrosome amplification and aneuploidy. These abnormalities drive malignant transformation [71]. Global *Sirt5* KO in C57BL/6J mice increases peroxisomal H_2_O_2_ and oxidative DNA damage. This defect stems from the loss of ACOX1 desuccinylation. Consequently, SIRT5 suppresses liver cancer by restraining peroxisomal oxidative stress [78]. Global *Sirt6* KO mice (129‐*Sirt6*^tm1Fwa/J) develop severe hypoglycemia and hepatic steatosis. Their livers exhibit genome‐wide hypomethylation and other oncogenic changes. These phenotypes establish SIRT6 as a tumor suppressor essential for hepatic metabolic and epigenetic homeostasis [80].

In PDAC, SIRT1 and SIRT6 have opposing effects on tumor progression. In B6;129 Pdx1–Cre;Kras^G12D mice, pancreas‐specific *Sirt1* KO reduces proliferation and glycolytic gene expression in early pancreatic intraepithelial neoplasia lesions, indicating that SIRT1 normally supports glycolysis and growth in early pancreatic neoplasia [298]. Pancreas‐specific *Sirt6* KO in 129 Sv/C57BL/6 p48–Cre;Kras^G12D;p53 background mice promotes PDAC development and metastasis. SIRT6 suppresses malignancy through epigenetic regulation. It deacetylates histones at the Lin28b promoter, thereby repressing Lin28b and maintaining let‐7 microRNA levels. Loss of this control derepresses Lin28b and drives metastatic disease [79].

These liver and pancreas models reveal distinct functions of SIRT in cancer suppression. SIRT4 and SIRT6 inhibit HCC and PDAC. SIRT1 suppresses HCC but can support early pancreatic neoplasia, indicating stage‐dependent effects. In contrast, under specific conditions, SIRT7, SIRT2, and SIRT5 exert context‐dependent effects on proliferation and immune evasion.

#### 3.8.4 Other Solid Malignancies

In prostate cancer, melanoma and skin squamous cell carcinoma, SIRTs play tissue‐specific tumor‐suppressive or tumor‐promoting roles.

Global Sirt1ΔE4 mice develop a reduced prostate size but display prostatic intraepithelial neoplasia. These lesions show increased cellularity and nuclear atypia and can progress to invasive cancer. SIRT1 normally protects against prostate cancer by maintaining autophagic flux. This enzyme also limits ROS accumulation and restrains androgen‐driven proliferation [67]. Together, these functions suppress tumorigenesis through the autophagy‒oxidative stress axis.

SIRT5 is required for melanoma maintenance. In a Braf/Pten/Tyr‐driven model, global *Sirt5* KO reduced tumor burden. These mice remain viable and show only mild cardiac phenotypes. SIRT5 sustains melanoma cell survival through epigenetic control. It maintains histone acetylation and methylation patterns [299]. These markers preserve the expression of lineage‐critical oncogenes, including microphthalmia‐associated transcription factor and c‐MYC.

K14–Cre‐mediated *Sirt6* deletion accelerates 7,12‐dimethylbenz[a]anthracene/12‐O‐tetradecanoylphorbol‐13‐acetate‐induced skin carcinogenesis in C57BL/6 mice. Mutant animals exhibit earlier tumor onset and greater disease burden. SIRT6 suppresses malignancy through epigenetic control. It deacetylates histones at glycolytic promoters, restricting both glycolysis and antioxidant responses in tumor‐propagating cells. Loss of this regulation enhances glycolytic flux and promotes progression [81].

#### 3.8.5 Hematologic Malignancies

SIRT family members control energy metabolism in hematological malignancies. They modulate glutamine utilization, TCA cycle activity, and oxidative phosphorylation across malignant and stem cell populations.

In T‐ALL, SIRT1 promotes disease progression. C57BL/6J mice with Notch1‐driven T‐ALL exhibit attenuated disease and prolonged survival following *Sirt1* deletion in hematopoietic stem and progenitor cells (Mx1–Cre). The underlying mechanism involves cyclin‐dependent kinase 2 deacetylation. This posttranslational modification destabilizes p27 through phosphorylation and degradation. Loss of this checkpoint drives cell cycle progression in malignant T cells [300].

In chronic myeloid leukemia models, SIRT1 maintains leukemia stem cells and drives drug resistance. Mx1–Cre‐mediated *Sirt1* deletion in hematopoietic stem and progenitor cells attenuates chronic myeloid leukemia development and extends survival. This protection stems from defective mitochondrial oxidative phosphorylation. Loss of SIRT1 also impairs PGC‐1α‐mediated resistance to tyrosine kinase inhibitors [301].

In a VavP–Bcl2 diffuse large B‐cell lymphoma model, global *Sirt3* KO C57BL/6J mice exhibited reduced lymphoma development and improved survival. The formation of the germinal center remains largely intact. SIRT3 functions as a metabolic oncogene. It deacetylates glutamate dehydrogenase to increase TCA cycle flux and mitochondrial metabolism. These metabolic changes support diffuse large B‐cell lymphoma proliferation and survival [302].

In Eμ‐Myc‐driven B‐cell lymphoma, global *Sirt4* KO accelerates disease onset and decreases survival. SIRT4 suppresses Myc‐induced lymphoma by blocking mitochondrial glutamine metabolism. Notably, this inhibition operates independently of Myc itself. Thus, SIRT4 serves as a metabolic brake on B‐cell proliferation [77].

#### 3.8.6 Research Limitations and Future Perspectives

SIRT proteins serve as context‐dependent metabolic and epigenetic regulators. They are not uniformly tumor suppressive or oncogenic. Their functions shift with the tissue environment and metabolic state. SIRT1, SIRT3, SIRT4, and SIRT6 often restrain tumor growth. They limit glycolysis, reduce glutamine flux, prevent ROS damage, or block cell cycle progression. In contrast, SIRT2, SIRT5, and SIRT7 can promote tumor survival. They support oxidative phosphorylation, detoxify ROS, modulate T‐cell differentiation, or enable immune evasion. These opposing roles depend entirely on the cellular context.

Transgenic mouse models provide strong mechanistic support for targeting SIRTs in cancer. However, translation faces major hurdles. Most studies rely on germline mutations in limited strains. These models may not capture human tumor heterogeneity or compensatory networks. Cell type‐specific functions create additional risks. SIRT1 suppresses prostate cancer but promotes early pancreatic lesions. SIRT6 inhibits liver and pancreatic tumors but supports BC metabolism. Such variation indicates that systemic modulation can cause on‐target, off‐tissue toxicity.

Pharmacological tools also remain nonselective. The tissue distribution of mitochondrial versus nuclear SIRT targeting is unclear. Definitions of optimal NAD^+^‐precursor dosing and long‐term safety are needed. Future work must combine multiallelic, conditional, and time‐controlled models with patient‐derived organoids, xenografts, and single‐cell multiomics. Only then can we resolve functional redundancies and identify tractable targets. This approach provides mechanistic insights into rational strategies for cancer prevention and therapy.

### 3.9 SIRT Family Transgenic Mouse Models of Immune Diseases

Unlike most parenchymal organs, the immune system is characterized by rapid cell turnover, metabolic reprogramming, and phase‑specific responses to antigen, infection, and tissue damage. In this context, *Sirt1–7* does not simply buffer against stress. They act as switches that bias lineage choice, tune inflammatory amplitude, and shape the transition from acute to chronic responses. Transgenic mouse models targeting *Sirt1–7* in hematopoietic stem cells, T and B lymphocytes, DCs, macrophages, mast cells, and innate lymphoid cells have been crucial for evaluating SIRT function. These models cover autoimmunity, graft‑versus‑host disease (GVHD), allergy, and host defense. These immune phenotypes and pathways are summarized in Table S11, and a detailed discussion is provided below.

#### 3.9.1 Autoimmune Diseases

In experimental autoimmune encephalomyelitis (EAE), a mouse model of multiple sclerosis, SIRT1 and SIRT2 predominantly promote pathogenic inflammation, whereas SIRT6 restrains myeloid‐driven responses.

In C57BL/6 mice, DC‐specific *Sirt1* deletion (CD11c–Cre) protects against MOG_35_‐_55_‐induced EAE. Mutant animals show reduced Th17 differentiation and diminished inflammation. SIRT1 normally deacetylates interferon regulatory factor 1 to suppress IL‐27 expression. In the absence of SIRT1, the level of IL‐27 is restored. This cytokine surge limits Th17‐driven pathology [303]. T‐cell‐specific *Sirt1* deletion (RORγt–Cre) also attenuated Th17 differentiation and ameliorated EAE. This enzyme promotes Th17 fate through the deacetylation and activation of RORγt. Loss of this regulation protects against autoimmune pathology [305]. Astrocyte‐specific *Sirt1* KO (GFAP–Cre; C57BL/6) increases EAE severity. These mice exhibit reduced central nervous system inflammation and diminished demyelination. This protection involves NRF2 activation. By increasing the expression of this transcription factor, *the* loss of *Sirt1* expands the populations of IL‐10‐producing macrophages and microglia. Anti‐inflammatory and antioxidant gene programs are consequently induced [304].

SIRT2 exerts a more uniform proinflammatory effect on adaptive immunity. In C57BL/6J *Sirt2*KO mice, CD4^+^ T cells exhibit reduced Th17 differentiation, increased IL‐2 production, and protection against adoptive transfer EAE and lupus‐like disease. SIRT2 normally drives Th17 commitment while restraining IL‐2. It achieves this by deacetylating p70S6K, c‐Jun, and histones at the Il2 promoter. These modifications enhance autoimmune responses [306].

In rheumatoid arthritis, myeloid‐specific *Sirt6* deletion (LysM–Cre; B6/129) exacerbates collagen‐induced arthritis. Mutant mice display heightened macrophage infiltration and severe joint destruction. SIRT6 normally restrains macrophage activation and migration by deacetylating FOXO1. This epigenetic control suppresses synovial inflammation [134].

#### 3.9.2 Graft Versus Host Disease

In GVHD after allogeneic hematopoietic stem‐cell transplantation, donor T‐cell SIRT1 and SIRT3 promote pathogenicity through distinct mechanisms.

In C57BL/6 acute and chronic GVHD models, CD4–Cre‐mediated *Sirt1* deletion in donor T cells attenuated disease severity and extended survival. SIRT1 normally drives T‐cell activation and inflammatory cytokine production through p53 deacetylation. In the absence of this enzyme, p53 acetylation increases. This stabilizes induced regulatory T cells and curbs pathogenic effector responses [307].

SIRT3 similarly facilitates GVHD by regulating oxidative stress in donor T cells. In allogeneic bone marrow transplantation models, compared with those receiving WT T cells, those receiving Sirt3‐deficient T cells markedly improved survival. This protection is associated with reduced ROS production, which is consistent with a role for SIRT3 in promoting ROS accumulation and aggravating GVHD pathology [308].

The findings establish a dual control mechanism. SIRT1 ensures iTreg stability via p53 deacetylation, whereas SIRT3 governs oxidative stress in activated donor T cells. This suggests a therapeutic strategy: transient inhibition of these SIRTs in donor cells might suppress GVHD while preserving antileukemic activity.

#### 3.9.3 Allergic and Inflammatory Diseases

Keratinocyte SIRT1 protects against atopic dermatitis by preserving barrier function. In K14–Cre;*Sirt1*^flox/flox C57BL/6 mice, epidermal *Sirt1* loss produces spontaneous AD‐like skin lesions and sensitizes animals to epicutaneous ovalbumin. This enzyme promotes filaggrin expression through aryl hydrocarbon receptor (AhR)/Akt‐mediated deacetylation. This pathway reinforces barrier integrity and prevents allergen‐driven inflammation [118].

SIRT6 governs allergic responses by restraining mast cell activation. Myeloid‐specific *Sirt6* KO (LysM–Cre; C57BL/6) enhances both systemic and cutaneous IgE‐mediated passive anaphylaxis. Mast‐cell degranulation increases correspondingly. This enzyme suppresses FcepsilonRI signaling through protein tyrosine phosphatase receptor type C transcriptional repression. This mechanism dampens mast‐cell activation and protects against allergies [135].

These models identify SIRT1 as a key regulator of the skin AHR–barrier axis and SIRT6 as a brake on mast‐cell FcepsilonRI‐degranulation, together maintaining tolerance at the barrier and limiting systemic effector responses.

#### 3.9.4 Infection and Sepsis

In infection and sepsis, SIRTs play context‐dependent roles.

Global *Sirt2* KO C57BL/6 mice clear chronic *S. aureus* infection more efficiently. These animals exhibit enhanced macrophage phagocytosis and improved survival. SIRT2 normally limits bacterial uptake by deacetylating α‐tubulin in macrophages. This modification suppresses glycolytic metabolism and dampens anti‐infective responses [309]. During *L. monocytogenes* infection, SIRT2 is recruited to host chromatin via PI3K/Akt signaling. It deacetylates H3K18 and silences immune‐related genes. *Sirt2*‐deficient mice harbor lower splenic bacterial loads and display reduced susceptibility [310].

SIRT3 and SIRT1 defend against mycobacterial infection. In C57BL/6 mice with mycobacteria, global *Sirt3* KO increases lung bacterial burden and mortality. These animals also develop severe pathological inflammation with neutrophil infiltration. Mitochondrial dysfunction and ROS accumulation accompany this phenotype. SIRT3 normally maintains PPARα and TFEB expression through deacetylation. This epigenetic control coordinates mitochondrial homeostasis with autophagy activation. Consequently, antimicrobial autophagosomes form efficiently. Excessive inflammation is simultaneously restrained [312]. In sepsis, SIRT3 cooperates with SIRT1 and RelB to form a signaling axis. This pathway reprograms energy metabolism in myeloid cells. Notably, global *Sirt3* KO did not significantly alter survival in cecal ligation and puncture models, suggesting that compensatory mechanisms or context‐dependent requirements [311] exist; these pathways highlight the role of SIRT3 in immunometabolic adaptation.

DoubleKO models further emphasize cooperative or antagonistic SIRT interactions. Global *Sirt2*/*Sirt3* double KO C57BL/6 mice exhibit expansion of peritoneal B‐1a cells and protection from LPS‐induced endotoxemia, with altered macrophage metabolism and cytokine production [69]. Similarly, global *Sirt3*/*Sirt5* double KO mice display enhanced inflammatory responses but improved resistance to acute Listeria infection, with reduced morbidity and bacterial burden [133]. Mechanistically, *Sirt3*/*Sirt5* deficiency leads to increased mROS production in macrophages and neutrophils, as well as elevated proinflammatory cytokine secretion, which collectively contributes to enhanced bacterial clearance. In sepsis models, *Sirt5* deficiency attenuates cytokine production and immune responses during both the hyperinflammatory phase and the hypoinflammatory phase, whereas it promotes antibacterial defenses under inflammatory conditions [132].

Infectious disease models reveal opposing SIRT functions. SIRT2 restrains innate immunity and can be hijacked by bacteria. In contrast, SIRT3 and SIRT5 support host defense, although their effects can be context dependent. Their combined deficiency enhances inflammation and bacterial clearance, but the timing, tissue, and disease phase of SIRT modulation in sepsis must be considered.

#### 3.9.5 Research Limitations and Future Perspectives

Transgenic mouse models have shown that several isoforms, especially SIRT1, SIRT2, SIRT3, SIRT5, and SIRT6, are true rate‐limiting regulators of immunity in vivo. They can shift the Th17/Treg balance, influence tolerance, modulate mast‑cell activation, shape macrophage‑driven inflammation, and alter anti‑infective defense. Moreover, they also reveal strong context dependence. The same SIRT can have opposite effects in different cell types. For example, SIRT1 acts differently in DCs and T cells, and SIRT2 behaves differently during infection and sterile inflammation. Thus, immune phenotypes reflect the behavior of a network rather than a single linear pathway.

Despite these advances, the current evidence from mouse models is still incomplete. Most studies use single‑gene, germline, or broad lineage‑specific KOs. They are usually carried out in one or two inbred strains and in acute disease models. This design makes it difficult to separate developmental from adult functions and to model chronic or relapsing disease and aging. Catalytic–dead or point–mutant alleles are rare. Multiallelic combinations are uncommon. Many important cell types, such as barrier and stromal cells and rare innate subsets, remain underexplored. Integration of these models is essential.

### 3.10 Summary

Taken together, these findings provide the most comprehensive transgenic evidence for SIRT1, SIRT3, and SIRT6. For these isoforms, multiple global, tissue‑specific, and sometimes inducible models exist, often in more than one strain background and across different ages and stress conditions. Their roles as central regulators of metabolism, mitochondrial function, genome stability, stress responses, and inflammation are therefore supported by broad, convergent in vivo data. SIRT7 is moderately well characterized, with clear links to lipid metabolism, ER/proteotoxic stress, and cancer, although fewer organs and disease contexts have been systematically explored.

In contrast, models for SIRT2, SIRT4, and SIRT5 are less comprehensive and are often restricted to single disease areas or cell types. SIRT2 has several global KOs and a few conditional alleles, but its functions have been investigated in a narrow set of neurodegenerative, inflammatory, and tumor models. SIRT4 and SIRT5 are represented mainly by global KOs, with relatively few tissue‑specific or inducible lines and limited exploration outside selected metabolic and cancer contexts. As a result, our confidence in isoform–disease relationships is highest for SIRT1, SIRT3, and SIRT6; moderate for SIRT7; and still provisional for SIRT2, SIRT4, and SIRT5, highlighting where future genetic work is most needed.

### 3.11 From Mice to Humans: Consistencies, Contradictions, and Unresolved Issues

*Sirt1–7* transgenic mice are indispensable for clarifying how individual SIRTs function in vivo. Many outcomes are consistent with human data. For SIRT1, the evidence is most abundant. Its polymorphisms are associated with high risks of cardiovascular, metabolic, and autoimmune diseases across African, European, and Asian populations [380]. With respect to SIRT6, direct links to the human lifespan have drawn particular attention. Its polymorphisms, such as the stop‐gained variant rs117385980 (T allele), are associated with a shorter lifespan [381].

However, the pathological phenotypes and physiological impacts of SIRTs can differ substantially between mice and humans. For example, hepatic SIRT5 OE improves glucose and lipid metabolism in mice [51]. In contrast, the human SIRT5 rs12216101 T>G gain‐of‐function variant is directly associated with an increased risk of significant fibrosis and nonalcoholic steatohepatitis [382]. These differences may stem from species‐specific genetic backgrounds, distinct gene functions, or compensatory mechanisms.

Beyond differences in gene function, several fundamental issues limit the translational value of mouse models. For instance, mice and humans differ markedly in immune system architecture, gene regulation, tissue organization, and metabolic rate. Moreover, the short lifespan of mice may be insufficient for certain chronic pathological processes to fully manifest. Another limitation is that most available data come from single pathways in single tissues or cells. As a result, we have a limited understanding of intertissue cross‐talk and how different SIRT isoforms coordinate or antagonize each other at the whole‐organism level. Whether human gene functions, pathway interactions, and genetic epistasis mirror those in mice remains unknown. Large‐scale, well‐designed human cohort studies with longitudinal follow‐up are still needed to validate findings from mouse models. The translational gap remains substantial.

## 4 Translational Medicine: SIRTs as Therapeutic Targets

Genetic manipulation of *Sirt1–7* in mice clearly revealed that individual isoforms can be rate‐limiting in metabolism, stress responses, inflammation, and tumorigenesis. Translation, however, depends on whether these axes can be engaged in a drug‐like manner in disease models and in patients. Over the past two decades, small‐molecule activators and inhibitors of SIRT1–SIRT7 have moved from tool compounds to agents with defined cellular and in vivo activity. Table S12 summarizes representative modulators from this work. Together with the mouse models discussed in Sections 2 and 3, these molecules begin to outline which SIRT‐centered pathways are most tractable for clinical translation and in which directions (activation vs. inhibition).

### 4.1 Overview of Pharmacological Modulators: Panactivators/Inhibitors Versus Isoform‐Selective Agents

The pharmacology of SIRTs is shaped by two largely sequential waves. The first wave focused on SIRT1 and was driven by the discovery of resveratrol and synthetic STACs. Resveratrol allosterically enhances SIRT1 activity on selected hydrophobic substrates and, in human studies, improves insulin sensitivity, lipid profiles, and endothelial function in obese or cardiometabolic cohorts, although the effects are modest and heterogeneous [383, 384]. SRT2104, a more potent STAC that binds an N‐terminal SIRT1 activation domain, improves glucose homeostasis and arterial compliance in obese rodents and in smokers and patients with Type 2 diabetes [385]. These compounds provide proof‐of‐principle that SIRT1 can be pharmacologically activated in humans, but they also highlight a central limitation of this first wave: broad engagement of SIRT1, largely independent of tissue context and substrate specificity, does not automatically translate into robust disease modification.

A second wave results in isoform‐resolved modulation. Within the SIRT1 arm, small molecules such as MC2562 and ISIDE11 exemplify more targeted activation. MC2562, a 1,4‐dihydropyridine‐based activator, enhances SIRT1‐dependent deacetylation and nitric oxide‐linked endothelial responses and accelerates wound healing in mice [386]. ISIDE11 shows selectivity for class I/II HDACs and rescues vascular dysfunction and thrombosis in mice [387]. At the mitochondrial level, honokiol directly binds and activates SIRT3 while upregulating its expression, improving cognition in AD mice, ameliorating colitis and attenuating diabetes‐related bone loss [388]. Amiodarone‐derived activators such as ADTL–SA1215 (3c) bind the SIRT3 acyl channel, promote SOD2 deacetylation and suppress triple‐negative BC xenografts, although at the cost of dose‐limiting lung toxicity [389].

With respect to nuclear SIRTs, activators of SIRT6, such as UBCS039 and the MDL‐800/811 series, have opened a further axis. These agents bind distal pockets of the acyl channel and can increase H3K9/H3K56 deacetylation [390, 391, 392, 393]. Collectively, these data show that it is now feasible to upregulate SIRT1, SIRT3, or SIRT6 activity in a mechanistically informed, isoform‐biased manner in vivo.

In parallel, inhibition strategies have matured. Selisistat (EX‐527) remains the best‐characterized SIRT1‐preferring inhibitor: it traps a SIRT1–ADP‐ribose intermediate in the catalytic pocket, is safe according to Phase I studies, shows short‐term clinical and neuropsychiatric benefits in early HD, and potentiates chemotherapy in lung and esophageal cancer xenografts [394, 395, 396]. With respect to SIRT2, reversible inhibitors (AGK2), “selectivity pocket” binders (SirReal2) and mechanism‐based thiomyristoyl‐lysine analogs (TM, AF8) now connect biochemical SIRT2 blockade to α‐tubulin hyperacetylation, protection from colitis, and enhanced antitumor immunity and c‐Myc destabilization in c‐Myc‐driven breast and colorectal cancers [397, 398, 399, 400, 401].

Other SIRTs have only recently become pharmacologically tractable. MC3482 and its derivatives selectively inhibit SIRT5 desuccinylase activity and increase global succinylation, stimulating brown‐fat‐like programs in vitro [402]. More potent mechanism‐based SIRT5 inhibitors, such as Et‐40b/Et‐40c (NRD167/NRD139) and DK1‐04, form stalled thioimidate intermediates with ADP‐ribose and selectively kill SIRT5‐dependent acute myeloid leukemia (AML) and BC cells in xenografts [295, 403]. With respect to SIRT6, small‐molecule inhibitors (OSS_128167 and compound 8a) and the PROTAC degrader SZU‐B6 have been shown to dampen airway inflammation in patients with asthma, increase chemosensitivity in patients with pancreatic cancer, and suppress liver cancer xenograft growth [404, 405, 406]. The first small‐molecule SIRT7 inhibitor, ID:97491, increases p53 acetylation and phosphorylation and restricts uterine sarcoma xenografts [407].

While these agents validate *Sirt1–7* targets in vivo, they lack clinical maturity and require significant optimization to become viable therapeutic drugs.

### 4.2 Promising Therapeutic Strategies in Preclinical Models

SIRT1 activation is the most extensively investigated SIRT‐based strategy. Resveratrol‐class STACs and more selective agents, such as MC2562 and ISIDE11, improve insulin sensitivity, lipid handling, and vascular function in obese and diabetic patients [384, 385, 386, 387]. These effects mirror the protective effects of moderate *Sirt1* OE and the inverse of liver‐, endothelial‐, and podocyte‐specific *Sirt1* deletion. However, human trials have shown only partial and often short‐lived benefits, indicating that SIRT1 is not a single dominant switch in complex metabolic syndromes and that further optimization is needed.

SIRT3‐ and SIRT6‐directed activators have complementary indications. Honokiol and ADTL‐SA1215 reproduce key features of *Sirt3* OE‐improved mitochondrial redox control, SOD2 deacetylation and mitophagy in AD, colitis, and TNBC models [388, 389]. SIRT6 activators such as UBCS039 and MDL‐800/811 improve hepatic and vascular homeostasis and suppress tumor growth, which is consistent with the cardiometabolic protection and lifespan extension observed in *Sirt6*OE mice [390, 391, 392, 393].

In contrast, the inhibition or degradation of SIRT2, SIRT5, SIRT6, and SIRT7 has been explored mainly in tumor and inflammatory conditions. SIRT2 inhibitors reproduce aspects of the *Sirt2*‐null phenotype, including protection in colitis, enhanced antitumor immunity, and efficacy in c‐Myc‐driven tumors [397, 398, 399, 400, 401]. Mechanism‐based SIRT5 inhibitors selectively kill SIRT5‐dependent AML and BC cells, suggesting that an oncogene‐addiction phenotype remains poorly studied clinically [295, 403]. With respect to SIRT6 and SIRT7, small‐molecule inhibitors and degraders that target defined chromatin marks (H3K9/H3K56 and H3K18) and p53‐linked pathways provide initial evidence that these nuclear SIRTs can be manipulated in cancer and immune disease [404, 405, 406, 407].

Notably, there are also clear absences. Pharmacologically credible SIRT4 activators are essentially lacking, despite strong genetic evidence for the role of SIRT4 in amino acid metabolism, insulin secretion, and SAP. With respect to SIRT3, the bulk of its activator work is still preclinical and, outside of honokiol, has not moved toward human studies. For all mitochondrial SIRTs, including SIRT5, systematic evaluation of nonmalignant indications such as cardiomyopathy, neurodegeneration or AKI remains lacking. These gaps identify obvious priorities for the next generation of SIRT‐directed therapeutics.

### 4.3 Current Clinical Trial Landscape and Outcomes

Our previous review summarized clinical studies targeting SIRT pathways in detail [18]. Here, we update that overview with more recent trials and focus on patterns that emerge across indications. The full list of trials and SIRT‐related readouts is provided in Table S13.

Most interventional studies still center on SIRT1. Many use indirect modulators such as polyphenols, vitamins, dietary change, exercise, metformin, or NAD^+^ precursors rather than isoform‐selective drugs. In Type 2 diabetes and NAFLD, several small RCTs reported increased circulating or peripheral blood mononuclear cell SIRT1 after treatment with turmeric, green cardamom, ellagic acid, vitamin D, green coffee, pomegranate juice, and sildenafil [408, 409, 410, 411, 412, 413, 414]. These changes are often accompanied by modest improvements in insulin resistance, liver enzymes, or inflammatory markers. Resveratrol has heterogeneous effects. In NAFLD, 600 mg/day did not significantly alter SIRT1 expression [415]. In T2D, 500 mg/day increased SIRT1 and reduced H3K56ac, which is consistent with SIRT1‐dependent histone deacetylation [416]. Metformin in individuals with prediabetes also increases SIRT1 expression and the accessibility of the SIRT1 promoter via AMPK [417].

Cardiometabolic and aging studies report similar trends. Circuit training in healthy elderly participants increases blood SIRT1 and SIRT3 activity [418]. Diets enriched in monounsaturated fat increase SIRT1 mRNA expression in metabolic syndrome [409]. In coronary artery disease, n‐3 fatty acids plus vitamin E upregulate SIRT1 and PGC‐1α [419], whereas crocetin and crocin increase SIRT1 and AMPK expression and improve atherogenic and inflammatory profiles [420, 421]. A trial of high‐dose resveratrol in postmenopausal women with coronary artery disease (NCT05808387) aimed to measure serum concentrations and gene expression of SIRT1 and SIRT3 after 90 days and provided further information on dose‒response relationships in this population. The SIRT1 activator SRT2104 improves arterial compliance and blood pressure in healthy smokers and patients with T2D [385], providing pharmacological proof of concept for vascular SIRT1 activation in humans. Ongoing trials with nicotinamide riboside and MIB‐626 in vascular dysfunction, perioperative AKI and AD aim to test whether NAD^+^ precursors engage the SIRT–NAD axis in vivo (e.g., NCT07073352, NCT06521307, and NCT05040321).

In autoimmune and inflammatory disease, yoga‐based programs and selenomethionine increase the expression of SIRT1 and mitochondrial biogenesis markers in rheumatoid arthritis and ulcerative colitis [422, 423, 424], whereas SRT2104 does not clearly improve clinical efficacy in mild–moderate ulcerative colitis despite its acceptable safety [425]. In oncology, a Phase I study in which vorinostat was combined with niacinamide in lymphoma demonstrated increased acetylation of BCL6 and p53, which is consistent with inhibition in vivo [426].

Overall, human data confirm that SIRT1 (and to a lesser extent SIRT3) can be modulated in patients and that such modulation tracks with favorable changes in metabolic, vascular, or inflammatory biomarkers. However, trials remain small, short, and biomarker‐driven, use pleiotropic interventions, and almost never test true isoform‐selective agents or hard clinical endpoints. This gap contrasts sharply with the robust causal evidence from Sirt1–7 mouse models and highlights the need for next‐generation, isoform‐specific modulators, and adequately powered, mechanism‐anchored RCTs.

### 4.4 Challenges and Future Directions: Bioavailability, Tissue Specificity, and Context‐Dependent Effects

The compounds in Table S12 show that SIRTs are pharmacologically tractable and that isoform‐specific modulation can alter disease‐relevant pathways in mice and, to a limited extent, in humans. Moreover, they highlight that SIRT‐based therapies are not yet clinically mature.

Isoform selectivity is improved but still incomplete. STACs, tenovin‐class inhibitors and older probes affect multiple SIRTs and other NAD^+^ users, and even “selective” agents such as EX‐527, AGK2, or ID:97491 show assay‐ and substrate‐dependent cross‐reactivity [396, 407]. Mechanism‐based covalent inhibitors of SIRT2 and SIRT5 further depend on prodrug chemistry, which can introduce new off‐targets or change isoform profiles [295, 403, 404]. Robust selectivity profiling and target‐engagement assays are therefore essential.

Strong context‐dependence is equally clear. The same SIRT can be protective or harmful depending on tissue and disease: SIRT6 activation benefits cardiometabolic disease, whereas SIRT6 inhibition or degradation is advantageous in asthma and some cancers [404, 405, 406]. SIRT2 inhibition protects against colitis and neuroinflammation, yet Sirt2‐null mice accumulate late‐life tumors; SIRT5 blockade kills SIRT5‐addicted AML but may exacerbate oxidative or nitrogen stress in other conditions. These patterns argue against global “activation” or “paninhibition” and favor isoform‐, tissue‐, and disease‐matched modulation.

Drug‐like properties and clinical development remain underpowered. Many potent compounds have poor solubility, short half‐lives or limited tissue penetration; PROTACs such as SZU‐B6 and peptide‐derived SIRT5 inhibitors illustrate the trade‐off between biochemical potency and pharmacokinetics [295, 404]. Beyond indirect SIRT1 modulation and limited experience with EX‐527, highly selective SIRT2–SIRT7 modulators have not yet been investigated in large, mechanism‐anchored trials.

Thus, the agents in Table S12 are best regarded as translational probes rather than finished therapeutics. They validate the draggability of SIRT axes defined by *Sirt1–7* mouse models and indicate promising disease settings, but they also delineate the main obstacles. The key challenge is now to move from this exploratory phase to a disciplined program of isoform specific, context‐aware SIRT modulation in human disease.

### 4.5 The Gap Between Animal Models and Translational Medicine

Despite decades of intensive research using SIRT transgenic mouse models, the successful translation of SIRT‐targeted therapies remains limited. Several factors contribute to this gap. First, mouse models and human diseases differ markedly in genetics and physiology. Mouse experiments are typically conducted in young, inbred, standard diet‐fed mice, whereas patients are older, outbred, genetically diverse, and exposed to complex environmental influences. Mouse disease models are often driven by single factors and develop rapidly, whereas human diseases arise from polygenic interactions, progress slowly over decades, and are shaped by comorbidities such as obesity, hypertension, and diabetes. Moreover, genetic KO or OE mimics lifelong, complete loss or gain of SIRT function. Clinical drugs, however, are given intermittently, achieve only partial target engagement, and often have off‐target effects. As a result, phenotypes that appear promising in mice may not be achievable in humans, where small‐molecule activators may fail to reach sufficient exposure in the relevant tissues.

Clinical translation is further limited by the lack of robust biomarkers. Trials need biomarkers to select patients and to monitor target engagement. However, for most SIRT family members, validated, noninvasive biomarkers are lacking. Without biomarker‐based stratification, even efficacious drugs may fail in unselected trial populations. Publication bias exacerbates this problem. Positive findings in mouse models are more likely to be published, whereas negative or null results often remain unreported. This distorts perceptions of efficacy and encouraging overly optimistic translational attempts that ultimately fail in human trials.

To narrow this gap, more rigorous preclinical study designs are needed, including randomization, blinding, and formal sample size justification. Greater use of aged, comorbid, or humanized mouse models is warranted. Early validation in human ex vivo systems such as organoids and patient‐derived cells is also needed. Collaborative efforts to standardize outcome measures and to share negative data are also essential. Only through such measures can SIRT‐based therapies be reliably advanced from the bench to the bedside.

## 5 Conclusion and Future Perspectives

The results of the transgenic mouse studies reviewed here show that whole‑body and tissue‑specific *Sirt1–7* manipulations are powerful tools for establishing causality. Global and conditional KOs reveal where the loss of a given isoform is sufficient to trigger or accelerate disease. OE models, in turn, show where increased activity can prevent or delay pathology. Within this framework, the most promising drug targets are SIRTs whose deletion promotes disease, while moderate OE or activation is protective. SIRT1, SIRT3, and SIRT6 meet these criteria in many systems. The loss of these isoforms worsens metabolic, cardiovascular, neurodegenerative, renal, and inflammatory phenotypes. Their OE often improves insulin sensitivity, preserves mitochondrial function, reduces inflammation, and extends healthspan. SIRT1, SIRT3, and SIRT6 activators can mimic key protective effects observed in transgenic mice. This genetic–pharmacologic concordance strongly supports further drug development.

For other isoforms, the reverse logic applies. When KO is beneficial and OE is harmful, inhibition or degradation is more attractive. SIRT2, SIRT5, and SIRT7 provide such examples in specific contexts. *Sirt*2 deletion can ameliorate colitis, allergic airway disease, some neurodegenerative models, and certain c‑Myc‑driven tumors. *Sirt5* loss sensitizes cells to SIRT5‐dependent cancers. *Sirt*7 deficiency restrains liver and hematologic malignancies in selected settings. Corresponding inhibitors and degraders now exist. However, transgenic data also show that these isoforms can be protective in other tissues or phases of disease. This argues for context‑specific, rather than global, inhibition strategies.

Transgenic mouse models have therefore been indispensable for SIRT biology. They define causal gene functions, pinpoint disease‑relevant cell types, validate targets in vivo, and allow for the testing of delivery and combination strategies. Moreover, they have important limitations. Mouse SIRT networks and disease trajectories are not identical to those in humans. Disease models are induced rather than spontaneous and do not fully recapitulate the natural course. Small‑molecule modulators in mice are usually given systemically and lack the cell‑type precision of genetic tools.

Taken together, these points suggest clear priorities for the next generation of models, including for SIRT1, SIRT3, and SIRT6. First, models should favor inducible, adult‑onset, cell‑type‑specific OE at defined expression levels. Second, they should include catalytic‑dead and separation‑of‑function knock‑ins that dissect individual domains or substrates. Third, combinatorial or double‐allele strategies in disease‐relevant genetic backgrounds are important. In parallel, tissue‑targeted delivery systems, systematic mapping of SIRT‒SIRT interactions, exploration in new disease areas and robust isoform‑specific biomarkers are needed. Together, these tools are essential for converting current qualitative genetic insights into quantitative guidance for SIRT‐directed drug discovery and clinical translation.

## Author Contributions

J‐YW, F‐LJ, Q‐JW, H‐HC, and T‐TG contributed to the study design. J‐YW, F‐LJ, F‐YZ, and H‐HC collected the data. J‐YW, F‐LJ, F‐YZ, D‐HH, X‐YL, SG, HY, Q‐JW, H‐HC, and T‐TG wrote the first draft of the manuscript and edited the manuscript. All the authors read and approved the final manuscript. J‐YW, F‐LJ, and F‐YZ contributed equally to this work.

## Ethics Statement

The authors have nothing to report.

## Conflicts of Interest

The authors declare no conflicts of interest.

## Supporting information




**Supporting File 1**: mco270866‐sup‐0001‐SuppMat.pdf

## Data Availability

The authors have nothing to report.
