# Supplementary material for: SIRT Family: Biological Functions and Therapeutic Targets
Source: MedComm (2020). 2026 Jul 22;7(8):e70866. doi: 10.1002/mco2.70866 (PMC13390616; doi:10.1002/mco2.70866)
Supplement: Supplementary file 1 — Supporting File 1: mco270866‐sup‐0001‐SuppMat.pdf [file MCO2-7-e70866-s001.pdf]

SIRT family: biological functions and therapeutic targets

Jia-Yi Wang [1 #], Feng-Li Jiang [2 #], Fang-Yuan Zhang [3 #], Dong-Hui Huang [4], Xiao-Ying Li [4], Song Gao [2], Hua You [5 *], Qi-Jun Wu [6, 7, 8 *], Huan-Huan Chen [4, 9 *], Ting-Ting Gong [1 *]

1. Department of Obstetrics and Gynecology, Liaoning Institute of birth health and development, Reproductive Hospital of China Medical University, China.
2. Department of Obstetrics and Gynecology, Shengjing Hospital of China Medical University, Shenyang, China.
3. Department of General Surgery, Shengjing Hospital of China Medical University, Shenyang, China.
4. Department of Clinical Epidemiology, Shengjing Hospital of China Medical University, Shenyang, China.
5. Laboratory for Excellence in Systems Biomedicine of Pediatric Oncology, Department of Pediatric Hematology and Oncology, Chongqing Key Laboratory of Pediatric Metabolism and Inflammatory Diseases, Ministry of Education Key Laboratory of Child Development and Disorders, National Clinical Research Center for Child Health and Disorders, Children's Hospital of Chongqing Medical University, Chongqing, China.
6. Department of Epidemiology, School of Public Health, China Medical University, Shenyang, China.

7. Key Laboratory of Environmental Stress and Chronic Disease Control & Prevention, Ministry of Education, China Medical University, Shenyang, China.
8. NHC Key Laboratory of Advanced Reproductive Medicine and Fertility (China Medical University), National Health Commission, Shenyang, China.
9. Department of Oncology, Shengjing Hospital of China Medical University, Shenyang, China.

# These authors contributed equally to this work.

*Corresponding author:

Hua You (youhua307@163.com)

Qi-Jun Wu (wuqj@sj-hospital.org)

Huan-Huan Chen (749509033@qq.com)

Ting-Ting Gong (gongtt@sj-hospital.org)

Supplementary Table S1. Summary of global knockout studies of *Sirt* genes in transgenic mice

Gene	Mutation type	Discovery (year/lab/1st author)	Genotype	Survival	Size/weight	Fetal growth	Cause of death	Reproductive capacity	Circulatory system	Respiratory system	Digestive system	Nervous system	Endocrine system	Urogenital system	Musculoskeletal system	Cancer	Immune system	Others	Reference
*Sirt*1	exon 5-6 del	2003 McBurney	hom	0%, 1 mon, inbred 129/Sv	↓	↓	n/a	cannot survive to reproductive stage	right ventricular hypertrophy	↓ lung	↓ spleen	exencephaly	↓ pancreas	n/a	↓ mineralization of the digits;↓ cartilage	n/a	↓ CD8-positive T cells in spleen	↓ eyelid;short snout	[9-11]
			hom	50%, ≥12 mon, outbred 129/Sv-CD1	↓	NC	n/a	loss ♂. loss ♀	right ventricular hypertrophy	↓ lung	↓ spleen	exencephaly;↓ GnRH neuronal migration	↑metabolism;↓glucose clearance;↓pancreas;	↓ sperm; ↓ hormone ♀;↓ ovary	↓ bone	n/a	↓ CD8-positive T cells in spleen	↓ eyelid;short snout;lethargy;↓ white adipose	[9, 12-14]
			het	some > 6 mon, inbred 129/SvJ	↓	n/a	n/a	NC	n/a	n/a	n/a	n/a	n/a	n/a	↓ cartilage;↓ craniofacial	n/a	n/a	↓ eye;↓ fur	[11]
	exon 4 del		hom	33%, 1 wee, 129/Sv	↓	↓	heart	loss ♂; NC ♀	↓ heart	n/a	n/a	exencephaly	n/a	↓ sperm; NC oocyte	n/a	n/a	NC	↓ eye;↓ retinal	[15, 16]

		2003 Frederick (Cheng)	het	most > 12 mon, C57BL6/J	NC	NC	n/a	NC ♂; NC ♀	n/a	n/a	n/a	n/a	↓ glucose metabolism	NC sperm; NC oocyte	n/a	n/a	n/a	↓ age-related hearing	[16, 17]
	mut exon del 5	2011 McBurney (Seifert)	hom	61%, 3 wee, inbred 129/SvJ	n/a	n/a	n/a	n/a	n/a	n/a	n/a	n/a	n/a	n/a	n/a	n/a	n/a	n/a	[12]
				64%, 3 wee, outbred 129/Sv-CD1	↓	NC	n/a	loss ♂; NC ♀	NC heart	↓ lung; elevated respiration	↓ liver; NC spleen	NC brain	↓ glucose clearance; ↓ metabolism; NC pancreas	↓ kidney; ↓ kidney aging ♂; ↓ sperm	n/a	n/a	n/a	short snout;↓white adipose	[12, 18]
			het	NC, inbred 129/SvJ	NC	n/a	n/a	n/a	n/a	n/a	n/a	n/a	n/a	n/a	n/a	n/a	n/a	n/a	[12]
				50%, 100 wee, outbred 129/Sv-CD1	NC	NC	n/a	NC ♂; NC ♀	n/a	n/a	n/a	n/a	n/a	NC kidney	n/a	n/a	n/a	aging ♂	[12, 18]
Sirt2	exon 5-8 del	2011 Deng (Kim)	hom	↓, C57BL/6	NC	NC	cancer	NC	NC	lung cancer	liver cancer; stomach cancer; duodenum cancer	NC	pancreatic cancer	prostate cancer; mammary cancer	NC	↓	B cell lymphoma	n/a	[19]
	exon 5-6,part 7 del	2013 Vaquero and Serrano	hom	↓, C57BL/6	NC	NC	NC	NC	NC	NC	NC	NC	NC	NC	NC	tumorigenesis (skin tumorigenesis assay)	↑ tumor-reactive T cells	↓genome stability	[20, 21]

*Sirt*3	exon 2-3 del	2007 Frederick and Verdin（Lombard and Schwer）	hom	> 12 mon, C57BL/6J	NC	NC	n/a	NC	NC heart (structure)	NC	NC liver (structure)	NC brain (structure)	NC	NC kidney (structure)	NC (structure)	n/a	n/a	prone to aging	[22]
	exon 2-4 del	2008 Deng（Ahn and Kim）	hom	> 12 mon	NC	NC	n/a	NC	↓ heart basal ATP level	n/a	↓ liver basal ATP level	n/a	NC pancreas	↓ kidney basal ATP level	n/a	n/a	n/a	n/a	[23]
*Sirt*4	exon 1-3 del	2006 Guarente (Haigis)	hom	NC, 129Sv	NC	NC	n/a	NC	NC	NC	NC	NC	↑ insulin secretion; ↑ insulin resistance (C57BL/6NJ);↓ lipid synthesis	NC	↑ exercise tolerance	promote liver cancer; promote lymphoma (C57BL/6)	n/a	n/a	[24-28]
*Sirt*5	exon 2-5 del	2007 Frederick and Verdin（Lombard and Schwer）	hom	> 18 mon, 129Sv	NC	NC	NC	NC	NC	NC	NC	NC	↓ blood ammonia (fasted); ↓ urea cycle	NC	NC	NC	NC	n/a	[22, 29]

Sirt6	exon 1-6 del	2006 Frederick (Mostoslavsky, Chua and Lombard)	hom	0%, 4 wee, 129SvJ	↓	↓	acute degenerative processes	↓	NC	NC	↓ superficial colonic epithelium	NC	↓ subcutaneous fat; hypoglycemia	NC	osteopenia; lordokyphosis	NC	↓ lymphocyte	prone to aging	[30]
			het	3-9 mon, 129SvJ	NC	NC	NC	NC	↓ endothelial; ↓heart	n/a	NC	NC	NC	NC	NC	NC	↑	prone to aging	[31-33]
Sirt7	exon 4-9 del	2008 Bober (Vakhrusheva)	hom	50%, 8mon, mixed C57Bl/6 and 129Sv	n/a	↓	heart	NC	↓ heart	NC	NC	NC	↓ subcutaneous fat	NC	↓ kyphosis	NC	NC	prone to aging	[34]
	exon 4-11 del	2013 Shin, He Liu, Paredes Frederick, Chua and Chen	hom	80%, 1mon/0%, 20 mon, mixed C57Bl/6 and 129Sv	↓	NC	n/a	n/a	n/a	n/a	↑ hepatic steatosis; ↓ spleen	n/a	↓ fat	n/a	↓kyphosis	n/a	↓ bone marrow; ↓ thymus	↓ genome stability; prone to aging; ↓ER stress	[35, 36]

Abbreviations: Del, deletion; het: heterozygous; hom: homozygous; n/a: not applicable/not detected; NC: no change; mon: months of age; wee: weeks of age; ♂ : males; ♀ : females; (): model;↓: decreased/ smaller organ/adverse effect; ↑: increased/ enlarged organ/ protective effect.

Supplementary Table S2. Summary of global overexpression studies of *Sirt* genes in transgenic mice

Gene	Discovery (year/lab/1st author)	Genotype	Survival	Size/weight	Fetal growth	Cause of death	Reproductive capacity	Circulatory system	Respiratory system	Digestive system	Nervous system	Endocrine system	Urogenital system	Musculoskeletal system	Cancer	Immune system	Others	Reference
Sirt1	2008 Gu, and Accili (Banks)	hom	≥11 mon, C57BL/6J	NC	NC	NC	↓ oxygen consumption	NC	NC	↑ hepatic insulin sensitivity	n/a	↑ glucose tolerance	n/a	n/a	n/a	n/a	↓ body temperature; ↓ locomotor activity	[1]
	2007 Bordone	hom	0, mixed C57BL/6 and 129/Sv	n/a	n/a	n/a	n/a	n/a	n/a	n/a	n/a	n/a	n/a	n/a	n/a	n/a	n/a	[2]
		hem	≥12 mon, mixed C57BL/6 and 129/Sv	↓	↓	n/a	↓ oxygen consumption	↓ blood cholesterol	n/a	n/a	n/a	↑ glucose tolerant; ↓ adipokines; ↓ insulin; ↓ fasted glucose; ↓ white adipose tissue	n/a	n/a	n/a	n/a	↑ rotarod challenge	[2]
Sirt2	2014 Sinclair (North)	n/a	↑ (BubR1 hypomorphic), C57BL/6J	NC	NC	n/a	n/a	reverse cardiac abnormalities (BubR1 hypomorphic)	n/a	n/a	n/a	n/a	n/a	n/a	n/a	n/a	n/a	[3]
Sirt3	2014 Brown (Verdin)	hem	n/a, C57BL/6	n/a	n/a	n/a	n/a	n/a	n/a	n/a	n/a	n/a	n/a	n/a	n/a	n/a	↑ hearing	[4]
	2020 Dikalov (Dikalova)	hom	≥12 mon, C57BL/6J	n/a	n/a	n/a	n/a	↑ vascular function	n/a	n/a	n/a	n/a	n/a	n/a	n/a	n/a	inhibit aging	[5]
Sirt5	2010 Inagaki (Ogura)	hom	≥14 wee, C57BL/6	NC	NC	NC	NC	NC	NC	↑ ammonia detoxification	NC	NC	NC	NC	n/a	n/a	protein hypoacylation	[6, 7]
Sirt6	2010 Cohen (Kanfi)	hom	increase lifespan♂; ≥6 mon, C57BL/6J	NC	NC	NC	n/a	n/a	n/a	NC liver	n/a	↑ glucose tolerance; ↓ fat; ↓ LDL-cholesterol; ↓ triglycerides	n/a	NC lean body mass	n/a	n/a	protect diet-induced obesity	[8]

Abbreciations: Del, deletion; het: heterozygous; hom: homozygous; n/a: not applicable/not detected; NC: no change; mon: months of age; wee: weeks of age; ♂ : males; ♀ : females; (): model;↓: decreased/ smaller organ/adverse effect; ↑: increased/ enlarged organ/ protective effect.

Supplementary Table S3. Summary of phenotypes and mechanisms of *Sirt* genes in transgenic mice with cardiovascular diseases

Gene	Disease	Strains	Cre promoter	system specific	KO or OE	Inducible	Model background	Phenotype	Mechanism and conclusion	Reference
Sirt1	arrhythmia	C57BL/6J	α-MHC	cardiomyocyte	KO	no	no	Specific *Sirt*1 KO mice exhibit cardiac dysfunction, increased ventricular arrhythmia, and impaired Ca^{2+} and Na^{+} regulation with elevated ROS production.	SIRT1 protects against cardiac dysfunction and arrhythmogenesis by regulating intracellular Ca^{2+} and Na^{+} homeostasis, and its deficiency exacerbates oxidative stress and disrupts ion balance.	[37]
Sirt1	arrhythmia	C57BL/6	Mef2c	atria and right ventricle	KO	no	age-related atrial fibrillation model	The *Sirt*1KO mice exhibit an enlarged atrial diameter and heightened vulnerability to atrial fibrillation	SIRT1 seficiency promotes age-related atrial fibrillation through enhancing atrial necroptosis by activation of RIPK1 acetylation	[38]
Sirt1	arrhythmia	C57BL/6	α-MHC	cardiomyocyte	KO	tamoxifen	no	*Sirt*1 cardiac-specific KO mice exhibited cardiac hypertrophy, impaired cardiac function, and abnormal electrophysiological activities, leading to arrhythmia	SIRT1 regulates cardiac function and energy metabolism by modulating lysine crotonylation of SERCA2a and PPAR pathway proteins, with its deficiency causing cardiac dysfunction and arrhythmia.	[39]
Sirt1	mitochondrial metabolism and cardiac dysfunction	C57BL/6J	α-MHC	cardiomyocyte	KO	tamoxifen	transverse aortic constriction induced cardiac pressure overload.	Specific *Sirt*1 KO mice exhibit progressive cardiac dysfunction with increased sensitivity to pressure overload, characterized by mild left ventricular systolic dysfunction and elevated mitochondrial ROS production.	SIRT1 plays a cardioprotective role by re31269804gulating cardiac energy metabolism and mitochondrial function, and its deficiency exacerbates cardiac dysfunction and oxidative stress under stress conditions.	[40]
Sirt1	mitochondrial metabolism and cardiac dysfunction	FVB	α-MHC	cardiomyocyte	OE 2.5, 7.5, and 12.5 fold	no	transverse aortic constriction induced cardiac pressure overload;oxidative stress induced by paraquat	Moderate OE of *Sirt*1 in the heart retards age-dependent cardiac hypertrophy, fibrosis, and dysfunction, while high levels of *Sirt*1 induce cardiomyopathy with increased apoptosis and oxidative stress.	SIRT1 regulates cardiac aging and resistance to oxidative stress by modulating apoptosis, fibrosis, and mitochondrial function through FoxO-dependent pathways, with beneficial effects observed at low to moderate expression levels.	[41]
Sirt3	mitochondrial metabolism and cardiac dysfunction	C57BL/6; 129	no	global	KO	no	no	*Sirt*3 KO mice exhibit shortened lifespan and severe cardiac abnormalities, including hypertrophy and fibrosis, as they age.	SIRT3 plays a critical role in maintaining cardiac mitochondrial function by regulating optic atrophy 1 deacetylation, and its deficiency leads to impaired mitochondrial bioenergetics and heart failure.	[42]
Sirt3	mitochondrial	129/SvImJ	no	global	KO	no	transverse	*Sirt*3 KO mice exhibit progressive cardiac	SIRT3 is crucial for maintaining	[43]

Gene	Disease	Strains	Cre promoter	system specific	KO or OE	Inducible	Model background	Phenotype	Mechanism and conclusion	Reference
	metabolism and cardiac dysfunction						aortic constriction induced cardiac pressure overload;	dysfunction, hypertrophy, and fibrosis with age, characterized by impaired mitochondrial energetics and substrate oxidation.	cardiac mitochondrial function by regulating the acetylation status of key metabolic enzymes; its deficiency leads to energy depletion and contractile dysfunction, potentially contributing to heart failure.	
Sirt1	mitochondrial metabolism and cardiac dysfunction	C57BL/6J	α-MHC	cardiomyocyte	KO	no	transverse aortic constriction	*Sirt*1 KO mice exhibit impaired cardiac contractility and increased apoptosis associated with elevated oxidative stress and ER stress as they age.	SIRT1 protects the heart from age-related contractile dysfunction by inhibiting ER stress-mediated apoptosis through the regulation of oxidative stress and nitric oxide synthase expression.	[44]
Sirt1	mitochondrial metabolism and cardiac dysfunction	C57BL/6J	UBC-Cre-ERT2	global	KO	tamoxifen	tunicamycin elicites ER stress; ISO elicits cardiac dysfunction associated with ER stress	*Sirt1* KO mice exhibit exacerbated cardiac dysfunction and reduced autophagy in response to ER stress, indicating a protective role of SIRT1 in the heart.	SIRT1 protects the heart from ER stress-induced injury by promoting autophagy through the eEF2K/eEF2 pathway, thereby reducing cell death and maintaining cardiac function.	[45]
Sirt3	cardiac fibrosis	B6; 129S5	no	global	KO	no	no	*Sirt*3 KO mice exhibit cardiac fibrosis and inflammation characterized by increased transcriptional activity of AP-1, but do not show cardiac hypertrophy or dysfunction.	SIRT3 exerts anti-inflammatory and anti-fibrotic effects in the heart by inhibiting FOS transcription through histone H3 deacetylation, thus attenuating the FOS/AP-1 pathway, which is implicated in cardiac hypertrophy, heart failure, and diabetic cardiomyopathy.	[46]
Sirt3	cardiac fibrosis	no	α-MHC	cardiomyocyte	KO	no	no	*Sirt*3 KO mice exhibit significant cardiac remodeling, increased cardiac fibrosis, and enhanced ferroptosis, characterized by elevated levels of 4-HNE and reduced GPX-4 expression.	SIRT3 deficiency enhances ferroptosis and promotes cardiac fibrosis via p53 acetylation	[47]
Sirt3	cardiac fibrosis	n/a	no	global	KO	no	cardiac hypertrophy and fibrosis induced by transverse aortic constriction	*Sirt*3 KO mice exhibit exacerbated cardiac hypertrophy and fibrosis in response to hypertrophic stimuli.	SIRT3 protects from hypertrophy and fibrosis via the TGF-β/Smad3 pathway.	[48]
Sirt1	Cardiomyopat	n/a	no	global	KO	no	no	*Sirt*1 KO mice develop dilated	SIRT1 plays a critical role in	[49]

Gene	Disease	Strains	Cre promoter	system specific	KO or OE	Inducible	Model background	Phenotype	Mechanism and conclusion	Reference
	hy				((*Sirt*1Δ E4, homozygous and heterozygous)			cardiomyopathy characterized by ventricular dilation and reduced cardiomyocyte size without cardiac hypertrophy or fibrosis.	maintaining cardiac mitochondrial integrity and normal myocardial development by regulating the acetylation status of Mef2 transcription factors, which are essential for mitochondrial function and heart development.	
Sirt2	Cardiomyopathy	C57BL/6	no	global	KO	no	isoproterenol-induced hypertrophy	*Sirt*2 KO mice develop spontaneous cardiac hypertrophy, fibrosis, and heart failure in an age-dependent manner, with exacerbated hypertrophic responses to agonist stimulation.	SIRT2 acts as an endogenous negative regulator of cardiac hypertrophy by deacetylating and inhibiting the transcriptional activity of nuclear factor of activated T cells, cytoplasmic 2, thereby preventing pathological cardiac remodeling and heart failure.	[50]
Sirt3	Cardiomyopathy	no	α-MHC	cardiomyocyte	KO	no	salt-induced cardiac hypertrophy	*Sirt*3 KO mice exhibit exacerbated cardiac hypertrophy and dysfunction	SIRT3 protects against salt-induced cardiac hypertrophy by preserving mitochondrial function through deacetylating MPC1 and maintaining glucose oxidation, which is crucial for preventing metabolic reprogramming and cardiac hypertrophy	[51]
Sirt6	Cardiomyopathy	129sv/C57BL6	α-MHC	cardiomyocyte	KO; OE	tamoxifen	transverse aortic constriction, isoproterenol or angiotensin II induced hypertrophy	*Sirt*6 KO mice develop cardiac hypertrophy and heart failure, while SIRT6 transgenic mice are protected from hypertrophic stimuli.	SIRT6 acts as a negative regulator of cardiac hypertrophy by inhibiting IGF-Akt signaling through deacetylation of histone H3K9 and suppression of c-Jun transcriptional activity, thereby preventing the development of cardiac hypertrophy and heart failure.	[52]
Sirt7	Cardiomyopathy	C57Bl/6J	α-MHC	cardiomyocyte	KO	no	transverse aortic constriction; angiotensin II induced hypertrophy	*Sirt*7 KO mice exhibit exacerbated cardiac hypertrophy and fibrosis in response to pressure overload, characterized by increased heart weight, cardiomyocyte size, and interstitial fibrosis.	SIRT7 exerts an antihypertrophic effect by interacting with and promoting the deacetylation of GATA4, thereby regulating cardiac hypertrophy and remodeling. This interaction is critical for preventing pathological cardiac hypertrophy and heart failure.	[53]

Gene	Disease	Strains	Cre promoter	system specific	KO or OE	Inducible	Model background	Phenotype	Mechanism and conclusion	Reference
Sirt7	Cardiomyopathy	mixed C57Bl/6 and 129Sv background	no	global	KO	no	no	*Sirt*7 KO mice exhibit an inflammatory cardiomyopathy phenotype characterised by markedly reduced lifespan, progressive Cardiomyopathy with extensive interstitial fibrosis , lipofuscin deposition, inflammatory cell infiltration, and increased myocardial cell apoptosis.	SIRT7 suppresses p53 transcriptional activity through deacetylation modification. Its absence leads to excessive p53 acetylation and activation, thereby promoting apoptosis. This is accompanied by overactivation of the Akt signalling pathway, downregulation of PTEN, and enhanced Ras-Raf-MEK-ERK cascade reactions, collectively driving cardiomyocyte death and pathological remodelling.	[54]
Sirt3	carotid thrombosis	C57BL6/J	no	global	KO	no	LPS induced inflammatory	*Sirt*3 KO mice exhibit accelerated arterial thrombosis, increased neutrophil extracellular trap formation, and elevated plasma tissue factor activity.	SIRT3 protects against arterial thrombosis by reducing oxidative stress and inhibiting neutrophil extracellular trap formation and tissue factor activity.	[55]
Sirt3	MI	129S1/SvImJ	no	global	KO	no	ligation of left anterior descending coronary artery induces myocardial ischemia	*Sirt*3 KO mice exhibit coronary microvascular dysfunction, reduced capillary density, and impaired cardiac recovery following myocardial ischemia, characterized by decreased cardiac function and increased apoptosis.	SIRT3 protects against post-myocardial infarction cardiac dysfunction by maintaining coronary microvascular function and promoting angiogenesis through regulation of angiogenic growth factors and metabolic pathways.	[56]
Sirt5	MI	C57BL/6J	albumin	liver	OE	no	ligation of the left coronary artery induces MI	Hepatocyte-specific *Sirt*5 OE mice exhibit reduced MI and fibrosis areas, improved cardiac function, and increased circulating and cardiac FGF21 levels following acute myocardial infarction.	SIRT5 exerts a cardioprotective effect by enhancing hepatic FGF21 secretion and improving energy metabolism through a liver-cardiac crosstalk mechanism, potentially involving fatty acid β-oxidation and mitochondrial metabolism.	[57]
Sirt1	IRI	C57BL/6J	α-MHC	cardiomyocyte	KO	tamoxifen	IRI model with coronary artery occlusion and reperfusion	Specific *Sirt*1 KO mice exhibit increased susceptibility to IRI, characterized by impaired cardiac function, larger infarct size, and altered metabolic responses.	SIRT1 protects against ischemic heart disease by modulating AMPK activation via LKB1 deacetylation, thereby regulating cardiac metabolism and stress responses.	[58]
Sirt1	IRI	C57BL/6J	α-MHC	cardiomyocyte	KO	tamoxifen	IRI model with coronary artery	Specific *Sirt*1 KO mice exhibit increased susceptibility to IRI, characterized by	SIRT1 modulates cardiac NLRP3 inflammasome activation through	[59]

Gene	Disease	Strains	Cre promoter	system specific	KO or OE	Inducible	Model background	Phenotype	Mechanism and conclusion	Reference
							occlusion and reperfusion	higher levels of NLRP3 inflammasome activation, increased ROS generation, and more pyroptosis.	metabolic regulation of pyruvate dehydrogenase during IRI, thereby reducing inflammation and pyroptosis.	
Sirt1	IRI	C57BL/6J	α-MHC	cardiomyocyte	KO	no	caloric restriction	Specific *Sirt*1 KO mice exhibit exacerbated IRI, characterized by impaired recovery of left ventricular function and increased lactate dehydrogenase release.	SIRT1 in cardiomyocytes mediates the cardioprotective effects of caloric restriction by suppressing local complement system activation through downregulation of complement component 3 expression, thereby reducing inflammation and tissue damage during IRI.	[60]
Sirt3	IRI	129S6/SvEvTac	no	global	KO	no	IRI model with coronary artery occlusion and reperfusion	*Sirt*3 KO mice exhibit increased susceptibility to IRI, characterized by larger infarct size and failure to respond to ischemic postconditioning.	SIRT3 protects against IRI by deacetylating cyclophilin D , thereby inhibiting the opening of the mitochondrial permeability transition pore and reducing cell death.	[61]
Sirt7	MI	C57BL/6	no	global	KO	no	MI and hind-limb ischemia, model	*Sirt*7 KO mice exhibit impaired wound healing, reduced fibrosis, and delayed blood flow recovery after MI and hind-limb ischemia, leading to increased susceptibility to cardiac rupture and poor tissue repair.	SIRT7 stabilizes TβRI by modulating autophagy and interacting with protein interacting with PICK1, thereby promoting TGF-β signaling and contributing to tissue repair processes.	[62]
Sirt1	drug-induced cardiac damage	C57BL/6J	myh6-CreEsr1	cardiomyocyte	KO	tamoxifen	DOX-induced cardiotoxicity	*Sirt*1 KO mice exhibit exacerbated cardiac dysfunction, hypertrophy, and oxidative stress in response to DOX-induced cardiotoxicity.	SIRT1 protects against DOX-induced cardiotoxicity by targeting SESN2 to reduce oxidative stress and apoptosis via the AMPK pathway.	[63]
Sirt3	drug-induced cardiac damage	n/a	no	global	KO	no	DOX-induced cardiotoxicity	*Sirt*3 KO mice exhibit exacerbated cardiac hypertrophy and increased sensitivity to DOX-induced cardiotoxicity.	SIRT3 protects against DOX-induced cardiomyopathy by maintaining mitochondrial DNA integrity and regulating oxidative stress through OGG1.	[64]
Sirt3	drug-induced cardiac damage	C57BL/6	no	global	KO	no	DOX-induced cardiotoxicity	*Sirt*3 OE exhibit resistance to doxorubicin-induced cardiac remodeling and dysfunction, preserving cardiac function and reducing oxidative stress.	SIRT3 protects against DOX-induced cardiotoxicity by modulating protein acetylation and reducing oxidative stress through the deacetylation of key mitochondrial proteins.	[65]
Sirt3	drug-	no	no	global	KO	no	sunitinib-	*Sirt*3 KO mice are protected against	SIRT3 promotes sensitivity to	[66]

Gene	Disease	Strains	Cre promoter	system specific	KO or OE	Inducible	Model background	Phenotype	Mechanism and conclusion	Reference
	induced cardiac damage						induced cardiotoxicity	sunitinib-induced cardiotoxicity.	sunitinib-induced cardiotoxicity by inhibiting the GSTP1/JNK/autophagy pathway in cardiac pericytes.	
Sirt6	drug-induced cardiac damage	C57BL/6J	no	global	KO (heterozygous)	no	DOX-induced cardiotoxicity	*Sirt*6 KO mice exhibit exacerbated cardiac dysfunction, apoptosis, and oxidative stress in response to DOX-induced cardiotoxicity.	SIRT6 protects against doxorubicin-induced cardiotoxicity by inhibiting p53/Fas-dependent cell death and augmenting endogenous antioxidant defense mechanisms.	[67]
Sir1	HF	C57BL/6J	α-MHC	cardic specific	KO	no	no	Cardiac-specific *Sirt*1 KO mice exhibit impaired calcium handling, reduced contractility, and adverse left ventricular remodeling with decreased ejection fraction	SIRT1 reduced SERCA2a acetylation and restored its activity in HF	[68]
Sirt1	congenital heart disease	C57BL/6	ISL1	ISL1+ cardiac progenitor cells	KO	no	$CoCl_2$ induced hypoxia responses	*Sirt*1 KO in ISL1+ cardiac progenitor cells prevents hypoxia-induced congenital heart defects by maintaining Isl1 expression and promoting progenitor cell proliferation.	SIRT1 forms a complex with HIF1α and HES1 to repress Isl1 expression under hypoxic conditions, leading to reduced progenitor cell proliferation and increased cardiomyocyte specification, contributing to congenital heart defects.	[69]
Sirt3	HF	C57BL/6; 129-*Sirt*3tm1.1Fwa/J; 129/SvlmJ	no	global	KO	no	pressure-overloaded HF models	*Sirt*3 KO mice exhibit exacerbated cardiac dysfunction and energy metabolism abnormalities when combined with mtMOF overexpression.	mitochondrial MOF disrupts cardiac energy metabolism and induces heart failure by hyperacetylating ATP5B, a key component of the ATP synthase complex, which is counteracted by SIRT3.	[70]
Sirt3	HF	C57BL/6	MyHC	cardiomyocyte	KO	no	no	*Sirt*3 KO mice exhibit severe cardiac dysfunction	SIRT3 protects against cardiac dysfunction by regulating mitochondrial iron homeostasis and ferroptosis; its deficiency activates mitochondrial ferroptosis through increased p53 acetylation	[71]

Gene	Disease	Strains	Cre promoter	system specific	KO or OE	Inducible	Model background	Phenotype	Mechanism and conclusion	Reference
									and impaired iron-sulfur cluster biogenesis, leading to cardiac dysfunction	
Sirt3	HF	C57BL/6J	no	global	KO	no	ISO and TAC	*Sirt*3 KO mice exhibited exacerbated cardiac hypertrophy and heart failure, with no significant improvement from NADPH treatment.	SIRT3 plays a crucial role in preventing pathological cardiac hypertrophy and heart failure by deacetylating target proteins to improve mitochondrial function and energy metabolism.	[72]
Sirt1	arterial stiffness	C57BL/6	no	global	OE	no	no	Lifelong *Sirt*1 OE in mice attenuates age-related aortic stiffening and prevents deleterious structural changes in the aorta.	SIRT1 protects against age-related aortic stiffening by maintaining elastin content and preventing collagen accumulation, AGEs, and calcification, potentially through enhanced antioxidant capacity.	[73]
Sirt1	arterial stiffness	C57Bl/6J	no	global	KO	no	high fat, high sucrose diet induced aortic stiffness	*Sirt*1 deletion in VSM exacerbates diet-induced arterial stiffness.	SIRT1 protects against diet-induced arterial stiffness by reducing inflammation and oxidants in the aorta, suggesting its potential as a therapeutic target for cardiovascular complications in metabolic syndrome.	[74]
Sirt1	arterial stiffness	B6.129S6-Taglntm2 (cre) Yec/J	SM22α	VSMC	OE	no	acarotid artery ligation injury model	*Sirt*1 KO mice exhibit increased neointimal formation and enhanced vascular inflammation in response to injury.	SIRT1 exerts anti-inflammatory effects in VSMC by promoting SM22α expression through a CKII-SIRT1-SM22α loop, which inhibits EZH2-mediated epigenetic silencing of SM22α.	[75]
Sirt2	arterial stiffness	C57BL/6	no	global	KO	no	natural aging model and angiotensin II-induced vascular remodeling model	*Sirt*2 KO mice exhibited aggravated ageing-induced vascular remodelling, increased arterial stiffness, and impaired vascular function.	SIRT2 acts as a cytoplasmic deacetylase to inhibit p66Shc and mROS production, thereby repressing vascular ageing and remodelling.	[76]
Sirt1	atherosclerosis	C57BL/6	Tie2	endothelium	KO	no	apolipoprotein E-deficient mice model of atherosclerosis	*Sirt*1 KO mice exhibit increased atherosclerosis in both athero-prone and athero-protective areas.	SIRT1 promotes an anti-inflammatory and anti-oxidative phenotype in endothelial cells by deacetylating targets such as eNOS and FoxO1, thereby reducing atherosclerosis.	[77]
Sirt1	atherosclerosis	C57BL/6	VE-Cadherin	endotheliuml	OE	no	apolipoprotein	*Sirt*1OE mice exhibit improved high fat-	SIRT1 protects against	[78]

Gene	Disease	Strains	Cre promoter	system specific	KO or OE	Inducible	Model background	Phenotype	Mechanism and conclusion	Reference
							E-deficient mice model of atherosclerosis	induced impairment in endothelium-dependent vasorelaxation	atherosclerosis by inhibiting endothelial cell apoptosis and improving endothelial function, likely through upregulation of endothelial nitric oxide synthase and increased nitric oxide production.	
Sirt1	atherosclerosis	C57BL/6J	n/a	endotheliuml	OE	no	apolipoprotein E-deficient mice model of atherosclerosis	CDK5 as an upstream kinase promoting S47 phosphorylation. Hyperphosphorylation at this residue abolished the antisenescent and anti inflammatory activity of *Sirt*1OE.	SIRT1 inhibits endothelial senescence and atherosclerosis by preventing hyperphosphorylation at serine 47, which is mediated by cyclin-dependent kinase 5 . This phosphorylation alters SIRT1's intracellular localization and interactions, reducing its anti-aging and anti-inflammatory activities.	[79]
Sirt1	atherosclerosis	129; C57BL/6J	no	global	KO (*Sirt*1-null heterozygous)	no	apolipoprotein E-deficient mice model of atherosclerosis	*Sirt*1 KO mice exhibit increased atherosclerosis, characterized by larger plaques and enhanced macrophage and T-cell accumulation in the aortic wall.	SIRT1 reduces atherosclerosis by decreasing macrophage foam cell formation through the suppression of Lox-1 expression via deacetylation of RelA/p65, thereby inhibiting NF-κB signaling.	[80]
Sirt3	atherosclerosis	C57BL/6	VE-Cadherin	endotheliuml	KO	no	apolipoprotein E-deficient mice model of atherosclerosis	*Sirt*3 KO mice exhibit increased vascular inflammation, oxidative stress, and endothelial dysfunction, leading to exacerbated atherosclerosis.	SIRT3 protects against vascular inflammation and atherosclerosis by regulating mitochondrial function and reducing oxidative stress. NAD^+ supplementation exerts both SIRT3-dependent and -independent effects, improving endothelial function and reducing plaque formation.	[81]
Sirt6	atherosclerosis	C57BL/6	VE-Cadherin	endotheliuml	KO	no	transient middle cerebral artery occlusion	Specific *Sirt*6 KO mice exhibit increased infarct volumes, mortality, and neurological deficits after transient middle cerebral artery occlusion, indicating exacerbated ischemic stroke outcomes.	SIRT6 protects against ischemic stroke by preserving blood-brain barrier integrity and reducing apoptosis through the Akt salvage pathway, thereby attenuating cerebral damage and improving neurological outcomes.	[82]
Sirt6	endothelial dysfunction	C57BL/6	Tie2	endothelium	KO	no	paraquat dichloride x-	*Sirt*6 KO mice exhibit exacerbated vascular senescence under oxidative stress	SIRT6 protects against endothelial cell senescence by regulating cell	[83]

Gene	Disease	Strains	Cre promoter	system specific	KO or OE	Inducible	Model background	Phenotype	Mechanism and conclusion	Reference
							hydrate - induced senescence	conditions.	cycle progression via FOXM1.	
Sirt1	hypertension	C57Bl/6J	SM22α	VSMC	KO; OE	no	angiotensin II-induced hypertension	*Sirt1* KO in VSMCs leads to high mortality due to aortic dissection under Angiotensin II-induced hypertension, whereas its OE protects against aortic stiffness and dissection.	SIRT1 in VSMCs maintains aortic wall integrity by suppressing oxidant-induced matrix metalloproteinase activity, thus protecting against aortic dissection and rupture.	[84]
Sirt1	hypertension	C57/BL6	SM22α	VSMC	OE	no	angiotensin II-induced hypertension	*Sirt1* OE in VSMCs reduces systolic blood pressure and alleviates vascular remodeling induced by Angiotensin II infusion,	SIRT1 in VSMCs inhibits AngII-induced hypertension and vascular remodeling by reducing ROS production and suppressing TGF-β1 expression and its downstream signaling pathway, thereby protecting against hypertension and related vascular diseases.	[85]
Sirt3	hypertension	C57BL/6J	no	global	KO; OE	no	angiotensin II or DOCA-salt induced hypertension	*Sirt3* KO mice exhibit increased oxidative stress, vascular inflammation, and hypertension, with accelerated vascular aging and age-dependent blood pressure elevation, while *Sirt*3 OE mice show reduced oxidative stress, preserved endothelial function, and attenuated hypertension.	SIRT3 protects against vascular dysfunction and hypertension by reducing oxidative stress, inhibiting inflammation, and preserving endothelial function through deacetylation of SOD2 and regulation of metabolic pathways.	[86]
Sirt3	hypertension	C57BL/6J	no	global	KO	no	angiotensin II induced hypertension	*Sirt3* KO mice exhibit increased vascular oxidative stress, diminished endothelial nitric oxide, and exacerbated hypertension in response to angiotensin II infusion.	SIRT3 protects against hypertension by deacetylating and activating SOD2, thereby reducing mitochondrial oxidative stress and preserving endothelial function. Its impairment leads to SOD2 inactivation and contributes to hypertension.	[87]
Sirt3	hypertension	C57BL/6J	no	global	KO	no	angiotensin II or saline induced hypertension	*Sirt3* KO mice exhibit exacerbated cardiac remodeling, microvascular rarefaction, and mitochondrial dysfunction in response to angiotensin II infusion, leading to increased cardiac fibrosis and impaired angiogenesis.	SIRT3 promotes angiogenesis and attenuates hypertensive cardiac remodeling by enhancing Pink1/Parkin-mediated mitophagy, thereby reducing mitochondrial oxidative stress and preserving cardiac function.	[88]
Sirt3	hypertension	129	no	global	KO	no	angiotensin II-induced	*Sirt3* KO mice exhibit exacerbated cardiac remodeling, reduced lymphatic capillary	SIRT3 promotes lymphangiogenesis and attenuates	[89]

Gene	Disease	Strains	Cre promoter	system specific	KO or OE	Inducible	Model background	Phenotype	Mechanism and conclusion	Reference
							hypertension	density, and impaired cardiac function in response to angiotensin II infusion.	hypertensive cardiac injury by upregulating the vascular endothelial growth factor C/vascular endothelial growth factor receptor3 axis and ERK pathway, thereby reducing cardiac fibrosis and improving cardiac function.	
Sirt1	AAA	C57BL/6J	SM22α	VSMC	KO; OE	no	angiotensin II-induced and calcium chloride-induced mouse models of AAAs; apolipoprotein E-deficient mice model	*Sirt1* KO mice exhibit increased susceptibility to Angiotensin II-induced AAA formation and rupture, characterized by enhanced vascular cell senescence and inflammation.	SIRT1 protects against AAA formation by inhibiting vascular cell senescence and inflammation through the p53-p21 axis, thereby reducing NF-κB activation and MCP-1/chemokine (C-C motif) ligand 2 expression.	[90]
Sirt6	hypertension	C57BL/6	Tie2	endothelium	KO	no	hypertension induced by desoxycorticosterone acetate/salt and angiotensin II	Specific *Sirt6* KO mice exhibit exacerbated hypertension, endothelial dysfunction, and cardiorenal injury.	SIRT6 protects against hypertension and associated organ injuries by maintaining endothelial function through Nkx3.2-GATA5 signaling.	[91]
Sirt6	aortic aneurysms	C57BL/6	no	VSMC-specific	KO	no	no	Genetic KO of *Sirt6* in mouse vascular smooth muscle cells accelerated thoracic aortic aneurysms formation and rupture, reduced survival, and increased vascular inflammation and senescence after angiotensin II infusion	SIRT6 inhibits the recruitment of transcription factor IRF8 and IL-1β transcription by directly binding to the IL-1β promoter and mediating the deacetylation of H3K9 and H3K56; *Sirt*6 deficiency induces hyperacetylation of histones (H3K9ac/H3K56ac) in the IL-1β promoter region, thereby promoting IRF8 binding and driving IL-1β expression upregulation. This subsequently triggers downstream inflammatory cascades and cellular senescence, ultimately accelerating the onset and progression of TAA.	[92]
Sirt6	aortic	C57BL/6	no	global	OE	no	no	*Sirt6* OE markedly suppressed Ang II-	SIRT6 blocks the pathological	[93]

Gene	Disease	Strains	Cre promoter	system specific	KO or OE	Inducible	Model background	Phenotype	Mechanism and conclusion	Reference
	aneurysms							induced abdominal aortic aneurysm formation, manifested by reduced aortic dilatation, preservation of elastic fibre structure, retention of vascular smooth muscle cells, and downregulation of ageing markers (P16/P21).	progression of abdominal aortic aneurysms by antagonising stress-induced premature senescence in vascular smooth muscle cells and the subsequent ageing-associated secretory phenotype (SASP)-mediated chronic inflammation and pathological angiogenesis.	
Sirt6	aortic aneurysms	C57BL/6	no	VSMC-specific	KO	no	no	Specific deletion of *Sirt6* in VSMCs markedly accelerates the formation and rupture of aortic aneurysms and dissections , manifesting as aortic dilatation, elastic fibre rupture, and exacerbated vascular fibrosis. This is accompanied by increased VSMC apoptosis and senescence, alongside mitochondrial structural disruption and functional impairment.	SIRT6 maintains NRF2 transcriptional activity by binding to NRF2 and deacetylating its K518 site, thereby regulating mitochondrial biogenesis and oxidative phosphorylation-related gene expression. *Sirt*6 deficiency leads to increased NRF2 acetylation, downregulation of mitochondrial complex expression, and functional impairment, ultimately triggering VSMC apoptosis and senescence while promoting AAD progression.	[94]
Sirt1	atherosclerosis	129/sv-CD1 (homozygous); C57BL/6 (heterozygous)	no	global	KO (*Sirt*1-null)	no	no	*Sirt1* KO mice exhibit reduced plasma HDL cholesterol levels, impaired cholesterol efflux, and blunted responses to liver X receptor agonists.	SIRT1 deacetylates liver X receptor proteins, thereby enhancing cholesterol efflux and reducing the risk of atherosclerosis and other age-associated diseases.	[95]
Sirt1	endothelial dysfunction	129/Sv; C57BL/6	Tie2	endothelium	KO	no	hindlimb ischemia	*Sirt1* KO mice exhibit impaired postnatal neovascularization and defective ischemia-induced angiogenesis.	SIRT1 regulates postnatal angiogenesis by modulating the transcriptional activity of Foxo1; its activation could counteract vascular rarefaction associated with aging.	[96]
Sirt1	endothelial dysfunction	C57BL/6	n/a	endothelium	OE	no	streptozotocin-induced diabetic mice	*Sirt1* OE mice exhibit decreased p66Shc expression, improved endothelial function, and reduced oxidative stress.	SIRT1 protects against hyperglycemia-induced endothelial dysfunction by downregulating p66Shc expression through epigenetic chromatin modification.	[97]

Abbreviations: AMPK, monophosphate-activated protein kinase; AP-1, activator protein-1; AAA, abdominal aortic aneurysm; AS, Atherosclerosis; Cdh, cadherine; DOX, doxorubicin; EC,

endothelial cell; eEF, eukaryotic elongation factor; ER stress, endoplasmic reticulum stress; ERK, extracellular signal-regulated kinase; FGF2, fibroblast growth factor 2; FoxO, forkhead box protein O; FOXM1, forkhead box M1; GSTP, glutathione S-transferase P1; HI, hind-limb ischemia; IGF-Akt, insulin-like growth factor -Akt; ISL1, islet 1; IRI, ischemia-reperfusion injury; KO, knockout; Mef2, myocyte enhancer factor 2; MCP-1, monocyte chemoattractant protein 1; NAD, nicotinamide adenine dinucleotide; NLRP3, nucleotide-binding oligomerization domain-like receptor with a pyrin domain 3; NF-κB, nuclear factor-κB; NRF2, nuclear factor erythroid2-related factor2; OGG1, 8-Oxoguanine DNA Glycosylase 1; Parkin, parkin RBR E3 ubiquitin protein ligase; Pink, PTEN-induced kinase; ROS, reactive oxygen species; *Sirt*, sirtuin; SERCA2a, sarco-endoplasmic reticulum Ca2+-ATPase; SOD, superoxide dismutase; TGF-β, transforming growth factor-β; TβRI, transforming growth factor-β type I receptor; VEGF, vascular endothelial growth factor; VSMC, vascular smooth muscle cells; α-MHC, α-myosin heavy chain; n/a, not applicable.

Supplementary Table S4. Summary of phenotypes and mechanisms of *Sirt* genes in transgenic mice with respiratory diseases

Gene	Disease	Strains	Cre promoter	system specific	KO or OE	Inducible	Model background	Phenotype	Mechanism and conclusion	Reference
*Sirt*1	pulmonary fibrosis	C57BL/6	no	global	KO（*Sirt* 1y/y）	no	bleomycin-induced lung fibrosis	*Sirt*1 KO mice exhibit reduced lung fibrosis with deCreased FLIP levels and increased Ku70 acetylation.	SIRT1 promotes fibrosis by stabilizing FLIP via Ku70 deacetylation, contributing to apoptosis resistance in fibroblasts.	[98]
*Sirt*3	pulmonary fibrosis	129/SJ	no	global	KO	no	asbestos and bleomycin-induced lung fibrosis	*Sirt*3 KO mice exhibit increased lung fibrosis, alveolar epithelial cell mitochondrial DNA damage, and apoptosis.	SIRT3 protects against lung fibrosis by reducing mitochondrial DNA damage and apoptosis in alveolar epithelial cells via deacetylation of key mitochondrial proteins like MnSOD and OGG1.	[99]
*Sirt*3	pulmonary fibrosis	C57Bl/6J	no	global	OE, 4-fold	no	asbestos-induced pulmonary fibrosis	*Sirt*3 OE mice exhibit reduced pulmonary fibrosis, lung mtDNA damage, and recruitment of fibrogenic Mo-AMs.	SIRT3 protects against pulmonary fibrosis by preserving mitochondrial DNA integrity and reducing recruitment of fibrogenic macrophages in the lung.	[100]
*Sirt*1	COPD	129/SvJ	no	global	KO (*Sirt*1-null heterozygous)	no	cigarette smoke expose	Elevated levels of autophagy were induced by CS in the lungs of SIRT1 deficient mice.	Inhibition of poly(ADP-ribose)-polymerase-1 (PARP-1) attenuated CS-induced autophagy via SIRT1 activation.	[101]
*Sirt*1	COPD/ emphysema	C57BL/6J; 129/SvJ	Epi ;Mac	airway epithelium- and myeloid cell-specific	KO	no	cigarette smoke exposed COPD	Airway epithelium, but not in myeloid cells *Sirt*1 KO mice develop spontaneous airspace enlargement and are more susceptible to emphysema.	SIRT1 protects against emphysema by reducing cellular senescence via the FOXO3 pathway, independent of inflammation.	[102]
*Sirt*1	asthma	C57BL/6	CD11c	bone dendritic cells	KO	no	OVA induced allergic airway disease	*Sirt*1 conditional KO mice exhibit reduced airway inflammation and Th2 responses.	SIRT1 promotes Th2 responses and airway allergy by repressing PPAR-γ activity in dendritic cells, highlighting its pro-inflammatory role in allergic conditions.	[103]
*Sirt*1	asthma	n/a	LysM	myeloid	KO	no	HDM induced allergic asthma	*Sirt*1 KO mice exhibit exacerbated airway inflammation and increased cytokine production.	SIRT1 suppresses allergic airway inflammation in macrophages by inhibiting the ERK/p38 MAPK pathways.	[104]
*Sirt*2	asthma	C57BL/6	no	global	KO	no	dust mite, ragweed,	*Sirt*2 KO mice exhibit reduced allergic inflammation and goblet cell	SIRT2 protects against allergic asthmatic inflammation by	[105]

Gene	Disease	Strains	Cre promoter	system specific	KO or OE	Inducible	Model background	Phenotype	Mechanism and conclusion	Reference
							and A. fumigatus induced allergic asthma	hyperplasia.	modulating macrophage activation and CCL17 production.	
*Sirt*1	respiratory syncytial virus infection	C57BL/6J	CD11c	bone dendritic cells	KO	no	no	*Sirt*1 KO mice exhibit exacerbated lung pathology and dysregulated immune responses.	SIRT1 regulates mitochondrial function and immune homeostasis by controlling fatty acid synthesis, thereby modulating immune responses and limiting pathology during RSV infection.	[106]
*Sirt*1	RSV infection	C57BL/6J	CD11c	bone dendritic cells	KO	no	RSV infection	*Sirt*1 KO mice exhibit exacerbated lung pathology, increased viral load, and enhanced Th2 cytokine production.	SIRT1 promotes dendritic cell activation and autophagy-mediated processes during RSV infection, thereby directing efficient antiviral immune responses and limiting lung pathology.	[107]
*Sirt*1	tuberculosis	C57BL/6	no	myeloid cell	KO	no	acute and chronic tuberculosis infection	*Sirt*1 KO mice exhibit increased susceptibility to Mtb infection and enhanced inflammatory responses.	SIRT1 restricts Mtb growth by inducing autophagy and phagosome-lysosome fusion, and normalizes Mtb-induced inflammatory responses by deacetylating RelA/p65.	[108]
*Sirt*3	tuberculosis	C57BL/6J	no	global	KO	no	mycobacterium tuberculosis infection	*Sirt*3 KO mice exhibit increased mortality and higher bacterial burden.	SIRT3 regulates mitochondrial metabolism and redox homeostasis in macrophages, and its downregulation by mycobacterium tuberculosis promotes oxidative stress and cell death, contributing to disease progression.	[109]
*Sirt*3	Mabc infection	n/a	no	global	KO	no	Mabc infection	*Sirt*3 KO mice exhibit increased bacterial loads, exacerbated lung pathology, and enhanced inflammation	SIRT3 is essential for maintaining mitochondrial homeostasis and limiting oxidative stress and inflammation during Mabc infection, thereby contributing to host defense.	[110]
*Sirt*1	acute lung	C57	no	global	KO	no	CLP	*Sirt*1 KO mice exhibited exacerbated lung tissue damage, oxidative stress, and ferroptosis in sepsis-induced	SIRT1 regulates macrophage ferroptosis and oxidative stress via the Nrf2/HO-1 pathway, with its	[111]

Gene	Disease	Strains	Cre promoter	system specific	KO or OE	Inducible	Model background	Phenotype	Mechanism and conclusion	Reference
	injury							acute lung injury.	deficiency worsening sepsis-induced acute lung injury.	
*Sirt*3	acute lung injury	129S1/SvImJ	no	global	KO	no	endotoxin-induced ALI	*Sirt*3 KO mice exhibit exacerbated ALI with increased inflammation and mitochondrial dysfunction.	SIRT3 mitigates endotoxin-induced ALI by reducing mitochondrial ROS and inflammasome activation.	[112]
*Sirt*3	lung injury	ICR	no	global	KO	no	LPS	*Sirt*3 KO mice exhibited significantly exacerbated sepsis-induced ALI and lung cell injury.	SIRT3 is a crucial regulator in sepsis-induced ALI, exerting protective effects by inhibiting the NLRP3 inflammasome pathway.	[113]
*Sirt*3	CdCl2-induced COPD-like lung injury	C57BL/6	no	global	KO	no	CdCl2-induced alveolar epithelial senescence	*Sirt*3 KO mice exhibited exacerbated CdCl2-induced alveolar epithelial senescence, COPD-like alveolar structure damage, and lung function decline.	SIRT3 regulates mitochondrial function and alveolar epithelial senescence by deacetylating proteins like SOD2 and IDH2, with its deficiency worsening CdCl2-induced COPD-like lung injury.	[114]

Abbreviations: ALI, acute lung injury; CCL, C-C motif chemokine ligand; COPD, chronic obstructive pulmonary disease; FLIP, Flice-like inhibitory protein; FOXO3, forkhead box O3; IGF, insulin-like growth factor; LPS, lipopolysaccharide; MAPK, mitogen-activated protein kinase; Mabc, mycobacterium abscessus; Mtb, M. tuberculosis; MnSOD, manganese superoxide dismutase; n/a, not applicable; OE, overexpression; OGG1, 8-Oxoguanine DNA glycosylase 1; OVA,, ovalbumin; PPAR-γ, peroxisome proliferators-activated receptors-γ; ROS, reactive oxygen species; RSV, respiratory syncytial virus; *Sirt*, sirtuin; Th2, T helper type 2 cells; mtDNA, mitochondrial DNA; MO-AMs, monocyte-derived alveolar macrophages; KO, knockout; NF-κB, nuclear factor kappa-light-chain-enhancer of activated B cells.

Supplementary Table S5. Summary of phenotypes and mechanisms of *Sirt* genes in transgenic mice with digestive diseases

Gene	Disease	Strains	Cre promoter	system specific	KO or OE	Inducible	Model background	Phenotype	Mechanism and conclusion	Reference
*Sirt*1	non-alcoholic fatty liver	n/a	albumin	liver	KO	no	fasting	In *Sirt*1 KO mice, reduced liver and blood FGF21 levels were associated with decreased expression of genes involved in fatty acid oxidation and ketogenesis, while lipogenesis genes were upregulated.	SIRT1-mediated activation of FGF21 prevents liver steatosis caused by fasting.	[115]
*Sirt*2	non-alcoholic fatty liver	C57BL/6	no	global	KO	no	HFD-fed	*Sirt*2 KO mice exhibited absence of fat accumulation and elevated expression of genes associated with liver fibrosis.	SIRT2 has a critical role in regulating lipid metabolic homeostasis and in sustaining liver integrity by modulating related gene expression.	[116]
*Sirt*3	non-alcoholic fatty liver	129Sv	no	global	KO	no	no	*Sirt*3 KO mice demonstrated upregulated p-AMPK and p-ACC levels, as well as decreased phospho-p70S6k1 abundance.	SIRT3 negatively regulates autophagy via suppressing AMPK.	[117]
Sirt4	non-alcoholic fatty liver	C57BL/6NJ	no	global	KO	no	no	*Sirt*4 KO mice exhibit MCCC hyperacylation, reduced leucine catabolism, and increased insulin secretion.	SIRT4 is a lysine deacylase targeting MG-, HMG-, MGc-, and glutaryl-lysine modifications; its loss impairs leucine catabolism, elevating leucine to drive GDH and insulin secretion, leading to age-related insulin resistance.	[118]
Sirt4	non-alcoholic fatty liver	129/Sv; C57BL/6 (caloric restriction)	no	global	KO	no	adlibitum feeding; caloric restriction; HFD	*Sirt4* KO mice exhibit increased insulin secretion.	SIRT4 negatively regulates insulin secretion by ADP-ribosylating glutamate dehydrogenase, suggesting a potential role in modulating glucose homeostasis in conditions like diabetes.	[119]
Sirt4	non-alcoholic fatty liver	129/Sv	no	global	KO	no	fasted	*Sirt4* KO mice exhibitdecreased ATP levels in liver and muscle; increased AMPK phosphorylation; increased	SIRT4 regulates ATP homeostasis via ANT2; loss of SIRT4 activates AMPK-PGC1α retrograde signaling, promoting fatty acid	[120]

Gene	Disease	Strains	Cre promoter	system specific	KO or OE	Inducible	Model background	Phenotype	Mechanism and conclusion	Reference
								ACC phosphorylation; elevated PGC1α expression.	oxidation and mitochondrial biogenesis.	
Sirt4	non-alcoholic fatty liver	129/Sv	no	global	KO	no	fasted	*Sirt4* KO mice exhibit increased expression of PPARα target genes, enhanced fatty acid oxidation, increased NAD^+levels, mitochondrial elongation in periportal zone.	SIRT4 represses PPARα transcriptional activity and fatty acid oxidation by modulating NAD^+levels and SIRT1 activity. SIRT4 loss activates SIRT1, which binds PPARα and promotes lipid catabolism.	[121]
Sirt4	non-alcoholic fatty liver	129Sv × C57BL/6J	no	global	KO	no	fasted (6 h)/aged (up to 15 months)	*Sirt4* KO mice exhibit reduced body weight, elevated plasma glycerol, age-induced hyperglycemia and insulin resistance, and increased leucine-stimulated insulin secretion.	SIRT4 loss leads to accelerated age-induced insulin resistance across genetic backgrounds; elevated insulin secretion (glucose- and leucine-stimulated) may drive insulin resistance; genetic background influences leucine-stimulated insulin response.	[122]
Sirt5	non-alcoholic fatty liver	C57BL/6	No	Global	OE	no	Fed / 16h fasted / 24h fasted + refed	↑ liver SIRT5 mRNA; ↓ CPS1 acetylation; ↑ CPS1 activity; ↑ urea production in hepatocytes; no anatomical abnormalities	SIRT5 overexpression deacetylates and activates CPS1, enhancing urea cycle flux and ammonia detoxification; fasting-induced SIRT5 may contribute to adaptation.	[6]

Gene	Disease	Strains	Cre promoter	system specific	KO or OE	Inducible	Model background	Phenotype	Mechanism and conclusion	Reference
Sirt5	non-alcoholic fatty liver	C57BL/6NJ	No	Global	KO	no	Fed / 24h fasted / 1h refed	↑ liver GCDH glutarylation (especially fasted); ↓ GCDH tetramer stability and glutaryl-CoA oxidation; ↑ cellular amino acids	SIRT5 deglutarylates GCDH to restore activity; its loss causes hyperglutarylation, impairing lysine oxidation and leading to amino acid accumulation.	[123]
Sirt5	non-alcoholic fatty liver	129/Sv	No	Global	KO	no	no	Elevated PDC and SDH activity in KO cells; enhanced mitochondrial respiration; no change in complex IV	SIRT5 inhibits PDC and SDH via deacetylation; SIRT5 deficiency enhances respiration.	[124]
Sirt5	non-alcoholic fatty liver	C57BL/6J × 129 mixed	No	Global	KO	no	Coconut oil diet	Periportal steatosis; ↑ liver triglycerides; ↓ C12 oxidation; ↑ C8 oxidation and urine adipic acid; ↓ mitochondrial C12-CoA synthetase activity.	SIRT5 deficiency impairs C12 activation to C12-CoA, reducing C12 oxidation and causing lipid accumulation; compensatory peroxisomal/ω-oxidation insufficient.	[125]
Sirt5	non-alcoholic fatty liver	C57BL/6	Alb-Cre	Liver	OE	no	obese mice (5-7 months)	↓ global malonylation/succinylation; ↑ glycolysis & fatty acid oxidation; ↓ gluconeogenesis & liver triglycerides; ↑ serum TG; no change in body weight, glucose tolerance, RER	Hepatic SIRT5 OE improves glucose and lipid metabolism in ob/ob mice by demalonylating glycolytic/gluconeogenic enzymes and desuccinylating OXPHOS enzymes.	[126]

Gene	Disease	Strains	Cre promoter	system specific	KO or OE	Inducible	Model background	Phenotype	Mechanism and conclusion	Reference
*Sirt*6	non-alcoholic fatty liver	mixed C57BL/6J, 129/sv	albumin	liver	KO	no	western diet-induced NAFLD	*Sirt*6 KO mice showed more susceptible to diet-induced NAFLD.	SIRT6 controls hepatic lipogenesis by suppressing ChREBP and SREBP1.	[127]
*Sirt*6	non-alcoholic fatty liver	C57BL/6J	albumin	liver	KO	no	HFD-induced NAFLD	Specific *Sirt*6 KO mice exhibited exacerbated NAFLD.	SIRT6 protects against NAFLD progression by deacetylating ACSL5 in the cytoplasm, thereby promoting fatty acid oxidation and preventing lipid accumulation.	[128]
*Sirt*1	liver injury	C57BL/6J	albumin	liver	KO	no	liver IR injury	*Sirt*1 KO mice aggravated hepatic IR injury.	SIRT1 inhibits the downstream XBP1/NLRP3 inflammatory pathway by activating miR-182, thus alleviating hepatic IR injury in mice.	[129]
*Sirt*1	liver injury	C57BL/6J	albumin	liver	KO	no	liver fibrosis induced by carbon tetrachloride injection or alcohol feeding	*Sirt*1 KO mice exhibited more severe and persistent liver fibrosis with increased collagen deposition and activation of hepatic stellate cells.	SIRT1 plays a protective role in liver fibrosis by inhibiting the NLRP3 inflammasome and reducing inflammation, thereby preventing excessive extracellular matrix deposition and fibrosis progression.	[130]
*Sirt*1	liver injury	C57BL/6	albumin	liver	KO	no	D-galactosamine /lipopolysaccharide induced liver damage, mimicking septic liver injury and fulminant hepatitis	*Sirt*1 KO mice shows an enhanced NF-κB response, resulting in protection from liver damage.	Inactivation of SIRT1 in mouse livers protects against endotoxemic liver injury by acetylating and activating NF-κB.	[131]
*Sirt*2	liver injury	C57BL/6J	no	global	KO	no	hepatic I/R injury	*Sirt*2 KO mice show reduced necrotic and apoptotic areas, along with lower AST and ALT levels, indicating attenuated hepatocellular injury.	SIRT2 exacerbates hepatic I/R injury by deacetylating and inhibiting MAPK phosphatase-1, thereby promoting MAPK activation.	[132]

Gene	Disease	Strains	Cre promoter	system specific	KO or OE	Inducible	Model background	Phenotype	Mechanism and conclusion	Reference
*Sirt*3	liver injury	C57BL/6	no	global	KO	no	acute radiation-induced liver injury	*Sirt*3 KO mice exhibit increased oxidative stress and sensitivity to radiation-induced liver injury.	SIRT3 regulates mitochondrial oxidative metabolism and antioxidant defenses, protecting against radiation-induced tissue damage by controlling superoxide levels.	[133]
*Sirt*3	liver injury	129S1/SvImJ	no	global	KO	no	liver fibrosis induced by intraperitoneal injections of carbon tetrachloride	*Sirt*3 KO mice exhibited exacerbated liver fibrosis and inflammation.	SIRT3 plays a protective role in liver fibrosis by suppressing inflammation.	[134]
*Sirt*3	liver injury	B6/Sv129	no	global	KO	no	radiation-induced liver injury	*Sirt*3 KO livers showed increased inflammation, bile duct loss, DNA damage , and protein oxidation.	SIRT3 deficiency may promote IR-induced long-term persistent liver injury via hydrogen peroxide and hydroperoxide sensitive signaling cascades.	[135]
*Sirt*6	liver injury	n/a	albumin	liver	KO	no	mice received BDL surgery or a 0.1% DDC diet to induce cholestatic liver injury	*Sirt*6 KO mice exhibit exacerbated liver injury and fibrosis in response to cholestatic stress.	SIRT6 protects against cholestatic liver injury by deacetylating and destabilizing ERRγ, thereby inhibiting CYP7A1 expression and reducing bile acid synthesis.	[136]
*Sirt*6	liver injury	C57BL/6J	albumin	liver	KO;OE	no	acetaminophen overdose and bile duct ligation to induce acute liver failure	Hepatic-specific *Sirt*6 KO mice exhibited exacerbated liver damage in acute liver failure models, while *Sirt*6 OE protected against APAP-induced hepatotoxicity and improved survival rates.	SIRT6 protects against acute liver failure by reducing oxidative stress, inflammation, and cell death through mechanisms involving Nrf2/HO-1 activation and PARP1 inhibition.	[137]
*Sirt*7	liver injury	C57BL/6J	LysM	myeloid cell	KO	no	chronic liver fibrosis induced by carbon tetrachloride	Specific *Sirt*7 KO mice exhibited exacerbated liver fibrosis and increased stellate cell activation after CCl4 treatment.	SIRT7 protects against liver fibrosis by suppressing stellate cell activation via deacetylation and inhibition of the TGF-β/SMAD2/3 signaling pathway.	[138]

Gene	Disease	Strains	Cre promoter	system specific	KO or OE	Inducible	Model background	Phenotype	Mechanism and conclusion	Reference
*Sirt*1	inflammatory bowel disease	C57BL/6	villin	intestinal epithelial	KO	no	DSS-induced colitis	*Sirt*1 KO mice exhibit increased intestinal inflammation and altered gut microbiota with age, leading to enhanced susceptibility to colitis.	SIRT1 maintains intestinal tissue homeostasis, and its deficiency exacerbates intestinal inflammation and susceptibility to colitis by disrupting the balance of gut microbiota.	[139]
*Sirt*1	inflammatory bowel disease	C57BL/6	bovine keratin 5 promoter	epithelial cells	OE	no	DSS-induced colitis	*Sirt*1 OE mice exhibit attenuated symptoms of DSS-induced colitis, including reduced weight loss, less severe rectal bleeding, and decreased colon shortening.	SIRT1 plays a protective role in maintaining intestinal epithelial barrier integrity by stabilizing b-TrCP1 to degrade Snail1, thereby upregulating the expression of cell adhesion proteins such as E-cadherin, Occludin, and Claudin-1.	[140]
*Sirt*1	inflammatory bowel disease	C57BL/6	na	smooth muscle cell	OE	no	DSS-induced colitis	*Sirt*1 OE mice exhibited abnormal baseline intestinal architecture with decreased goblet cells and impaired colonic epithelium.	SIRT1 in smooth muscle cells negatively regulates colonic epithelial regeneration by releasing cZFP609, which inhibits HIF-1α nuclear translocation and impairs mucosal repair in colitis.	[141]
*Sirt*2	inflammatory bowel diseas	C57BL/6J	CMV	global	KO	no	DSS-induced colitis	*Sirt*2 KO mice displayed more severe clinical and histological manifestations.	SIRT2 acts as a suppressor of inflammation in colitis by inhibiting NF-κB activation and promoting M2 macrophage polarization through deacetylation.	[142]
*Sirt*2	inflammatory bowel diseas	C57BL/6N	no	global	KO	no	intestinal stress induced by chronic cold exposure	*Sirt*2 KO mice exhibited reduced intestinal permeability and enhanced autophagy.	SIRT2 regulates intestinal barrier function by modulating autophagy and ER stress through FoxO1 acetylation, SIRT2 KO alleviates ileal injury via enhanced autophagy under cold exposure	[143]
*Sirt*2	inflammatory bowel diseas	B6.129	no	global	KO	no	no	*Sirt*2 KO showed decreased intestinal enterocyte and goblet cell differentiation but increased the Paneth cell lineage and increased proliferation of IECs.	SIRT2 plays a protective role in maintaining intestinal homeostasis by inhibiting Wnt-β-catenin signaling.	[144]

Gene	Disease	Strains	Cre promoter	system specific	KO or OE	Inducible	Model background	Phenotype	Mechanism and conclusion	Reference
*Sirt*6	inflammatory bowel diseas	C57BL/6J	villin	intestinal epithelial	KO	no	DSS-induced colitis	*Sirt*6 KO mice exhibited susceptibility to DSS-induced colitis.	SIRT6 protects intestinal epithelial cells against inflammatory injury by preserving R-spondin-1levels in the colonic cells.	[145]
*Sirt*6	inflammatory bowel diseas	C57BL/6	RAR-related orphan receptor gamma	(produce no mature T cells or B cells)	KO	no	Citrobacter rodentium infection and DSS-induced colitis	*Sirt*6 KO mice exhibited enhanced IL-22 production by innate lymphoid cells and improved resistance to colitis.	SIRT6 negatively regulates IL-22 production in group 3 innate lymphoid cells by a cell-intrinsic mechanism, and its deletion enhances resistance to bacterial infection and colitis.	[146]
*Sirt*4	SAP	C57BL/6	no	global	KO	no	L-arginine induced SAP	*Sirt*4 KO mice showed serious pancreatic tissue injury and related lung and kidney injury.	SIRT4 plays a protective role in SAP by regulating the HIF-1α/HO-1 pathway to inhibit ferroptosis.	[147]

Abbreviation: AMPK, AMP - activated protein kinase; ALT, Alanine aminotransferase; AST, Aspartate aminotransferase; ATGL, adipose triglyceride lipase; BA,bile acid; ChREBP, Carbohydrate - responsive element - binding protein; CR, calorie restriction; CYP7A1, Cytochrome P450 family 7 subfamily A member 1; DSS, dextran sulfate sodium;EBP1,Eukaryotic initiation factor 4 - gamma - binding protein 1; ERRγ, Estrogen - related receptor gamma;FGF21, Fibroblast growth factor 21; IEC, intestinal epithelial cell;IR, Insulin resistance; KO, knockout; MAPK, Mitogen - activated protein kinase; NAFLD, Non - alcoholic fatty liver disease; NLRP3, NACHT, LRR and PYD domains - containing protein 3; NF - κB,Nuclear factor - kappa B; Nrf2, Nuclear factor erythroid 2 - related factor 2; OE, overexpression; p - ACC, phosphorylated Acetyl - CoA carboxylase;SAP, severe acute pancreatitis;SRALT, Sterol - regulated acyl - CoA lyase - like transmembrane protein;XBP1,X - box - binding protein 1; n/a,not applicable.

Supplementary Table S6. Summary of phenotypes and mechanisms of *Sirt* genes in transgenic mice with neuropsychiatric diseases

Gene	Disease	Strains	Cre promoter	system specific	KO or OE	Inducible	Model background	Phenotype	Mechanism and conclusion	Reference
*Sirt*1	PD	C57BL/6J	no	global	KO (exon 4 mutant)	no	MPTP-induced PD; hm2α-SYN-39 transgenic PD mouse model	*Sirt*1 KO mice showed worsened motor behavior phenotype, but no significant differences in anxiety or depression-like behaviors.	SIRT1 protects against neurodegeneration in Parkinson's disease by inhibiting the CDK5-mediated ubiquitin-proteasome pathway.	[148]
*Sirt*3	PD	C57BL/6J	CRISPR-Cas	global	KO	no	MPTP-induced PD	*Sirt*3 KO mice exhibited more sensitivity and dopaminergic neuronal loss in MPTP treatment.	SIRT3 protects against dopaminergic neuronal death in PD by directly deacetylating SOD2 and ATP synthase β.	[149]
*Sirt*3	PD	n/a	no	global	KO	no	MPTP-induced PD; DJ-1 KO mouse model developing autosomal recessive early onset PD	*Sirt*3 KO mice exhibited increased dopaminergic neuronal loss in MPTP-induced PD models and greater oxidative stress and degeneration in DJ-1 KO models.	SIRT3 protects dopaminergic neurons in Parkinson's disease by deacetylating MnSOD on K68 to reduce mitochondrial oxidative stress.	[150]
*Sirt*5	PD	C57BL/6	no	global	KO	no	MPTP-induced PD	*Sirt*5 KO mice showed normal behavior but exhibited more severe motor deficits and dopaminergic neuron.	SIRT5 protects against dopaminergic neuron loss in PD by preserving mitochondrial antioxidant capacity.	[151]
*Sirt*2	PD	C57BL/6	no	global	KO	no	MPTP-induced PD	*Sirt*2 KO mice show reduced MPTP-induced nigrostriatal damage.	SIRT2 promotes neurodegeneration in PD by deacetylating Foxo3a and activating Bim to induce apoptosis.	[152]
*Sirt*2	PD	C57BL/6J	no	global	KO	no	brain-specific Cdk5 KO mice mimicking PD	*Sirt*2 KO mice exhibit reduced dopaminergic neuron loss and improved behavioral phenotypes.	SIRT2 promotes neuronal death in PD by translocating to the nucleus after Cdk5-mediated phosphorylation.	[153]
*Sirt*1	AD	n/a	no	global	KO (*Sirt*1-null)	no	no	*Sirt*1 KO mice exhibit impaired cognitive function and synaptic plasticity.	SIRT1 maintains normal cognitive function and synaptic plasticity by regulating chromatin remodeling, gene expression, and signaling pathways like ERK1/2, and supports neuronal dendritic complexity.	[154]
*Sirt*2	AD	C57BL/6J	no	global	KO	no	no	*Sirt*2 KO mice exhibit resistance to neurodegeneration and improved cognitive function	SIRT2 is a microtubule deacetylase that contributes to neurodegeneration by destabilizing microtubules and impairing autophagy, promoting tau phosphorylation and Aβ oligomer accumulation.	[155]

Gene	Disease	Strains	Cre promoter	system specific	KO or OE	Inducible	Model background	Phenotype	Mechanism and conclusion	Reference
*Sirt*2	AD	C57BL/6	no	global	KO	no	AppPs1 mutant transgenic AD mice	*Sirt*2 KO improves cognitive deficits and reduces Aβ pathology.	SIRT2 exerts a detrimental role in AD by deacetylating APP at lysines 132 and 134, promoting amyloidogenic processing and Aβ production.	[156]
*Sirt*3	AD	C57BL/6J	no	global	KO (heterozygous)	no	AppPs1 mutant transgenic AD mice	*Sirt*3 KO mice exhibit early epileptiform EEG activity, seizures, and premature death, with a significant loss of GABAergic interneurons in the cerebral cortex and associated neuronal network hyperexcitability.	SIRT3 protects GABAergic interneurons and suppresses neuronal network hyperexcitability by enhancing mitochondrial function, and its reduction exacerbates AD-related pathology.	[157]
*Sirt*1	HD	C57BL/6	no	global	OE	no	N171-82Q mice mimicking HD symptoms	*Sirt*1 OE improves motor function, reduces brain atrophy, enhances BDNF signaling, and ameliorates metabolic abnormalities.	SIRT1 enhances neuronal survival and function by deacetylating key substrates like FOXO3a, boosting BDNF levels, and activating TrkB signaling, thereby protecting against neurodegeneration and metabolic dysfunction.	[158]
*Sirt*2	HD	CBAxC57BL/6 F1	no	global	KO	no	R6/2 mouse model of HD	*Sirt*2 KO mice exhibit no significant impact on tubulin acetylation, cholesterol biosynthesis, or HD progression	SIRT2 inhibition does not modify disease progression in the R6/2 mouse model of HD	[159]
*Sirt*1	depression	C57BL/6J	emx1-ires	forebrain excitatory neurons	KO	no	no	Selectively *Sirt*1 KO causes male mice to display depression-like behaviors.	In depression, SIRT1 enhances neuronal excitability and synaptic transmission by deacetylating substrates such as PGC-1α, thereby promoting mitochondrial biogenesis and alleviating depressive behaviors.	[160]
*Sirt*2	depression	C57BL/6	no	global	KO	no	social defeat stress-induced depression mouse model	*Sirt*2 KO mice exhibit blocked development of social defeat stress-induced depressive-like behavior.	SIRT2 promotes depressive-like behaviors by translocating into the nucleus of amygdala neurons upon CDK5-mediated phosphorylation at serine residues 368 and 372.	[161]
*Sirt*6	depression/ anxiety	C57BL/6	hGFAP	astrocyte	KO; OE	no	chronic unpredictable mild stress model	*Sirt*6 KO mice do not spontaneously develop depression-like behaviors, reexpression of SIRT6 in astrocytes reverses anti-depressant-like effects.	SIRT6 in astrocytes modulates depression and anxiety behaviors by regulating purine metabolism and cAMP levels in the medial prefrontal cortex.	[162]
*Sirt*1	anxiety	C57BL/6J (inferred)	nestin	brain	KO/OE	no	MPTP-induced PD	Specific *Sirt1* KO mice exhibit less anxious, enhanced exploratory drive, resistant to depression-like behaviors.	SIRT1 deacetylates transcription factor NHLH2 at K49, activating MAO-A transcription → reduces serotonin levels	[163]

Gene	Disease	Strains	Cre promoter	system specific	KO or OE	Inducible	Model background	Phenotype	Mechanism and conclusion	Reference
								SIRT1 OE mice: more anxious, reduced exploration, susceptible to depression.	→ increases anxiety and decreases exploratory drive. Human SNPs in SIRT1 are associated with anxiety disorders.	
*Sirt*1	anxiety	C57BL/6J	D1-Cre	D1 receptor-expressing medium spiny neurons	KO	no	chronic social defeat stress	*Sirt*1 KO reduces susceptibility to social defeat stress and anxiety-like behaviors.	SIRT1 in D1-MSNs regulates synaptic gene expression, influencing excitatory and inhibitory neurotransmission, and contributes to depression- and anxiety-like behaviors through modulating neural activity.	[164]
*Sirt*1	Injury	C57BL/6J	no	global	OE	no	traumatic injury	*Sirt*1 OE in mice mitigates visual dysfunction and retinal ganglion cell loss following traumatic brain injury.	*Sirt*1 exerts a protective role in traumatic optic neuropathy by promoting retinal ganglion cell survival and preserving visual function.	[165]
*Sirt*3	Injury	C57BL/6	Map2-Cre ERT2	neuron	KO	tamoxifen,	FNDC5 KO mice;traumatic injury	*Sirt*3 KO mice exhibited exacerbated mitochondrial damage, oxidative stress, and neuronal apoptosis after traumatic brain injury.	FNDC5/irisin exerts a protective role against acute brain injury by promoting SIRT3-dependent mitochondrial quality control.	[166]
*Sirt*1	Injury	C57BL/6J	Tie2-Cre	endothelial cells	KO	no	spinal cord injury	*Sirt*1 KO exacerbates blood-spinal cord barrier disruption, inflammation, neural cell death, and impairs functional recovery after spinal cord injury.	SIRT1 exerts a protective role in spinal cord injury by attenuating blood-spinal cord barrier disruption through deacetylating p66Shc and reducing oxidative stress.	[167]

Abbreviations: AD, Alzheimer's disease; AKT, protein kinase B; BDN, brain-derived neurotrophic factor; BDNF, brain-derived neurotrophic factor; CDK5, cyclin-dependent kinase 5; EEG, electroencephalogram; ERK, extracellular signal-regulated kinase; FOXO, forkhead box O; GSK-3β, glycogen synthase kinase 3 beta; HD, Huntington's disease; KO, knockout; MPT, mitochondrial permeability transition; MPTP, 1-methyl-4-phenyl-1,2,3,6-tetrahydropyridine hydrochloride; MnSOD, manganese superoxide dismutase; OE, overexpression; PD, Parkinson's disease; *Sirt*, sirtuin; SOD2, superoxide dismutase 2

Supplementary Table S7. Summary of phenotypes and mechanisms of *Sirt* genes in transgenic mice with endocrine-related metabolic disorders

Gene	Disease	Strains	Cre promoter	system specific	KO or OE	Inducible	Model background	Phenotype	Mechanism and conclusion	Reference
*Sirt*1	DM	mixed 129/FVB/Black Swiss; C57BL/6	albumin	liver	KO	no	no	*Sirt*1 KO mice exhibit hyperglycemia, oxidative damage, and insulin resistance.	SIRT1 protects against hyperglycemia and insulin resistance by promoting mTORC2/Akt signaling and inhibiting gluconeogenesis in the liver.	[168]
*Sirt*1	DM	C57BLKS	podocin	podocytes	KO	no	db/db mice	*Sirt*1 KO mice exhibit increased oxidative stress and mitochondrial dysfunction, leading to various metabolic disorders.	SIRT1 protects against diabetic kidney disease by deacetylating and inhibiting the transcriptional activity of NF-κB and STAT3, reducing inflammation and oxidative stress in podocytes.	[169]
*Sirt*2	DM	C57BL/6J	no	global	KO	no	HFD	*Sirt*2 KO mice exhibit increased weight gain, insulin resistance in skeletal muscle, and exacerbated hepatic insulin resistance.	SIRT2 protects against obesity-induced insulin resistance by regulating mitochondrial protein acetylation and inflammation.	[170]
*Sirt*2	DM	C57BL/6J	no	global	KO	no	metabolic stress induced by intermittent cold exposure	*Sirt*2 KO mice exhibited impaired glucose tolerance, insulin resistance, and increased susceptibility to obesity and hepatic metabolic dysfunction.	SIRT2 plays a protective role in metabolic diseases by regulating glucose metabolism through deacetylation of PKM2, reducing glycolysis and maintaining metabolic homeostasis.	[171]
*Sirt*3	DM	129 Sv	no	global	KO	no	chronic HFD	*Sirt*3 KO mice exhibit increased vulnerability to oxidative stress and impaired pancreatic beta cell function.	SIRT3 acts as a mitochondrial deacetylase to protect pancreatic beta cells from oxidative stress-induced damage, suggesting its potential role in mitigating beta cell dysfunction in type 2 diabetes.	[172]
*Sirt*3	DM	C57BL/6J	adiponectin	adipose	KO	no	cold exposure	*Sirt*3 KO mice exhibit impaired thermogenesis and lipid accumulation in interscapular brown adipose tissue during cold exposure, along with increased body weight and metabolic dysfunction.	SIRT3 plays a protective role in aging-related metabolic disorders by regulating acylcarnitine metabolism via the HIF1α-PPARα signaling pathway to maintain thermogenesis in brown adipose tissue.	[173]
*Sirt*6	DM	C57BL/6J	LysM	macrophages; myeloid	KO	no	cold exposure; HFD	*Sirt*6 KO mice exhibited obesity and insulin resistance due to impaired thermogenesis in brown	SIRT6 protects against obesity and metabolic disorders by enhancing thermogenesis and energy expenditure	[174]

Gene	Disease	Strains	Cre promoter	system specific	KO or OE	Inducible	Model background	Phenotype	Mechanism and conclusion	Reference
								adipose tissue.	in brown adipose tissue through the regulation of norepinephrine levels via H3K9 acetylation.	
*Sirt*6	DM	C57BL/6	creatine kinase muscle	muscle	KO	no	HFD-induced obesity	Specific *Sirt*6 KO mice exhibit impaired glucose homeostasis and insulin sensitivity.	SIRT6 regulates metabolic homeostasis in skeletal muscle through activation of AMPK, suggesting its potential role in treating type 2 diabetes	[175]
*Sirt*4	DM	129/Sv; C57BL/6 (caloric restriction)	no	global	KO	no	ad libitum feeding; caloric restriction; HFD	*Sirt*4 KO mice exhibit increased insulin secretion.	SIRT4 negatively regulates insulin secretion by ADP-ribosylating glutamate dehydrogenase, suggesting a potential role in modulating glucose homeostasis in conditions like diabetes.	[24]
Sirt4	DM	C57BL/6NJ	no	global	KO	no	No	*Sirt4* KO mice exhibit MCCC hyperacylation, reduced leucine catabolism, and increased insulin secretion.	SIRT4 is a lysine deacylase targeting MG-, HMG-, MGc-, and glutaryl-lysine modifications; its loss impairs leucine catabolism, elevating leucine to drive GDH and insulin secretion, leading to age-related insulin resistance.	[118]
Sirt4	DM	129Sv × C57BL/6J	no	global	KO	no	fasted (6 h)/aged (up to 15 months)	*Sirt4* KO mice exhibit reduced body weight, elevated plasma glycerol, age-induced hyperglycemia and insulin resistance, and increased leucine-stimulated insulin secretion.	SIRT4 loss leads to accelerated age-induced insulin resistance across genetic backgrounds; elevated insulin secretion (glucose- and leucine-stimulated) may drive insulin resistance; genetic background influences leucine-stimulated insulin response.	[122]

Gene	Disease	Strains	Cre promoter	system specific	KO or OE	Inducible	Model background	Phenotype	Mechanism and conclusion	Reference
Sirt4	DM	C57BL/6J	MIP-Cre ERT1Lphi	pancreatic β-cell	KO	no	fasted (6 h) / aged (3 months and 12 months)	β-cell-specific*Sirt4* KO mice exhibit normal body weight; normal glucose tolerance; normal glucose-stimulated insulin secretion; normal leucine-stimulated insulin secretion ; no age-induced hyperinsulinemia or glucose intolerance.	β-cell-specific SIRT4 ablation does not recapitulate the insulin hypersecretion phenotype of global SIRT4KO mice; SIRT4 regulates insulin secretion via extra-islet tissues rather than directly in β-cells.	[176]
Sirt4	DM	C57BL/6	Adipoq Cre	adipose tissue	KO	no	normal fed	*Sirt*4 KO mice exhibit elevated BCAA levels in adipose tissue and plasma.	SIRT4 promotes BCAA catabolism in adipose tissue in vivo; loss of SIRT4 impairs BCAA oxidation, leading to tissue and systemic BCAA accumulation.	[177]
*Sirt*7	DM	C57BL/6J	no	global	KO	no	no	*Sirt*7 KO mice exhibit extended lifespan, improved glucose tolerance, and increased serum FGF21 levels in aged male mice.	SIRT7 deficiency protects against aging-associated metabolic dysfunction and extends lifespan in male mice by maintaining high levels of FGF21, which improves glucose metabolism and insulin sensitivity.	[178]
*Sirt*7	DM	C57BL/6J	adipoq-Cre; Ucp1-Cre	global; adipose tissue; brown adipose tissue	KO	no	no	*Sirt*7 KO mice exhibit increased energy expenditure and thermogenesis due to enhanced brown adipose tissue function.	SIRT7 acts as an energy-saving factor by suppressing brown adipose tissue thermogenesis through deacetylating IGF2BP2 to inhibit Ucp1 mRNA translation.	[179]
*Sirt*1	hyperlip idemia	C57BL/6	albumin	liver	KO	no	HFD	Specific *Sirt*1 KO mice develop hepatic steatosis, inflammation, and endoplasmic reticulum stress.	SIRT1 plays a crucial role in regulating hepatic lipid metabolism by positively regulating PPARα signaling, and its activation may be beneficial for preventing obesity-associated metabolic diseases.	[180]

Gene	Disease	Strains	Cre promoter	system specific	KO or OE	Inducible	Model background	Phenotype	Mechanism and conclusion	Reference
Sirt4	hyperlip idemia	129/Sv	no	global	KO	no	fasted	*Sirt4* KO mice exhibit increased expression of PPARα target genes, enhanced fatty acid oxidation, increased NAD^{+}levels, mitochondrial elongation in periportal zone.	SIRT4 represses PPARα transcriptional activity and fatty acid oxidation by modulating NAD^{+}levels and SIRT1 activity. SIRT4 loss activates SIRT1, which binds PPARα and promotes lipid catabolism.	[121]
Sirt4	hyperlip idemia	129/Sv	no	global	KO	no	fasted	*Sirt4* KO mice exhibit decreased ATP levels in liver and muscle; increased AMPK phosphorylation; increased ACC phosphorylation; elevated PGC1α expression.	SIRT4 regulates ATP homeostasis via ANT2; loss of SIRT4 activates AMPK-PGC1α retrograde signaling, promoting fatty acid oxidation and mitochondrial biogenesis.	[120]
*Sirt*6	hyperlip idemia	C57BL／6J	no	global	OE	no	HFD	*Sirt4 OE* mice on HFD exhibit ↓visceral fat, ↓ LDL-cholesterol, ↓triglycerides, ↓free fatty acids; no change in food intake or energy expenditure；↑glucose tolerance and ↑glucose-stimulated insulin secretion ; no change in insulin tolerance .	SIRT6 OE protects against diet-induced obesity and dyslipidemia by downregulating PPARγ target genes involved in lipid storage and clearance.	[8]

Abbreviation: ADP, adenosine diphosphate; AMPK, AMP-activated protein kinase; Akt, protein kinase B; DM, diabetes mellitus; HFD, high-fat diet; mTOR, mammalian target of rapamycin; NF-κB, nuclear factor kappa-light-chain-enhancer of activated B cells; OE, overexpression; PPARα, peroxisome proliferator-activated receptor alpha; SIRT, sirtuin; STAT3, signal transducer and activator of transcription 3; KO, knockout

Supplementary Table S8. Summary of phenotypes and mechanisms of *Sirt* genes in transgenic mice with genitourinary system diseases

Gene	Disease	Strains	Cre promoter	system specific	KO or OE	Inducible	Model background	Phenotype	Mechanism and conclusion	Reference
*Sirt*3	Ischaemia-reperfusion-induced AKI	C57BL/6J	no	global	KO	no	IR-induced AKI	*Sirt*3 KO mice, mitochondrial dynamics are disturbed, with excessive fission, impaired fusion and early fibrosis after IRI	SIRT3 primarily protects tubular epithelial cells by stabilising mitochondrial function and limiting oxidative bursts.	[181]
*Sirt*7	ischaemia-reperfusion-induced AKI	C57BL/6	no	global	KO	no	IR-induced AKI	*Sirt*7 KO reduces inflammation and tubular damage in AKI.	SIRT7 promotes renal inflammation and tubular damage in AKI by enhancing NFκB signaling through p65 activation.	[182]
*Sirt*2	infection induced AKI	C57BL/6J	no	global	KO	no	tubular injury caused by LPS	*Sirt*2 KO mice exhibited decreased renal CXCL2 and CCL2 expression, reduced neutrophil and macrophage infiltration, and attenuated acute tubular injury and renal dysfunction.	SIRT2 promotes renal inflammatory injury by regulating CXCL2 and CCL2 expression through the MKP-1-MAPK signaling pathway and p65 binding to their promoters.	[183]
*Sirt*3	infection induced AKI	C57BL/6	no	global	KO	no	sepsis-induced acute kidney injury	*Sirt*3 KO mice exhibited exacerbated kidney dysfunction, increased mitochondrial alterations, and enhanced ROS production.	SIRT3 protects against sepsis-induced AKI by attenuating ROS production, inhibiting the NLRP3 inflammasome, and reducing oxidative stress and apoptosis.	[184]
*Sirt*3	infection induced AKI	C57BL/6	no	global	KO	no	LPS induced AKI	*Sirt*3 KO mice exhibited exacerbated mitochondrial damage, apoptosis, and renal pathological injury in sepsis-induced AKI.	SIRT3 protects against LPS-induced mitochondrial damage and apoptosis in renal tubular epithelial cells by deacetylating YME1L1 and promoting OPA1-mediated mitochondrial fusion.	[185]
*Sirt*1	drug- and contrast-induced AKI	C57BL/6	no	global	KO (*Sirt*1ΔE4, heterozygous)	no	cisplatin-induced AKI	*Sirt*1 KO mice exhibit exacerbated renal damage and increased susceptibility to AKI following cisplatin exposure..	SIRT1 acts as a crucial mediator in renal aging and AKI susceptibility by regulating the JNK signaling pathway via deacetylation of DUSP16, protecting against renal injury.	[186]
*Sirt*1	drug- and contrast-induced AKI	C57BL/6	no	global	KO (*Sirt*1ΔE4, heterozygous)	no	hypoxic conditions	*Sirt*1 KO mice exhibit increased mitochondrial oxidative damage, impaired autophagy, and enhanced apoptosis .	SIRT1 promotes cell adaptation to hypoxia by enhancing autophagy and inhibiting apoptosis through the deacetylation of Foxo3, which is crucial for protecting against age- and hypoxia-related tissue	[187]

Gene	Disease	Strains	Cre promoter	system specific	KO or OE	Inducible	Model background	Phenotype	Mechanism and conclusion	Reference
									damage.	
*Sirt*3	drug- and contrast-induced AKI	129S1/SvImJ	no	global	KO	no	contrast-induced AKI	*Sirt*3 KO mice exhibited aggravated renal dysfunction and histological injury in a CIAKI model, with increased oxidative stress and apoptosis.	SIRT3 plays a protective role in CIAKI by mitigating oxidative stress and apoptosis, and its deficiency exacerbates renal injury through increased ROS production and apoptotic pathways.	[188]
*Sirt*5	drug- and contrast-induced AKI	B6;129-*Sirt*5tm1Fwa/J	no	global	KO	no	a renal IRI and cisplatin-induced AKI	*Sirt*5 KO mice exhibit improved kidney function and reduced tissue damage compared with WT mice.	SIRT5 regulates the balance of mitochondrial versus peroxisomal fatty acid oxidation in proximal tubular epithelial cells, protecting against acute kidney injury by enhancing peroxisomal function and reducing mitochondrial oxidative stress.	[189]
*Sirt*6	drug- and contrast-induced AKI	129Sv/ C57BL6	no	global	OE, 4-fold	no	cisplatin-induced AKI	*Sirt*6 OE mice exhibit attenuated renal dysfunction, inflammation, and apoptosis .	SIRT6 protects against cisplatin-induced kidney injury by inhibiting ERK1/2 signaling, thereby reducing inflammation and apoptosis.	[190]
*Sirt*6	drug- and contrast-induced AKI	C57BL/6	no	global	KO	no	cisplatin-induced AKI	*Sirt*6 KO mice exhibited exacerbated kidney injury and ferroptosis in cisplatin-induced AKI.	SIRT6 protects against cisplatin-induced AKI by inhibiting ferroptosis through transcriptional repression of BAP1 via H3K9 acetylation.	[191]
*Sirt*2	drug- and contrast-induced AKI	C57BL/6J	no	global	KO	no	cisplatin-induced AKI	*Sirt*2 KO mice exhibit improved kidney function, reduced renal injury, and increased survival.	SIRT2 exacerbates cisplatin-induced renal injury by regulating MKP-1 expression and MAPK signaling, which promotes inflammation and apoptosis.	[192]
*Sirt*7	drug- and contrast-induced AKI	C57/BL6J	no	global	KO	no	cisplatin-induced AKI	*Sirt*7 KO mice exhibited resistance to cisplatin-induced AKI, with decreased expression of pro-inflammatory cytokines and reduced renal damage.	SIRT7 plays a detrimental role in cisplatin-induced AKI by promoting inflammation through the regulation of NF-κB p65 nuclear translocation and TNF-α expression.	[193]
*Sirt*1	obstruction induced acute kidney injury	C57BL/6J	Tenascin-C	renal interstitial cell	KO	no	UUO-induced renal fibrosis	*Sirt*1 KO exacerbates renal fibrosis and damage in UUO model mice.	SIRT1 protects against renal fibrosis by inhibiting HIF-2α expression and activity.	186
*Sirt*1	renal fibrosis	B6;129	Tie2	endothelial	KO	no	folic acid-induced tubulointerstitial fibrosis	*Sirt*1 KO mice exhibit impaired angiogenesis, increased vascular senescence, and exaggerated fibrotic response to nephrotoxic injury.	Endothelial SIRT1 deficiency exacerbates nephrosclerosis by downregulating MMP-14, leading to impaired matrix degradation and fibrosis.	187

Gene	Disease	Strains	Cre promoter	system specific	KO or OE	Inducible	Model background	Phenotype	Mechanism and conclusion	Reference
*Sirt*6	obstruction induced acute kidney injury	C57/BL6	Ggt1	kidney proximal tubule	KO	no	UUO-induced renal injuries and fibrosis	Loss of proximal tubule *Sirt*6 exacerbates UUO-induced renal tubulointerstitial inflammation and fibrosis.	SIRT6 in proximal tubules protects against renal fibrosis by regulating β-catenin acetylation and ECM protein promoter transcription.	184
*Sirt*1	diabetic nephropathy	female C57BL/6	no	global	OE	no	kidney disorders in male offspring due to maternal high-fat diet	*Sirt*1 OE in offspring mitigates renal lipid accumulation, oxidative stress, and inflammation induced by maternal high-fat diet, but has limited effects on fibrosis and albuminuria.	SIRT1 exerts protective effects against kidney disorders by promoting lipid catabolism, reducing oxidative stress, and attenuating inflammation through modulating downstream pathways such as PGC-1α and antioxidant enzymes.	[194]
*Sirt*1	diabetic nephropathy	C57BL/6J	γGT	tubule-specific	KO	no	streptozotocin-induced diabetic mice; obese-type diabetic mice	*Sirt*1 KO mice exhibited increased albuminuria and higher claudin-1 expression in podocytes.	SIRT1 in proximal tubules protects against diabetic albuminuria by maintaining NMN concentrations and suppressing claudin-1 OE in podocytes through epigenetic regulation.	[195]
*Sirt*1	diabetic nephropathy	C57BL/6; 129	NPHS2	podocyte	KO	Dox	Aging-induced glomerulosclerosis and albuminuria	*Sirt*1 KO in podocytes exacerbates glomerulosclerosis, albuminuria, and oxidative stress in aging mice.	SIRT1 in podocytes protects against aging-induced kidney injury by regulating oxidative stress, cellular senescence, and transcription factors PGC1α, FOXO3, FOXO4, and NF-κB.	[196]
*Sirt*2	diabetic nephropathy	n/a	no	global	KO	no	no	*Sirt*2 KO mice exhibited alleviated renal injury and inflammatory response in HFD/STZ-induced diabetic nephropathy.	SIRT2 exacerbates renal inflammation and injury in diabetic nephropathy by deacetylating c-Jun/c-Fos, promoting AP-1 activity and downstream inflammatory gene transcription.	[197]
*Sirt*3	AKI	B6; 129	no	global	KO	no	cisplatin-induced AKI	*Sirt*3 KO mice exhibit more severe AKI and fail to respond to UC-MSC treatment.	SIRT3 protects against AKI by regulating mitochondrial function and microtubule dynamics, thereby promoting renal repair.	[198]
*Sirt*6	kidney injury	C57BL/6J	NPHS2	podocyte	KO	no	angiotensin-induced cholesterol accumulation and injury	*Sirt*6 KO mice exhibit exacerbated kidney injury and cholesterol accumulation in podocytes.	SIRT6 protects against angiotensin II-induced podocyte injury by regulating cholesterol efflux via ABCG1, highlighting its potential as a therapeutic target in chronic kidney disease.	[199]
*Sirt*6	CKD	C57BL/6J	adeno-associated virus	vascular smooth muscle	KO	no	phosphate-induced	*Sirt*6 KO mice exhibit severe VC in CKD, with increased osteogenic transdifferentiation of	SIRT6 protects against vascular calcification by deacetylating Runx2, promoting its nuclear export and	[200]

Gene	Disease	Strains	Cre promoter	system specific	KO or OE	Inducible	Model background	Phenotype	Mechanism and conclusion	Reference
			serotype 2	cell				VSMCs.	degradation via the ubiquitin-proteasome system, thereby inhibiting osteogenic transdifferentiation of VSMCs.	
*Sirt*6	renal fibrosis	C57/BL6	Ggt1	kidney proximal tubule	KO	no	UUO-induced renal injuries and fibrosis	Loss of proximal tubule *Sirt*6 exacerbates UUO-induced renal tubulointerstitial inflammation and fibrosis.	SIRT6 in proximal tubules protects against renal fibrosis by regulating β-catenin acetylation and ECM protein promoter transcription.	[201]
*Sirt*1	renal fibrosis	C57BL/6J	Tenascin-C	renal interstitial cell	KO	no	UUO-induced renal fibrosis	*Sirt*1 KO exacerbates renal fibrosis and damage in UUO model mice.	SIRT1 protects against renal fibrosis by inhibiting HIF-2α expression and activity.	[202]
*Sirt*1	renal fibrosis	B6;129	Tie2	endothelial	KO	no	folic acid-induced tubulointerstitial fibrosis	*Sirt*1 KO mice exhibit impaired angiogenesis, increased vascular senescence, and exaggerated fibrotic response to nephrotoxic injury.	Endothelial SIRT1 deficiency exacerbates nephrosclerosis by downregulating MMP-14, leading to impaired matrix degradation and fibrosis.	[203]
*Sirt*1	female infertility	n/a	Zona pellucida 3	oocyte	KO	no	n/a	*Sirt*1 KO mice exhibit accelerated age-related decline in female fertility, with 50% of females becoming sterile between 9 and 11 months of age due to compromised oocyte quality.	SIRT1 maintains female fertility by preserving oocyte quality during aging, likely through regulating mitochondrial function and antioxidant defense, thereby preventing oxidative stress in embryos that leads to developmental arrest.	[204]
*Sirt*1	n/a	n/a	no	global	KO (*Sirt*1ΔE4, heterozygous)	no	n/a	*Sirt*1 KO mice exhibit perinatal lethality with accumulation of damaged organelles and disrupted energy homeostasis.	SIRT1 regulates autophagy by deacetylating key autophagy proteins, playing a crucial role in cellular nutrient adaptation and potentially mitigating 18296641agen/arelated pathologies.	[205]
*Sirt*3	female fertility	129S1/SvImJ	no	global	KO	no	n/a	*Sirt*3 KO mice exhibit increased ROS levels but normal ATP levels and intact female fertility under both standard and high-fat diet conditions.	SIRT3 regulates mitochondrial function and redox balance in oocytes, but its absence is compensated by other mechanisms, such as increased mitochondrial mass, to maintain ATP levels and female fertility.	[206]
*Sirt*1	placental development	129/Sv	no	global	KO (*Sirt*1-null)	no	n/a	*Sirt*1 KO mice exhibit embryonic lethality, small placentas with morphologic defects, and blunted trophoblast	SIRT1 is essential for proper trophoblast differentiation and placental development; its deficiency disrupts key signaling pathways, leading to abnormal placental	[207]

Gene	Disease	Strains	Cre promoter	system specific	KO or OE	Inducible	Model background	Phenotype	Mechanism and conclusion	Reference
								differentiation.	morphology and impaired fetal growth.	
*Sirt*1	manifested lactation failure	mixed 129SvJ/C57B6	no	global	KO (*Sirt*1ΔE4)	no	pregnancy-induced mammary gland development	*Sirt*1 KO mice exhibit growth retardation, impeded ductal morphogenesis in virgin females, and lactation failure due to underdeveloped mammary glands.	SIRT1 modulates the estrogen-IGF-1 signaling pathway, which is critical for mammary gland development and function; its deficiency deregulates IGF-1 signaling, leading to impaired ductal morphogenesis and lactation failure.	[208]
*Sirt*1	pregnancy failure	C57BL/6N	Elf5	trophoblast	KO	no	n/a	*Sirt*1 KO mice exhibit placental senescence, reduced fetal weight, and disrupted placental development.	SIRT1 promotes trophoblast EMT by deacetylating vimentin, enhancing placental development and potentially improving pregnancy outcomes in AMA pregnancies.	[209]
*Sirt*1	infertility	n/a	steroidogenic factor 1;Tnap	steroidogenic cell; germ cell	KO	no	n/a	Germ cell-specific *Sirt*1 KO mice exhibit severe infertility, reduced testis size, and malformed spermatozoa with abnormal acrosomes.	SIRT1 regulates acrosome biogenesis by modulating autophagic flux through LC3 deacetylation, which is essential for normal spermiogenesis and male fertility.	[210]
*Sirt*1	male infertility	n/a	Tnap	germ cell	KO	no	n/a	*Sirt*1 KO mice exhibit infertility with abnormal sperm morphology and disrupted acrosome biogenesis.	SIRT1 regulates acrosome biogenesis by modulating autophagy flux through deacetylation of LC3 and Atg7, which is essential for normal spermiogenesis and may contribute to male infertility when disrupted.	[211]
*Sirt*1	testicular injury	C57BL/6 J	SF1	Leydig cell-specific	KO	no	concentrated ambient PM2.5	*Sirt*1 KO mice exhibited exacerbated testicular injury and ferroptosis after exposure to PM2.5	SIRT1 protects against PM2.5-induced testicular injury and testosterone disruption by inhibiting the HIF-1α signaling pathway and ferroptosis.	[212]

Abbreviations: ABCG1, ATP-binding cassette transporter G1;AKI, acute kidney injury; AMA, advanced maternal age; Atg, autophagy-related gene; CCL, C-C motif chemokine ligand; CXCL, C-X-C motif chemokine ligand; CIAKI, contrast-induced acute kidney injury; CKD, chronic kidney disease; Dox, doxorubicin;DUSP16, dual Specificity Phosphatase 16; EMT, epithelial-mesenchymal transition; ERK, extracellular signal-regulated kinase; FOXO, forkhead box O; HIF, hypoxia-inducible factor; IGF, insulin-like growth factor; IRI, ischemia-reperfusion injury; KO, knockout; LPS, lipopolysaccharide; LC3, Microtubule-associated protein 1 light chain 3; MMP, matrix metalloproteinase; MSC, mesenchymal stromal cells; NMN, nicotinamide mononucleotide; OE, overexpression; PGC1α, PPAR-α coactivador-1; PPAR, proliferator-activated receptor; ROS, reactive oxygen species; *Sirt*, sirtuin; TNF, tumor necrosis factor; UUO, unilateral ureteral obstruction; VC, vascular calcification; VSMC, vascular smooth muscle cell

Supplementary Table S9. Summary of phenotypes and mechanisms of *Sirt* genes in transgenic mice with musculoskeletal diseases

Gene	Disease	Strains	Cre promoter	system specific	KO or OE	Inducible	Model background	Phenotype	Mechanism and conclusion	Reference
*Sirt*1	osteoporosis	C57BL/6J	paired related homeobox 1	mesenchymal stem cells	OE, 7.5 fold	no	Bmi1 KO mice exhibit an osteoporotic phenotype	*Sirt*1 OE mice exhibit enhanced bone formation, increased bone mass, and improved skeletal growth.	SIRT1 regulates bone metabolism by deacetylating FOXO3a, reducing oxidative stress and promoting osteoblastic bone formation.	[213]
*Sirt*1	osteoporosis	n/a	no	osteoblast	KO	tamoxifen	no	*Sirt*1 KO mice exhibit reduced bone mass due to decreased bone formation and resorption, with inhibited osteoblast differentiation, mineralization, and increased apoptosis.	SIRT1 maintains bone homeostasis by regulating osteoblast glycolysis through deacetylation of GOT1, highlighting its potential as a therapeutic target for bone-related diseases like osteoporosis.	[214]
*Sirt*1	osteoporosis	C57BL/6J	no	global	KO	no	separation-based anorexia model	*Sirt*1 KO mice exhibit decreased bone mass and increased bone marrow adiposity.	SIRT1 inhibits Runx2 and FOXO1 acetylation, inducing BMSC to differentiate into osteoblasts, increasing the bone mass to prevent bone loss.	[215]
*Sirt*1	osteoporosis	C57BL/6J	paired related homeobox 1	mesenchymal stem cells	OE	no	Bmi1 heterozygous mice exhibit an osteoporotic phenotype	*Sirt*1 OE leads to increased alveolar bone volume in mice.	SIRT1 promotes osteogenic differentiation by inducing deacetylation and nuclear translocation of Bmi1, thereby enhancing bone formation.	[216]
*Sirt*6	osteoporosis	C57BL/6	osteocalcin	osteoblast/ osteocyte	KO	no	no	*Sirt*6 KO mice exhibit reduced bone mass due to increased osteoclast formation.	SIRT6 in osteoblasts and osteocytes inhibits osteoclastogenesis via a paracrine mechanism by regulating OPG expression.	[217]
*Sirt*6	osteoporosis	B6;129	LysM	myeloid	KO	no	sham-operated or ovariectomized surgical models	*Sirt*6 KO mice exhibit exacerbated cancellous bone loss, accompanied by reduced ERα protein levels and increased osteoclast formation.	Deacetylation and upregulation of ERα by SIRT6 in preosteoclasts prevent bone loss by inhibiting osteoclast-mediated bone resorption.	[218]
*Sirt*3	osteoporosis	B6;129	no	global	KO	no	sham-operated or ovariectomized surgical models	*Sirt*3 KO mice exhibit increased bone mass.	SIRT3 promotes osteoclastogenesis and bone resorption by enhancing mitochondrial function and mitophagy in osteoclasts, contributing to age-related and estrogen deficiency-induced bone loss.	[219]
*Sirt*3	age-related	C57BL/6	LysM	osteoclasts and	KO	no	mouse model of skeletal aging	*Sirt*3 KO mice exhibit attenuated age-related bone loss in female but not	SIRT3 promotes bone resorption and skeletal aging by maintaining	[220]

Gene	Disease	Strains	Cre promoter	system specific	KO or OE	Inducible	Model background	Phenotype	Mechanism and conclusion	Reference
	osteoporosis			osteoclasts				male mice, with decreased osteoclast function and mitochondrial dysfunction in osteoclasts.	mitochondrial quality in osteoclasts via deacetylation of mitochondrial proteins, including ATPIF1, which is essential for mitophagy and mitochondrial function.	
*Sirt*1	osteoarthritis	C57BL/6	α- tubulin chondrocyte	cartilage	KO	no	post-traumatic osteoarthritis;age-dependent osteoarthritis	*Sirt*1 KO mice exhibit reduced cartilage integrity and elevated osteoarthritis severity, with decreased serum NT/CT SIRT1 ratio.	SIRT1 plays a crucial role in maintaining cartilage homeostasis and its deficiency accelerates osteoarthritis development, likely through non-senescent chondrocyte apoptosis.	[221]
*Sirt*1	osteoarthritis	n/a	no	global	KO（*Sirt* 1$^{y/y}$）	no	no	*Sirt*1 KO mice presence of defects in the cartilage of the skull, spine, rib cages, and joints.	SIRT1 plays a crucial role in maintaining cartilage homeostasis by regulating gene expression and deacetylation of histone targets, and its deficiency accelerates cartilage degradation and osteoarthritis development.	[222]
*Sirt*1	osteoarthritis	C57BL/6	aggrecan enhancer	cartilage	KO	tetracycline	post-traumatic OA	*Sirt*1 KO mice exhibit enhanced cartilage mineralization and severe OA structural alterations, particularly in the lateral joint compartment.	SIRT1 negatively regulated the expression of Lef1 and its absence exacerbates OA progression via increased inflammatory pathways and cartilage catabolism.	[223]
*Sirt*3	osteoarthritis	C57BL/6J	no	global	KO	no	no	*Sirt*3 KO mice exhibit early-stage OA with increased oxidative stress and reduced cartilage SOD2 activity.	SIRT3 protects cartilage by deacetylating and activating SOD2, thereby reducing oxidative stress and mitigating OA progression.	[224]
*Sirt*1	age-related sarcopenia pathogenesis	C57BL/6J	prion	brain	OE	no	no	*Sirt*1 OE mice exhibit more youthful neuromuscular junction morphology.	Hypothalamic SIRT1 protects terminal Schwann cells and neuromuscular junctions from age-related morphological changes.	[225]
*Sirt*1	age-related sarcopenia pathogen	C57BL/6J	paired box 7;muscle creatine kinase	satellite cell;skeletal muscle	KO	no	older mice	*Sirt*1 KO in satellite cells of older mice reduces maximal muscle force; *Sirt*1 KO in muscle increases satellite cell proliferation but shows no significant difference in muscle	SIRT1 improves muscle repair and function after injury by interacting with p53 and enhancing satellite cell activation and mitochondrial function.	[226]

Gene	Disease	Strains	Cre promoter	system specific	KO or OE	Inducible	Model background	Phenotype	Mechanism and conclusion	Reference
	esis							structure or function.		
*Sirt*6	age-related sarcopenia pathogenesis	129svJ	no	global	KO	no	n/a	*Sirt*6 KO mice exhibit muscle wasting, reduced muscle fiber size, increased fibrosis, and upregulated atrophic markers such as myostatin.	SIRT6 inhibited the development of muscular atrophy by negatively regulating Mstn expression via suppressing NF-κB signaling.	[227]

Abbreviations: Bmi1, B cell-specific Moloney murine leukemia virus integration site 1; CT, C-terminal; ER, estrogen receptor; FOXO, forkhead box O; KO, knockout; Lef1, lymphoid enhancer-binding factor 1; Mstn, myostatin; NF-κB, nuclear factor kappa-light-chain-enhancer of activated B cells; NT, N-terminal; OPG, osteoprotegerin; OA, osteoarthritis; OE, overexpression; Runx2, runt-related transcription factor 2; SOD2, superoxide dismutase 2; *Sirt*, sirtuin.

Supplementary Table S10. Summary of phenotypes and mechanisms of *Sirt* genes in transgenic mice with malignant tumours

Gene	Disease	Strains	Cre promoter	system specific	KO or OE	Inducible	Model background	Phenotype	Mechanism and conclusion	Reference
*Sirt*2	BC	C57BL/6N	no	global	KO	no	no	*Sirt*2 KO mice exhibit abnormal T cell differentiation, characterized by increased naive T cells and decreased effector memory T cells.	SIRT2 promotes CD8+ T cell differentiation into effector memory T cells by enhancing aerobic oxidation and inhibiting GSK3β acetylation, thereby enhancing antitumor immunity.	[228]
*Sirt*4	BC	n/a	no	global	KO	no	MMTV-Neu mice, which mimic the human luminal phenotype and develop spontaneous mammary tumors	*Sirt*4 KO mice exhibit increased mammary gland ductal side-branching and a higher number of mammary stem cells, leading to enhanced tumorigenesis and metastasis.	SIRT4 acts as a tumor suppressor by inhibiting glutamine metabolism, thereby downregulating SIRT1 expression and suppressing breast cancer stemness through modulation of H4K16ac and BRCA1.	[229]
*Sirt*6	BC	129SvJ	no	global	KO	no	expressing the PyMT under the MMTV promo ter induced BC	*Sirt*6 KO mice exhibit increased tumor latency and enhanced survival.	SIRT6 promotes breast cancer progression by enhancing oxidative phosphorylation and energy status, while its depletion reduces tumor growth through metabolic stress.	[230]
*Sirt*5	BC	n/a	no	global	KO	no	MMTV	*Sirt*5 KO mice reduces tumour volume and weight	SIRT5 promotes tumour survival by facilitating mitochondrial ROS detoxification	[231]
*Sirt*1	LC	n/a	no	global	KI	no	LLC xenograft	*Sirt*1 KI mice develop larger tumours (~15% volume increase) than WT controls	SIRT1 facilitates endothelial cell branching and proliferation to increase vessel density and promote lung tumor growth through down-regulation of DLL4/Notch signaling and deacetylation of N1IC	[232]
*Sirt*1	HCC	n/a	albumin	liver	KO	no	DEN-induced HCC	*Sirt*1 KO mice exhibit resistance to DEN-induced HCC development, with reduced tumor burden and enhanced glutathione metabolism.	SIRT1 deficiency upregulates glutathione metabolism via Nrf2 activation, creating a reductive environment that prevents HCC initiation by reducing DNA damage and cell proliferation.	[233]
*Sirt*4	HCC	n/a	no	global	KO	no	DEN-induced HCC	*Sirt*4 KO mice exhibit increased HCC tumor development, with enhanced	SIRT4 acts as a tumor suppressor in HCC by inhibiting glutamine metabolism, thereby increasing	[234]

Gene	Disease	Strains	Cre promoter	system specific	KO or OE	Inducible	Model background	Phenotype	Mechanism and conclusion	Reference
								tumor masses, sizes, and numbers, as well as increased lung metastasis and reduced survival rates.	ADP/AMP levels to activate AMPKα via LKB1, which subsequently blocks the mTOR signaling pathway.	
*Sirt*7	HCC	C57BL/6	albumin	liver	KO	no	implantation of tumor cells into the parenchyma of the lowersurface of the left liver lobe	*Sirt*7 KO mice exhibit enhanced antitumor immunity but reduced tumor growth due to increased PD-L1 expression and T-cell infiltration.	SIRT7 promotes HCC progression by regulating PD-L1 expression via MEF2D acetylation, thereby modulating antitumor immunity.	[235]
*Sirt*2	HCC (male); BC (femal e)	NIH Black Swiss or C57B6	no	global	KO	no	no	*Sirt*2 KO mice develop gender-specific tumorigenesis, with females primarily developing mammary tumors and males developing more hepatocellular carcinoma.	SIRT2 acts as a tumor suppressor by maintaining genome integrity through regulating APC/C activity via deacetylation of its coactivators CDH1 and CDC20.	[236]
*Sirt*5	HCC	C57BL/6J	no	global	KO	no	no	*Sirt*5 KO mice exhibit increased peroxisomal H_2O_2 production, oxidative DNA damage.	SIRT5 inhibits peroxisomal ACOX1 activity through desuccinylation, preventing oxidative damage and suppressing liver cancer development.	[237]
*Sirt*6	HCC	129-*Sirt*6^{tm1Fwa}/J	no	global	KO	no	no	*Sirt*6 KO mice exhibit severe hypoglycemia, increased hepatic fat deposition, and global DNA hypomethylation, leading to a pro-oncogenic phenotype.	SIRT6 acts as a tumor suppressor by maintaining liver homeostasis and metabolic stability; its deficiency induces epigenetic changes and oncogenic transformations.	[238]
*Sirt*1	PDAC	B6;129-*Sirt*1^{tm1Ygu}/J	pdx1	pancreas	KO	no	Kras^{G12D} mutant mice predisposes to develop PanIN and PDAC	*Sirt*1 KO mice exhibit reduced proliferation and glycolysis gene expression in early mucinous PanIN lesions.	SIRT1 promotes the proliferation and glycolysis gene expression in pancreatic neoplastic lesions by regulating metabolic pathways, contributing to tumor progression.	[239]
*Sirt*6	PDAC	mixed 129Sv/C57 BL/6	p48	pancreas	KO	no	p53 and Kras^{G12D} mutant mice to develop PDAC	*Sirt*6 KO mice develop pancreatic tumors more rapidly and exhibit increased metastasis.	SIRT6 acts as a tumor suppressor in pancreatic cancer by inhibiting the expression of Lin28b through histone deacetylation, thereby preventing	[240]

Gene	Disease	Strains	Cre promoter	system specific	KO or OE	Inducible	Model background	Phenotype	Mechanism and conclusion	Reference
									tumor growth and metastasis.	
*Sirt*1	PC	n/a	no	global	KO (*Sirt*1Δ E4)	no	no	*Sirt*1 KO mice exhibit reduced prostate size and develop PIN with increased cellularity and nuclear atypia.	SIRT1 acts as a tumor suppressor in the prostate by promoting autophagy and inhibiting cell proliferation through regulation of androgen signaling pathways.	[241]
*Sirt*5	melanoma	n/a	no	global	KO	no	Braf and Pten, Tyr mutant mice to develop melanoma	*Sirt*5 KO mice are viable and mostly healthy, with mild phenotypes observed in the myocardium.	SIRT5 promotes melanoma cell survival and proliferation by maintaining histone acetylation and methylation, thereby supporting the expression of critical oncogenes such as MITF and c-MYC.	[242]
*Sirt*6	HNSCC	C57BL/6	K14	skin epithelium	KO	no	DMBA /TPA-induced skin carcinogenesis	*Sirt*6 KO mice exhibit accelerated tumor onset and larger tumor size.	SIRT6 acts as a tumor suppressor by negatively regulating glycolysis, and its loss promotes tumor progression through enhanced glycolysis and antioxidant response in tumor-propagating cells.	[243]
*Sirt*1	T-ALL	C57BL/6J	Mx1	hematopoietic stem and progenitor cells	KO	poly I:C	notch1-induced leukemia	*Sirt*1 KO mice exhibit reduced leukemia burden and prolonged survival in a T-ALL model.	SIRT1 promotes T-ALL progression by deacetylating CDK2, which increases p27 phosphorylation and degradation, thereby enhancing cell cycle progression.	[244]
*Sirt*1	CML	C57BL/6	Mx1	hematopoietic stem and progenitor cells	KO	tamoxifen	CML model	*Sirt*1 KO mice exhibit reduced leukemia development and increased survival.	SIRT1 promotes leukemia stem cell maintenance and tyrosine kinase inhibitor resistance in CML by enhancing mitochondrial oxidative phosphorylation	[245]
*Sirt*3	DLBCL	C57BL/6J	no	global	KO	no	VavP-Bcl2 transgenic model mimic B-cell lymphomagenes	*Sirt*3 KO mice exhibit reduced lymphoma development and improved survival in a VavP-Bcl2 transgenic model.	SIRT3 promotes DLBCL proliferation and survival by enhancing mitochondrial metabolism through GDH deacetylation, supporting its role as a metabolic oncogene.	[246]
*Sirt*4	B Cell Lymphoma	C57BL/6	no	global	KO	no	Myc-induced B cell lymphoma	*Sirt*4 KO mice exhibit accelerated lymphomagenesis and	SIRT4 suppresses Myc-induced B cell lymphoma by inhibiting mitochondrial glutamine metabolism,	[247]

Gene	Disease	Strains	Cre promoter	system specific	KO or OE	Inducible	Model background	Phenotype	Mechanism and conclusion	Reference
								reduced survival in a Myc-induced B cell lymphoma model.	independent of Myc, highlighting its role as a tumor suppressor.	

Abbreviations:

ACOX1, acyl-coA oxidase 1; AMP, adenosine-monophosphate; AMPKα, AMP-activated protein kinase alpha; APC/C, anaphase-promoting complex/cyclosome; BC, breast cancer; CDT, chromatin licensing and DNA replication factor 1; CML, chronic myeloid leukemia; DEN, diethylnitrosamine; DLBCL, diffuse large B cell lymphoma; DMBA, 7,12-dimethylbenz[a]anthracene; DOX, doxorubicin; FAO, fatty acid oxidation; FOXO3, forkhead box O3; GDH, glutamine dehydrogenase; GSK3β, glycogen synthase kinase 3β; HCC, hepatocellular carcinoma; HNSCC, head and neck squamous cell carcinoma; KD, knockdown; KO, knockout; LKB1, liver kinase B1; MEF2D, myocyte enhancer factor 2D; MITF, melanocyte-inducing transcription factor; mTOR, mammalian target of rapamycin; Nrf2, nuclear factor-erythroid 2-related factor 2; NSCLC, non-small cell lung cancer; PDAC, pancreatic ductal adenocarcinoma; PD-L1, programmed cell death 1 ligand 1; PanIN, pancreatic intraepithelial neoplasia; PC, prostate cancer; PIN, prostatic intraepithelial neoplasia; PPARδ, peroxisome proliferator-activated receptor gamma; PyMT, polyoma middle T antigen; MMTV, mouse mammary tumor virus; SIRT, sirtuin; T-ALL, T-cell acute lymphoblastic leukemia; TPA, 12-O-tetradecanoylphorbol-13-acetate; c-MYC, c-Myc proto-oncogene protein.

Supplementary Table S11. Summary of phenotypes and mechanisms of *Sirt* genes in transgenic mice with immune diseases

Gene	Disease	Strains	Cre promoter	system specific	KO or OE	Inducible	Model background	Phenotype	Mechanism and conclusion	Reference
*Sirt*1	EAE	C57BL/6	CD11c	DC	KO	no	EAE induced by MOG35-55	Specific *Sirt*1 KO are resistant to MOG-induced EAE and exhibit reduced Th17 differentiation and inflammation,	SIRT1 promotes Th17 differentiation and inflammation by deacetylating the IRF1 transcription factor, thereby suppressing IL-27 expression in dendritic cells.	[248]
*Sirt*1	EAE	C57BL/6	GFAP	astrocyte	KO	no	no	*Sirt*1 KO mice exhibit reduced progression of experimental autoimmune EAE with decreased CNS inflammation, demyelination, and increased numbers of IL-10-producing macrophages/microglia.	SIRT1 inactivation in astrocytes promotes an anti-inflammatory phenotype by enhancing the activity of the transcription factor NRF2, which upregulates anti-inflammatory and antioxidant genes, thereby inhibiting CNS autoimmunity and demyelination in diseases like multiple sclerosis.	[249]
*Sirt*1	EAE	C57BL/6	RORγt	T-cell	KO	no	EAE induced by MOG35-55	Specific *Sirt*1 KO mice exhibit reduced Th17 cell differentiation and are protected from EAE.	SIRT1promotes Th17 cell differentiation by deacetylating RORγt, enhancing its transcriptional activity and driving proinflammatory responses in autoimmune diseases.	[250]
*Sirt*2	SLE	C57BL/6J	no	global	KO	no	EAE induced by adoptive cell transfer	*Sirt*2-deficient CD4+ T cells exhibit reduced Th17 differentiation, increased IL-2 production, and protection against adoptive transfer EAE and lupus-like disease.	SIRT2 promotes Th17 cell differentiation and suppresses IL-2 production by deacetylating p70S6K, c-Jun, and histones at the Il-2 gene promoter, contributing to autoimmune disease pathogenesis.	[251]
*Sirt*6	RA	B6; 129	LysM	myeloid	KO	no	collagen-induced arthritis	Specific *Sirt*6 KO mice exhibited more severe arthritis with increased macrophage infiltration and joint destruction.	SIRT6 regulates macrophage activation and migration by deacetylating FoxO1, thereby suppressing inflammation and joint destruction in RA.	[252]
*Sirt*1	GVHD	C57BL/6	CD4	T-cell	KO	no	acute GVHD and chronic GVHD induced by allogeneic bone marrow transplantation	*Sirt*1 KO mice exhibit reduced T-cell pathogenicity, with enhanced p53 acetylation and increased iTreg stability.	SIRT1 promotes T-cell activation and inflammatory cytokine production through p53 deacetylation, enhancing GVHD pathogenesis.	[253]
*Sirt*3	GVHD	C57BL/6	no	global	KO	no	allogeneic bone	*Sirt*3 KO donor T cells exhibit	SIRT3 promotes T cell activation and	[254]

Gene	Disease	Strains	Cre promoter	system specific	KO or OE	Inducible	Model background	Phenotype	Mechanism and conclusion	Reference
							marrow transplantation	reduced activation, lower CXCR3 expression, and decreased ROS production, attenuated GVHD severity.	ROS production, thereby exacerbating GVHD, while its absence in donor T cells mitigates GVHD by reducing T cell activation and ROS levels.	
*Sirt*1	atopic dermati tis	C57BL/6	K14	skin keratino cyte	KO	no	cutaneous treatment with ovalbumin (allergen challenge)	*Sirt*1 KO mice develop atopic dermatitis-like skin lesions and exhibit increased sensitivity to epicutaneous allergen challenge.	SIRT1 maintains skin barrier integrity by promoting FLG expression through the deacetylase-dependent AhR/Akt pathway, thereby protecting against allergen-induced skin inflammation.	[255]
*Sirt*6	allergy	C57BL/6	LysM	myeloid	KO	no	passive systemic anaphylaxis ; passive cutaneous anaphylaxis	*Sirt*6 KO mice exhibit enhanced IgE-mediated anaphylactic responses and increased mast cell degranulation.	SIRT6 acts as a negative regulator of FcεRI signaling in mast cells by suppressing PTPRC transcription, thereby attenuating allergic reactions.	[256]
*Sirt*2	infectio n	C57BL/6	n/a	global	KO	no	chronic Staphylococcus aureus infection	*Sirt*2 KO mice show increased macrophage phagocytosis and improved survival	SIRT2 negatively regulates bacterial phagocytosis in macrophages and host anti-infective immunity by deacetylating α-tubulin and inhibiting glycolytic metabolism.	[257]
*Sirt*2	infectio n	C57BL/6	n/a	global	KO	no	listeria monocytogenes infection	*Sirt*2 KO mice exhibited reduced susceptibility to Listeria monocytogenes infection, with significantly lower bacterial loads in spleens.	SIRT2 mediates histone H3K18 deacetylation in response to Listeria monocytogenes infection, promoting bacterial replication by reprogramming host gene expression through the PI3K/Akt signaling pathway.	[258]
*Sirt*3	infectio n	C57BL/6 ; 129S1/Sv ImJ	n/a	global	KO	no	cecal ligation and puncture	*Sirt*3 KO mice did not show significant differences in sepsis survival rates.	SIRT3 interacts sequentially with Rel B and SIRT1 to regulate mitochondrial bioenergetics and immune function during sepsis adaptation, potentially contributing to improved sepsis outcomes as a therapeutic target.	[259]
*Sirt*3	infectio n	C57BL/6	n/a	global	KO	no	mycobacterial infection	*Sirt*3 KO mice exhibited markedly increased bacterial load in the lungs, elevated mortality, exacerbated pathological inflammation accompanied by neutrophil infiltration, alongside	SIRT3 maintains PPARA and TFEB expression through deacetylation, coordinating mitochondrial homeostasis with autophagy activation. This inhibits excessive inflammation and promotes the formation of antimicrobial autophagosomes to	[260]

Gene	Disease	Strains	Cre promoter	system specific	KO or OE	Inducible	Model background	Phenotype	Mechanism and conclusion	Reference
								mitochondrial dysfunction and ROS accumulation.	eliminate intracellular mycobacteria.	
*Sirt*2 and *Sirt*3	infectio n	C57BL/6	n/a	global	KO	no	LPS -induced endotoxemia model	*Sirt*2 and *Sirt*3 KO mice exhibit subtle alterations in immune cell populations and metabolism, with increased peritoneal B-1a cells and protection from endotoxemia.	SIRT2 and SIRT3 act in concert to regulate macrophage metabolism and cytokine production, with their dual deficiency enhancing anti-inflammatory responses and protecting against acute inflammation.	[261]
*Sirt*3 and *Sirt*5	infectio n	C57BL/6	n/a	global	KO	no	LPS -induced endotoxemia model and the listeriosis model induced by intravenous injection of Listeria monocytogenes	*Sirt*3 and *Sirt*5 KO mice exhibit normal development with subtle alterations in immune cell populations and enhanced inflammatory responses, showing improved resistance to Listeria monocytogenes infection.	SIRT3 and SIRT5 regulate mitochondrial metabolism and redox homeostasis, with their dual deficiency leading to increased inflammatory cytokine production and bactericidal activity, suggesting potential therapeutic targets for inflammatory and infectious diseases.	[262]
*Sirt*5	infectio n	C57BL/6	n/a	global	KO	no	sepsis induced by LPS	*Sirt*5 KO mice exhibit reduced inflammatory cytokine production and attenuated immune responses in both hyper-inflammatory and hypo-inflammatory phases of sepsis.	SIRT5 promotes the innate inflammatory response by enhancing NF-κB activation through acetylation of p65, counteracting SIRT2, and rescuing hypo-inflammatory states in endotoxin-tolerant macrophages, suggesting its potential as a therapeutic target for sepsis.	[263]

Abbreviations: Akt, protein kinase B; AhR, aryl hydrocarbon receptor; AD, atopic dermatitis; DC, dendritic cell; EAE, experimental autoimmune encephalomyelitis; FcεRI, Fcε receptor I; FLG, filaggrin; FoXO, forkhead box O; GVHD, graft-versus-host disease; H3K18, histone 3 Lysine 18; IFN-β, interferon β; IL, cytokines interleukin; IRF1, IFN regulatory factor-1; iTreg, regulatory T cell; IgE, immunoglobulin E; LPS, lipopolysaccharide; MOG35-55, myelin oligodendrocyte glycoprotein -derived peptide; mTOR, mammalian target of rapamycin; n/a, not applicable; PI3K, phosphatidylinositol 3-kinase; PTPRC, protein tyrosine phosphatase receptor type C; RA, rheumatoid arthritis; RORγt, retinoic acid-related orphan receptor gamma T; ROS, reactive oxygen species; SIRT, sirtuin; Th17, T helper 17; WT, wild type; CXCR3, C-X-C motif chemokine receptor 3; NF-κB, nuclear factor kappa-light-chain-enhancer of activated B cells; KO, knockout.

Supplementary Table S12. Representative small-molecule sirtuin modulators with cellular and in vivo evidence

Type	Compound / drug	Primary SIRT target(s)	Mechanism of Action	Key findings	Reference
Activator	Resveratrol	SIRT1	Polyphenolic STAC; allosterically enhances SIRT1 activity on selected hydrophobic substrates	Improves insulin sensitivity, lipid profile and endothelial function in obese / T2D / CAD patients	[264, 265]
Activator	SRT2104	SIRT1	Potent small-molecule STAC; binds N-terminal SIRT1 activation domain	Improves insulin sensitivity, lipid handling and arterial compliance in obese rodents and in smokers / T2D patients	[266]
Activator	MC2562	SIRT1/2/3	1,4 dihydropyridine based SIRT1/2/3 activator; increases SIRT1 dependent deacetylation and NO release	Accelerates cutaneous wound healing in mice; reduces H4K16 acetylation in tumour cells	[267]
Activator	ISIDE11	SIRT1	Small molecule SIRT1 activator with selectivity over class I/II HDACs, and in vivo cardiovascular efficacy	Rescues vascular dysfunction and prevents thrombosis in MTHFR deficient mice	[268]
Activator	Honokiol	SIRT3	Traditional Chinese medicine Magnolia officinalis directly binds to enhance SIRT3 deacetylase activity and transcriptionally upregulate SIRT3 expression.	Improves cognitive function in AD model；ameliorates colitis； reduces alveolar bone loss caused by diabetes-related periodontitis in mice	[269]
Activator	ADTL SA1215 (3c)	SIRT3	Amiodarone derived SIRT3 activator; binds entrance of SIRT3 acyl channel and enhances deacetylase activity	Induces SOD2 deacetylation, autophagy/mitophagy and suppresses TNBC xenograft growth (lung toxicity at high dose)	[270]
Activator	UBCS039	SIRT6， mild SIRT5	Tricyclic SIRT6 activator; binds distal acyl channel	ameliorates hepatic lipogenesis through liver X receptor deacetylation in mice	[271]
Activator	MDL-800/811	SIRT6	Potent, highly selective allosteric SIRT6 activators; >20 fold increase in deacetylase activity	Suppress HCC and NSCLC xenograft growth; ameliorate osteoarthritis and cardiomyopathy;	[272-274]
Inhibitor	Selisistat (EX-527)	SIRT1>SIRT2/3	Binds catalytic pocket after alkylimidate formation and traps SIRT1-ADPR intermediate; blocks deacetylase activity	Safe in Phase I; in early HD patients shows short-term clinical/neuropsychiatric improvement ; anti-lung cancer-xenograft model; anti-esophageal cancer-xenograft model	[275-277]
Inhibitor	AGK2	SIRT2 ≫ SIRT1/3	Reversible SIRT2 inhibitor; increases α-tubulin acetylation	Protects mice from colitis； enhanced anti-tumor immune in vivo	[278, 279]
Inhibitor	SirReal2	SIRT2	Highly selective SIRT2 inhibitor binding the "selectivity pocket"; locks SIRT2 in open conformation	Increases α-tubulin acetylation; impairs growth of cancer xenografts	[280, 281]
Inhibitor	Thiomyristoyl TM/AF8	SIRT2	Mechanism-based thiomyristoyl-lysine inhibitors; form long-lived covalent adduct with NAD^+, sustaining SIRT2 inhibition and α-tubulin hyperacetylation	Induce c-Myc degradation and show strong anti-proliferative activity in c-Myc-driven breast and colorectal cancer xenograft models (limited by low solubility and complex synthesis)	[282, 283]
Inhibitor	Tenovin-6	SIRT1/2 (weak SIRT3)	Direct inhibitor of SIRT1-3 catalytic activity	Increases p53 and α-tubulin acetylation; induces	[284]

Type	Compound / drug	Primary SIRT target(s)	Mechanism of Action	Key findings	Reference
				apoptosis in medulloblastoma and melanoma; slows xenograft growth	
Inhibitor	MC3482	SIRT5	ε-N-glutaryl-lysine analogue; selectively inhibits SIRT5 desuccinylase activity and increases global succinylation	stimulates brown adipogenesis in vitro	[285]
Inhibitor	Et-40b / Et-40c (NRD167/NRD139)	SIRT5	Mechanism-based thioglutaryl-lysine inhibitors (prodrugs); form stalled 1′-thioimidate with ADP-ribose and block deacylation	Selectively kill SIRT5-dependent AML cells; reduce growth and induce apoptosis in SIRT5-addicted AML xenografts	[286]
Inhibitor	DK1-04 (Et-DK1-04 / Ac-DK1-04)	SIRT5	Thiosuccinyl-peptide-derived inhibitor; covalently traps SIRT5 and blocks desuccinylation	Increases protein succinylation; reduces tumour burden in breast-cancer models	[231]
Inhibitor	Balsalazide	SIRT5	Clinically used 5-aminosalicylic acid prodrug that also occupies SIRT5 acyl-binding pocket	Approved for ulcerative colitis	[287]
Inhibitor	OSS_128167	SIRT6	Small-molecule SIRT6 inhibitor; reduces H3K9/H3K56 deacetylation	Reduces airway inflammation and remodelling in asthma mouse models	[288]
Inhibitor	Compound 8a	SIRT6	Non-competitive SIRT6 inhibitor (pyrrolo-pyridyl-imidazole) binding distal acyl-channel site	Enhances chemosensitivity and impairs DNA-damage repair in pancreatic cancer xenografts	[289]
Inhibitor	SZU-B6	SIRT6	PROTAC degrader that recruits an E3 ligase to induce proteasomal degradation of SIRT6	Depletes SIRT6 and shows anti-tumour efficacy in liver-cancer xenograft models	[290]
Activator	Resveratrol	SIRT1	Polyphenolic STAC; allosterically enhances SIRT1 activity on selected hydrophobic substrates	Improves insulin sensitivity, lipid profile and endothelial function in obese / T2D / CAD patients	[264, 291]
Activator	SRT2104	SIRT1	Potent small-molecule STAC; binds N-terminal SIRT1 activation domain	Improves insulin sensitivity, lipid handling and arterial compliance in obese rodents and in smokers / T2D patients	[266]
Activator	MC2562	SIRT1/2/3	1,4 dihydropyridine based SIRT1/2/3 activator; increases SIRT1 dependent deacetylation and NO release	Accelerates cutaneous wound healing in mice; reduces H4K16 acetylation in tumour cells	[267]
Activator	ISIDE11	SIRT1	Small molecule SIRT1 activator with selectivity over class I/II HDACs, and in vivo cardiovascular efficacy	Rescues vascular dysfunction and prevents thrombosis in MTHFR deficient mice	[268]
Activator	Honokiol	SIRT3	Traditional Chinese medicine Magnolia officinalis directly binds to enhance SIRT3 deacetylase activity and transcriptionally upregulate SIRT3 expression.	Improves cognitive function in AD model; ameliorates colitis; reduces alveolar bone loss caused by diabetes-related periodontitis in mice	[269]
Activator	ADTL SA1215 (3c)	SIRT3	Amiodarone derived SIRT3 activator; binds entrance of SIRT3 acyl channel and enhances	Induces SOD2 deacetylation, autophagy/mitophagy and suppresses TNBC	[270]

			deacetylase activity	xenograft growth (lung toxicity at high dose)	
Activator	UBCS039 (also mild SIRT5)	SIRT6 (also mild SIRT5)	Tricyclic SIRT6 activator; binds distal acyl channel	ameliorates hepatic lipogenesis through liver X receptor deacetylation in mice	[271]
Activator	MDL-800/811	SIRT6	Potent, highly selective allosteric SIRT6 activators; >20 fold increase in deacetylase activity	Suppress HCC and NSCLC xenograft growth; ameliorate osteoarthritis and cardiomyopathy;	[272-274]
Inhibitor	Selisistat (EX-527)	SIRT1≻SIRT2/3	Binds catalytic pocket after alkylimidate formation and traps SIRT1-ADPR intermediate; blocks deacetylase activity	Safe in Phase I; in early HD patients shows short-term clinical/neuropsychiatric improvement ; anti-lung cancer-xenograft model ; anti-esophageal cancer-xenograft model	[277] [276]
Inhibitor	AGK2	SIRT2 ≫ SIRT1/3	Reversible SIRT2 inhibitor; increases α-tubulin acetylation	Protects mice from colitis; enhanced anti-tumor immune in vivo	[278, 279]
Inhibitor	SirReal2	SIRT2	Highly selective SIRT2 inhibitor binding the "selectivity pocket"; locks SIRT2 in open conformation	Increases α-tubulin acetylation; impairs growth of cancer xenografts	[280, 281]
Inhibitor	Thiomyristoyl TM/AF8	SIRT2	Mechanism-based thiomyristoyl-lysine inhibitors; form long-lived covalent adduct with NAD^+, sustaining SIRT2 inhibition and α-tubulin hyperacetylation	Induce c-Myc degradation and show strong anti-proliferative activity in c-Myc-driven breast and colorectal cancer xenograft models (limited by low solubility and complex synthesis)	[282, 283]
Inhibitor	Tenovin-6	SIRT1/2 (weak SIRT3)	Direct inhibitor of SIRT1-3 catalytic activity	Increases p53 and α-tubulin acetylation; induces apoptosis in medulloblastoma and melanoma; slows xenograft growth	[284]
Inhibitor	MC3482	SIRT5	ε-N-glutaryl-lysine analogue; selectively inhibits SIRT5 desuccinylase activity and increases global succinylation	stimulates brown adipogenesis in vitro	[285]
Inhibitor	Et-40b / Et-40c (NRD167/NRD139)	SIRT5	Mechanism-based thioglutaryl-lysine inhibitors (prodrugs); form stalled 1′-thioimidate with ADP-ribose and block deacylation	Selectively kill SIRT5-dependent AML cells; reduce growth and induce apoptosis in SIRT5-addicted AML xenografts	[286]
Inhibitor	DK1-04 (Et-DK1-04 / Ac-DK1-04)	SIRT5	Thiosuccinyl-peptide-derived inhibitor; covalently traps SIRT5 and blocks desuccinylation	Increases protein succinylation; reduces tumour burden in breast-cancer models	[231]
Inhibitor	Balsalazide	SIRT5	Clinically used 5-aminosalicylic acid prodrug that also occupies SIRT5 acyl-binding pocket	Approved for ulcerative colitis	[287]
Inhibitor	OSS_128167	SIRT6	Small-molecule SIRT6 inhibitor; reduces H3K9/H3K56 deacetylation	Reduces airway inflammation and remodelling in asthma mouse models	[288]

Inhibitor	Compound 8a	SIRT6	Non-competitive SIRT6 inhibitor (pyrrolo-pyridyl-imidazole) binding distal acyl-channel site	Enhances chemosensitivity and impairs DNA-damage repair in pancreatic cancer xenografts	[289]
Inhibitor	SZU-B6	SIRT6	PROTAC degrader that recruits an E3 ligase to induce proteasomal degradation of SIRT6	Depletes SIRT6 and shows anti-tumour efficacy in liver-cancer xenograft models	[290]
Inhibitor	ID:97491	SIRT7	Small-molecule SIRT7 inhibitor; reduces deacetylase activity	Increases p53 acetylation and phosphorylation; promotes apoptosis and suppresses tumour growth in uterine-sarcoma xenografts	[292]

Key activators and inhibitors of SIRT1-SIRT7 are summarized, including their primary isoform selectivity, core mechanism of action, main preclinical or clinical indications, and supporting references.

Supplementary Table S13. Representative preclinical clinical trials of SIRT-modulating interventions, grouped by pathway and mechanism of action

Diseases	Trial ID	Intervention	Participant	SIRT-related findings	Outcomes	Primary mechanism of action
Type 2 Diabetes and NAFLD	IRCT201406183664N12 [293]	Turmeric powder (500-mg capsules)	NAFLD patients	Effectively improved serum SIRT1 levels	Blood pressure, serum SIRT1, and adiponectin	Anti-inflammatory and metabolic regulation via SIRT1 upregulation
	IRCT2015121317254N4 [294]	Green cardamom (3 g/day)	Obese patients with NAFLD	Improved biomarkers related to fatty liver including SIRT1	Serum SIRT1 and inflammation	SIRT1-mediated reduction of hepatic inflammation
	IRCT201511233664N16 [295]	Resveratrol (600 mg/day)	NAFLD patients	No significant changes seen in SIRT1 levels	Anthropometric indices and serum SIRT1	Caloric restriction mimicking (failed to reach significance)
	IRCT20141025019669N13 [296]	Oral ellagic acid (180 mg once daily)	T2D patients	Significantly increased SIRT1 levels	Insulin resistance and serum SIRT1	Antioxidant-driven upregulation of SIRT1 signaling
	IRCT201604202365N11 [297]	Vitamin D (50,000 IU/week)	Obese T2D patients	Increase of serum SIRT1 was significant	HbA1c, IR indexes, and serum SIRT1	Vitamin D receptor (VDR) mediated SIRT1 activation
	IRCT2016042717254N5 [298]	Green coffee (GC) powder (3 g/day)	Overweight or obese T2D patients	Increase in SIRT1 concentration	HbA1c, insulin level, IR, and TG level	Chlorogenic acid-mediated SIRT1 activation
	IRCT201206144010N8 [299]	Pomegranate juice (250 mL daily)	T2D patients	SIRT1 was significantly higher in PJ group	SIRT1 in the PBMC and inflammatory markers	Pomegranate polyphenol-induced SIRT1 expression
	NCT00692237 [300]	Sildenafil (100 mg/day)	T2D patients	Upregulation of SIRT1 in serum and subcutaneous fat	Anthropometric and metabolic parameters	Modulation of miR-22-3p targeting SIRT1
	NCT02244879 [301]	Resveratrol (500 mg/day)	T2D patients	Increased SIRT1 was associated with H3K56ac reduction	SIRT1 level and variation in H3K56ac	SIRT1-catalyzed histone deacetylation (H3K56ac)
Prediabetes	NCT01765946 [302]	Metformin (1500 mg/day)	Prediabetic patients	Metformin increased SIRT1 expression	AMPK activation and SIRT1 promoter accessibility	AMPK-dependent activation of SIRT1 signaling

Diseases	Trial ID	Intervention	Participant	SIRT-related findings	Outcomes	Primary mechanism of action
Prediabetes	NCT03439592 [303]	Hypocaloric diet + Metformin	Obese with pre-diabetic condition	Inverse correlation between SIRT1 and myocardial performance	SIRT1 levels in subcutaneous abdominal fat	Synergistic effect on energy-sensing SIRT1-AMPK axis
Cardiometabolic &Aging	DRKS00014322 [304]	Exercise circuit training	Healthy elderly participants	Activity of SIRT1 and SIRT3 increased	SIRT activities (SIRT1, SIRT3, SIRT5) in blood	Exercise-induced mitochondrial and metabolic stress
	IRCT20161102030649N1 [305]	Crocetin (10 mg/d)	CAD patients	Expression of SIRT1 gene statistically changed	SIRT1, LOX1, ICAM1 and MCP-1 expression	SIRT1-mediated anti-inflammatory/anti-atherogenic effect
	IRCT201512102017N26 [306]	Crocin (30 mg/d)	CAD patients	Beneficial effect by increasing SIRT1 and AMPK genes	Gene expression of SIRT1 and AMPK	Upregulation of SIRT1-AMPK lipid management axis
	NCT00429195 [296]	High-MUFA and LF-HCC diets	MetS patients	Produced higher SIRT1 mRNA levels	Advanced glycation and SIRT1 expression	Dietary fat quality modulation of SIRT1 expression
	NCT02011906 [307]	n-3 fatty acids + Vitamin E	CAD patients	SIRT1 and PGC-1α gene increased significantly	SIRT1 and PGC-1α gene expression	Enhancement of mitochondrial biogenesis via SIRT1
	NCT01031108 [266]	SRT2104 (SIRT1 activator)	Healthy smokers and T2D patients	Improved arterial compliance and arterial pressure	Pulse wave analysis and blood pressure	Pharmacological activation of vascular SIRT1
Autoimmune Diseases	CTRI/2017/05/008589 [308]	Yoga practice (5 times a week)	Rheumatoid Arthritis (RA)	SIRT1 mRNA expression levels not statistically different	Disease activity and functional status	Mind-body stress reduction affecting immuno-metabolism
	IRCT20091114002709N51 [309]	Selenomethionine (200g/day)	Ulcerative Colitis (UC)	Significant increase in SIRT1 gene expression	Expression of SIRT1 and PGC-1αgenes	Antioxidant response and mitochondrial biogenesis
	REF/2016/01/010500 [310]	Yogic practices	Rheumatoid Arthritis (RA)	Increased levels of SIRT1	Psycho-neuro-immune axis markers	Modulation of neuroendocrine-immune SIRT1 axis
	NCT01453491 [311]	SRT2104 (SIRT1 activator)	Mild to moderate UC	Did not demonstrate significant clinical activity	Safety and clinical activity of SRT2104	Pharmacological SIRT1 activation (Insufficient efficacy)

Diseases	Trial ID	Intervention	Participant	SIRT-related findings	Outcomes	Primary mechanism of action
Cancer/	NCT00691210 [312]	Vorinostat + Niacinamide	Lymphoma patients	Synergistic cytotoxicity and acetylation of Bcl6 and p53	MTD and dose-limiting toxicity	Inhibition of sirtuin deacetylases to promote tumor apoptosis
Tumor Metabolism	REF/2014/09/007532 [313]	YBLI program (Yoga)	Parents of retinoblastoma patients	Led to a significant increase in SIRT1	SIRT1 levels and psychological stress	Stress-induced reduction of SIRT1-mediated neuro-protection

References

1. Banks, A.S., N. Kon, C. Knight, et al., "Sirt1 Gain of Function Increases Energy Efficiency and Prevents Diabetes in Mice,"Cell Metabolism 8 (2008): 333-41
2. Bordone, L., D. Cohen, A. Robinson, et al., "Sirt1 Transgenic Mice Show Phenotypes Resembling Calorie Restriction,"Aging Cell 6 (2007): 759-67
3. North, B.J., M.A. Rosenberg, K.B. Jeganathan, et al., "Sirt2 Induces the Checkpoint Kinase Bubr1 to Increase Lifespan,"The EMBO Journal 33 (2014): 1438-53
4. Brown, Kevin D., S. Maqsood, J.-Y. Huang, et al., "Activation of Sirt3 by the Nad+ Precursor Nicotinamide Riboside Protects from Noise-Induced Hearing Loss,"Cell Metabolism 20 (2014): 1059-68
5. Dikalova, A.E., A. Pandey, L. Xiao, et al., "Mitochondrial Deacetylase Sirt3 Reduces Vascular Dysfunction and Hypertension While Sirt3 Depletion in Essential Hypertension Is Linked to Vascular Inflammation and Oxidative Stress,"Circulation Research 126 (2020): 439-52
6. Ogura, M., Y. Nakamura, D. Tanaka, et al., "Overexpression of Sirt5 Confirms Its Involvement in Deacetylation and Activation of Carbamoyl Phosphate Synthetase 1,"Biochem Biophys Res Commun 393 (2010): 73-8
7. Bentley, N.L., C.E. Fiveash, B. Osborne, et al., "Protein Hypoacylation Induced by Sirt5 Overexpression Has Minimal Metabolic Effect in Mice,"Biochemical and Biophysical Research Communications 503 (2018): 1349-55
8. Kanfi, Y., V. Peshti, R. Gil, et al., "Sirt6 Protects against Pathological Damage Caused by Diet-Induced Obesity,"Aging Cell 9 (2010): 162-73
9. Mcburney, M.W., X. Yang, K. Jardine, et al., "The Mammalian Sir2α Protein Has a Role in Embryogenesis and Gametogenesis,"Molecular and Cellular Biology 23 (2003): 38-54
10. Han, C., P. Linser, H.-J. Park, et al., "Sirt1 Deficiency Protects Cochlear Cells and Delays the Early Onset of Age-Related Hearing Loss in C57bl/6 Mice,"Neurobiology of Aging 43 (2016): 58-71
11. Gabay, O., K.J. Zaal, C. Sanchez, et al., "Sirt1-Deficient Mice Exhibit an Altered Cartilage Phenotype,"Joint Bone Spine 80 (2013): 613-20
12. Seifert, E.L., A.Z. Caron, K. Morin, et al., "Sirt1 Catalytic Activity Is Required for Male Fertility and Metabolic Homeostasis in Mice,"The FASEB Journal 26 (2011): 555-66

13. Hernández-Jiménez, M., O. Hurtado, M.I. Cuartero, et al., "Silent Information Regulator 1 Protects the Brain against Cerebral Ischemic Damage,"Stroke 44 (2013): 2333-37
14. Di Sante, G., L. Wang, C. Wang, et al., "Sirt1-Deficient Mice Have Hypogonadotropic Hypogonadism Due to Defective Gnrh Neuronal Migration,"Molecular Endocrinology 29 (2015): 200-12
15. Cheng, H.L., R. Mostoslavsky, S. Saito, et al., "Developmental Defects and P53 Hyperacetylation in Sir2 Homolog (Sirt1)-Deficient Mice,"Proc Natl Acad Sci U S A 100 (2003): 10794-9
16. Sulaiman, M., M.J. Matta, N.R. Sunderesan, et al., "Resveratrol, an Activator of Sirt1, Upregulates Sarcoplasmic Calcium Atpase and Improves Cardiac Function in Diabetic Cardiomyopathy,"American Journal of Physiology-Heart and Circulatory Physiology 298 (2010): H833-H43
17. Alvarez, Y., M. Rodríguez, C. Municio, et al., "Sirtuin 1 Is a Key Regulator of the Interleukin-12 P70/Interleukin-23 Balance in Human Dendritic Cells,"Journal of Biological Chemistry 287 (2012): 35689-701
18. Bellin, A.R., Y. Zhang, K. Thai, et al., "Impaired Sirt1 Activity Leads to Diminution in Glomerular Endowment without Accelerating Age-Associated Gfr Decline,"Physiological Reports 7 (2019): e14044
19. Kim, H.-S., A. Vassilopoulos, R.-H. Wang, et al., "Sirt2 Maintains Genome Integrity and Suppresses Tumorigenesis through Regulating Apc/C Activity,"Cancer Cell 20 (2011): 487-99
20. Serrano, L., P. Martínez-Redondo, A. Marazuela-Duque, et al., "The Tumor Suppressor Sirt2 Regulates Cell Cycle Progression and Genome Stability by Modulating the Mitotic Deposition of H4k20 Methylation,"Genes Dev 27 (2013): 639-53
21. Hamaidi, I., L. Zhang, N. Kim, et al., "Sirt2 Inhibition Enhances Metabolic Fitness and Effector Functions of Tumor-Reactive T Cells,"Cell Metabolism 32 (2020): 420-36.e12
22. Lombard, D.B., F.W. Alt, H.-L. Cheng, et al., "Mammalian Sir2 Homolog Sirt3 Regulates Global Mitochondrial Lysine Acetylation,"Molecular and Cellular Biology 27 (2007): 8807-14

23. Ahn, B.H., H.S. Kim, S. Song, et al., "A Role for the Mitochondrial Deacetylase Sirt3 in Regulating Energy Homeostasis,"Proc Natl Acad Sci U S A 105 (2008): 14447-52
24. Haigis, M.C., R. Mostoslavsky, K.M. Haigis, et al., "Sirt4 Inhibits Glutamate Dehydrogenase and Opposes the Effects of Calorie Restriction in Pancreatic Beta Cells,"Cell 126 (2006): 941-54
25. Anderson, K.A., F.K. Huynh, K. Fisher-Wellman, et al., "Sirt4 Is a Lysine Deacylase That Controls Leucine Metabolism and Insulin Secretion,"Cell Metabolism 25 (2017): 838-55.e15
26. Laurent, G., Natalie J. German, Asish K. Saha, et al., "Sirt4 Coordinates the Balance between Lipid Synthesis and Catabolism by Repressing Malonyl Coa Decarboxylase,"Molecular Cell 50 (2013): 686-98
27. Wang, Y.S., L. Du, X. Liang, et al., "Sirtuin 4 Depletion Promotes Hepatocellular Carcinoma Tumorigenesis through Regulating Adenosine-Monophosphate–Activated Protein Kinase Alpha/Mammalian Target of Rapamycin Axis in Mice,"Hepatology 69 (2019): 1614-31
28. Jeong, S.M., A. Lee, J. Lee, et al., "Sirt4 Protein Suppresses Tumor Formation in Genetic Models of Myc-Induced B Cell Lymphoma,"Journal of Biological Chemistry 289 (2014): 4135-44
29. Nakagawa, T., D.J. Lomb, M.C. Haigis, et al., "Sirt5 Deacetylates Carbamoyl Phosphate Synthetase 1 and Regulates the Urea Cycle,"Cell 137 (2009): 560-70
30. Mostoslavsky, R., K.F. Chua, D.B. Lombard, et al., "Genomic Instability and Aging-Like Phenotype in the Absence of Mammalian Sirt6,"Cell 124 (2006): 315-29
31. Greiten, L.E., B. Zhang, C.M. Roos, et al., "Sirtuin 6 Protects against Oxidative Stress and Vascular Dysfunction in Mice,"Front Physiol 12 (2021): 753501
32. Becherini, P., I. Caffa, F. Piacente, et al., "Sirt6 Enhances Oxidative Phosphorylation in Breast Cancer and Promotes Mammary Tumorigenesis in Mice,"Cancer & Metabolism 9 (2021):
33. Pillai, V.B., S. Samant, S. Hund, et al., "The Nuclear Sirtuin Sirt6 Protects the Heart from Developing Aging-Associated Myocyte Senescence and Cardiac Hypertrophy,"Aging (Albany NY) 13 (2021): 12334-58
34. Vakhrusheva, O., C. Smolka, P. Gajawada, et al., "Sirt7 Increases Stress Resistance of Cardiomyocytes and Prevents Apoptosis and Inflammatory Cardiomyopathy in Mice,"Circulation Research 102 (2008): 703-10

35. Shin, J., M. He, Y. Liu, et al., "Sirt7 Represses Myc Activity to Suppress Er Stress and Prevent Fatty Liver Disease,"Cell Rep 5 (2013): 654-65

36. Vazquez, B.N., J.K. Thackray, N.G. Simonet, et al., "Sirt 7 Promotes Genome Integrity and Modulates Non-Homologous End Joining DNA Repair,"The EMBO Journal 35 (2016): 1488-503

37. Yang, H.Y., F.Z. Lin, H.W. Yang, et al., "The Effect of Sirt1 Deficiency on Ca(2+) and Na(+) Regulation in Mouse Ventricular Myocytes,"J Cell Mol Med 24 (2020): 6762-72

38. Jin, X., Y. Zhang, Y. Zhou, et al., "Sirt1 Deficiency Promotes Age-Related Af through Enhancing Atrial Necroptosis by Activation of Ripk1 Acetylation,"Circ Arrhythm Electrophysiol 17 (2024): e012452

39. Chen, H.X., X.C. Wang, H.T. Hou, et al., "Lysine Crotonylation of Serca2a Correlates to Cardiac Dysfunction and Arrhythmia in Sirt1 Cardiac-Specific Knockout Mice,"Int J Biol Macromol 242 (2023): 125151

40. Sanz, M.N., L. Grimbert, M. Moulin, et al., "Inducible Cardiac-Specific Deletion of Sirt1 in Male Mice Reveals Progressive Cardiac Dysfunction and Sensitization of the Heart to Pressure Overload,"Int J Mol Sci 20 (2019):

41. Alcendor, R.R., S. Gao, P. Zhai, et al., "Sirt1 Regulates Aging and Resistance to Oxidative Stress in the Heart,"Circ Res 100 (2007): 1512-21

42. Benigni, A., P. Cassis, S. Conti, et al., "Sirt3 Deficiency Shortens Life Span and Impairs Cardiac Mitochondrial Function Rescued by Opa1 Gene Transfer,"Antioxid Redox Signal 31 (2019): 1255-71

43. Koentges, C., K. Pfeil, T. Schnick, et al., "Sirt3 Deficiency Impairs Mitochondrial and Contractile Function in the Heart,"Basic Res Cardiol 110 (2015): 36

44. Hsu, Y.J., S.C. Hsu, C.P. Hsu, et al., "Sirtuin 1 Protects the Aging Heart from Contractile Dysfunction Mediated through the Inhibition of Endoplasmic Reticulum Stress-Mediated Apoptosis in Cardiac-Specific Sirtuin 1 Knockout Mouse Model,"Int J Cardiol 228 (2017): 543-52

45. Pires Da Silva, J., K. Monceaux, A. Guilbert, et al., "Sirt1 Protects the Heart from Er Stress-Induced Injury by Promoting Eef2k/Eef2-Dependent Autophagy,"Cells 9 (2020):

46. Palomer, X., M.S. Roman-Azcona, J. Pizarro-Delgado, et al., "Sirt3-Mediated Inhibition of Fos through Histone H3 Deacetylation Prevents Cardiac Fibrosis and Inflammation,"Signal Transduct Target Ther 5 (2020): 14

47. Su, H., A.C. Cantrell, J.X. Chen, et al., "Sirt3 Deficiency Enhances Ferroptosis and Promotes Cardiac Fibrosis Via P53 Acetylation,"Cells 12 (2023):
48. Chen, T., J. Li, J. Liu, et al., "Activation of Sirt3 by Resveratrol Ameliorates Cardiac Fibrosis and Improves Cardiac Function Via the Tgf-Beta/Smad3 Pathway,"Am J Physiol Heart Circ Physiol 308 (2015): H424-34
49. Planavila, A., E. Dominguez, M. Navarro, et al., "Dilated Cardiomyopathy and Mitochondrial Dysfunction in Sirt1-Deficient Mice: A Role for Sirt1-Mef2 in Adult Heart,"J Mol Cell Cardiol 53 (2012): 521-31
50. Sarikhani, M., S. Maity, S. Mishra, et al., "Sirt2 Deacetylase Represses Nfat Transcription Factor to Maintain Cardiac Homeostasis,"J Biol Chem 293 (2018): 5281-94
51. Zhao, Y., Z. Lu, H. Zhang, et al., "Sodium-Glucose Exchanger 2 Inhibitor Canagliflozin Promotes Mitochondrial Metabolism and Alleviates Salt-Induced Cardiac Hypertrophy Via Preserving Sirt3 Expression,"J Adv Res 70 (2025): 255-69
52. Sundaresan, N.R., P. Vasudevan, L. Zhong, et al., "The Sirtuin Sirt6 Blocks Igf-Akt Signaling and Development of Cardiac Hypertrophy by Targeting C-Jun,"Nat Med 18 (2012): 1643-50
53. Yamamura, S., Y. Izumiya, S. Araki, et al., "Cardiomyocyte Sirt (Sirtuin) 7 Ameliorates Stress-Induced Cardiac Hypertrophy by Interacting with and Deacetylating Gata4,"Hypertension 75 (2020): 98-108
54. Vakhrusheva, O., C. Smolka, P. Gajawada, et al., "Sirt7 Increases Stress Resistance of Cardiomyocytes and Prevents Apoptosis and Inflammatory Cardiomyopathy in Mice,"Circ Res 102 (2008): 703-10
55. Gaul, D.S., J. Weber, L.J. Van Tits, et al., "Loss of Sirt3 Accelerates Arterial Thrombosis by Increasing Formation of Neutrophil Extracellular Traps and Plasma Tissue Factor Activity,"Cardiovasc Res 114 (2018): 1178-88
56. He, X., H. Zeng, and J.X. Chen, "Ablation of Sirt3 Causes Coronary Microvascular Dysfunction and Impairs Cardiac Recovery Post Myocardial Ischemia,"Int J Cardiol 215 (2016): 349-57
57. Zhou, B., M. Xiao, H. Hu, et al., "Cardioprotective Role of Sirt5 in Response to Acute Ischemia through a Novel Liver-Cardiac Crosstalk Mechanism,"Front Cell Dev Biol 9 (2021): 687559
58. Wang, L., N. Quan, W. Sun, et al., "Cardiomyocyte-Specific Deletion of Sirt1 Gene Sensitizes Myocardium to Ischaemia and Reperfusion Injury,"Cardiovasc Res 114 (2018): 805-21
59. Han, Y., W. Sun, D. Ren, et al., "Sirt1 Agonism Modulates Cardiac Nlrp3 Inflammasome through Pyruvate Dehydrogenase During Ischemia and Reperfusion,"Redox Biol 34 (2020): 101538

60. Yamamoto, T., K. Tamaki, K. Shirakawa, et al., "Cardiac Sirt1 Mediates the Cardioprotective Effect of Caloric Restriction by Suppressing Local Complement System Activation after Ischemia-Reperfusion,"Am J Physiol Heart Circ Physiol 310 (2016): H1003-14
61. Bochaton, T., C. Crola-Da-Silva, B. Pillot, et al., "Inhibition of Myocardial Reperfusion Injury by Ischemic Postconditioning Requires Sirtuin 3-Mediated Deacetylation of Cyclophilin D,"J Mol Cell Cardiol 84 (2015): 61-9
62. Araki, S., Y. Izumiya, T. Rokutanda, et al., "Sirt7 Contributes to Myocardial Tissue Repair by Maintaining Transforming Growth Factor-Beta Signaling Pathway,"Circulation 132 (2015): 1081-93
63. Wang, A.J., Y. Tang, J. Zhang, et al., "Cardiac Sirt1 Ameliorates Doxorubicin-Induced Cardiotoxicity by Targeting Sestrin 2,"Redox Biol 52 (2022): 102310
64. Pillai, V.B., S. Bindu, W. Sharp, et al., "Sirt3 Protects Mitochondrial DNA Damage and Blocks the Development of Doxorubicin-Induced Cardiomyopathy in Mice,"Am J Physiol Heart Circ Physiol 310 (2016): H962-72
65. Tomczyk, M.M., K.G. Cheung, B. Xiang, et al., "Mitochondrial Sirtuin-3 (Sirt3) Prevents Doxorubicin-Induced Dilated Cardiomyopathy by Modulating Protein Acetylation and Oxidative Stress,"Circ Heart Fail 15 (2022): e008547
66. Yang, Y., N. Li, T. Chen, et al., "Sirt3 Promotes Sensitivity to Sunitinib-Induced Cardiotoxicity Via Inhibition of Gtsp1/Jnk/Autophagy Pathway in Vivo and in Vitro,"Arch Toxicol 93 (2019): 3249-60
67. Wu, S., J. Lan, L. Li, et al., "Sirt6 Protects Cardiomyocytes against Doxorubicin-Induced Cardiotoxicity by Inhibiting P53/Fas-Dependent Cell Death and Augmenting Endogenous Antioxidant Defense Mechanisms,"Cell Biol Toxicol 39 (2023): 237-58
68. Gorski, P.A., S.P. Jang, D. Jeong, et al., "Role of Sirt1 in Modulating Acetylation of the Sarco-Endoplasmic Reticulum Ca(2+)-Atpase in Heart Failure,"Circ Res 124 (2019): e63-e80
69. Yuan, X., H. Qi, X. Li, et al., "Disruption of Spatiotemporal Hypoxic Signaling Causes Congenital Heart Disease in Mice,"J Clin Invest 127 (2017): 2235-48
70. Hu, Y., Y. Zheng, C. Liu, et al., "Mitochondrial Mof Regulates Energy Metabolism in Heart Failure Via Atp5b Hyperacetylation,"Cell Rep 43 (2024): 114839
71. Cantrell, A.C., J. Besanson, Q. Williams, et al., "Ferrostatin-1 Specifically Targets Mitochondrial Iron-Sulfur Clusters and Aconitase to Improve Cardiac Function in Sirtuin 3 Cardiomyocyte Knockout Mice,"J Mol Cell Cardiol 192 (2024): 36-47
72. Qian, K., J. Tang, Y.J. Ling, et al., "Exogenous Nadph Exerts a Positive Inotropic Effect and Enhances Energy Metabolism Via Sirt3 in Pathological Cardiac Hypertrophy and Heart Failure,"EBioMedicine 98 (2023): 104863

73. Machin, D.R., Y. Auduong, V.R. Gogulamudi, et al., "Lifelong Sirt-1 Overexpression Attenuates Large Artery Stiffening with Advancing Age,"Aging (Albany NY) 12 (2020): 11314-24
74. Fry, J.L., L. Al Sayah, R.M. Weisbrod, et al., "Vascular Smooth Muscle Sirtuin-1 Protects against Diet-Induced Aortic Stiffness,"Hypertension 68 (2016): 775-84
75. Shu, Y.N., L.H. Dong, H. Li, et al., "Ckii-Sirt1-Sm22alpha Loop Evokes a Self-Limited Inflammatory Response in Vascular Smooth Muscle Cells,"Cardiovasc Res 113 (2017): 1198-207
76. Zhang, Y., X. Wang, X.K. Li, et al., "Sirtuin 2 Deficiency Aggravates Ageing-Induced Vascular Remodelling in Humans and Mice,"Eur Heart J 44 (2023): 2746-59
77. Wen, L., Z. Chen, F. Zhang, et al., "Ca2+/Calmodulin-Dependent Protein Kinase Kinase Beta Phosphorylation of Sirtuin 1 in Endothelium Is Atheroprotective,"Proc Natl Acad Sci U S A 110 (2013): E2420-7
78. Zhang, Q.J., Z. Wang, H.Z. Chen, et al., "Endothelium-Specific Overexpression of Class Iii Deacetylase Sirt1 Decreases Atherosclerosis in Apolipoprotein E-Deficient Mice,"Cardiovasc Res 80 (2008): 191-9
79. Bai, B., Y. Liang, C. Xu, et al., "Cyclin-Dependent Kinase 5-Mediated Hyperphosphorylation of Sirtuin-1 Contributes to the Development of Endothelial Senescence and Atherosclerosis,"Circulation 126 (2012): 729-40
80. Stein, S., C. Lohmann, N. Schafer, et al., "Sirt1 Decreases Lox-1-Mediated Foam Cell Formation in Atherogenesis,"Eur Heart J 31 (2010): 2301-9
81. Cao, X., Y. Wu, H. Hong, et al., "Sirtuin 3 Dependent and Independent Effects of Nad(+) to Suppress Vascular Inflammation and Improve Endothelial Function in Mice,"Antioxidants (Basel) 11 (2022):
82. Liberale, L., D.S. Gaul, A. Akhmedov, et al., "Endothelial Sirt6 Blunts Stroke Size and Neurological Deficit by Preserving Blood-Brain Barrier Integrity: A Translational Study,"Eur Heart J 41 (2020): 1575-87
83. Lee, O.H., Y.M. Woo, S. Moon, et al., "Sirtuin 6 Deficiency Induces Endothelial Cell Senescence Via Downregulation of Forkhead Box M1 Expression,"Aging (Albany NY) 12 (2020): 20946-67
84. Fry, J.L., Y. Shiraishi, R. Turcotte, et al., "Vascular Smooth Muscle Sirtuin-1 Protects against Aortic Dissection During Angiotensin Ii-Induced Hypertension,"J Am Heart Assoc 4 (2015): e002384
85. Gao, P., T.T. Xu, J. Lu, et al., "Overexpression of Sirt1 in Vascular Smooth Muscle Cells Attenuates Angiotensin Ii-Induced Vascular Remodeling and Hypertension in Mice,"J Mol Med (Berl) 92 (2014): 347-57

86. Dikalova, A.E., A. Pandey, L. Xiao, et al., "Mitochondrial Deacetylase Sirt3 Reduces Vascular Dysfunction and Hypertension While Sirt3 Depletion in Essential Hypertension Is Linked to Vascular Inflammation and Oxidative Stress,"Circ Res 126 (2020): 439-52
87. Dikalova, A.E., H.A. Itani, R.R. Nazarewicz, et al., "Sirt3 Impairment and Sod2 Hyperacetylation in Vascular Oxidative Stress and Hypertension,"Circ Res 121 (2017): 564-74
88. Wei, T., G. Huang, J. Gao, et al., "Sirtuin 3 Deficiency Accelerates Hypertensive Cardiac Remodeling by Impairing Angiogenesis,"J Am Heart Assoc 6 (2017):
89. Zhang, C., N. Li, M. Suo, et al., "Sirtuin 3 Deficiency Aggravates Angiotensin Ii-Induced Hypertensive Cardiac Injury by the Impairment of Lymphangiogenesis,"J Cell Mol Med 25 (2021): 7760-71
90. Chen, H.Z., F. Wang, P. Gao, et al., "Age-Associated Sirtuin 1 Reduction in Vascular Smooth Muscle Links Vascular Senescence and Inflammation to Abdominal Aortic Aneurysm,"Circ Res 119 (2016): 1076-88
91. Guo, J., Z. Wang, J. Wu, et al., "Endothelial Sirt6 Is Vital to Prevent Hypertension and Associated Cardiorenal Injury through Targeting Nkx3.2-Gata5 Signaling,"Circ Res 124 (2019): 1448-61
92. Ding, Y.N., T.T. Wang, S.J. Lv, et al., "Sirt6 Is an Epigenetic Repressor of Thoracic Aortic Aneurysms Via Inhibiting Inflammation and Senescence,"Signal Transduct Target Ther 8 (2023): 255
93. Yang, L., X. Wu, S. Bian, et al., "Sirt6-Mediated Vascular Smooth Muscle Cells Senescence Participates in the Pathogenesis of Abdominal Aortic Aneurysm,"Atherosclerosis 392 (2024): 117483
94. Yu, X.T., N. Zhao, Y.T. Ma, et al., "Sirtuin 6 Mitigates Thoracic Aortic Aneurysm Progression Via Maintenance of Mitochondria Homeostasis in Vascular Smooth Muscle Cells,"Acta Pharmacol Sin 47 (2026): 103-18
95. Li, X., S. Zhang, G. Blander, et al., "Sirt1 Deacetylates and Positively Regulates the Nuclear Receptor Lxr,"Mol Cell 28 (2007): 91-106
96. Potente, M., L. Ghaeni, D. Baldessari, et al., "Sirt1 Controls Endothelial Angiogenic Functions During Vascular Growth,"Genes Dev 21 (2007): 2644-58
97. Zhou, S., H.Z. Chen, Y.Z. Wan, et al., "Repression of P66shc Expression by Sirt1 Contributes to the Prevention of Hyperglycemia-Induced Endothelial Dysfunction,"Circ Res 109 (2011): 639-48
98. Bulvik, R., R. Breuer, M. Dvir-Ginzberg, et al., "Sirt1 Deficiency, Specifically in Fibroblasts, Decreases Apoptosis Resistance and Is Associated with Resolution of Lung-Fibrosis,"Biomolecules 10 (2020):

99. Jablonski, R.P., S.J. Kim, P. Cheresh, et al., "Sirt3 Deficiency Promotes Lung Fibrosis by Augmenting Alveolar Epithelial Cell Mitochondrial DNA Damage and Apoptosis,"FASEB J 31 (2017): 2520-32
100. Cheresh, P., S.J. Kim, R. Jablonski, et al., "Sirt3 Overexpression Ameliorates Asbestos-Induced Pulmonary Fibrosis, Mt-DNA Damage, and Lung Fibrogenic Monocyte Recruitment,"Int J Mol Sci 22 (2021):
101. Hwang, J.W., S. Chung, I.K. Sundar, et al., "Cigarette Smoke-Induced Autophagy Is Regulated by Sirt1-Parp-1-Dependent Mechanism: Implication in Pathogenesis of Copd,"Arch Biochem Biophys 500 (2010): 203-9
102. Yao, H., S. Chung, J.W. Hwang, et al., "Sirt1 Protects against Emphysema Via Foxo3-Mediated Reduction of Premature Senescence in Mice,"J Clin Invest 122 (2012): 2032-45
103. Legutko, A., T. Marichal, L. Fievez, et al., "Sirtuin 1 Promotes Th2 Responses and Airway Allergy by Repressing Peroxisome Proliferator-Activated Receptor-Gamma Activity in Dendritic Cells,"J Immunol 187 (2011): 4517-29
104. Lai, T., G. Su, D. Wu, et al., "Myeloid-Specific Sirt1 Deletion Exacerbates Airway Inflammatory Response in a Mouse Model of Allergic Asthma,"Aging (Albany NY) 13 (2021): 15479-90
105. Lee, Y.G., B.F. Reader, D. Herman, et al., "Sirtuin 2 Enhances Allergic Asthmatic Inflammation,"JCI Insight 4 (2019):
106. Elesela, S., S.B. Morris, S. Narayanan, et al., "Sirtuin 1 Regulates Mitochondrial Function and Immune Homeostasis in Respiratory Syncytial Virus Infected Dendritic Cells,"PLoS Pathog 16 (2020): e1008319
107. Owczarczyk, A.B., M.A. Schaller, M. Reed, et al., "Sirtuin 1 Regulates Dendritic Cell Activation and Autophagy During Respiratory Syncytial Virus-Induced Immune Responses,"J Immunol 195 (2015): 1637-46
108. Cheng, C.Y., N.M. Gutierrez, M.B. Marzuki, et al., "Host Sirtuin 1 Regulates Mycobacterial Immunopathogenesis and Represents a Therapeutic Target against Tuberculosis,"Sci Immunol 2 (2017):
109. Smulan, L.J., N. Martinez, M.C. Kiritsy, et al., "Sirtuin 3 Downregulation in Mycobacterium Tuberculosis-Infected Macrophages Reprograms Mitochondrial Metabolism and Promotes Cell Death,"mBio 12 (2021):
110. Kim, Y.J., S.H. Lee, S.M. Jeon, et al., "Sirtuin 3 Is Essential for Host Defense against Mycobacterium Abscessus Infection through Regulation of Mitochondrial Homeostasis,"Virulence 11 (2020): 1225-39
111. Bai, X., Y. Liu, J. Liu, et al., "Adscs-Derived Exosomes Suppress Macrophage Ferroptosis Via the Sirt1/Nrf2 Signaling Axis to Alleviate Acute Lung Injury in Sepsis,"Int Immunopharmacol 146 (2025): 113914

112. Kurundkar, D., A.R. Kurundkar, N.B. Bone, et al., "Sirt3 Diminishes Inflammation and Mitigates Endotoxin-Induced Acute Lung Injury,"JCI Insight 4 (2019):
113. Wang, J., W. Li, F. Zhao, et al., "Sirt3 Regulates Nlrp3 and Participates in the Effects of Plantainoside D on Acute Lung Injury Sepsis,"Aging (Albany NY) 15 (2023): 6710-20
114. Peng, K., Y.X. Yao, X. Lu, et al., "Mitochondrial Dysfunction-Associated Alveolar Epithelial Senescence Is Involved in Cdcl(2)-Induced Copd-Like Lung Injury,"J Hazard Mater 476 (2024): 135103
115. Li, Y., K. Wong, A. Giles, et al., "Hepatic Sirt1 Attenuates Hepatic Steatosis and Controls Energy Balance in Mice by Inducing Fibroblast Growth Factor 21,"Gastroenterology 146 (2014): 539-49 e7
116. Park, S., M.J. Chung, J.Y. Son, et al., "The Role of Sirtuin 2 in Sustaining Functional Integrity of the Liver,"Life Sci 285 (2021): 119997
117. Li, S., X. Dou, H. Ning, et al., "Sirtuin 3 Acts as a Negative Regulator of Autophagy Dictating Hepatocyte Susceptibility to Lipotoxicity,"Hepatology 66 (2017): 936-52
118. Anderson, K.A., F.K. Huynh, K. Fisher-Wellman, et al., "Sirt4 Is a Lysine Deacylase That Controls Leucine Metabolism and Insulin Secretion,"Cell Metab 25 (2017): 838-55 e15
119. Lankisch, P.G., H. Buschmann-Kaspari, J. Otto, et al., "Correlation of Pancreatic Enzyme Levels with the Patient's Recovery from Acute Edematous Pancreatitis,"Klin Wochenschr 68 (1990): 565-9
120. Ho, L., A.S. Titus, K.K. Banerjee, et al., "Sirt4 Regulates Atp Homeostasis and Mediates a Retrograde Signaling Via Ampk,"Aging (Albany NY) 5 (2013): 835-49
121. Laurent, G., V.C. De Boer, L.W. Finley, et al., "Sirt4 Represses Peroxisome Proliferator-Activated Receptor Alpha Activity to Suppress Hepatic Fat Oxidation,"Mol Cell Biol 33 (2013): 4552-61
122. Huynh, F.K., X. Hu, Z. Lin, et al., "Loss of Sirtuin 4 Leads to Elevated Glucose- and Leucine-Stimulated Insulin Levels and Accelerated Age-Induced Insulin Resistance in Multiple Murine Genetic Backgrounds,"J Inherit Metab Dis 41 (2018): 59-72
123. Bhatt, D.P., C.A. Mills, K.A. Anderson, et al., "Deglutarylation of Glutaryl-Coa Dehydrogenase by Deacylating Enzyme Sirt5 Promotes Lysine Oxidation in Mice,"J Biol Chem 298 (2022): 101723
124. Park, J., Y. Chen, D.X. Tishkoff, et al., "Sirt5-Mediated Lysine Desuccinylation Impacts Diverse Metabolic Pathways,"Mol Cell 50 (2013): 919-30

125. Goetzman, E.S., S.S. Bharathi, Y. Zhang, et al., "Impaired Mitochondrial Medium-Chain Fatty Acid Oxidation Drives Periportal Macrovesicular Steatosis in Sirtuin-5 Knockout Mice,"Sci Rep 10 (2020): 18367
126. Du, Y., H. Hu, S. Qu, et al., "Sirt5 Deacylates Metabolism-Related Proteins and Attenuates Hepatic Steatosis in Ob/Ob Mice,"EBioMedicine 36 (2018): 347-57
127. Zhu, C., M. Huang, H.G. Kim, et al., "Sirt6 Controls Hepatic Lipogenesis by Suppressing Lxr, Chrebp, and Srebp1,"Biochim Biophys Acta Mol Basis Dis 1867 (2021): 166249
128. Hou, T., Y. Tian, Z. Cao, et al., "Cytoplasmic Sirt6-Mediated Acsl5 Deacetylation Impedes Nonalcoholic Fatty Liver Disease by Facilitating Hepatic Fatty Acid Oxidation,"Mol Cell 82 (2022): 4099-115 e9
129. Li, F., L. Zhang, H. Xue, et al., "Sirt1 Alleviates Hepatic Ischemia-Reperfusion Injury Via the Mir-182-Mediated Xbp1/Nlrp3 Pathway,"Mol Ther Nucleic Acids 23 (2021): 1066-77
130. Adjei-Mosi, J., Q. Sun, S.B. Smithson, et al., "Age-Dependent Loss of Hepatic Sirt1 Enhances Nlrp3 Inflammasome Signaling and Impairs Capacity for Liver Fibrosis Resolution,"Aging Cell 22 (2023): e13811
131. Cui, X., Q. Chen, Z. Dong, et al., "Inactivation of Sirt1 in Mouse Livers Protects against Endotoxemic Liver Injury by Acetylating and Activating Nf-Kappab,"Cell Death Dis 7 (2016): e2403
132. Wang, J., H.W. Koh, L. Zhou, et al., "Sirtuin 2 Aggravates Postischemic Liver Injury by Deacetylating Mitogen-Activated Protein Kinase Phosphatase-1,"Hepatology 65 (2017): 225-36
133. Coleman, M.C., A.K. Olivier, J.A. Jacobus, et al., "Superoxide Mediates Acute Liver Injury in Irradiated Mice Lacking Sirtuin 3,"Antioxid Redox Signal 20 (2014): 1423-35
134. Zhao, S., J. Li, X. Xing, et al., "Oxyberberine Suppressed the Carbon Tetrachloride-Induced Liver Fibrosis by Inhibiting Liver Inflammation in a Sirtuin 3-Dependent Manner in Mice,"Int Immunopharmacol 116 (2023): 109876
135. Lobianco, F.V., K.J. Krager, G.S. Carter, et al., "The Role of Sirtuin 3 in Radiation-Induced Long-Term Persistent Liver Injury,"Antioxidants (Basel) 9 (2020):
136. Hao, L., I.H. Bang, J. Wang, et al., "Errgamma Suppression by Sirt6 Alleviates Cholestatic Liver Injury and Fibrosis,"JCI Insight 5 (2020):
137. Luo, J., H. Liu, Y. Xu, et al., "Hepatic Sirt6 Activation Abrogates Acute Liver Failure,"Cell Death Dis 15 (2024): 283
138. Ding, C., B. Liu, T. Yu, et al., "Sirt7 Protects against Liver Fibrosis by Suppressing Stellate Cell Activation Via Tgf-Beta/Smad2/3 Pathway,"Biomed Pharmacother 180 (2024): 117477

139. Wellman, A.S., M.R. Metukuri, N. Kazgan, et al., "Intestinal Epithelial Sirtuin 1 Regulates Intestinal Inflammation During Aging in Mice by Altering the Intestinal Microbiota,"Gastroenterology 153 (2017): 772-86
140. Wang, L., J. Li, M. Jiang, et al., "Sirt1 Stabilizes Beta-Trcp1 to Inhibit Snail1 Expression in Maintaining Intestinal Epithelial Integrity to Alleviate Colitis,"Cell Mol Gastroenterol Hepatol 18 (2024): 101354
141. Liu, X., Y. Song, M. Shen, et al., "Smooth Muscle Silent Information Regulator 1 Contributes to Colitis in Mice,"Int J Mol Sci 26 (2025):
142. Lo Sasso, G., K.J. Menzies, A. Mottis, et al., "Sirt2 Deficiency Modulates Macrophage Polarization and Susceptibility to Experimental Colitis,"PLoS One 9 (2014): e103573
143. Guo, J., J. Xu, L. Chen, et al., "Role of Sirt2 in Intestinal Barrier under Cold Exposure,"Life Sci 330 (2023): 121949
144. Li, C., Y. Zhou, P. Rychahou, et al., "Sirt2 Contributes to the Regulation of Intestinal Cell Proliferation and Differentiation,"Cell Mol Gastroenterol Hepatol 10 (2020): 43-57
145. Liu, F., H.F. Bu, H. Geng, et al., "Sirtuin-6 Preserves R-Spondin-1 Expression and Increases Resistance of Intestinal Epithelium to Injury in Mice,"Mol Med 23 (2017): 272-84
146. Su, X., L. Zhao, H. Zhang, et al., "Sirtuin 6 Inhibits Group 3 Innate Lymphoid Cell Function and Gut Immunity by Suppressing Il-22 Production,"Front Immunol 15 (2024): 1402834
147. Liu, Y., H. Cui, C. Mei, et al., "Sirtuin4 Alleviates Severe Acute Pancreatitis by Regulating Hif-1alpha/Ho-1 Mediated Ferroptosis,"Cell Death Dis 14 (2023): 694
148. Zhang, Q., P. Zhang, G.J. Qi, et al., "Cdk5 Suppression Blocks Sirt1 Degradation Via the Ubiquitin-Proteasome Pathway in Parkinson's Disease Models,"Biochim Biophys Acta Gen Subj 1862 (2018): 1443-51
149. Zhang, X., X. Ren, Q. Zhang, et al., "Pgc-1alpha/Erralpha-Sirt3 Pathway Regulates Daergic Neuronal Death by Directly Deacetylating Sod2 and Atp Synthase Beta,"Antioxid Redox Signal 24 (2016): 312-28
150. Shi, H., H.X. Deng, D. Gius, et al., "Sirt3 Protects Dopaminergic Neurons from Mitochondrial Oxidative Stress,"Hum Mol Genet 26 (2017): 1915-26
151. Liu, L., C. Peritore, J. Ginsberg, et al., "Protective Role of Sirt5 against Motor Deficit and Dopaminergic Degeneration in Mptp-Induced Mice Model of Parkinson's Disease,"Behav Brain Res 281 (2015): 215-21
152. Liu, L., A. Arun, L. Ellis, et al., "Sirt2 Enhances 1-Methyl-4-Phenyl-1,2,3,6-Tetrahydropyridine (Mptp)-Induced Nigrostriatal Damage Via Apoptotic Pathway,"Front Aging Neurosci 6 (2014): 184

153. Yan, J., P. Zhang, J. Tan, et al., "Cdk5 Phosphorylation-Induced Sirt2 Nuclear Translocation Promotes the Death of Dopaminergic Neurons in Parkinson's Disease,"NPJ Parkinsons Dis 8 (2022): 46
154. Michan, S., Y. Li, M.M. Chou, et al., "Sirt1 Is Essential for Normal Cognitive Function and Synaptic Plasticity,"J Neurosci 30 (2010): 9695-707
155. Silva, D.F., A.R. Esteves, C.R. Oliveira, et al., "Mitochondrial Metabolism Power Sirt2-Dependent Deficient Traffic Causing Alzheimer's-Disease Related Pathology,"Mol Neurobiol 54 (2017): 4021-40
156. Bai, N., N. Li, R. Cheng, et al., "Inhibition of Sirt2 Promotes App Acetylation and Ameliorates Cognitive Impairment in App/Ps1 Transgenic Mice,"Cell Rep 40 (2022): 111062
157. Cheng, A., J. Wang, N. Ghena, et al., "Sirt3 Haploinsufficiency Aggravates Loss of Gabaergic Interneurons and Neuronal Network Hyperexcitability in an Alzheimer's Disease Model,"J Neurosci 40 (2020): 694-709
158. Jiang, M., J. Wang, J. Fu, et al., "Neuroprotective Role of Sirt1 in Mammalian Models of Huntington's Disease through Activation of Multiple Sirt1 Targets,"Nat Med 18 (2011): 153-8
159. Bobrowska, A., G. Donmez, A. Weiss, et al., "Sirt2 Ablation Has No Effect on Tubulin Acetylation in Brain, Cholesterol Biosynthesis or the Progression of Huntington's Disease Phenotypes in Vivo,"PLoS One 7 (2012): e34805
160. Lei, Y., J. Wang, D. Wang, et al., "Sirt1 in Forebrain Excitatory Neurons Produces Sexually Dimorphic Effects on Depression-Related Behaviors and Modulates Neuronal Excitability and Synaptic Transmission in the Medial Prefrontal Cortex,"Mol Psychiatry 25 (2020): 1094-111
161. Zhang, Z., P. Zhang, G.J. Qi, et al., "Cdk5-Mediated Phosphorylation of Sirt2 Contributes to Depressive-Like Behavior Induced by Social Defeat Stress,"Biochim Biophys Acta Mol Basis Dis 1864 (2018): 533-41
162. Hu, K., H. Chen, Y. Gao, et al., "Astrocytic Sirt6 Is a Potential Anti-Depression and Anti-Anxiety Target,"Prog Neuropsychopharmacol Biol Psychiatry 123 (2023): 110702
163. Libert, S., K. Pointer, E.L. Bell, et al., "Sirt1 Activates Mao-a in the Brain to Mediate Anxiety and Exploratory Drive,"Cell 147 (2011): 1459-72
164. Kim, H.D., J. Wei, T. Call, et al., "Sirt1 Coordinates Transcriptional Regulation of Neural Activity and Modulates Depression-Like Behaviors in the Nucleus Accumbens,"Biol Psychiatry 96 (2024): 495-505

165. Kwok, A., B. Chaqour, R.S. Khan, et al., "Pharmacological Activation and Transgenic Overexpression of Sirt1 Attenuate Traumatic Optic Neuropathy Induced by Blunt Head Impact,"Transl Vis Sci Technol 13 (2024): 27
166. Ge, Y., X. Wu, Y. Cai, et al., "Fndc5 Prevents Oxidative Stress and Neuronal Apoptosis after Traumatic Brain Injury through Sirt3-Dependent Regulation of Mitochondrial Quality Control,"Cell Death Dis 15 (2024): 364
167. Jiang, T., T. Qin, P. Gao, et al., "Sirt1 Attenuates Blood-Spinal Cord Barrier Disruption after Spinal Cord Injury by Deacetylating P66shc,"Redox Biol 60 (2023): 102615
168. Wang, R.H., H.S. Kim, C. Xiao, et al., "Hepatic Sirt1 Deficiency in Mice Impairs Mtorc2/Akt Signaling and Results in Hyperglycemia, Oxidative Damage, and Insulin Resistance,"J Clin Invest 121 (2011): 4477-90
169. Liu, R., Y. Zhong, X. Li, et al., "Role of Transcription Factor Acetylation in Diabetic Kidney Disease,"Diabetes 63 (2014): 2440-53
170. Lantier, L., A.S. Williams, C.C. Hughey, et al., "Sirt2 Knockout Exacerbates Insulin Resistance in High Fat-Fed Mice,"PLoS One 13 (2018): e0208634
171. Guo, J., J. Nie, D. Li, et al., "The Role of Nad-Dependent Deacetylase Sirtuin-2 in Liver Metabolic Stress through Regulating Pyruvate Kinase M2 Ubiquitination,"J Transl Med 22 (2024): 656
172. Zhou, Y., A.C.K. Chung, R. Fan, et al., "Sirt3 Deficiency Increased the Vulnerability of Pancreatic Beta Cells to Oxidative Stress-Induced Dysfunction,"Antioxid Redox Signal 27 (2017): 962-76
173. Zhang, K., Y. Wang, Y. Sun, et al., "Sirtuin 3 Reinforces Acylcarnitine Metabolism and Maintains Thermogenesis in Brown Adipose Tissue of Aging Mice,"Aging Cell 23 (2024): e14332
174. Wang, W., J. Liang, Y. Zhang, et al., "Myeloid Sirtuin 6 Deficiency Causes Obesity in Mice by Inducing Norepinephrine Degradation to Limit Thermogenic Tissue Function,"Sci Signal 18 (2025): eadl6441
175. Cui, X., L. Yao, X. Yang, et al., "Sirt6 Regulates Metabolic Homeostasis in Skeletal Muscle through Activation of Ampk,"Am J Physiol Endocrinol Metab 313 (2017): E493-E505
176. Huynh, F.K., B.S. Peterson, K.A. Anderson, et al., "Beta-Cell-Specific Ablation of Sirtuin 4 Does Not Affect Nutrient-Stimulated Insulin Secretion in Mice,"Am J Physiol Endocrinol Metab 319 (2020): E805-E13
177. Zaganjor, E., H. Yoon, J.B. Spinelli, et al., "Sirt4 Is an Early Regulator of Branched-Chain Amino Acid Catabolism That Promotes Adipogenesis,"Cell Rep 36 (2021): 109345

178. Mizumoto, T., T. Yoshizawa, Y. Sato, et al., "Sirt7 Deficiency Protects against Aging-Associated Glucose Intolerance and Extends Lifespan in Male Mice,"Cells 11 (2022):
179. Yoshizawa, T., Y. Sato, S.U. Sobuz, et al., "Sirt7 Suppresses Energy Expenditure and Thermogenesis by Regulating Brown Adipose Tissue Functions in Mice,"Nat Commun 13 (2022): 7439
180. Purushotham, A., T.T. Schug, Q. Xu, et al., "Hepatocyte-Specific Deletion of Sirt1 Alters Fatty Acid Metabolism and Results in Hepatic Steatosis and Inflammation,"Cell Metab 9 (2009): 327-38
181. Shen, L., Q. Zhang, S. Tu, et al., "Sirt3 Mediates Mitofusin 2 Ubiquitination and Degradation to Suppress Ischemia Reperfusion-Induced Acute Kidney Injury,"Exp Cell Res 408 (2021): 112861
182. Sanchez-Navarro, A., M.A. Martinez-Rojas, A. Albarran-Godinez, et al., "Sirtuin 7 Deficiency Reduces Inflammation and Tubular Damage Induced by an Episode of Acute Kidney Injury,"Int J Mol Sci 23 (2022):
183. Jung, Y.J., A.S. Lee, T. Nguyen-Thanh, et al., "Sirt2 Regulates Lps-Induced Renal Tubular Cxcl2 and Ccl2 Expression,"J Am Soc Nephrol 26 (2015): 1549-60
184. Zhao, W.Y., L. Zhang, M.X. Sui, et al., "Protective Effects of Sirtuin 3 in a Murine Model of Sepsis-Induced Acute Kidney Injury,"Sci Rep 6 (2016): 33201
185. Jian, Y., Y. Yang, L. Cheng, et al., "Sirt3 Mitigates Lps-Induced Mitochondrial Damage in Renal Tubular Epithelial Cells by Deacetylating Yme1l1,"Cell Prolif 56 (2023): e13362
186. Guan, Y., S.R. Wang, X.Z. Huang, et al., "Nicotinamide Mononucleotide, an Nad(+) Precursor, Rescues Age-Associated Susceptibility to Aki in a Sirtuin 1-Dependent Manner,"J Am Soc Nephrol 28 (2017): 2337-52
187. Kume, S., T. Uzu, K. Horiike, et al., "Calorie Restriction Enhances Cell Adaptation to Hypoxia through Sirt1-Dependent Mitochondrial Autophagy in Mouse Aged Kidney,"J Clin Invest 120 (2010): 1043-55
188. Zhang, Q., X. Liu, N. Li, et al., "Sirtuin 3 Deficiency Aggravates Contrast-Induced Acute Kidney Injury,"J Transl Med 16 (2018): 313
189. Chiba, T., K.D. Peasley, K.R. Cargill, et al., "Sirtuin 5 Regulates Proximal Tubule Fatty Acid Oxidation to Protect against Aki,"J Am Soc Nephrol 30 (2019): 2384-98
190. Li, Z., K. Xu, N. Zhang, et al., "Overexpressed Sirt6 Attenuates Cisplatin-Induced Acute Kidney Injury by Inhibiting Erk1/2 Signaling,"Kidney Int 93 (2018): 881-92

191. Yang, S., L. Chen, S. Din, et al., "The Sirt6/Bap1/Xct Signaling Axis Mediates Ferroptosis in Cisplatin-Induced Aki,"Cell Signal 125 (2025): 111479
192. Jung, Y.J., W. Park, K.P. Kang, et al., "Sirt2 Is Involved in Cisplatin-Induced Acute Kidney Injury through Regulation of Mitogen-Activated Protein Kinase Phosphatase-1,"Nephrol Dial Transplant 35 (2020): 1145-56
193. Miyasato, Y., T. Yoshizawa, Y. Sato, et al., "Sirtuin 7 Deficiency Ameliorates Cisplatin-Induced Acute Kidney Injury through Regulation of the Inflammatory Response,"Sci Rep 8 (2018): 5927
194. Nguyen, L.T., C.H. Mak, H. Chen, et al., "Sirt1 Attenuates Kidney Disorders in Male Offspring Due to Maternal High-Fat Diet,"Nutrients 11 (2019):
195. Hasegawa, K., S. Wakino, P. Simic, et al., "Renal Tubular Sirt1 Attenuates Diabetic Albuminuria by Epigenetically Suppressing Claudin-1 Overexpression in Podocytes,"Nat Med 19 (2013): 1496-504
196. Chuang, P.Y., W. Cai, X. Li, et al., "Reduction in Podocyte Sirt1 Accelerates Kidney Injury in Aging Mice,"Am J Physiol Renal Physiol 313 (2017): F621-F28
197. Chen, L., D. Li, Z. Zhan, et al., "Sirtuin 2 Exacerbates Renal Tubule Injury and Inflammation in Diabetic Mice Via Deacetylation of C-Jun/C-Fos,"Cell Mol Life Sci 82 (2025): 45
198. Perico, L., M. Morigi, C. Rota, et al., "Human Mesenchymal Stromal Cells Transplanted into Mice Stimulate Renal Tubular Cells and Enhance Mitochondrial Function,"Nat Commun 8 (2017): 983
199. Yang, Q., J. Hu, Y. Yang, et al., "Sirt6 Deficiency Aggravates Angiotensin Ii-Induced Cholesterol Accumulation and Injury in Podocytes,"Theranostics 10 (2020): 7465-79
200. Li, W., W. Feng, X. Su, et al., "Sirt6 Protects Vascular Smooth Muscle Cells from Osteogenic Transdifferentiation Via Runx2 in Chronic Kidney Disease,"J Clin Invest 132 (2022):
201. Jin, J., W. Li, T. Wang, et al., "Loss of Proximal Tubular Sirtuin 6 Aggravates Unilateral Ureteral Obstruction-Induced Tubulointerstitial Inflammation and Fibrosis by Regulation of Beta-Catenin Acetylation,"Cells 11 (2022):
202. Li, P., Y. Liu, X. Qin, et al., "Sirt1 Attenuates Renal Fibrosis by Repressing Hif-2alpha,"Cell Death Discov 7 (2021): 59
203. Vasko, R., S. Xavier, J. Chen, et al., "Endothelial Sirtuin 1 Deficiency Perpetrates Nephrosclerosis through Downregulation of Matrix Metalloproteinase-14: Relevance to Fibrosis of Vascular Senescence,"J Am Soc Nephrol 25 (2014): 276-91

204. Iljas, J.D., Z. Wei, and H.A. Homer, "Sirt1 Sustains Female Fertility by Slowing Age-Related Decline in Oocyte Quality Required for Post-Fertilization Embryo Development,"Aging Cell 19 (2020): e13204
205. Lee, I.H., L. Cao, R. Mostoslavsky, et al., "A Role for the Nad-Dependent Deacetylase Sirt1 in the Regulation of Autophagy,"Proc Natl Acad Sci U S A 105 (2008): 3374-9
206. Iljas, J.D., and H.A. Homer, "Sirt3 Is Dispensable for Oocyte Quality and Female Fertility in Lean and Obese Mice,"FASEB J 34 (2020): 6641-53
207. Arul Nambi Rajan, K., M. Khater, F. Soncin, et al., "Sirtuin1 Is Required for Proper Trophoblast Differentiation and Placental Development in Mice,"Placenta 62 (2018): 1-8
208. Li, H., G.K. Rajendran, N. Liu, et al., "Sirt1 Modulates the Estrogen-Insulin-Like Growth Factor-1 Signaling for Postnatal Development of Mammary Gland in Mice,"Breast Cancer Res 9 (2007): R1
209. Xiong, L., X. Ye, Z. Chen, et al., "Advanced Maternal Age-Associated Sirt1 Deficiency Compromises Trophoblast Epithelial-Mesenchymal Transition through an Increase in Vimentin Acetylation,"Aging Cell 20 (2021): e13491
210. Liu, C., Z. Song, L. Wang, et al., "Sirt1 Regulates Acrosome Biogenesis by Modulating Autophagic Flux During Spermiogenesis in Mice,"Development 144 (2017): 441-51
211. Seifert, E.L., A.Z. Caron, K. Morin, et al., "Sirt1 Catalytic Activity Is Required for Male Fertility and Metabolic Homeostasis in Mice,"FASEB J 26 (2012): 555-66
212. Zheng, S., J. Jiang, Z. Shu, et al., "Fine Particulate Matter (Pm(2.5)) Induces Testosterone Disruption by Triggering Ferroptosis through Sirt1/Hif-1alpha Signaling Pathway in Male Mice,"Free Radic Biol Med 221 (2024): 40-51
213. Sun, W., W. Qiao, B. Zhou, et al., "Overexpression of Sirt1 in Mesenchymal Stem Cells Protects against Bone Loss in Mice by Foxo3a Deacetylation and Oxidative Stress Inhibition,"Metabolism 88 (2018): 61-71
214. Jin, X., X. Sun, X. Ma, et al., "Sirt1 Maintains Bone Homeostasis by Regulating Osteoblast Glycolysis through Got1,"Cell Mol Life Sci 81 (2024): 204
215. Louvet, L., D. Leterme, S. Delplace, et al., "Sirtuin 1 Deficiency Decreases Bone Mass and Increases Bone Marrow Adiposity in a Mouse Model of Chronic Energy Deficiency,"Bone 136 (2020): 115361
216. Wang, H., Z. Hu, J. Wu, et al., "Sirt1 Promotes Osteogenic Differentiation and Increases Alveolar Bone Mass Via Bmi1 Activation in Mice,"J Bone Miner Res 34 (2019): 1169-81

217. Kim, S.J., Y. Piao, M.G. Lee, et al., "Loss of Sirtuin 6 in Osteoblast Lineage Cells Activates Osteoclasts, Resulting in Osteopenia,"Bone 138 (2020): 115497

218. Moon, Y.J., Z. Zhang, I.H. Bang, et al., "Sirtuin 6 in Preosteoclasts Suppresses Age- and Estrogen Deficiency-Related Bone Loss by Stabilizing Estrogen Receptor Alpha,"Cell Death Differ 26 (2019): 2358-70

219. Ling, W., K. Krager, K.K. Richardson, et al., "Mitochondrial Sirt3 Contributes to the Bone Loss Caused by Aging or Estrogen Deficiency,"JCI Insight 6 (2021):

220. Richardson, K.K., G.O. Adam, W. Ling, et al., "Mitochondrial Protein Deacetylation by Sirt3 in Osteoclasts Promotes Bone Resorption with Aging in Female Mice,"Mol Metab 88 (2024): 102012

221. Batshon, G., J. Elayyan, O. Qiq, et al., "Serum Nt/Ct Sirt1 Ratio Reflects Early Osteoarthritis and Chondrosenescence,"Ann Rheum Dis 79 (2020): 1370-80

222. Gabay, O., C. Sanchez, M. Dvir-Ginzberg, et al., "Sirtuin 1 Enzymatic Activity Is Required for Cartilage Homeostasis in Vivo in a Mouse Model,"Arthritis Rheum 65 (2013): 159-66

223. Elayyan, J., I. Carmon, L. Zecharyahu, et al., "Lef1 Ablation Alleviates Cartilage Mineralization Following Posttraumatic Osteoarthritis Induction,"Proc Natl Acad Sci U S A 119 (2022): e2116855119

224. Fu, Y., M. Kinter, J. Hudson, et al., "Aging Promotes Sirtuin 3-Dependent Cartilage Superoxide Dismutase 2 Acetylation and Osteoarthritis,"Arthritis Rheumatol 68 (2016): 1887-98

225. Snyder-Warwick, A.K., A. Satoh, K.B. Santosa, et al., "Hypothalamic Sirt1 Protects Terminal Schwann Cells and Neuromuscular Junctions from Age-Related Morphological Changes,"Aging Cell 17 (2018): e12776

226. Myers, M.J., D.L. Shepherd, A.J. Durr, et al., "The Role of Sirt1 in Skeletal Muscle Function and Repair of Older Mice,"J Cachexia Sarcopenia Muscle 10 (2019): 929-49

227. Samant, S.A., A. Kanwal, V.B. Pillai, et al., "The Histone Deacetylase Sirt6 Blocks Myostatin Expression and Development of Muscle Atrophy,"Sci Rep 7 (2017): 11877

228. Jiang, C., J. Liu, M. Guo, et al., "The Nad-Dependent Deacetylase Sirt2 Regulates T Cell Differentiation Involved in Tumor Immune Response,"Int J Biol Sci 16 (2020): 3075-84

229. Du, L., X. Liu, Y. Ren, et al., "Loss of Sirt4 Promotes the Self-Renewal of Breast Cancer Stem Cells,"Theranostics 10 (2020): 9458-76

230. Becherini, P., I. Caffa, F. Piacente, et al., "Sirt6 Enhances Oxidative Phosphorylation in Breast Cancer and Promotes Mammary Tumorigenesis in Mice,"Cancer Metab 9 (2021): 6
231. Abril, Y.L.N., I.R. Fernandez, J.Y. Hong, et al., "Pharmacological and Genetic Perturbation Establish Sirt5 as a Promising Target in Breast Cancer,"Oncogene 40 (2021): 1644-58
232. Xie, M., M. Liu, and C.S. He, "Sirt1 Regulates Endothelial Notch Signaling in Lung Cancer,"PLoS One 7 (2012): e45331
233. Qiu, P., W. Hou, H. Wang, et al., "Sirt1 Deficiency Upregulates Glutathione Metabolism to Prevent Hepatocellular Carcinoma Initiation in Mice,"Oncogene 40 (2021): 6023-33
234. Wang, Y.S., L. Du, X. Liang, et al., "Sirtuin 4 Depletion Promotes Hepatocellular Carcinoma Tumorigenesis through Regulating Adenosine-Monophosphate-Activated Protein Kinase Alpha/Mammalian Target of Rapamycin Axis in Mice,"Hepatology 69 (2019): 1614-31
235. Xiang, J., N. Zhang, H. Sun, et al., "Disruption of Sirt7 Increases the Efficacy of Checkpoint Inhibitor Via Mef2d Regulation of Programmed Cell Death 1 Ligand 1 in Hepatocellular Carcinoma Cells,"Gastroenterology 158 (2020): 664-78 e24
236. Kim, H.S., A. Vassilopoulos, R.H. Wang, et al., "Sirt2 Maintains Genome Integrity and Suppresses Tumorigenesis through Regulating Apc/C Activity,"Cancer Cell 20 (2011): 487-99
237. Chen, X.F., M.X. Tian, R.Q. Sun, et al., "Sirt5 Inhibits Peroxisomal Acox1 to Prevent Oxidative Damage and Is Downregulated in Liver Cancer,"EMBO Rep 19 (2018):
238. Marquardt, J.U., K. Fischer, K. Baus, et al., "Sirtuin-6-Dependent Genetic and Epigenetic Alterations Are Associated with Poor Clinical Outcome in Hepatocellular Carcinoma Patients,"Hepatology 58 (2013): 1054-64
239. Pinho, A.V., A. Mawson, A. Gill, et al., "Sirtuin 1 Stimulates the Proliferation and the Expression of Glycolysis Genes in Pancreatic Neoplastic Lesions,"Oncotarget 7 (2016): 74768-78
240. Kugel, S., C. Sebastian, J. Fitamant, et al., "Sirt6 Suppresses Pancreatic Cancer through Control of Lin28b,"Cell 165 (2016): 1401-15
241. Powell, M.J., M.C. Casimiro, C. Cordon-Cardo, et al., "Disruption of a Sirt1-Dependent Autophagy Checkpoint in the Prostate Results in Prostatic Intraepithelial Neoplasia Lesion Formation,"Cancer Res 71 (2011): 964-75
242. Giblin, W., L. Bringman-Rodenbarger, A.H. Guo, et al., "The Deacylase Sirt5 Supports Melanoma Viability by Influencing Chromatin Dynamics,"J Clin Invest 131 (2021):
243. Choi, J.E., C. Sebastian, C.M. Ferrer, et al., "A Unique Subset of Glycolytic Tumour-Propagating Cells Drives Squamous Cell Carcinoma,"Nat Metab 3 (2021): 182-95

244. Wang, F., Z. Li, J. Zhou, et al., "Sirt1 Regulates the Phosphorylation and Degradation of P27 by Deacetylating Cdk2 to Promote T-Cell Acute Lymphoblastic Leukemia Progression,"J Exp Clin Cancer Res 40 (2021): 259

245. Abraham, A., S. Qiu, B.K. Chacko, et al., "Sirt1 Regulates Metabolism and Leukemogenic Potential in Cml Stem Cells,"J Clin Invest 129 (2019): 2685-701

246. Li, M., Y.L. Chiang, C.A. Lyssiotis, et al., "Non-Oncogene Addiction to Sirt3 Plays a Critical Role in Lymphomagenesis,"Cancer Cell 35 (2019): 916-31 e9

247. Jeong, S.M., A. Lee, J. Lee, et al., "Sirt4 Protein Suppresses Tumor Formation in Genetic Models of Myc-Induced B Cell Lymphoma,"J Biol Chem 289 (2014): 4135-44

248. Yang, H., S.M. Lee, B. Gao, et al., "Histone Deacetylase Sirtuin 1 Deacetylates Irf1 Protein and Programs Dendritic Cells to Control Th17 Protein Differentiation During Autoimmune Inflammation,"J Biol Chem 288 (2013): 37256-66

249. Zhang, W., D. Xiao, X. Li, et al., "Sirt1 Inactivation Switches Reactive Astrocytes to an Antiinflammatory Phenotype in Cns Autoimmunity,"J Clin Invest 132 (2022):

250. Lim, H.W., S.G. Kang, J.K. Ryu, et al., "Sirt1 Deacetylates Rorgammat and Enhances Th17 Cell Generation,"J Exp Med 212 (2015): 607-17

251. Hisada, R., N. Yoshida, M. Umeda, et al., "The Deacetylase Sirt2 Contributes to Autoimmune Disease Pathogenesis by Modulating Il-17a and Il-2 Transcription,"Cell Mol Immunol 19 (2022): 738-50

252. Woo, S.J., H.S. Noh, N.Y. Lee, et al., "Myeloid Sirtuin 6 Deficiency Accelerates Experimental Rheumatoid Arthritis by Enhancing Macrophage Activation and Infiltration into Synovium,"EBioMedicine 38 (2018): 228-37

253. Daenthanasanmak, A., S. Iamsawat, P. Chakraborty, et al., "Targeting Sirt-1 Controls Gvhd by Inhibiting T-Cell Allo-Response and Promoting Treg Stability in Mice,"Blood 133 (2019): 266-79

254. Toubai, T., H. Tamaki, D.C. Peltier, et al., "Mitochondrial Deacetylase Sirt3 Plays an Important Role in Donor T Cell Responses after Experimental Allogeneic Hematopoietic Transplantation,"J Immunol 201 (2018): 3443-55

255. Ming, M., B. Zhao, C.R. Shea, et al., "Loss of Sirtuin 1 (Sirt1) Disrupts Skin Barrier Integrity and Sensitizes Mice to Epicutaneous Allergen Challenge,"J Allergy Clin Immunol 135 (2015): 936-45 e4

256. Jang, H.Y., D.H. Ha, S.Y. Rah, et al., "Sirtuin 6 Is a Negative Regulator of Fcepsilonri Signaling and Anaphylactic Responses,"J Allergy Clin Immunol 149 (2022): 156-67 e7

257. Ciarlo, E., T. Heinonen, C. Theroude, et al., "Sirtuin 2 Deficiency Increases Bacterial Phagocytosis by Macrophages and Protects from Chronic Staphylococcal Infection,"Front Immunol 8 (2017): 1037
258. Eskandarian, H.A., F. Impens, M.A. Nahori, et al., "A Role for Sirt2-Dependent Histone H3k18 Deacetylation in Bacterial Infection,"Science 341 (2013): 1238858
259. Liu, T.F., V. Vachharajani, P. Millet, et al., "Sequential Actions of Sirt1-Relb-Sirt3 Coordinate Nuclear-Mitochondrial Communication During Immunometabolic Adaptation to Acute Inflammation and Sepsis,"J Biol Chem 290 (2015): 396-408
260. Kim, T.S., Y.B. Jin, Y.S. Kim, et al., "Sirt3 Promotes Antimycobacterial Defenses by Coordinating Mitochondrial and Autophagic Functions,"Autophagy 15 (2019): 1356-75
261. Heinonen, T., E. Ciarlo, E. Rigoni, et al., "Dual Deletion of the Sirtuins Sirt2 and Sirt3 Impacts on Metabolism and Inflammatory Responses of Macrophages and Protects from Endotoxemia,"Front Immunol 10 (2019): 2713
262. Heinonen, T., E. Ciarlo, D. Le Roy, et al., "Impact of the Dual Deletion of the Mitochondrial Sirtuins Sirt3 and Sirt5 on Anti-Microbial Host Defenses,"Front Immunol 10 (2019): 2341
263. Qin, K., C. Han, H. Zhang, et al., "Nad(+) Dependent Deacetylase Sirtuin 5 Rescues the Innate Inflammatory Response of Endotoxin Tolerant Macrophages by Promoting Acetylation of P65,"J Autoimmun 81 (2017): 120-29
264. Springer, M., and S. Moco, "Resveratrol and Its Human Metabolites-Effects on Metabolic Health and Obesity,"Nutrients 11 (2019):
265. Milne, J.C., P.D. Lambert, S. Schenk, et al., "Small Molecule Activators of Sirt1 as Therapeutics for the Treatment of Type 2 Diabetes,"Nature 450 (2007): 712-6
266. Venkatasubramanian, S., R.M. Noh, S. Daga, et al., "Effects of the Small Molecule Sirt1 Activator, Srt2104 on Arterial Stiffness in Otherwise Healthy Cigarette Smokers and Subjects with Type 2 Diabetes Mellitus,"Open Heart 3 (2016): e000402
267. Spallotta, F., C. Cencioni, S. Straino, et al., "A Nitric Oxide-Dependent Cross-Talk between Class I and Iii Histone Deacetylases Accelerates Skin Repair,"J Biol Chem 288 (2013): 11004-12
268. Carrizzo, A., C. Iside, A. Nebbioso, et al., "Sirt1 Pharmacological Activation Rescues Vascular Dysfunction and Prevents Thrombosis in Mthfr Deficiency,"Cell Mol Life Sci 79 (2022): 410
269. Xiang, F., Z. Zhang, Y. Li, et al., "Honokiol Targeting Sirt3: From Molecular Mechanisms to Therapeutic Opportunities,"FASEB J 39 (2025): e70798

270. Zhang, J., L. Zou, D. Shi, et al., "Structure-Guided Design of a Small-Molecule Activator of Sirtuin-3 That Modulates Autophagy in Triple Negative Breast Cancer,"J Med Chem 64 (2021): 14192-216
271. Cho, Y.E., Y. Kim, H. Jo, et al., "Sirtuin 6 Activator Ubcs039 Ameliorates Hepatic Lipogenesis through Liver X Receptor Deacetylation,"Int Immunopharmacol 168 (2026): 115878
272. Ben Lulu, T., D.B. Manikandan, Y. Pevzner, et al., "Pharmacological Activation of Sirt6 Suppresses Progression of Head and Neck and Esophageal Squamous Cell Carcinoma by Modulation of Cellular Metabolism and Protein Translation,"Cell Death Dis 16 (2025): 727
273. Wu, S., J. Zhang, C. Peng, et al., "Sirt6 Mediated Histone H3k9ac Deacetylation Involves Myocardial Remodelling through Regulating Myocardial Energy Metabolism in Tac Mice,"J Cell Mol Med 27 (2023): 3451-64
274. Collins, J.A., C.J. Kim, A. Coleman, et al., "Cartilage-Specific Sirt6 Deficiency Represses Igf-1 and Enhances Osteoarthritis Severity in Mice,"Ann Rheum Dis 82 (2023): 1464-73
275. Qin, T., W. Liu, J. Huo, et al., "Sirt1 Expression Regulates the Transformation of Resistant Esophageal Cancer Cells Via the Epithelial-Mesenchymal Transition,"Biomed Pharmacother 103 (2018): 308-16
276. Chen, G., B. Zhang, H. Xu, et al., "Suppression of Sirt1 Sensitizes Lung Cancer Cells to Wee1 Inhibitor Mk-1775-Induced DNA Damage and Apoptosis,"Oncogene 36 (2017): 6863-72
277. Sussmuth, S.D., S. Haider, G.B. Landwehrmeyer, et al., "An Exploratory Double-Blind, Randomized Clinical Trial with Selisistat, a Sirt1 Inhibitor, in Patients with Huntington's Disease,"Br J Clin Pharmacol 79 (2015): 465-76
278. Gao, Q., L. Yang, S. Ye, et al., "Targeting Sirt2 Induces Mlh1 Deficiency and Boosts Antitumor Immunity in Preclinical Colorectal Cancer Models,"Sci Transl Med 17 (2025): eadv0766
279. Hou, D., T. Yu, X. Lu, et al., "Sirt2 Inhibition Improves Gut Epithelial Barrier Integrity and Protects Mice from Colitis,"Proc Natl Acad Sci U S A 121 (2024): e2319833121
280. Luo, Y., H. Zhao, J. Zhu, et al., "Sirt2 Inhibitor Sirreal2 Enhances Anti-Tumor Effects of Pi3k/Mtor Inhibitor Vs-5584 on Acute Myeloid Leukemia Cells,"Cancer Med 12 (2023): 18901-17
281. Spiegelman, N.A., I.R. Price, H. Jing, et al., "Direct Comparison of Sirt2 Inhibitors: Potency, Specificity, Activity-Dependent Inhibition, and on-Target Anticancer Activities,"ChemMedChem 13 (2018): 1890-94
282. Farooqi, A.S., J.Y. Hong, J. Cao, et al., "Novel Lysine-Based Thioureas as Mechanism-Based Inhibitors of Sirtuin 2 (Sirt2) with Anticancer Activity in a Colorectal Cancer Murine Model,"J Med Chem 62 (2019): 4131-41

283. Jing, H., J. Hu, B. He, et al., "A Sirt2-Selective Inhibitor Promotes C-Myc Oncoprotein Degradation and Exhibits Broad Anticancer Activity,"Cancer Cell 29 (2016): 297-310
284. Wang, B., T. Xu, C. Qiu, et al., "Tenovin-6 Exhibits Inhibitory Effects on the Growth of Sonic Hedgehog (Shh) Medulloblastoma, as Evidenced by Both in Vitro and in Vivo Studies,"Int Immunopharmacol 142 (2024): 113075
285. Molinari, F., A. Feraco, S. Mirabilii, et al., "Sirt5 Inhibition Induces Brown Fat-Like Phenotype in 3t3-L1 Preadipocytes,"Cells 10 (2021):
286. Yan, D., A. Franzini, A.D. Pomicter, et al., "Sirt5 Is a Druggable Metabolic Vulnerability in Acute Myeloid Leukemia,"Blood Cancer Discov 2 (2021): 266-87
287. Le Berre, C., G. Roda, M. Nedeljkovic Protic, et al., "Modern Use of 5-Aminosalicylic Acid Compounds for Ulcerative Colitis,"Expert Opin Biol Ther 20 (2020): 363-78
288. Quan, J., X. Wen, G. Su, et al., "Epithelial Sirt6 Governs Il-17a Pathogenicity and Drives Allergic Airway Inflammation and Remodeling,"Nat Commun 14 (2023): 8525
289. Song, N., X. Guan, S. Zhang, et al., "Discovery of a Pyrrole-Pyridinimidazole Derivative as Novel Sirt6 Inhibitor for Sensitizing Pancreatic Cancer to Gemcitabine,"Cell Death Dis 14 (2023): 499
290. Huang, J., J. Su, H. Wang, et al., "Discovery of Novel Protac Sirt6 Degraders with Potent Efficacy against Hepatocellular Carcinoma,"J Med Chem 67 (2024): 17319-49
291. Christians, U., K. Kohlhaw, J. Budniak, et al., "Ciclosporin Metabolite Pattern in Blood and Urine of Liver Graft Recipients. I. Association of Ciclosporin Metabolites with Nephrotoxicity,"Eur J Clin Pharmacol 41 (1991): 285-90
292. Kim, J.H., D. Kim, S.J. Cho, et al., "Identification of a Novel Sirt7 Inhibitor as Anticancer Drug Candidate,"Biochem Biophys Res Commun 508 (2019): 451-57
293. Kalhori, A., M. Rafraf, R. Navekar, et al., "Effect of Turmeric Supplementation on Blood Pressure and Serum Levels of Sirtuin 1 and Adiponectin in Patients with Nonalcoholic Fatty Liver Disease: A Double-Blind, Randomized, Placebo-Controlled Trial,"Prev Nutr Food Sci 27 (2022): 37-44
294. Daneshi-Maskooni, M., S.A. Keshavarz, M. Qorbani, et al., "Green Cardamom Increases Sirtuin-1 and Reduces Inflammation in Overweight or Obese Patients with Non-Alcoholic Fatty Liver Disease: A Double-Blind Randomized Placebo-Controlled Clinical Trial,"Nutr Metab (Lond) 15 (2018): 63

295. Asghari, S., M. Asghari-Jafarabadi, M.H. Somi, et al., "Comparison of Calorie-Restricted Diet and Resveratrol Supplementation on Anthropometric Indices, Metabolic Parameters, and Serum Sirtuin-1 Levels in Patients with Nonalcoholic Fatty Liver Disease: A Randomized Controlled Clinical Trial,"J Am Coll Nutr 37 (2018): 223-33

296. Ghadimi, M., F. Foroughi, S. Hashemipour, et al., "Decreased Insulin Resistance in Diabetic Patients by Influencing Sirtuin1 and Fetuin-a Following Supplementation with Ellagic Acid: A Randomized Controlled Trial,"Diabetol Metab Syndr 13 (2021): 16

297. Safarpour, P., M. Daneshi-Maskooni, M. Vafa, et al., "Vitamin D Supplementation Improves Sirt1, Irisin, and Glucose Indices in Overweight or Obese Type 2 Diabetic Patients: A Double-Blind Randomized Placebo-Controlled Clinical Trial,"BMC Fam Pract 21 (2020): 26

298. Aghasi, M., F. Koohdani, M. Qorbani, et al., "Beneficial Effects of Green Cardamom on Serum Sirt1, Glycemic Indices and Triglyceride Levels in Patients with Type 2 Diabetes Mellitus: A Randomized Double-Blind Placebo Controlled Clinical Trial,"J Sci Food Agric 99 (2019): 3933-40

299. Sohrab, G., J. Nasrollahzadeh, M. Tohidi, et al., "Pomegranate Juice Increases Sirtuin1 Protein in Peripheral Blood Mononuclear Cell from Patients with Type 2 Diabetes: A Randomized Placebo Controlled Clinical Trial,"Metab Syndr Relat Disord 16 (2018): 446-51

300. Fiore, D., D. Gianfrilli, E. Giannetta, et al., "Pde5 Inhibition Ameliorates Visceral Adiposity Targeting the Mir-22/Sirt1 Pathway: Evidence from the Cecsid Trial,"J Clin Endocrinol Metab 101 (2016): 1525-34

301. Bo, S., G. Togliatto, R. Gambino, et al., "Impact of Sirtuin-1 Expression on H3k56 Acetylation and Oxidative Stress: A Double-Blind Randomized Controlled Trial with Resveratrol Supplementation,"Acta Diabetol 55 (2018): 331-40

302. De Kreutzenberg, S.V., G. Ceolotto, A. Cattelan, et al., "Metformin Improves Putative Longevity Effectors in Peripheral Mononuclear Cells from Subjects with Prediabetes. A Randomized Controlled Trial,"Nutr Metab Cardiovasc Dis 25 (2015): 686-93

303. Sardu, C., G. Pieretti, N. D'onofrio, et al., "Inflammatory Cytokines and Sirt1 Levels in Subcutaneous Abdominal Fat: Relationship with Cardiac Performance in Overweight Pre-Diabetics Patients,"Front Physiol 9 (2018): 1030

304. Wasserfurth, P., J. Nebl, M.R. Ruhling, et al., "Impact of Dietary Modifications on Plasma Sirtuins 1, 3 and 5 in Older Overweight Individuals Undergoing 12-Weeks of Circuit Training,"Nutrients 13 (2021):

305. Abedimanesh, S., S.Z. Bathaie, A. Ostadrahimi, et al., "The Effect of Crocetin Supplementation on Markers of Atherogenic Risk in Patients with Coronary Artery Disease: A Pilot, Randomized, Double-Blind, Placebo-Controlled Clinical Trial,"Food Funct 10 (2019): 7461-75

306. Abedimanesh, N., B. Motlagh, S. Abedimanesh, et al., "Effects of Crocin and Saffron Aqueous Extract on Gene Expression of Sirt1, Ampk, Lox1, Nf-Kappab, and Mcp-1 in Patients with Coronary Artery Disease: A Randomized Placebo-Controlled Clinical Trial,"Phytother Res 34 (2020): 1114-22
307. Saboori, S., F. Koohdani, E. Nematipour, et al., "Beneficial Effects of Omega-3 and Vitamin E Coadministration on Gene Expression of Sirt1 and Pgc1alpha and Serum Antioxidant Enzymes in Patients with Coronary Artery Disease,"Nutr Metab Cardiovasc Dis 26 (2016): 489-94
308. Gautam, S., U. Kumar, M. Kumar, et al., "Yoga Improves Mitochondrial Health and Reduces Severity of Autoimmune Inflammatory Arthritis: A Randomized Controlled Trial,"Mitochondrion 58 (2021): 147-59
309. Khazdouz, M., N.E. Daryani, F. Alborzi, et al., "Effect of Selenium Supplementation on Expression of Sirt1 and Pgc-1alpha Genes in Ulcerative Colitis Patients: A Double Blind Randomized Clinical Trial,"Clin Nutr Res 9 (2020): 284-95
310. Gautam, S., M. Kumar, U. Kumar, et al., "Effect of an 8-Week Yoga-Based Lifestyle Intervention on Psycho-Neuro-Immune Axis, Disease Activity, and Perceived Quality of Life in Rheumatoid Arthritis Patients: A Randomized Controlled Trial,"Front Psychol 11 (2020): 2259
311. Sands, B.E., S. Joshi, J. Haddad, et al., "Assessing Colonic Exposure, Safety, and Clinical Activity of Srt2104, a Novel Oral Sirt1 Activator, in Patients with Mild to Moderate Ulcerative Colitis,"Inflamm Bowel Dis 22 (2016): 607-14
312. Amengual, J.E., S. Clark-Garvey, M. Kalac, et al., "Sirtuin and Pan-Class I/Ii Deacetylase (Dac) Inhibition Is Synergistic in Preclinical Models and Clinical Studies of Lymphoma,"Blood 122 (2013): 2104-13
313. Bisht, S., B. Chawla, M. Tolahunase, et al., "Impact of Yoga Based Lifestyle Intervention on Psychological Stress and Quality of Life in the Parents of Children with Retinoblastoma,"Ann Neurosci 26 (2019): 66-74
